# Relationship between temporal anomalies in PM_2.5_ concentrations and reported influenza/influenza-like illness activity

**DOI:** 10.1016/j.heliyon.2020.e04726

**Published:** 2020-08-15

**Authors:** T.P. DeFelice

**Affiliations:** University of Colorado Boulder, USA

**Keywords:** Atmospheric science, Climatology, Environmental analysis, Environmental health, Public health, Aerosol, PM2.5 concentrations, PM2.5 concentration anomalies, Influenza virus, Influenza and influenza-like illness (ILI), Aerosol-health interactions, Particulate matter(PM)

## Abstract

A small number of studies suggest atmospheric particulate matter with diameters 2.5 micron and smaller (PM_2.5_) may possibly play a role in the transmission of influenza and influenza-like illness (ILI) symptoms. Those studies were predominantly conducted under moderately to highly polluted outdoor atmospheres. The purpose of this study was to extend the data set to include a less polluted atmospheric environment. A relationship between PM2.5 and ILI activity extended to include lightly to moderately polluted atmospheres could imply a more complicated mechanism than that suggested by existing studies. We obtained concurrent PM_2.5_ mass concentration data, meteorological data and reported Influenza and influenza-like illness (ILI) activity for the light to moderately polluted atmospheres over the Tucson, AZ region. We found no relation between PM2.5 mass concentration and ILI activity. There was an expected relation between ILI, activity, temperature, and relative humidity. There was a possible relation between PM2.5 mass concentration anomalies and ILI activity. These results might be due to the small dataset size and to the technological limitations of the PM measurements. Further study is recommended since it would improve the understanding of ILI transmission and thereby improve ILI activity/outbreak forecasts and transmission model accuracies.

## 1 Introduction

A handful of all health-related studies have focused on the relationship between influenza and influenza-like illness (herein collectively denoted as ILI) activity and outdoor atmospheric aerosols. They are mostly associated with moderate to higher aerosol-burdened atmospheric boundary-layer environments. For example, Huang et al. (2016) found a relation between concentration of particulate matter (PM) with diameters of approximately 2.5 microns and smaller (denoted as PM_2.5_) and influenza-like cases in Nanjing, China. Feng et al. (2016) found a particulate matter with effective diameters of 2.5 micron and smaller (PM_2.5_) to be correlated with reported influenza and influenza-like illness (ILI) activity in the atmospheric boundary layer of Beijing. Wang et al. (2019) found a similar relationship, but with allergic rhinitis in a very polluted environment. Chen et al. (2020) found differences in diurnal pattern of PM_2.5_ in different seasons and cities in India. There are still unresolved questions addressing the effect of atmospheric aerosols on virus transmission (e.g., CDC, 2020; Yan et al., 2018; De Longueville et al., 2013; Lemieux et al., 2007).

We sought to pursue a more immediate question, Does the PM_2.5_ and ILI activity relationship only exist in high aerosol burdened or highly polluted boundary-layer air masses? Given the findings from the previous studies one would not expect that a similar relationship exists in a low to moderate aerosol-burdened atmospheric boundary-layer environment such as in the Tucson, Arizona area, United States all other transmission modes notwithstanding. A more comprehensive relation between PM and ILI activity can help to improve the understanding of ILI transmission and thereby improve ILI activity/outbreak forecasts and transmission model accuracies. The latter might include events like the recent global coronavirus outbreak, which presents some influenza-like illness symptoms (Center for Disease Control and Prevention, CDC, 2020).

The remainder of this section, primarily Section 1.1, contains general details to support the understanding of the data and how they might inter-relate. Section 2, Results and Discussion, includes conditions of the study area, the non-obvious methods and data characteristics to support the discussion of the relationship between ILI activity and environmental parameters, i.e., air temperature, relative humidity and wind field; PM concentrations versus ILI data; PM_2.5_ concentration temporal anomalies and ILI activity; followed by a more detailed look at larger scale windfield versus ILI activity. There is an on-line resource referenced to support this study. A special section 2.1 is included to address the need for improved technology dedicated to explicitly measuring or observing the physical, including morphological, attributes of individual aerosols plus their concurrent chemical composition. Section 3 contains the Conclusions and recommendation for future study, Section 4 contains Acknowledgements and Section 5 contains the references.

### 1.1 Background

Aerosols and other atmospheric constituents may be considered vectors for the transport of viruses, influenza, and influenza-like illnesses (e.g., CDC, 2020; Sajadi et al., 2020; CDC, 2018; World Health Organization, 2018; Fröhlich-Nowoisky et al., 2016; Linares et al., 2015; Landlová et al., 2014; Abraham and Baird, 2012; Mubareka et al., 2009; Tellier, 2006; McCarthy, 2001; Knight, 1980; Donaldson and Ferris, 1975; Knight, 1973; Benbough and Hood, 1971), bacteria and fungi and pathogenic microorganisms (e.g., Griffin, 2007; Williamson et al., 2003; Griffin et al., 2001), organic contaminants such as pesticides, and toxic elements and compounds from natural and anthropogenic sources. Culture or spore-counting techniques have verified that a wide range of dust-borne pathogenic microorganisms survive the physical stresses, (namely, exposure to UV radiation, desiccation, temperature, and phase changes associated with atmospheric water, especially into the ice phase), experienced during global-scale transport (e.g., Griffin, 2007; Griffin et al., 2001). Griffin et al. (2001) used a direct-count assay (i.e., use of a nucleic acid stain to count microorganisms via epifluorescence microscopy) of the virus-like particles sampled over the U.S. Virgin Islands. They reported a background virus-like concentration of ~1–50 L^−1^, which is enough to initiate the ice crystal precipitation process under the right environmental conditions, i.e., at sufficient relative humidity and air temperatures as high as 271 K or -2 °C per Vali (1996). As a matter of comparison, most non-bacteria biogenic nuclei, including pollens, typically nucleate ice around 261 K or -12 °C and lower. A good foundation on biological influence in the phase change of water to ice is summarized in Fröhlich-Nowoisky et al. (2016), Wiedinmyer et al. (2009), Von Blohn et al. (2005), Vali (1996) and Lee et al. (1995).

It is not clear if, why or how some influenza, viruses, infectious agents, and biogenic species, including dust-borne pathogenic microorganisms might survive transport through our atmosphere (e.g., Fröhlich-Nowoisky et al., 2016; Chen et al., 2010; Liu et al., 2009; Griffin, 2007; Tellier, 2006; Perdue and Swayne, 2005; Von Blohn et al., 2005; McCarthy, 2001; Donaldson and Ferris, 1975; Benbough and Hood, 1971; May et al., 1969). Von Blohn et al. (2005) hypothesized that the biological reason for the ice nucleating ability of pollens might be related to a freezing intolerance, i.e., the pollen's extracellular freezing response initiated freezing to protect the interior of the cells when air temperatures fall below freezing. A standing question resurfaces, i.e., Do airborne viruses, especially influenza and influenza-like illness viruses, go dormant (i.e., become inactive) during atmospheric transport, whether in cloud or not, and remain in that state until sometime after that virus lands in a ‘nurturing’ environment that transforms its non-dormant state into a metabolically active state? Despite the importance of the question and some popular known events that collectively might support an answer, i.e., chicken-pox virus becoming shingles later in a human's life, the findings of Yan et al., (2018) and recently the COVID-19 virus (CDC, 2020), the answer remains beyond this paper.

#### 1.1.1 Environmental factors

Environmental factors (e.g., air temperature, relative humidity, windfield, air quality, air pollutants), and/or aerosol characteristics (e.g., size, concentration, composition, anomalies) may play a role in the interactions between and impact realized from influenza and influenza-like illness (ILI) activity, cardiovascular- pulmonary- and respiratory-relevant issues and their transmission (e.g., de Aguiar Pontes Pamplona et al., 2020; Liu et al., 2020; Benaissa et al., 2019; Luong et al., 2019; Mulenga and Siziya, 2019; Su et al., 2019; Wang et al., 2019; Yang et al., 2019; Zhang et al., 2019; Dzullkiflli et al., 2018; Hamanaka and Mutlu, 2018; Hart et al., 2018; Horne et al., 2018; WHO, 2018; Yan et al., 2018; Lin et al., 2017; Feng et al., 2016; Wang et al., 2016; Xing et al., 2016; Zwozdziak et al., 2016; Luo et al., 2015; Morman and Plumlee, 2013; Noti et al., 2013; Prospero and Mayol-Bracero, 2013; Yin et al., 2013; Chen et al., 2010; Shaman et al., 2010; Liao et al., 2009; Liu et al., 2009; Shaman and Kohn, 2009; Mubareka et al., 2009; Tellier, 2009; Liu et al., 2007; Pope et al., 2006; Tellier, 2006; WHO, 2006; Perdue and Swayne, 2005; Pöschl, 2005; Thompson et al., 2004; McCarthy, 2001; Sugaya et al., 2000; Pope, 1991; Barker, 1986; Knight, 1973). The severity of the effect seems to depend on the amount and duration of the exposure, the physical and chemical characteristics of the particulate matter (PM) and the health of and environmental stressors on the exposed individuals (e.g., Davidson et al., 2005).

Virus activity is reduced as humidity is increased and during cloudy/foggy periods (e.g., Noti et al., 2013; Yang et al., 2012; Myatt et al., 2010; Shaman and Kohn, 2009). It is unclear whether the latter is due to their scavenging into the cloud/fog droplets, and/or to their ‘defense mechanism’ (e.g., Engelbrecht et al., 2009; Griffin, 2007; Von Blohn et al., 2005). The survival times of the influenza virus are generally increased as the humidity is lowered (e.g., Noti et al., 2013; Yang et al., 2012; Myatt et al., 2010; Shaman and Kohn, 2009). Influenza virus organisms generally seem able to survive for at least 0.5–2 days indoors once airborne and 1–7 days indoors once deposited on surfaces (e.g., Atkinson and Wein, 2008; Thomas et al., 2008; Tellier, 2009; Tellier, 2006).

#### 1.1.2 Particulate matter

The typical aerosol measurements obtained in respiratory effects studies are from high volume air flow samplers equipped with size cutoff inlets typically set at 10-micron diameters and 2.5-micron diameters. They provide near surface, bulk mass information for all collected atmospheric aerosols and other airborne constituents. They are usually made over a 12 h period to collect enough mass for chemical analyses. the duration of sampling is dependent on the aerosol loading. The a-priori measurements are commonly referred to as particulate matter (PM) measurements. Measurements of the bulk mass measurement of particulate matter with 2.5-micron diameters and smaller are referred to as PM_2.5_, and similarly labeled for other size cutoffs. Such samplers without a size cutoff inlet obtain what are commonly termed total suspended particulate measurements. The PM_2.5_ data used in this study were obtained from the Environmental Protection Agency (EPA) national network of air monitoring sites. These data were quality assured and obtained for the Tucson area during the same periods in Table 1.

**Table 1:** Monthly meteorological data for Tucson during October–March 2005–2007, and calendar year 2009.

Tucson, AZ	T (°C)	Td (°C)	Vis. (km)	SLP (mb)	SP (mb)	RH (%)	Wind Speed (m/s)	Wind Direction (°)	Precipitation (cm)	Max T (°C)	Min T (°C)
October (2005)	22.4	4.7	70	1011.4	919.6	28	7.2	174	0.79	35.0	8.9
November (2005)	16.9	–4.6	56	1015.9	922.5	23	6.2	177	0	31.1	-2.8
December (2005)	11.8	–6.7	54	1017.7	923.6	29	4.9	183	0.03	26.1	-1.1
January (2006)	12.1	–9.5	54	1017.8	923.6	23	6.2	180	0	26.1	-1.1
February (2006)	14.3	–9.2	56	1015.8	922.2	21	6.1	174	0	27.8	1.1
March (2006)	14.8	–4.4	56	1013.3	920.1	29	6.7	194	1.47	27.8	0.0

October (2006)	21.0	3.5	57	1011.7	919.4	35	5.4	175	0.69	36.1	7.2
November (2006)	17.0	–4.1	36	1014.7	921.4	25	4.8	186	0	30.0	-1.7
December (2006)	9.8	–5.3	16	1019.2	923.3	39	5.3	180	1.57	24.4	-2.8
January (2007)	8.9	–2.3	15	1017.6	922.3	55	5.0	187	1.80	23.3	-6.7
February (2007)	12.9	–3.8	16	1016.5	922.3	35	5.8	201	0.10	26.1	-1.1
March (2007)	17.4	–6.3	16	1014.6	921.3	24	7.2	194	1.50	34.4	-0.6

January (2009)	13.1	-1.3	16	1019.5	929.8	37	2.5	130	1.60	20.6	5.5
February (2009)	14.1	-3.7	16	1016.5	928.1	29	2.9	180	1.42	22.5	5.6
March (2009)	17.3	-6.9	16	1012.8	925.1	18	3.2	220	0.46	25.6	9.1
April (2009)	19.4	-6.6	16	1011.4	924.4	17	3.7	220	0.74	27.8	11.1
May (2009)	26.7	-0.1	16	1009.0	923.4	17	3.0	210	1.70	34.9	18.5
June (2009)	28.3	1.9	16	1007.7	922.3	18	3.2	220	0.03	36.0	20.5
July (2009)	32.3	13.8	16	1010.0	925.1	33	3.1	200	4.52	39.2	25.3
August (2009)	31.5	9.6	16	1010.0	924.7	26	3.0	190	0.84	38.8	24.1
September (2009)	28.7	8.6	16	1010.4	924.7	28	3.2	160	1.88	35.8	21.6
October (2009)	21.0	-0.4	16	1010.0	923.4	24	3.0	210	0.13	28.7	13.2
November (2009)	18.0	-3.0	16	1013.8	926.1	24	3.1	140	0.33	25.9	10.1
December (2009)	10.4	-2.3	16	1015.5	926.7	41	2.9	160	0.76	17.4	3.4

Note: SP = station pressure, SLP = sea level pressure, Td = dew point temperature, Vis = visibility.

The size distribution, morphology, and even the chemical composition of aerosols, or particulate matter, within the inhaled air are important for predicting the respiratory impact. Since the smaller the aerosol, the deeper their penetration might be into the human system assuming phase changes or possible internal growth are not significant enough to alter aerosol size or trajectory. Many atmospheric aerosols have sizes between 0.1 and 1 micron, also termed the Greenfield gap. These aerosols are too large to diffuse rapidly to a surface (e.g., lining of lung, earth's surface), and too small to settle out rapidly or deposit because of inertial effects. Since the fraction of aerosols deposited on the lining of a lung is a function of aerosol size, aerosol shape, lung geometry and airflow patterns, most Greenfield gap aerosols would likely reach the deepest parts of our lungs and not readily deposit on its lining. Zwozdziak et al. (2016) observed a greater negative health effect on lung function parameters from particulate matter with diameters of 1 micron and smaller (PM_1_), compared to same with diameters of 2.5 micron and smaller (PM_2.5_).

Bulk values of aerosol effective size (diameter or radius), aerosol number distribution, mass distribution and aerosol optical thickness are available independent from the PM measurements (e.g., O'Neill et al., 2008; Sinyuk et al., 2007; Dubovik et al., 2006; Dubovik, 2004; Holben et al., 2001; Dubovik and King, 2000). These data can help provide insight into the distribution of sizes within the population of particulate matter collected in the PM samples. They were obtained from the Tucson, AZ AERONET site (32.233° N, 110.953° W, Elevation: 779.0 m) during the periods covered in Table 1. This site best represents the area covered by the EPA and NOAA data used in this paper. The AERONET network contains standard instrumentation (sun photometers), calibration, processing, and distribution protocols (aeronet.gsfc.nasa.gov/new_web/index.html). However, these data are only available when there is a discernible solar disc. There are no available data when the sky covered by optically thick clouds, after dark and before sunrise. The volume or mass distribution is plotted versus diameter to generally indicate aerosol sources and sinks, which can be helpful when trying to understand aerosol population dynamics, or the evolution of size and composition within their population during sampling. The curve shapes could be influenced by; sample size (number of available data points), total aerosols and/or water vapor in column(s) viewed, zenith angle, small data sizes, temporal and spatial mismatches between the data, and the cloud screening algorithm. However, since they are obtained from sun photometers they might not be indicative of the near surface aerosol population. DeFelice and Wylie (2001) suggest the existence of distinct spectral signatures for clear skies, sub-visual cirrus, and optically thin clouds, for example, that would help minimize false interpretations. Identifying sky conditions when using the optical aerosol data helps reduce interpretation errors. For example, a sun photometer measurement may be assigned as being associated with boundary-layer aerosol, even though it was from sub-visual cirrus. Boundary-layer dust is more likely to influence human health, than might the sub-visual cirrus, for example.

#### 1.1.3 Influenza and influenza-like illness activity

Influenza activity impacts up to at least 20% of the U.S. population and is responsible for millions of cases of severe illness and hundreds of thousands of deaths worldwide on the average each year (CDC, 2018; WHO, 2018). Influenza and influenza-like illness (collectively termed ILI) microorganism behavior may be a function of environmental conditions, virus or microorganism type, and/or geographical region (e.g., WHO, 2018; Greene et al., 2006). Influenza and influenza-like illness virus transmission among humans has been studied and modeled (e.g., Yan et al., 2018; Norowitz et al., 2016; Richard and Fouchier, 2016; Yang et al., 2012; Huo et al., 2012; Stilianakis and Drossino, 2010; Lowen and Palese, 2009; Mubareka et al., 2009; Atkinson and Wein, 2008; Lowen et al., 2008; Lowen et al., 2007), but no specific wintertime maximum causal factors have been identified (e.g., Huo et al., 2012; Lofgren et al., 2007). Perhaps because influenza may play a role in many types of illnesses, allergic rhinitis, heart attacks, strokes, and respiratory distress (e.g., Wang et al., 2019; MacIntyre et al., 2013; Greene et al., 2006; Dushoff et al., 2006; Reichert et al., 2004; Madjid et al., 2003), or because multiple respiratory viruses may circulate concurrently in the population and account for a large proportion of influenza-like illness (Huo et al., 2012). Further, the results from Yan et al. (2018) might suggest another mode for ILI transmission, which might provide some relevant insight into current influenza-like illness viral outbreaks, such as COVID-19 (CDC, 2020; Asadi et al., 2020; Drossinos and Stilianakis, 2020; Pani et al., 2020). Yan et al. (2018) found influenza virus in exhaled breath from community-acquired influenza cases during natural breathing, prompted speech, coughing and sneezing for example.

## 2 Results and Discussion

**Figure 1:**
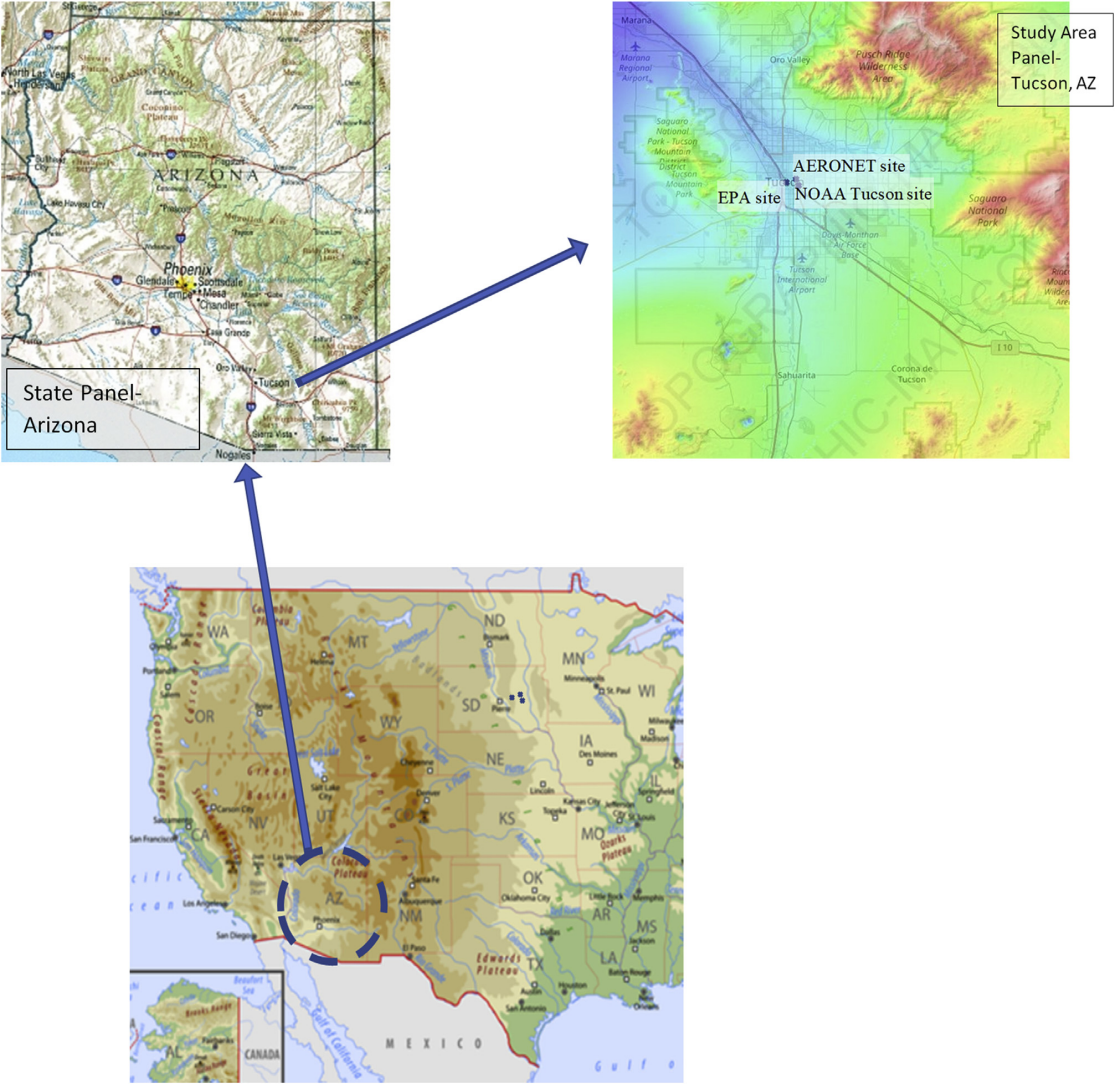
Topographical Map of the USA west of the Mississippi River, stepped to the state of Arizona (State panel-Arizona), and to the study area (Study Area Panel-Tuscon, AZ). (Sources: USGS online free map site, [Study Area Panel – Tucson, AZ], http://www.wanderlustwonder.com/wp-content/uploads/2012/04/topographic-map-of-arizona.jpg [State panel-Arizona(AZ)], and world atlas-https://www.worldatlas.com/img/us-map/physical-us-map.pngwww.worldatlas.com/img/us-map/physical-us-map.png [United States of America (USA) west of the Mississippi River Panel]).

The study area (Figure 1) consists of Tucson regional meteorological data, the particulate matter data from the Tucson area US EPA air quality site and supplemental aerosol optical property data from the University of Arizona AERONET sunphotometer site (32.233° N, 110.953° W, Elevation: 779.0 m). The reported influenza and influenza-like illness (ILI) data were obtained from the Google Flu web site. Daily meteorological data and co-available aerosol data were obtained for three meteorologically distinct peak influenza seasons, namely, the 2005–2006 and 2006–2007 winter seasons, and throughout 2009 (Table 1). The meteorological data were processed and grouped by seasons and then inter-compared using standard techniques. For example, the January–March 2006 period was the hottest and driest (of all periods shown), despite a mean wind direction that is similar to that during 2009. The same period is also cooler by 14–17° than that in 2007. The wind speeds were the lowest for 2009 compared to 2006 and 2007, which were virtually identical. It appears that the largest October–November temperature drop occurred during 2006, and the largest November–December temperature drop occurred in 2009. The largest January–February and February–March temperature increases occurred in 2007. This despite the average temperature during January–March 2007 not being the warmest, which might partially be due to the corresponding change in average wind direction. Table 1 shows the 2006/7 season as wetter than the 2005/6 season, despite both seasons having a similar mean air temperature. These results are at least qualitatively consistent with the relation found by Hondula et al. (2013), namely, with respect to environmental factors and influenza/influenza-like illness activity. The data and analyses included corresponding 850 mb flow charts from the National Oceanic and Administration over North America which will be discussed below.

Estimated weekly influenza and influenza-like illness (ILI) activity reported per 100,000 physician visits were obtained to provide a proxy for the influenza and influenza-like activity. Ginsberg et al. (2009) describe the aforementioned dataset and how it estimates the probability that a random physician visit in a particular region or state is related to an influenza and influenza-like illness (i.e., percentage of ILI-related physician visits), and how they validate their system performance. It was assumed that a physician office visit for influenza and/or influenza-like illness resulted from a susceptible person being exposed to the influenza virus and/or a microorganism causing influenza-like symptoms, contracting the flu or presenting flu-like symptoms, and becoming ill enough to warrant a physician or hospital doctor visit. It was also assumed that there would be a lag between the environmental condition(s) as defined above (Section 2) that increase(s) the rate of physician visits and the actual visits. Ginsberg et al. (2009) further caution that some of the data used were estimates. Consequently, these data are not a replacement for traditional surveillance networks nor are they to supplant the need for laboratory-based diagnoses and surveillance. The intended use herein of these data is as guidance.

**Figure 2:**
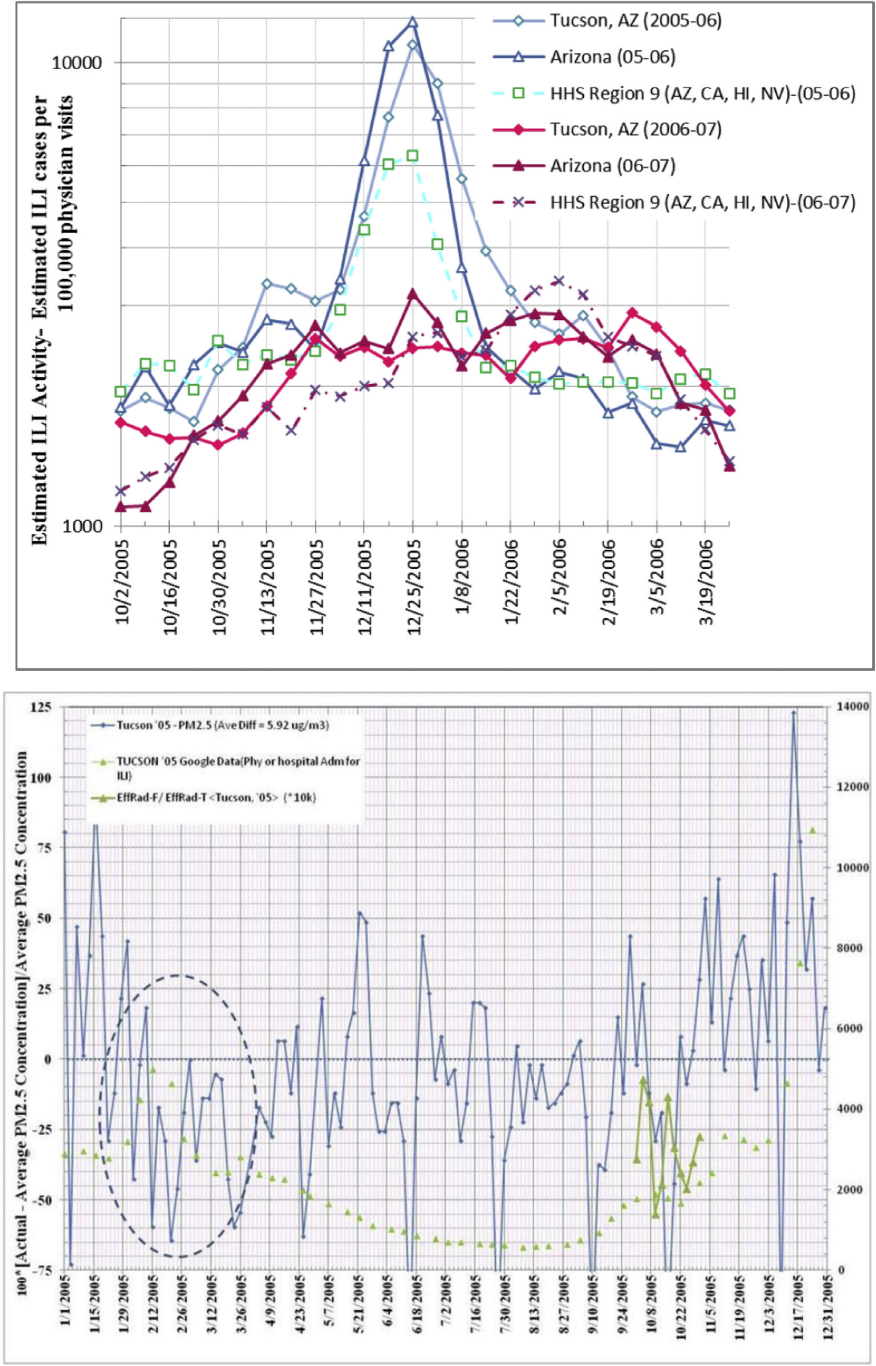
**a.** The estimated Influenza-like illness cases per 100,000 physician visits for Tucson and Arizona compared to those from USA Health and Human Services (HHS) Region 9 for the 2005–2006 flu seasons. (Data Sources: Arizona Department of Health Services; Google Flu Trends-www.google.org/flutrends). **b.** The anomaly in the actual PM_2.5_ concentrations for Tucson during 2005 calendar year. Also shown are the corresponding ratio of effective radii fine mode versus total population for Tucson and the estimated Physician reported cases of influenza-like illnesses (right axis) for Tucson during the respective periods. (Data Sources: NASA AERONET; PM data obtained through http://www.epa.gov/ttn/airs/airsaqs/; Google Flu Trends; www.google.org/flutrends).

**Figure 3:**
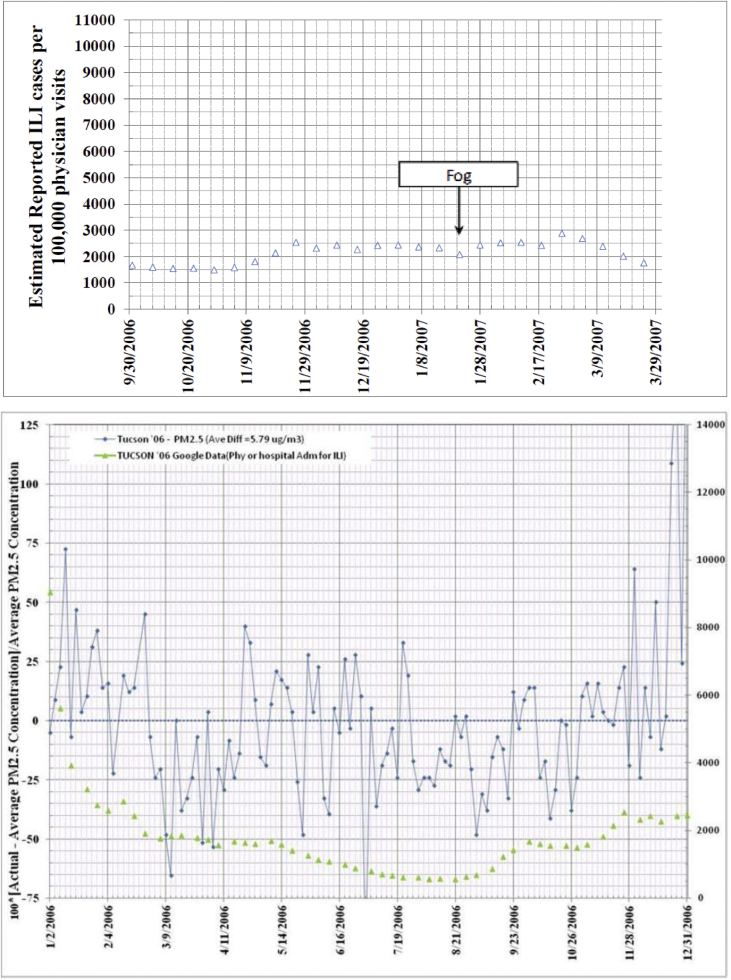
**a.** The estimated Influenza-like illness cases per 100,000 physician visits for Tucson for the 2006–2007 flu seasons. Note-The beginning day for the weekly 2006–2007 season data points is a day sooner than indicated, and Arizona and USA Health and Human Services (HHS) region records did not add any different insight. (Data Sources: Arizona Department of Health Services; Google Flu Trends-www.google.org/flutrends). **b.** The anomaly in the actual PM2.5 concentrations for Tucson during 2006 calendar year. Also shown are the corresponding ratio of effective radii fine mode versus total population for Tucson and the estimated Physician reported cases of influenza-like illnesses (right axis) for Tucson during the respective periods. The Tucson AERONET site effective radii ratio data for 2006 were unavailable. (Data Sources: NASA AERONET; PM data obtained through http://www.epa.gov/ttn/airs/airsaqs/; Google Flu Trends; www.google.org/flutrends).

**Figure 4:**
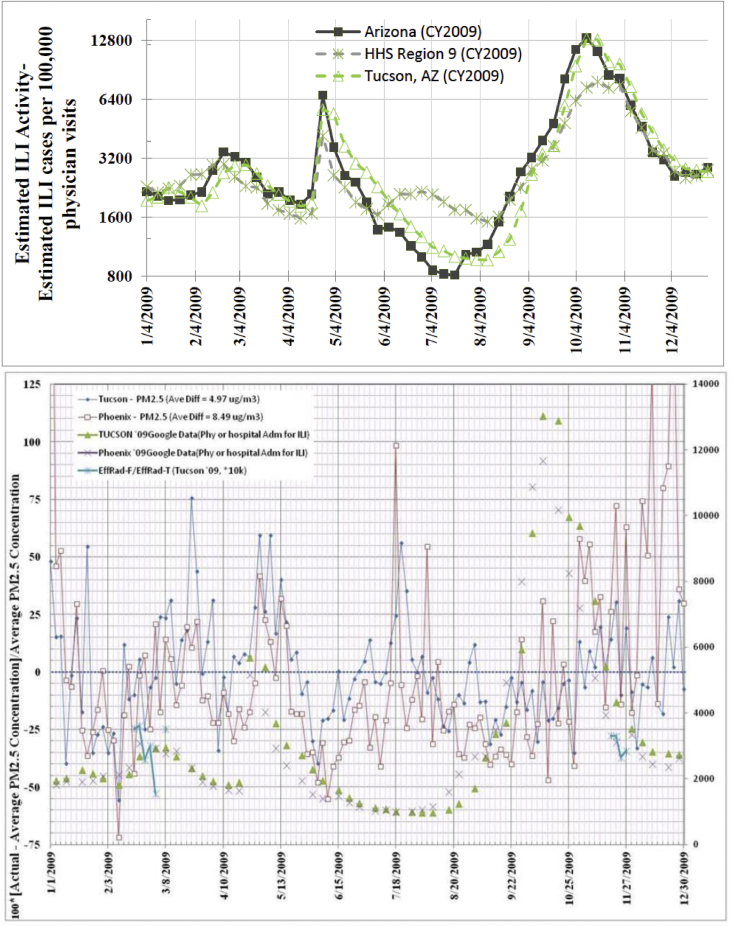
**a.** The estimated Influenza-like illness cases per 100,000 physician visits for Tucson and Arizona compared to those from USA Health and Human Services (HHS) Region 9 for the 2009 calendar year. (Data Sources: Arizona Department of Health Services; Google Flu Trends-www.google.org/flutrends). **b.** The anomaly in the actual PM_2.5_ concentrations for Tucson during 2009 calendar year. Also shown are the corresponding ratio of effective radii fine mode versus total population for Tucson and the estimated Physician reported cases of influenza-like illnesses (right axis) for Tucson during the respective periods. (Data Sources: NASA AERONET; PM data obtained through http://www.epa.gov/ttn/airs/airsaqs/; Google Flu Trends; www.google.org/flutrends).

The ILI, meteorological (Table 1) and aerosol data from the Tucson AZ region were collected, quality assured and analyzed for the following a-priori determined 2005–2006 flu season (Figure 2), 2006–2007 flu season (Figure 3) and the 2009 calendar year (Figure 4).

The comparison of the estimated influenza and influenza-like illness (ILI) activity per physician reported visits (Figures 2a, 3a, 4a) and the meteorological data (Table 1) suggest an ideal set of periods to assess the possible relationship between aerosol - influenza and influenza-like (ILI) symptom activity (i.e., ILI peak in 2005-6, ILI peaks in May, October and November 2009). They also appear to confirm the known relation between ILI activity and environmental conditions. For example, the observed 2006-7 season estimated influenza-like illness (ILI) activity exhibits a temporal pattern similar to that typically found in the tropical latitudes, whereas the 2005-6 season estimated ILI activity exhibits a temporal pattern similar to that typically found in the middle northern hemisphere latitudes (World Health Organization, 2018). Yang et al. (2012), for example, provide a mechanistic explanation for the tropical and non-tropical geographic regional relationship between influenza activity and humidity. Noti et al. (2013) also conclude that the long residence time of aerosols smaller than 4 microns are tempered by rapid inactivation during high humidity. The different patterns imply a possible influence from different air-masses or environmental conditions (e.g., different air flow patterns that may or may not mean different aerosol population characteristics). We extracted more specific features that relate ILI activity to environmental conditions (e.g., temperature and humidity). The period between January 21–23, 2007 (Figure 3a) indicates a reduction in the estimated ILI activity reported during obscured (OBS) skies (Table 2). There was a concurrent shift in wind direction. The obscurant was fog. The ILI reports did rebound following fog dissipation, which appears consistent with other studies (e.g., Hondula et al., 2013; Noti et al., 2013; Yang et al., 2012; Myatt et al., 2010; Shaman and Kohn, 2009). Further investigation reveals that the concurrent air temperatures were approximately 270–271 K. Such air temperatures are thresholds for triggering the ice crystal process and might possibly initiate a dormancy process in the ILI microorganism(s), (e.g., Wiedinmyer et al., 2009; Von Blohn et al., 2005; Vali, 1996; Lee et al., 1995). The biogenic species or whether its internal protective process was active is not known. Whether the fog, or the passage of the weather system that triggered the fog, disrupted the pseudo-steady state boundary-layer aerosol population and caused the reduction in estimated ILI activity or contributed to the initiation of ILI microorganism dormancy process remains undetermined, and the focus for a more comprehensive future study.

**Table 2:** Summary of the monthly meteorological cloud ceiling and cloud amount observations for Tucson during October–March 2005–2007, and calendar year 2009.

Tucson	Ave CLG (m)	Ave STDEVP CLG (m)	CLR	SCT	BKN	OVC	OBS	N
October (2005)	18854	6665	125	206	75	1	0	407
November (2005)	19079	6000	236	335	128	10	0	709
December (2005)	16549	7451	142	311	173	77	0	703
January (2006)	17918	6902	226	309	179	11	0	725
February (2006)	15128	7829	149	228	253	44	0	674
March (2006)	12985	8726	113	244	294	102	0	753

October (2006)	20247	5598	638	96	48	25	0	807
November (2006)	21515	3137	754	5	8	11	0	778
December (2006)	18735	7534	665	47	47	88	0	847
January (2007)	17189	8713	608	50	51	140	11∗	860
February (2007)	19832	6204	630	59	45	40	0	774
March (2007)	20723	4927	773	30	20	35	0	858

January (2009)	19207	6963	711	40	45	78	0	874
February (2009)	20140	5921	691	19	24	47	0	781
March (2009)	21174	3963	798	29	22	15	0	864
April (2009)	21504	3165	791	25	13	8	0	837
May (2009)	20774	4771	785	30	22	33	0	870
June (2009)	21582	2817	775	42	17	2	0	836
July (2009)	20872	4613	801	80	33	21	0	935
August (2009)	21265	3747	797	55	22	12	0	886
September (2009)	21088	4131	779	58	27	15	0	879
October (2009)	21223	3841	764	61	19	16	0	860
November (2009)	19207	6963	745	28	26	33	0	832
December (2009)	20140	5921	663	42	41	89	0	835

∗ Obscured (Fog) Jan 22, '07 for 2.5 h beginning 1030 am, Ave winds 14007, Ave T = 27.5F, Ave Td = 27.2F, Ave SLP = 1016.75 (1017.6@1255) mb; Ave SP = 955.7727mb, Ave PCP01 = 0 inches, AvePCP24 = 0.37 inches.

The PM_2.5_ concentration data points were not clearly related to the corresponding estimated influenza and influenza-like Illness (ILI) physician reported case values. They do appear to generally vary with season. Chen et al. (2020) also found differences in diurnal pattern of PM_2.5_ in different seasons and cities in India. This was also found for PM_10_ data points plotted versus the estimated ILI physician reported case values. However, the lack of a relationship between PM_2.5_ and ILI is different than that found by Huang et al. (2016) and Feng et al. (2016) who sample during comparatively more polluted atmospheric conditions. Wang et al. (2019) also found a relationship between PM_2.5_ and allergic rhinitis in a very polluted environment. The small dataset size is surely a contributing factor to our results, but there are likely other concurrent contributing factors. No PM composition data are available to address the effect of composition on respiratory defense mechanism efficiency (e.g., Engelbrecht et al., 2009; Griffin, 2007). There is still the possibility that this result was due to varying sizes and composition within the particulate matter population related with the evolution of a pseudo-steady state boundary-layer aerosol population, and/or with the long range transport of ILI micro-organisms (e.g., Sajadi et al., 2020; Guo et al., 2019; Nakata et al., 2013; Roelofs, 2013; Salvador et al., 2013; VanCuren et al., 2012; Griffin, 2007; Tafuro et al., 2006; Perry et al., 1997; Pruppacher and Jaenicke, 1995; Hoppel et al., 1989, 1990).

Since humans respond more to the relative conditions (e.g., Hondula et al., 2013), the relationship with influenza and influenza-like illnesses and particulate matter anomalies was investigated. Where available, based on this data analysis, the AERONET sunphotometer data-derived effective radii ratio was included for Tucson during 2005 (Figure 2b), 2006 (Figure 3b) and 2009 (Figure 4b). A positive anomaly in PM_2.5_ mass concentrations implies a general buildup of particulate matter in the region (compared to the corresponding typical amount), which could imply a transition to a more polluted state with respect to particulate matter. It might also support the notion of stagnating air (e.g., cold-base or stably-stratified boundary-layer, large scale subsidence inversion over the region), composition including virus cycling, and increased probability of inhaling influenza and influenza-like illness (ILI) microorganisms. The estimated ILI activity reports during the corresponding periods were overlaid for perspective. These curves indicate that the transition period to positive anomaly values, especially contiguously following a negative anomaly period, is associated with the transition to increased influenza-like illness activity (e.g., mid-late December in Figure 2b, late November in Figure 3b and late February in Figure 4b, and early October in Figure 4b). The first half of February 2005 (Figure 2b, hatched oval) appears to be an exception, despite the increasing estimates of ILI throughout. Data prior to and during this period are limited.

The corresponding meteorological data indicated a concurrent cooling and the early stages of a large-scale high-pressure area influencing Tucson and the surrounding region by middle February 2005 as shown in Online Resource 1 (i.e., “DeFelice-PM-ILI-HELIYON-OnlineResource 1”Available on Request). But the large-scale high-pressure feature breaks down by end of February 2005, likely disrupting the evolution of a pseudo-steady state aerosol population, and/or dispersing or de-activating the high influenza and influenza-like illness virus concentration. The peak during late April - early May 2009 might have been caused by a new flu strain or one not well covered, if at all, by the flu vaccine administered. The beginning of the increased ILI period during late October 2005 was dominated by predominantly coarse-mode sized aerosols.

**Figure 5:**
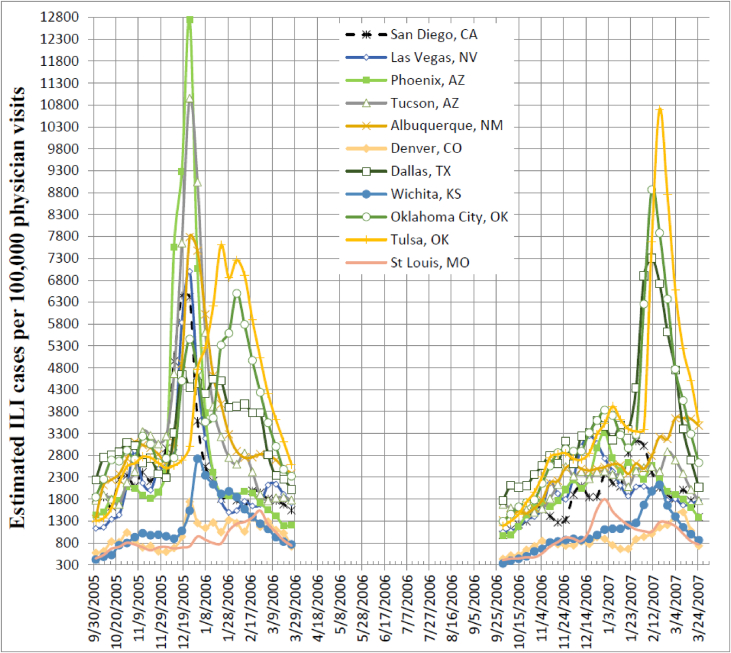
Temporal variation of reported estimated ILI activity for various metropolitan areas in the western south-central United States during 2005–2006 and 2006–2007 influenza seasons.

The question, is it possible for viruses to be transported long distances in our atmosphere under the right conditions, was revisited by comparing the temporal variation in the estimated ILI activity per 100,000 physician visits for 11 states across the southern to mid-western United States for the 2005 through 2007 seasons (Figure 5). Looking at the transect from San Diego, Phoenix, Tucson through Dallas through Oklahoma City, Tulsa and to a lesser extent St Louis, there appears to be a shift in the maximum ILI activity reported from December through January (Dallas; albeit weak), through early February. The focus is on when and where the peaks occur and not magnitude. The magnitude relies on the different thresholds for behavioral practices, and other local factors related to reporting and seeking medical attention for influenza-like symptoms. The data in Figures 2, 3, 4, and 5 have not been normalized to remove such biases. Therefore, the absolute numbers of physician reports are not being intercompared, nor shall they be.

One can see a tendency for a temporal shift from a near-concurrent San Diego CA, Las Vegas NV, Tucson AZ, Phoenix AZ, Albuquerque NM curve maxima in December (’05) to a near-concurrent Tulsa OK, Oklahoma City OK (OKC), Wichita KS curve maxima in January/February (’06) followed by a St Louis MO curve maximum in late February/March. The 2006–2007 period is clearly different as noted a-priori. Essentially all sites, except possibly Albuquerque, show a small curve maximum in December (’06)/January (’07). There appears to be a near-concurrent Dallas-Ft Worth and OKC to Tulsa OK and Wichita, KS curve maxima temporal shift, and a late season near-concurrent Tucson, Albuquerque, and Denver curve maxima temporal shift. Other factors including low-level air transport may be involved.

The 850 mb flow charts from the National Oceanic and Atmospheric Administration over North America were obtained. They were mainly used to explore the possibility that the temporal shift in the influenza and influenza-like illnesses (ILI) cases shown in Figures 1, 2, 3, and 4 could have been caused by low-level air transport. The 850 mb level is the closest standard level to the surface. It is commonly used to gauge low-level moist air advection. Physician reported influenza and influenza-like illness (ILI) activity data for 16 metropolitan areas were overlaid on the 850 mb charts for North America during the 2005–2006 season, 2006–2007 seasons and calendar year 2009 and are given in Online Resource 1 (i.e., “DeFelice-PM-ILI-HELIYON-OnlineResource 1”, Available on Request). There is an expected linkage between influenza and influenza-like illness (ILI) activity and environmental factors, particularly air temperature and humidity, i.e., colder air temperatures as well as drier air advection and higher estimated influenza activity. There is no discernable connection between a shift in the maximum peak in reported ILI (Figure 5) and atmospheric transport possibly due to the small dataset used in this study or a smaller scale phenomenon contributing to the non-correlation.

A positive anomaly in PM_2.5_ mass concentrations implies a general buildup of particulate matter in the region (compared to the corresponding baseline amount), which could imply a transition to a more polluted boundary-layer atmosphere with respect to particulate matter. Polluted environments as suggested by Huang et al. (2016), Feng et al. (2016), Wang et al. (2019) show a relationship between PM and ILI. They possess an increased probability of inhaling influenza and influenza-like illness (ILI) microorganisms. The PM population over time can become pseudo-stationary (e.g., Pruppacher and Klett, 1997), especially in a stably stratified boundary-layer. The aforementioned occurs from processes that replenish the smallest PM (e.g., gas to particle conversion, coagulation, diffusion) concurrently with processes that remove the largest PM of the aerosol population (e.g., coagulation, more mechanical processes, dry deposition, rainout and washout). Further it is possible that this population concurrently experiences condensation-evaporation cycles (even if to simply manifest as haze). These collectively support the chemical composition, including virus microorganism, cycling throughout the population. The PM from long range transport and local sources including humans contribute to the composition changes. The ILI reported activity then could increase as the number of ingested ILI microorganisms increase. Alternatively, the ILI reported activity and/or the concentration cycling result could remain relatively constant while the environmental conditions stress the human immune system to a point that triggers the ILI or other microorganism activity. The latter could support the findings of Huang et al. (2016), Feng et al. (2016), and Wang et al. (2019) since it could allow for the concentration of ingested ILI to reach a threshold level at which the PM concentration dominates the PM - ILI relationship. Such might also be applicable indoors, traditional transmission mechanisms notwithstanding.

The size distribution, morphology, and even the chemical composition of aerosols, or particulate matter, within the inhaled air are important for predicting the respiratory impact. But this impact is under-represented by our current aerosol measurement technology primarily since the technology is not able to concurrently determine size distribution, morphology, and even the chemical composition in near real-time on an individual basis. Improved technological capability could also provide beneficial insights toward answering a standing question, i.e., Do airborne viruses, especially influenza and influenza-like illness viruses, go dormant (i.e., become inactive) during atmospheric transport, whether in cloud or not, and remain in that state until sometime after that virus lands in a ‘nurturing’ environment that transforms its non-dormant state into a metabolically active state? A change in ILI concentration or a change in the environmental conditions can trigger a health impact, other human-related factors notwithstanding. For example, increased ILI (activity) in our body stresses our immune system, thereby making us more susceptible to showing symptoms. This discussion (i) does not account for the time scales involved and (ii) might rely on the ILI microorganism being active or becoming active when it lands inside our respiratory system. We do not have a readily useful technological capability to adequately begin to answer most of these unknowns.

### 2.1 A conceptual system for individual atmospheric aerosol size, concentration, morphology and composition measurements

Atmospheric aerosols generally interact in concurrent, multiple non-linear ways with physical, chemical, and biological processes that often occur simultaneously at variable time and/or spatial scales. Aerosol process-level properties vary on scales of at least meters to thousands of meters. The detection and simulation abilities of such interactions, especially those near and shorter than hundreds of meters scale, are major limiting challenges that have direct implications to understanding and modeling how aerosols might influence human health (e.g., Chen et al., 2020; Wang et al., 2019; Dong et al., 2018; Gao and Ji, 2018; Hart et al., 2018; Miller and Xu, 2018), how aerosols interact among themselves and/or other processes and their surroundings (e.g., Pani et al., 2019; DeFelice, 2018; Dong et al., 2018; Fröhlich-Nowoisky et al., 2016; Khain et al., 2015; Calvo et al., 2013; Li et al., 2013; Sun et al., 2013; Després et al., 2012; Jiménez-Escalona and Peralta, 2012; Tao et al., 2012; Udisti et al., 2012; Andrews et al., 2011; Hultin et al., 2011; Mahowald et al., 2011; Moore et al., 2011; Modini et al., 2010; Andreae, 2009; Fan et al., 2009; Khain and Lynn, 2009; Min et al., 2009; Rosenfeld et al., 2008; Griffin, 2007; Bates et al., 2006; Textor et al., 2006; Reid et al., 2005; Lewis and Schwartz, 2004; Mikhailov et al., 2004; Pinker et al., 2004; Pósfai et al., 2003; Zielinski, 2002; Baron and Willeke, 2001; Baron et al., 2001; Naoki et al., 2001; Fuentes et al., 2000; Leck and Bigg, 1999; DeFelice and Cheng, 1998; DeFelice, 1997a,b; Kaufman et al., 1997; Pruppacher and Klett, 1997; DeFelice, 1996; Saxena, 1996; Chylek and Wong, 1995; Pruppacher and Jaenicke, 1995; DeFelice and Saxena, 1994; Chiswell et al., 1993; Liou, 1992; Götz et al., 1991; Twomey, 1991; Hoppel et al., 1989, 1990; Saxena et al., 1989; Hidy, 1984; Gill et al., 1983; Deepak, 1982; Spinhirne et al., 1979; Hogan and Barnard, 1978; Friedlander, 1977; Schnell and Vali, 1976; Schaefer, 1972; Blanchard and Syzdek, 1970; Hoffer and Mallen, 1970).

There is a need for an operational, field-worthy system to measure aerosol size, concentration, morphology and composition of an individual aerosol among an aerosol population on submicron spatial and corresponding time scales. The current state-of-the-science aerosol physical and chemical attributes measuring systems are labor intensive, necessitate after-the-fact analyses (taking months and longer to receive the information), require expensive and extended maintenance/servicing, for example. Although some may argue the viability of limiting factors, technological advances in computational platforms and other auxiliary technologies, for example, no longer allow for using ‘age-old’ bulk aerosol attribute measurements.

The conceptual next generation system for measuring individual aerosol physical and chemical attributes needs to extend the abilities of contemporary scanning/transmission electron microscopy, while minimizing their limitations with respect to atomic mass, sample preparation and analyses. It would be a collective evolution of the most recent technological advances under development (e.g., Pan, 2015; Lack et al., 2014) with the current ongoing miniaturization of Scanning Electron Microscopes and corresponding streamlining of the sample preparation and analysis processes. The next generation system would at least require the following functional components on an individual aerosol basis; (a) identify location, physical atmospheric conditions, size distribution, morphology, (b) identify trace-level chemical composition, (c) create a high resolution image enabling a clear identification of each aerosol within a sampled population, and (d) contain a means to process, archive and disseminate the data and information from (a), (b), (c).

## 3 Conclusion

Previous studies have found relationships between outdoor particulate matter (PM) data and influenza and influenza-like illness (ILI) activity. These were primarily conducted in regions with moderate to high aerosol burdened or polluted atmospheric environments. Our objective was to assess the possibility of detecting a relationship between outdoor PM and influenza and influenza-like illness (ILI) activity in a typically less than or equal to moderately polluted region in the United States, such as Tucson Arizona. Given the predominant use of particulate matter with diameters 2.5 micron and smaller (PM_2.5_) mass concentration data in previous studies, we chose to use them as a proxy for outdoor PM data. We used the reported Influenza and influenza-like illness (ILI) activity obtained from the Google Flu Trends database as our proxy for ILI activity.

Our analyses show the well-known relation between ILI activity and environmental conditions, namely temperature and relative humidity. We did not find a correlation between PM_2.5_ mass concentration and reported ILI activity contrary to similar studies conducted in high polluted areas. We found a possible correlation between PM_2.5_ mass concentration anomalies and reported ILI activity that warrants further investigation. We emphasize the need for advanced aerosol measuring technology to ensure optimal understanding of the inhalable PM versus ILI activity relationship. We recommend a more comprehensive investigation of the physical and chemical evolution of a boundary-layer aerosol population and ILI activity, and of the ILI microorganism dormancy process as a function of environmental conditions. This would at least improve our understanding of ILI transmission and thereby improve ILI activity/outbreak forecasts and transmission model accuracies.

## Declarations

### Author contribution statement

T. P. DeFelice: Conceived and designed the experiments; Performed the experiments; Analyzed and interpreted the data; Contributed reagents, materials, analysis tools or data; Wrote the paper.

### Funding statement

This research did not receive any specific grant from funding agencies in the public, commercial, or not-for-profit sectors.

### Competing interest statement

The authors declare no conflict of interest.

### Additional information

No additional information is available for this paper.

## Appendix A Supplementary data

DeFelice-PM-ILI-HELIYON-OnlineResource 1 DeFelice-PM-ILI-HELIYON-OnlineResource 1
