# Supplementary material for: Relationship between temporal anomalies in PM2.5 concentrations and reported influenza/influenza-like illness activity
Source: Heliyon. 2020 Aug 15;6(8):e04726. doi: 10.1016/j.heliyon.2020.e04726 (PMC7428445; doi:10.1016/j.heliyon.2020.e04726)
Supplement: DeFelice-PM-ILI-HELIYON-OnlineResource 1 [file mmc1.pdf]

Online Resource 1-NOAA 850 mb Charts

Relationship between temporal anomalies in $PM_{2.5}$ concentrations and reported influenza/influenza-like illness activity~~On the relation between temporal anomalies in particulate matter mass concentrations and reported influenza and influenza-like illness activity~~

T. P. DeFelice*

*** University of Colorado Boulder, USA.**

Contents

ABSTRACT 8

FIGURE F05-A-USED THE 01/30/2005 WEEK-ENDING DATA FOR THE FEB 01 2005 00 Z 850 MB CHART. COURTESY OF NOAA NWS. BLANK RECTANGLES INDICATE NO REPORTS. 9

FIGURE F05-B-USED THE 02/06/2005 WEEK-ENDING DATA FOR THE FEB 06 2005 12 Z 850 MB CHART. COURTESY OF NOAA NWS. BLANK RECTANGLES INDICATE NO REPORTS. 10

FIGURE F05-C-USED THE 02/13/2005 WEEK-ENDING DATA FOR THE FEB 11 2005 12 Z 850 MB CHART. COURTESY OF NOAA NWS. BLANK RECTANGLES INDICATE NO REPORTS. 11

FIGURE F05-D-USED THE 02/13/2005 WEEK-ENDING DATA FOR THE FEB 16 2005 12 Z 850 MB CHART. COURTESY OF NOAA NWS. BLANK RECTANGLES INDICATE NO REPORTS. 12

FIGURE F05-E-USED THE 02/20/2005 WEEK-ENDING DATA FOR THE FEB 20 2005 12 Z 850 MB CHART. COURTESY OF NOAA NWS. BLANK RECTANGLES INDICATE NO REPORTS. 13

FIGURE F05-F-USED THE 02/27/2005 WEEK-ENDING DATA FOR THE FEB 27 2005 12 Z 850 MB CHART. COURTESY OF NOAA NWS. BLANK RECTANGLES INDICATE NO REPORTS. 14

FIGURE F05-G-USED THE 03/13/2005 WEEK-ENDING DATA FOR THE MAR 12 2005 12 Z 850 MB CHART. COURTESY OF NOAA NWS. BLANK RECTANGLES INDICATE NO REPORTS. 15

FIGURE F05-H-USED THE 03/20/2005 WEEK-ENDING DATA FOR THE MAR 20 2005 12 Z 850 MB CHART. COURTESY OF NOAA NWS. BLANK RECTANGLES INDICATE NO REPORTS. 15

FIGURE F05-I1-USED THE 03/27/2005 WEEK-ENDING DATA FOR THE MAR 26 2005 12 Z 850 MB CHART.

COURTESY OF NOAA NWS. BLANK RECTANGLES INDICATE NO REPORTS. 16

FIGURE F05-I2-USED THE 03/27/2005 WEEK-ENDING DATA FOR THE MAR 27 2005 12 Z 850 MB CHART. COURTESY OF NOAA NWS. BLANK RECTANGLES INDICATE NO REPORTS. 17

FIGURE F05-J1-USED THE 04/10/2005 WEEK-ENDING DATA FOR THE APR 09 2005 12 Z 850 MB CHART. COURTESY OF NOAA NWS. BLANK RECTANGLES INDICATE NO REPORTS. 18

FIGURE F05-J2-USED THE 04/10/2005 WEEK-ENDING DATA FOR THE APR 10 2005 12 Z 850 MB CHART. COURTESY OF NOAA NWS. BLANK RECTANGLES INDICATE NO REPORTS. 19

FIGURE F05-K-USED THE 04/24/2005 WEEK-ENDING DATA FOR THE APR 23 2005 12 Z 850 MB CHART. COURTESY OF NOAA NWS. BLANK RECTANGLES INDICATE NO REPORTS. 20

FIGURE 05G-A-USED THE 10/16/2005 WEEK-ENDING PHYSICIAN INFLUENZA-LIKE ILLNESS REPORT DATA, WITH THE OCT 15 2005 12 Z 850 MB CHART. (COURTESY OF NOAA NWS; GOOGLE FLU TRENDS). 21

FIGURE 05G-B-USED THE 10/23/2005 WEEK-ENDING PHYSICIAN INFLUENZA-LIKE ILLNESS REPORT DATA, WITH THE OCT 25 2005 12 Z 850 MB CHART. (COURTESY OF NOAA NWS; GOOGLE FLU TRENDS). 22

FIGURE 05G-C-USED THE 10/30/2005 WEEK-ENDING PHYSICIAN INFLUENZA-LIKE ILLNESS REPORT DATA, WITH THE OCT 30 2005 12 Z 850 MB CHART. (COURTESY OF NOAA NWS; GOOGLE FLU TRENDS). 23

FIGURE 05G-D-USED THE 11/06/2005 WEEK-ENDING PHYSICIAN INFLUENZA-LIKE ILLNESS REPORT DATA, WITH THE NOV 04 2005 12 Z 850 MB CHART. (COURTESY OF NOAA NWS; GOOGLE FLU TRENDS). 24

FIGURE 05G-E-USED THE 11/13/2005 WEEK-ENDING PHYSICIAN INFLUENZA-LIKE ILLNESS REPORT DATA, WITH THE NOV 12 2005 12 Z 850 MB CHART. (COURTESY OF NOAA NWS; GOOGLE FLU TRENDS). 25

FIGURE 05G-F-USED THE 11/20/2005 WEEK-ENDING PHYSICIAN INFLUENZA-LIKE ILLNESS REPORT DATA, WITH THE NOV 18 2005 12 Z 850 MB CHART. (COURTESY OF NOAA NWS; GOOGLE FLU TRENDS). 26

FIGURE 05G-G-USED THE 11/27/2005 WEEK-ENDING PHYSICIAN INFLUENZA-LIKE ILLNESS REPORT DATA, WITH THE NOV 24 2005 12 Z 850 MB CHART. (COURTESY OF NOAA NWS; GOOGLE FLU TRENDS). 27

FIGURE 05G-H-USED THE 11/27/2005 WEEK-ENDING PHYSICIAN INFLUENZA-LIKE ILLNESS REPORT DATA, WITH THE NOV 30 2005 12 Z 850 MB CHART. (COURTESY OF NOAA NWS; GOOGLE FLU TRENDS). 28

FIGURE 05G-I-USED THE 12/04/2005 WEEK-ENDING PHYSICIAN INFLUENZA-LIKE ILLNESS REPORT DATA, WITH THE DEC 07 2005 12 Z 850 MB CHART. (COURTESY OF NOAA NWS; GOOGLE FLU TRENDS). 29

FIGURE 05G-J-USED THE 12/11/2005 WEEK-ENDING PHYSICIAN INFLUENZA-LIKE ILLNESS REPORT DATA, WITH THE DEC 14 2005 12 Z 850 MB CHART. (COURTESY OF NOAA NWS; GOOGLE FLU TRENDS). 30

FIGURE 05G-K-USED THE 12/25/2005 WEEK-ENDING PHYSICIAN INFLUENZA-LIKE ILLNESS REPORT DATA, WITH THE DEC 22 2005 12 Z 850 MB CHART. (COURTESY OF NOAA NWS; GOOGLE FLU TRENDS). 31

FIGURE F06-A-USED THE 01/01/2006 WEEK-ENDING PHYSICIAN INFLUENZA-LIKE ILLNESS REPORT DATA, WITH THE JAN 03 2006 12 Z 850 MB CHART. (COURTESY OF NOAA NWS; GOOGLE FLU TRENDS). 32

FIGURE F06-B-USED THE 01/08/2006 WEEK-ENDING PHYSICIAN INFLUENZA-LIKE ILLNESS REPORT DATA, WITH THE JAN 11 2006 12 Z 850 MB CHART. (COURTESY OF NOAA NWS; GOOGLE FLU TRENDS). 33

FIGURE F06-C-USED THE 01/15/2006 WEEK-ENDING PHYSICIAN INFLUENZA-LIKE ILLNESS REPORT DATA, WITH THE JAN 17 2006 12 Z 850 MB CHART. (COURTESY OF NOAA NWS; GOOGLE FLU TRENDS). 34

FIGURE F06-D-USED THE 01/22/2006 WEEK-ENDING PHYSICIAN INFLUENZA-LIKE ILLNESS REPORT DATA, WITH THE JAN 23 2006 12 Z 850 MB CHART. (COURTESY OF NOAA NWS; GOOGLE FLU TRENDS). 35

FIGURE F06-E-USED THE 01/29/2006 WEEK-ENDING PHYSICIAN INFLUENZA-LIKE ILLNESS REPORT DATA, WITH THE JAN 30 2006 12 Z 850 MB CHART. (COURTESY OF NOAA NWS; GOOGLE FLU TRENDS). 36

FIGURE F06-F-USED THE 02/05/2006 WEEK-ENDING PHYSICIAN INFLUENZA-LIKE ILLNESS REPORT DATA, WITH

THE FEB 06 2006 12 Z 850 MB CHART. (COURTESY OF NOAA NWS; GOOGLE FLU TRENDS). 37

FIGURE F06-G-USED THE 02/12/2006 WEEK-ENDING PHYSICIAN INFLUENZA-LIKE ILLNESS REPORT DATA, WITH THE FEB 12 2006 12 Z 850 MB CHART. (COURTESY OF NOAA NWS; GOOGLE FLU TRENDS). 38

FIGURE F06-H-USED THE 02/19/2006 WEEK-ENDING PHYSICIAN INFLUENZA-LIKE ILLNESS REPORT DATA, WITH THE FEB 21 2006 12 Z 850 MB CHART. (COURTESY OF NOAA NWS; GOOGLE FLU TRENDS). 39

FIGURE F06-I-USED THE 02/26/2006 WEEK-ENDING PHYSICIAN INFLUENZA-LIKE ILLNESS REPORT DATA, WITH THE FEB 27 2006 12 Z 850 MB CHART. (COURTESY OF NOAA NWS; GOOGLE FLU TRENDS). 40

FIGURE F06-J1-USED THE 03/05/2006 WEEK-ENDING PHYSICIAN INFLUENZA-LIKE ILLNESS REPORT DATA, WITH THE MAR 04 2006 12 Z 850 MB CHART. (COURTESY OF NOAA NWS; GOOGLE FLU TRENDS). 41

FIGURE F06-J2-USED THE 03/05/2006 WEEK-ENDING PHYSICIAN INFLUENZA-LIKE ILLNESS REPORT DATA, WITH THE MAR 07 2006 12 Z 850 MB CHART. (COURTESY OF NOAA NWS; GOOGLE FLU TRENDS). 42

FIGURE F06-K1-USED THE 03/12/2006 WEEK-ENDING PHYSICIAN INFLUENZA-LIKE ILLNESS REPORT DATA, WITH THE MAR 09 2006 12 Z 850 MB CHART. (COURTESY OF NOAA NWS; GOOGLE FLU TRENDS). 43

FIGURE F06-K2-USED THE 03/12/2006 WEEK-ENDING PHYSICIAN INFLUENZA-LIKE ILLNESS REPORT DATA, WITH THE MAR 12 2006 12 Z 850 MB CHART. (COURTESY OF NOAA NWS; GOOGLE FLU TRENDS). 44

FIGURE F06-K3-USED THE 03/12/2006 WEEK-ENDING PHYSICIAN INFLUENZA-LIKE ILLNESS REPORT DATA, WITH THE MAR 14 2006 12 Z 850 MB CHART. (COURTESY OF NOAA NWS; GOOGLE FLU TRENDS). 45

FIGURE F06-L-USED THE 03/26/2006 WEEK-ENDING PHYSICIAN INFLUENZA-LIKE ILLNESS REPORT DATA, WITH THE MAR 24 2006 12 Z 850 MB CHART. (COURTESY OF NOAA NWS; GOOGLE FLU TRENDS). 46

FIGURE F06-M-USED THE 04/02/2006 WEEK-ENDING PHYSICIAN INFLUENZA-LIKE ILLNESS REPORT DATA, WITH THE APR 02 2006 12 Z 850 MB CHART. (COURTESY OF NOAA NWS; GOOGLE FLU TRENDS). 47

FIGURE F06-N-USED THE 04/09/2006 WEEK-ENDING PHYSICIAN INFLUENZA-LIKE ILLNESS REPORT DATA, WITH THE APR 11 2006 12 Z 850 MB CHART. (COURTESY OF NOAA NWS; GOOGLE FLU TRENDS). 48

FIGURE F06-O-USED THE 04/16/2006 WEEK-ENDING PHYSICIAN INFLUENZA-LIKE ILLNESS REPORT DATA, WITH THE APR 17 2006 12 Z 850 MB CHART. (COURTESY OF NOAA NWS; GOOGLE FLU TRENDS). 49

FIGURE F06-P-USED THE 04/30/2006 WEEK-ENDING PHYSICIAN INFLUENZA-LIKE ILLNESS REPORT DATA, WITH THE MAY 02 2006 12 Z 850 MB CHART. (COURTESY OF NOAA NWS; GOOGLE FLU TRENDS). 50

FIGURE F06-Q-USED THE 05/14/2006 WEEK-ENDING PHYSICIAN INFLUENZA-LIKE ILLNESS REPORT DATA, WITH THE MAY 16 2006 12 Z 850 MB CHART. (COURTESY OF NOAA NWS; GOOGLE FLU TRENDS). 51

FIGURE 06G-A-USED THE 08/27/2006 WEEK-ENDING PHYSICIAN INFLUENZA-LIKE ILLNESS REPORT DATA, WITH THE AUG 26 2006 12 Z 850 MB CHART. (COURTESY OF NOAA NWS; GOOGLE FLU TRENDS). 52

FIGURE 06G-B-USED THE 08/27/2006 WEEK-ENDING PHYSICIAN INFLUENZA-LIKE ILLNESS REPORT DATA, WITH THE AUG 27 2006 12 Z 850 MB CHART. (COURTESY OF NOAA NWS; GOOGLE FLU TRENDS). 53

FIGURE 06G-C-USED THE 09/03/2006 WEEK-ENDING PHYSICIAN INFLUENZA-LIKE ILLNESS REPORT DATA, WITH THE SEP 02 2006 12 Z 850 MB CHART. (COURTESY OF NOAA NWS; GOOGLE FLU TRENDS). 54

FIGURE 06G-D-USED THE 09/03/2006 WEEK-ENDING PHYSICIAN INFLUENZA-LIKE ILLNESS REPORT DATA, WITH THE SEP 05 2006 12 Z 850 MB CHART. (COURTESY OF NOAA NWS; GOOGLE FLU TRENDS). 55

FIGURE 06G-E-USED THE 09/10/2006 WEEK-ENDING PHYSICIAN INFLUENZA-LIKE ILLNESS REPORT DATA, WITH THE SEP 08 2006 12 Z 850 MB CHART. (COURTESY OF NOAA NWS; GOOGLE FLU TRENDS). 56

FIGURE 06G-F-USED THE 09/17/2006 WEEK-ENDING PHYSICIAN INFLUENZA-LIKE ILLNESS REPORT DATA, WITH THE SEP 14 2006 12 Z 850 MB CHART. (COURTESY OF NOAA NWS; GOOGLE FLU TRENDS). 57

FIGURE 06G-G-USED THE 09/17/2006 WEEK-ENDING PHYSICIAN INFLUENZA-LIKE ILLNESS REPORT DATA, WITH

THE SEP 20 2006 12 Z 850 MB CHART. (COURTESY OF NOAA NWS; GOOGLE FLU TRENDS). 58

FIGURE 06G-H-USED THE 09/24/2006 WEEK-ENDING PHYSICIAN INFLUENZA-LIKE ILLNESS REPORT DATA, WITH THE SEP 23 2006 12 Z 850 MB CHART. (COURTESY OF NOAA NWS; GOOGLE FLU TRENDS). 59

FIGURE 06G-I1-USED THE 10/08/2006 WEEK-ENDING PHYSICIAN INFLUENZA-LIKE ILLNESS REPORT DATA, WITH THE OCT 05 2006 12 Z 850 MB CHART. (COURTESY OF NOAA NWS; GOOGLE FLU TRENDS). 60

FIGURE 06G-I2-USED THE 10/08/2006 WEEK-ENDING PHYSICIAN INFLUENZA-LIKE ILLNESS REPORT DATA, WITH THE OCT 09 2006 12 Z 850 MB CHART. (COURTESY OF NOAA NWS; GOOGLE FLU TRENDS). 61

FIGURE 06G-I3-USED THE 10/08/2006 WEEK-ENDING PHYSICIAN INFLUENZA-LIKE ILLNESS REPORT DATA, WITH THE OCT 10 2006 12 Z 850 MB CHART. (COURTESY OF NOAA NWS; GOOGLE FLU TRENDS). 62

FIGURE 06G-J-USED THE 10/15/2006 WEEK-ENDING PHYSICIAN INFLUENZA-LIKE ILLNESS REPORT DATA, WITH THE OCT 14 2006 12 Z 850 MB CHART. (COURTESY OF NOAA NWS; GOOGLE FLU TRENDS). 63

FIGURE 06G-K-USED THE 10/22/2006 WEEK-ENDING PHYSICIAN INFLUENZA-LIKE ILLNESS REPORT DATA, WITH THE OCT 20 2006 12 Z 850 MB CHART. (COURTESY OF NOAA NWS; GOOGLE FLU TRENDS). 64

FIGURE 06G-L1-USED THE 10/29/2006 WEEK-ENDING PHYSICIAN INFLUENZA-LIKE ILLNESS REPORT DATA, WITH THE OCT 26 2006 12 Z 850 MB CHART. (COURTESY OF NOAA NWS; GOOGLE FLU TRENDS). 65

FIGURE 06G-L2-USED THE 10/29/2006 WEEK-ENDING PHYSICIAN INFLUENZA-LIKE ILLNESS REPORT DATA, WITH THE OCT 30 2006 12 Z 850 MB CHART. (COURTESY OF NOAA NWS; GOOGLE FLU TRENDS). 66

FIGURE 06G-M-USED THE 11/05/2006 WEEK-ENDING PHYSICIAN INFLUENZA-LIKE ILLNESS REPORT DATA, WITH THE NOV 02 2006 12 Z 850 MB CHART. (COURTESY OF NOAA NWS; GOOGLE FLU TRENDS). 67

FIGURE 06G-N-USED THE 11/05/2006 WEEK-ENDING PHYSICIAN INFLUENZA-LIKE ILLNESS REPORT DATA, WITH THE NOV 07 2006 12 Z 850 MB CHART. (COURTESY OF NOAA NWS; GOOGLE FLU TRENDS). 68

FIGURE 06G-O-USED THE 11/12/2006 WEEK-ENDING PHYSICIAN INFLUENZA-LIKE ILLNESS REPORT DATA, WITH THE NOV 12 2006 12 Z 850 MB CHART. (COURTESY OF NOAA NWS; GOOGLE FLU TRENDS). 69

FIGURE 06G-P-USED THE 11/19/2006 WEEK-ENDING PHYSICIAN INFLUENZA-LIKE ILLNESS REPORT DATA, WITH THE NOV 19 2006 12 Z 850 MB CHART. (COURTESY OF NOAA NWS; GOOGLE FLU TRENDS). 70

FIGURE 06G-Q1-USED THE 11/26/2006 WEEK-ENDING PHYSICIAN INFLUENZA-LIKE ILLNESS REPORT DATA, WITH THE NOV 25 2006 12 Z 850 MB CHART. (COURTESY OF NOAA NWS; GOOGLE FLU TRENDS). 71

FIGURE 06G-Q2-USED THE 11/26/2006 WEEK-ENDING PHYSICIAN INFLUENZA-LIKE ILLNESS REPORT DATA, WITH THE NOV 28 2006 12 Z 850 MB CHART. (COURTESY OF NOAA NWS; GOOGLE FLU TRENDS). 72

FIGURE 06G-R1-USED THE 12/10/2006 WEEK-ENDING PHYSICIAN INFLUENZA-LIKE ILLNESS REPORT DATA, WITH THE DEC 07 2006 12 Z 850 MB CHART. (COURTESY OF NOAA NWS; GOOGLE FLU TRENDS). 73

FIGURE 06G-R2-USED THE 12/10/2006 WEEK-ENDING PHYSICIAN INFLUENZA-LIKE ILLNESS REPORT DATA, WITH THE DEC 09 2006 12 Z 850 MB CHART. (COURTESY OF NOAA NWS; GOOGLE FLU TRENDS). 74

FIGURE 06G-S1-USED THE 12/17/2006 WEEK-ENDING PHYSICIAN INFLUENZA-LIKE ILLNESS REPORT DATA, WITH THE DEC 14 2006 12 Z 850 MB CHART. (COURTESY OF NOAA NWS; GOOGLE FLU TRENDS). 75

FIGURE 06G-S2-USED THE 12/17/2006 WEEK-ENDING PHYSICIAN INFLUENZA-LIKE ILLNESS REPORT DATA, WITH THE DEC 19 2006 12 Z 850 MB CHART. (COURTESY OF NOAA NWS; GOOGLE FLU TRENDS). 76

FIGURE 06G-T-USED THE 12/24/2006 WEEK-ENDING PHYSICIAN INFLUENZA-LIKE ILLNESS REPORT DATA, WITH THE DEC 24 2006 12 Z 850 MB CHART. (COURTESY OF NOAA NWS; GOOGLE FLU TRENDS). 77

FIGURE 06G-U1-USED THE 12/31/2006 WEEK-ENDING PHYSICIAN INFLUENZA-LIKE ILLNESS REPORT DATA, WITH THE DEC 28 2006 12 Z 850 MB CHART. (COURTESY OF NOAA NWS; GOOGLE FLU TRENDS). 78

FIGURE 06G-U2-USED THE 12/31/2006 WEEK-ENDING PHYSICIAN INFLUENZA-LIKE ILLNESS REPORT DATA, WITH

THE DEC 29 2006 12 Z 850 MB CHART. (COURTESY OF NOAA NWS; GOOGLE FLU TRENDS). 79

FIGURE 06G-U3-USED THE 12/31/2006 WEEK-ENDING PHYSICIAN INFLUENZA-LIKE ILLNESS REPORT DATA, WITH THE DEC 31 2006 12 Z 850 MB CHART. (COURTESY OF NOAA NWS; GOOGLE FLU TRENDS). 80

FIGURE F09-A1-USED THE 01/04/2009 WEEK-ENDING PHYSICIAN INFLUENZA-LIKE ILLNESS REPORT DATA, WITH THE JAN 01 2009 12 Z 850 MB CHART. (COURTESY OF NOAA NWS; GOOGLE FLU TRENDS). 91

FIGURE F09-A2-USED THE 01/04/2009 WEEK-ENDING PHYSICIAN INFLUENZA-LIKE ILLNESS REPORT DATA, WITH THE JAN 07 2009 12 Z 850 MB CHART. (COURTESY OF NOAA NWS; GOOGLE FLU TRENDS). 92

FIGURE F09-B1-USED THE 01/11/2009 WEEK-ENDING PHYSICIAN INFLUENZA-LIKE ILLNESS REPORT DATA, WITH THE JAN 10 2009 12 Z 850 MB CHART. (COURTESY OF NOAA NWS; GOOGLE FLU TRENDS). 93

FIGURE F09-B2-USED THE 01/11/2009 WEEK-ENDING PHYSICIAN INFLUENZA-LIKE ILLNESS REPORT DATA, WITH THE JAN 13 2009 12 Z 850 MB CHART. (COURTESY OF NOAA NWS; GOOGLE FLU TRENDS). 94

FIGURE F09-C1-USED THE 01/18/2009 WEEK-ENDING PHYSICIAN INFLUENZA-LIKE ILLNESS REPORT DATA, WITH THE JAN 16 2009 12 Z 850 MB CHART. (COURTESY OF NOAA NWS; GOOGLE FLU TRENDS). 95

FIGURE F09-C2-USED THE 01/18/2009 WEEK-ENDING PHYSICIAN INFLUENZA-LIKE ILLNESS REPORT DATA, WITH THE JAN 18 2009 12 Z 850 MB CHART. (COURTESY OF NOAA NWS; GOOGLE FLU TRENDS). 96

FIGURE F09-C3-USED THE 01/18/2009 WEEK-ENDING PHYSICIAN INFLUENZA-LIKE ILLNESS REPORT DATA, WITH THE JAN 20 2009 12 Z 850 MB CHART. (COURTESY OF NOAA NWS; GOOGLE FLU TRENDS). 97

FIGURE F09-D-USED THE 01/25/2009 WEEK-ENDING PHYSICIAN INFLUENZA-LIKE ILLNESS REPORT DATA, WITH THE JAN 26 2009 12 Z 850 MB CHART. (COURTESY OF NOAA NWS; GOOGLE FLU TRENDS). 98

FIGURE F09-E1-USED THE 02/01/2009 WEEK-ENDING PHYSICIAN INFLUENZA-LIKE ILLNESS REPORT DATA, WITH THE JAN 31 2009 12 Z 850 MB CHART. (COURTESY OF NOAA NWS; GOOGLE FLU TRENDS). 99

FIGURE F09- E2-USED THE 02/01/2009 WEEK-ENDING PHYSICIAN INFLUENZA-LIKE ILLNESS REPORT DATA, WITH THE FEB 03 2009 12 Z 850 MB CHART. (COURTESY OF NOAA NWS; GOOGLE FLU TRENDS). 100

FIGURE F09-F1-USED THE 02/08/2009 WEEK-ENDING PHYSICIAN INFLUENZA-LIKE ILLNESS REPORT DATA, WITH THE FEB 06 2009 12 Z 850 MB CHART. (COURTESY OF NOAA NWS; GOOGLE FLU TRENDS). 101

FIGURE F09-F2-USED THE 02/08/2009 WEEK-ENDING PHYSICIAN INFLUENZA-LIKE ILLNESS REPORT DATA, WITH THE FEB 09 2009 12 Z 850 MB CHART. (COURTESY OF NOAA NWS; GOOGLE FLU TRENDS). 102

FIGURE F09-G-USED THE 02/15/2009 WEEK-ENDING PHYSICIAN INFLUENZA-LIKE ILLNESS REPORT DATA, WITH THE FEB 15 2009 12 Z 850 MB CHART. (COURTESY OF NOAA NWS; GOOGLE FLU TRENDS). 103

FIGURE F09-H1-USED THE 02/22/2009 WEEK-ENDING PHYSICIAN INFLUENZA-LIKE ILLNESS REPORT DATA, WITH THE FEB 20 2009 12 Z 850 MB CHART. (COURTESY OF NOAA NWS; GOOGLE FLU TRENDS). 104

FIGURE F09-H2-USED THE 02/22/2009 WEEK-ENDING PHYSICIAN INFLUENZA-LIKE ILLNESS REPORT DATA, WITH THE FEB 23 2009 12 Z 850 MB CHART. (COURTESY OF NOAA NWS; GOOGLE FLU TRENDS). 105

FIGURE F09-H3-USED THE 02/22/2009 WEEK-ENDING PHYSICIAN INFLUENZA-LIKE ILLNESS REPORT DATA, WITH THE FEB 25 2009 12 Z 850 MB CHART. (COURTESY OF NOAA NWS; GOOGLE FLU TRENDS). 106

FIGURE F09-I1-USED THE 03/01/2009 WEEK-ENDING PHYSICIAN INFLUENZA-LIKE ILLNESS REPORT DATA, WITH THE FEB 27 2009 12 Z 850 MB CHART. (COURTESY OF NOAA NWS; GOOGLE FLU TRENDS). 107

FIGURE F09-I2-USED THE 03/01/2009 WEEK-ENDING PHYSICIAN INFLUENZA-LIKE ILLNESS REPORT DATA, WITH THE MAR 01 2009 12 Z 850 MB CHART. (COURTESY OF NOAA NWS; GOOGLE FLU TRENDS). 108

FIGURE F09-J1-USED THE 03/08/2009 WEEK-ENDING PHYSICIAN INFLUENZA-LIKE ILLNESS REPORT DATA, WITH THE MAR 08 2009 12 Z 850 MB CHART. (COURTESY OF NOAA NWS; GOOGLE FLU TRENDS). 109

FIGURE F09-J2-USED THE 03/08/2009 WEEK-ENDING PHYSICIAN INFLUENZA-LIKE ILLNESS REPORT DATA, WITH

THE MAR 10 2009 12 Z 850 MB CHART. (COURTESY OF NOAA NWS; GOOGLE FLU TRENDS). 110

FIGURE F09-K1-USED THE 03/29/2009 WEEK-ENDING PHYSICIAN INFLUENZA-LIKE ILLNESS REPORT DATA, WITH THE MAR 31 2009 12 Z 850 MB CHART. (COURTESY OF NOAA NWS; GOOGLE FLU TRENDS). 111

FIGURE F09-K2-USED THE 03/29/2009 WEEK-ENDING PHYSICIAN INFLUENZA-LIKE ILLNESS REPORT DATA, WITH THE APR 01 2009 12 Z 850 MB CHART. (COURTESY OF NOAA NWS; GOOGLE FLU TRENDS). 112

FIGURE F09-L-USED THE 04/05/2009 WEEK-ENDING PHYSICIAN INFLUENZA-LIKE ILLNESS REPORT DATA, WITH THE APR 05 2009 12 Z 850 MB CHART. (COURTESY OF NOAA NWS; GOOGLE FLU TRENDS). 113

FIGURE F09-M-USED THE 04/12/2009 WEEK-ENDING PHYSICIAN INFLUENZA-LIKE ILLNESS REPORT DATA, WITH THE APR 10 2009 12 Z 850 MB CHART. (COURTESY OF NOAA NWS; GOOGLE FLU TRENDS). 114

FIGURE F09-N-USED THE 04/19/2009 WEEK-ENDING PHYSICIAN INFLUENZA-LIKE ILLNESS REPORT DATA, WITH THE APR 19 2009 12 Z 850 MB CHART. (COURTESY OF NOAA NWS; GOOGLE FLU TRENDS). 115

FIGURE F09-O1-USED THE 04/26/2009 WEEK-ENDING PHYSICIAN INFLUENZA-LIKE ILLNESS REPORT DATA, WITH THE APR 24 2009 12 Z 850 MB CHART. (COURTESY OF NOAA NWS; GOOGLE FLU TRENDS). 116

FIGURE F09-O2-USED THE 04/26/2009 WEEK-ENDING PHYSICIAN INFLUENZA-LIKE ILLNESS REPORT DATA, WITH THE APR 27 2009 12 Z 850 MB CHART. (COURTESY OF NOAA NWS; GOOGLE FLU TRENDS). 117

FIGURE F09-P1-USED THE 05/03/2009 WEEK-ENDING PHYSICIAN INFLUENZA-LIKE ILLNESS REPORT DATA, WITH THE APR 30 2009 12 Z 850 MB CHART. (COURTESY OF NOAA NWS; GOOGLE FLU TRENDS). 118

FIGURE F09-P2-USED THE 05/03/2009 WEEK-ENDING PHYSICIAN INFLUENZA-LIKE ILLNESS REPORT DATA, WITH THE MAY 05 2009 12 Z 850 MB CHART. (COURTESY OF NOAA NWS; GOOGLE FLU TRENDS). 119

FIGURE F09-Q1-USED THE 05/10/2009 WEEK-ENDING PHYSICIAN INFLUENZA-LIKE ILLNESS REPORT DATA, WITH THE MAY 09 2009 12 Z 850 MB CHART. (COURTESY OF NOAA NWS; GOOGLE FLU TRENDS). 120

FIGURE F09-Q2-USED THE 05/10/2009 WEEK-ENDING PHYSICIAN INFLUENZA-LIKE ILLNESS REPORT DATA, WITH THE MAY 10 2009 12 Z 850 MB CHART. (COURTESY OF NOAA NWS; GOOGLE FLU TRENDS). 121

FIGURE F09-Q3-USED THE 05/10/2009 WEEK-ENDING PHYSICIAN INFLUENZA-LIKE ILLNESS REPORT DATA, WITH THE MAY 13 2009 12 Z 850 MB CHART. (COURTESY OF NOAA NWS; GOOGLE FLU TRENDS). 122

FIGURE 09G-A-USED THE 09/27/2009 WEEK-ENDING PHYSICIAN INFLUENZA-LIKE ILLNESS REPORT DATA, WITH THE SEP 28 2009 12 Z 850 MB CHART. (COURTESY OF NOAA NWS; GOOGLE FLU TRENDS). 123

FIGURE 09G-B-USED THE 10/04/2009 WEEK-ENDING PHYSICIAN INFLUENZA-LIKE ILLNESS REPORT DATA, WITH THE OCT 05 2009 12 Z 850 MB CHART. (COURTESY OF NOAA NWS; GOOGLE FLU TRENDS). 124

FIGURE 09G-C1-USED THE 10/11/2009 WEEK-ENDING PHYSICIAN INFLUENZA-LIKE ILLNESS REPORT DATA, WITH THE OCT 08 2009 12 Z 850 MB CHART. (COURTESY OF NOAA NWS; GOOGLE FLU TRENDS). 125

FIGURE 09G-C2-USED THE 10/11/2009 WEEK-ENDING PHYSICIAN INFLUENZA-LIKE ILLNESS REPORT DATA, WITH THE OCT 14 2009 12 Z 850 MB CHART. (COURTESY OF NOAA NWS; GOOGLE FLU TRENDS). 126

FIGURE 09G-D-USED THE 10/18/2009 WEEK-ENDING PHYSICIAN INFLUENZA-LIKE ILLNESS REPORT DATA, WITH THE OCT 20 2009 12 Z 850 MB CHART. (COURTESY OF NOAA NWS; GOOGLE FLU TRENDS). 127

FIGURE 09G-E-USED THE 10/25/2009 WEEK-ENDING PHYSICIAN INFLUENZA-LIKE ILLNESS REPORT DATA, WITH THE OCT 25 2009 12 Z 850 MB CHART. (COURTESY OF NOAA NWS; GOOGLE FLU TRENDS). 128

FIGURE 09G-F-USED THE 11/01/2005 WEEK-ENDING PHYSICIAN INFLUENZA-LIKE ILLNESS REPORT DATA, WITH THE NOV 04 2009 12 Z 850 MB CHART. (COURTESY OF NOAA NWS; GOOGLE FLU TRENDS). 129

FIGURE 09G-G-USED THE 11/15/2009 WEEK-ENDING PHYSICIAN INFLUENZA-LIKE ILLNESS REPORT DATA, WITH THE NOV 13 2009 12 Z 850 MB CHART. (COURTESY OF NOAA NWS; GOOGLE FLU TRENDS). 130

FIGURE 09G-H1-USED THE 11/22/2009 WEEK-ENDING PHYSICIAN INFLUENZA-LIKE ILLNESS REPORT DATA, WITH

THE NOV 20 2009 12 Z 850 MB CHART. (COURTESY OF NOAA NWS; GOOGLE FLU TRENDS). 131

FIGURE 09G-H2-USED THE 11/22/2009 WEEK-ENDING PHYSICIAN INFLUENZA-LIKE ILLNESS REPORT DATA, WITH THE NOV 24 2009 12 Z 850 MB CHART. (COURTESY OF NOAA NWS; GOOGLE FLU TRENDS). 132

FIGURE 09G-I1-USED THE 11/29/2009 WEEK-ENDING PHYSICIAN INFLUENZA-LIKE ILLNESS REPORT DATA, WITH THE NOV 27 2009 12 Z 850 MB CHART. (COURTESY OF NOAA NWS; GOOGLE FLU TRENDS). 133

FIGURE 09G-I2-USED THE 11/29/2009 WEEK-ENDING PHYSICIAN INFLUENZA-LIKE ILLNESS REPORT DATA, WITH THE NOV 30 2009 12 Z 850 MB CHART. (COURTESY OF NOAA NWS; GOOGLE FLU TRENDS). 134

FIGURE 09G-J-USED THE 12/06/2009 WEEK-ENDING PHYSICIAN INFLUENZA-LIKE ILLNESS REPORT DATA, WITH THE DEC 06 2009 12 Z 850 MB CHART. (COURTESY OF NOAA NWS; GOOGLE FLU TRENDS). 135

FIGURE 09G-K-USED THE 12/13/2009 WEEK-ENDING PHYSICIAN INFLUENZA-LIKE ILLNESS REPORT DATA, WITH THE DEC 12 2009 12 Z 850 MB CHART. (COURTESY OF NOAA NWS; GOOGLE FLU TRENDS). 136

FIGURE 09G-L-USED THE 12/13/2009 WEEK-ENDING PHYSICIAN INFLUENZA-LIKE ILLNESS REPORT DATA, WITH THE DEC 16 2009 12 Z 850 MB CHART. (COURTESY OF NOAA NWS; GOOGLE FLU TRENDS). 137

FIGURE 09G-M-USED THE 12/20/2009 WEEK-ENDING PHYSICIAN INFLUENZA-LIKE ILLNESS REPORT DATA, WITH THE DEC 22 2009 12 Z 850 MB CHART. (COURTESY OF NOAA NWS; GOOGLE FLU TRENDS). 138

FIGURE 09G-N1-USED THE 12/27/2009 WEEK-ENDING PHYSICIAN INFLUENZA-LIKE ILLNESS REPORT DATA, WITH THE DEC 26 2009 12 Z 850 MB CHART. (COURTESY OF NOAA NWS; GOOGLE FLU TRENDS). 139

FIGURE 09G-N2-USED THE 12/27/2009 WEEK-ENDING PHYSICIAN INFLUENZA-LIKE ILLNESS REPORT DATA, WITH THE DEC 30 2009 12 Z 850 MB CHART. (COURTESY OF NOAA NWS; GOOGLE FLU TRENDS). 140

FIGURE 09G-O-USED THE 01/03/2010 WEEK-ENDING PHYSICIAN INFLUENZA-LIKE ILLNESS REPORT DATA, WITH THE DEC 31 2009 12 Z 850 MB CHART. (COURTESY OF NOAA NWS; GOOGLE FLU TRENDS). 141

FIGURE 09H-A-USED THE 05/10/2009 WEEK-ENDING PHYSICIAN INFLUENZA-LIKE ILLNESS REPORT DATA, WITH THE MAY 13 2009 12 Z 850 MB CHART. (COURTESY OF NOAA NWS; GOOGLE FLU TRENDS). 142

FIGURE 09H-B-USED THE 05/24/2009 WEEK-ENDING PHYSICIAN INFLUENZA-LIKE ILLNESS REPORT DATA, WITH THE MAY 24 2009 12 Z 850 MB CHART. (COURTESY OF NOAA NWS; GOOGLE FLU TRENDS). 143

FIGURE 09H-B1-USED THE 06/07/2009 WEEK-ENDING PHYSICIAN INFLUENZA-LIKE ILLNESS REPORT DATA, WITH THE JUN 04 2009 12 Z 850 MB CHART. (COURTESY OF NOAA NWS; GOOGLE FLU TRENDS). 144

FIGURE 09H-B2-USED THE 06/07/2009 WEEK-ENDING PHYSICIAN INFLUENZA-LIKE ILLNESS REPORT DATA, WITH THE JUN 09 2009 12 Z 850 MB CHART. (COURTESY OF NOAA NWS; GOOGLE FLU TRENDS). 145

FIGURE 09H-C-USED THE 06/14/2009 WEEK-ENDING PHYSICIAN INFLUENZA-LIKE ILLNESS REPORT DATA, WITH THE JUN 15 2009 12 Z 850 MB CHART. (COURTESY OF NOAA NWS; GOOGLE FLU TRENDS). 146

FIGURE 09H-D-USED THE 06/21/2009 WEEK-ENDING PHYSICIAN INFLUENZA-LIKE ILLNESS REPORT DATA, WITH THE JUN 18 2009 12 Z 850 MB CHART. (COURTESY OF NOAA NWS; GOOGLE FLU TRENDS). 147

FIGURE 09H-E-USED THE 06/28/2009 WEEK-ENDING PHYSICIAN INFLUENZA-LIKE ILLNESS REPORT DATA, WITH THE JUN 27 2009 12 Z 850 MB CHART. (COURTESY OF NOAA NWS; GOOGLE FLU TRENDS). 148

FIGURE 09H-F-USED THE 07/05/2009 WEEK-ENDING PHYSICIAN INFLUENZA-LIKE ILLNESS REPORT DATA, WITH THE JUL 08 2009 12 Z 850 MB CHART. (COURTESY OF NOAA NWS; GOOGLE FLU TRENDS). 149

FIGURE 09H-G-USED THE 07/19/2009 WEEK-ENDING PHYSICIAN INFLUENZA-LIKE ILLNESS REPORT DATA, WITH THE JUL 18 2009 12 Z 850 MB CHART. (COURTESY OF NOAA NWS; GOOGLE FLU TRENDS). 150

FIGURE 09H-H-USED THE 07/26/2009 WEEK-ENDING PHYSICIAN INFLUENZA-LIKE ILLNESS REPORT DATA, WITH THE JUL 28 2009 12 Z 850 MB CHART. (COURTESY OF NOAA NWS; GOOGLE FLU TRENDS). 151

FIGURE 09H-I-USED THE 08/02/2009 WEEK-ENDING PHYSICIAN INFLUENZA-LIKE ILLNESS REPORT DATA, WITH

THE AUG 05 2009 12 Z 850 MB CHART. (COURTESY OF NOAA NWS; GOOGLE FLU TRENDS). 152

FIGURE 09H-J-USED THE 08/09/2009 WEEK-ENDING PHYSICIAN INFLUENZA-LIKE ILLNESS REPORT DATA, WITH THE AUG 11 2009 12 Z 850 MB CHART. (COURTESY OF NOAA NWS; GOOGLE FLU TRENDS). 153

FIGURE 09H-K-USED THE 08/16/2009 WEEK-ENDING PHYSICIAN INFLUENZA-LIKE ILLNESS REPORT DATA, WITH THE AUG 17 2009 12 Z 850 MB CHART. (COURTESY OF NOAA NWS; GOOGLE FLU TRENDS). 154

FIGURE 09H-L-USED THE 08/23/2009 WEEK-ENDING PHYSICIAN INFLUENZA-LIKE ILLNESS REPORT DATA, WITH THE AUG 20 2009 12 Z 850 MB CHART. (COURTESY OF NOAA NWS; GOOGLE FLU TRENDS). 155

FIGURE 09H-M1-USED THE 08/30/2009 WEEK-ENDING PHYSICIAN INFLUENZA-LIKE ILLNESS REPORT DATA, WITH THE AUG 30 2009 12 Z 850 MB CHART. (COURTESY OF NOAA NWS; GOOGLE FLU TRENDS). 156

FIGURE 09H-M2-USED THE 08/30/2009 WEEK-ENDING PHYSICIAN INFLUENZA-LIKE ILLNESS REPORT DATA, WITH THE SEP 01 2009 12 Z 850 MB CHART. (COURTESY OF NOAA NWS; GOOGLE FLU TRENDS). 157

FIGURE 09H-N-USED THE 09/13/2009 WEEK-ENDING PHYSICIAN INFLUENZA-LIKE ILLNESS REPORT DATA, WITH THE SEP 10 2009 12 Z 850 MB CHART. (COURTESY OF NOAA NWS; GOOGLE FLU TRENDS). 158

FIGURE 09H-O-USED THE 09/20/2009 WEEK-ENDING PHYSICIAN INFLUENZA-LIKE ILLNESS REPORT DATA, WITH THE SEP 22 2009 12 Z 850 MB CHART. (COURTESY OF NOAA NWS; GOOGLE FLU TRENDS). 159

FIGURE 09H-P-USED THE 09/27/2009 WEEK-ENDING PHYSICIAN INFLUENZA-LIKE ILLNESS REPORT DATA, WITH THE SEP 28 2009 12 Z 850 MB CHART. (COURTESY OF NOAA NWS; GOOGLE FLU TRENDS). 160

ABSTRACT

The following charts are the NOAA 850 mb charts with the tabulated physician reports for Influenza and Influenza-like Illness (ILI) per 100,000 visits overlaid. The ILI reports are for San Diego, CA; Las Vegas, NV; Phoenix, AZ; Tucson, AZ; Albuquerque, NM; Denver, CO; Dallas(Fort Worth), TX; Wichita, KS; Oklahoma City, OK; Tulsa, OK; and St. Louis, MO; and Baja California, Mexico; Sonora, Mexico; Chihuahua, Mexico; and Nuevo León, Mexico. The ILI reports are from Google Flu Trends {Google Flu Trends, http://www.google.org/flutrends}. The entries for Chiuahua, Mexico for the 2005—06 season are blank because no data were available. The 850 mb flow over North America and the physician visit reported Influenza-like visit data for 16 metropolitan areas during October-March 2005-2006, October - March 2006-2007, and the calendar year 2009.

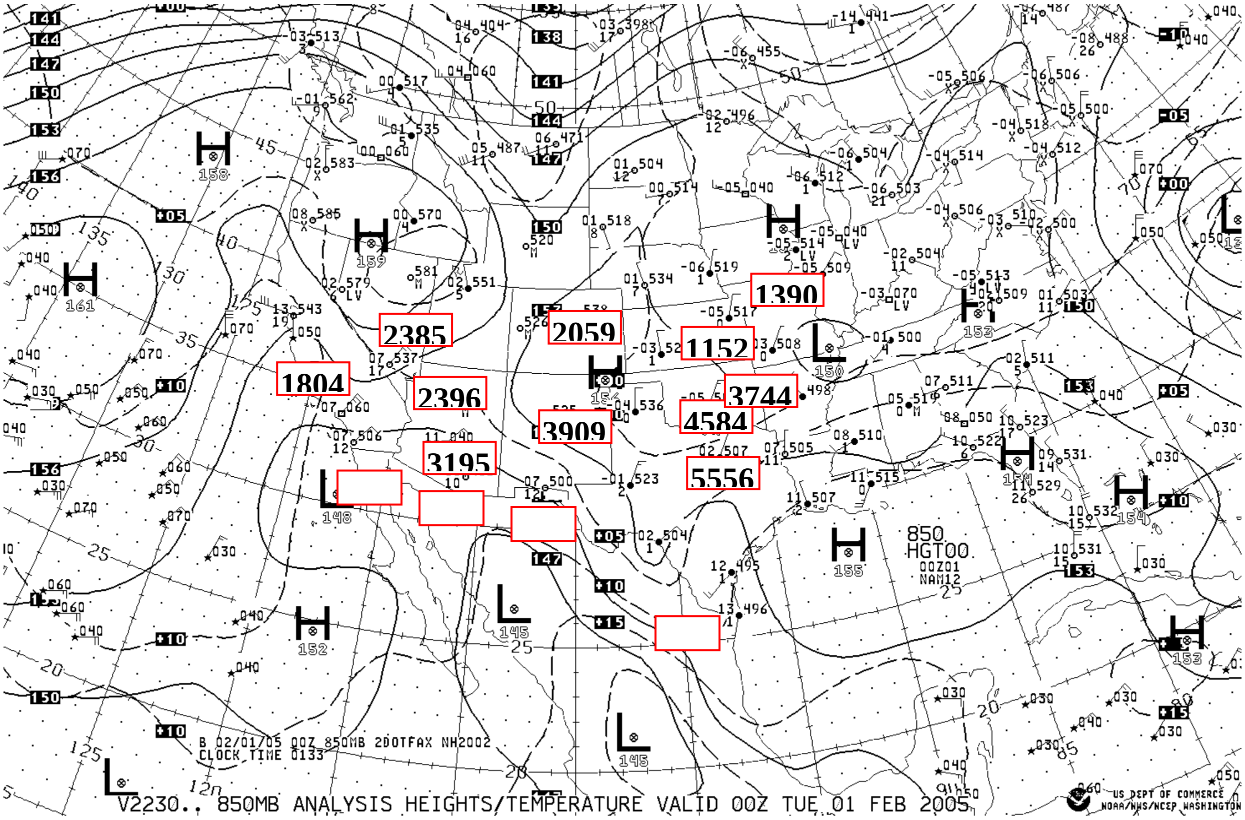

Figure F05-a-Used the 01/30/2005 week-ending data for the Feb 01 2005 00 z 850 mb chart. Courtesy of NOAA NWS. Blank Rectangles indicate no reports.

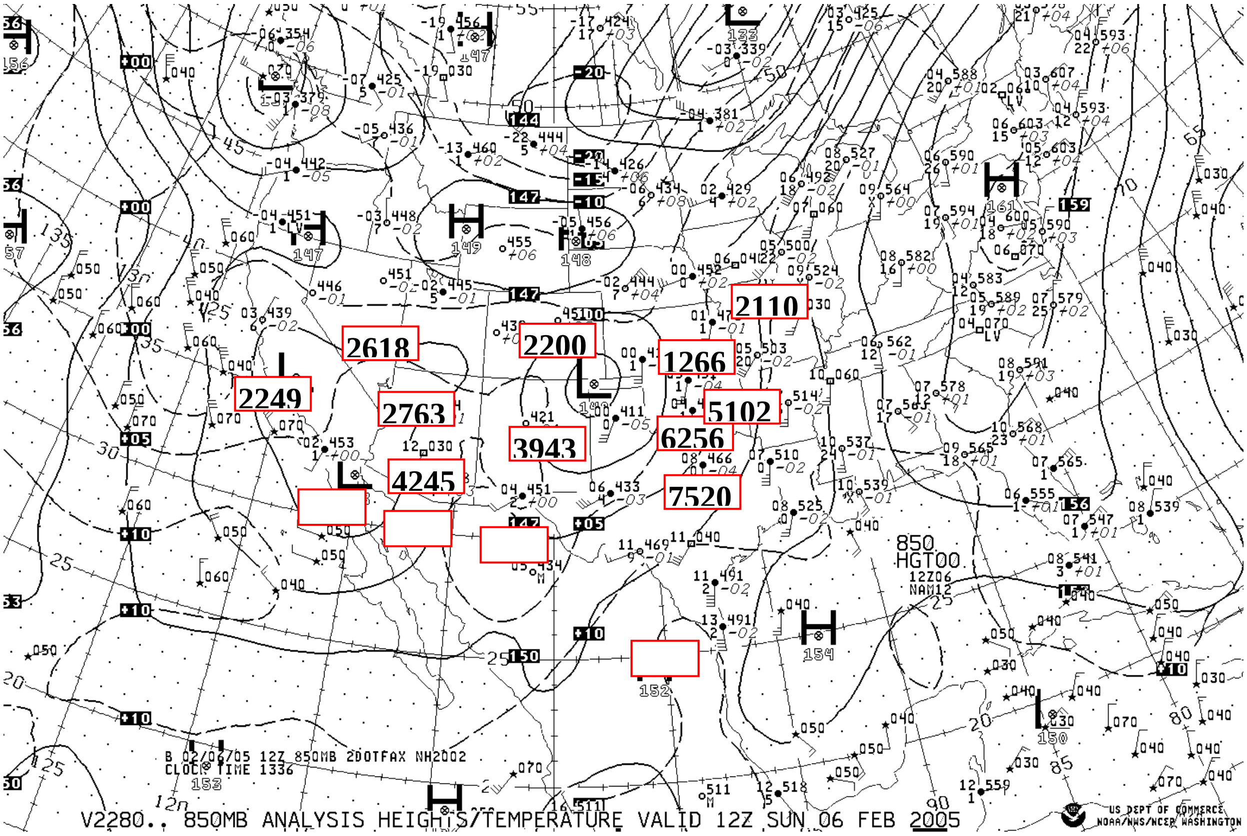

Figure F05-b-Used the 02/06/2005 week-ending data for the Feb 06 2005 12 z 850 mb chart. Courtesy of NOAA NWS. Blank Rectangles indicate no reports.

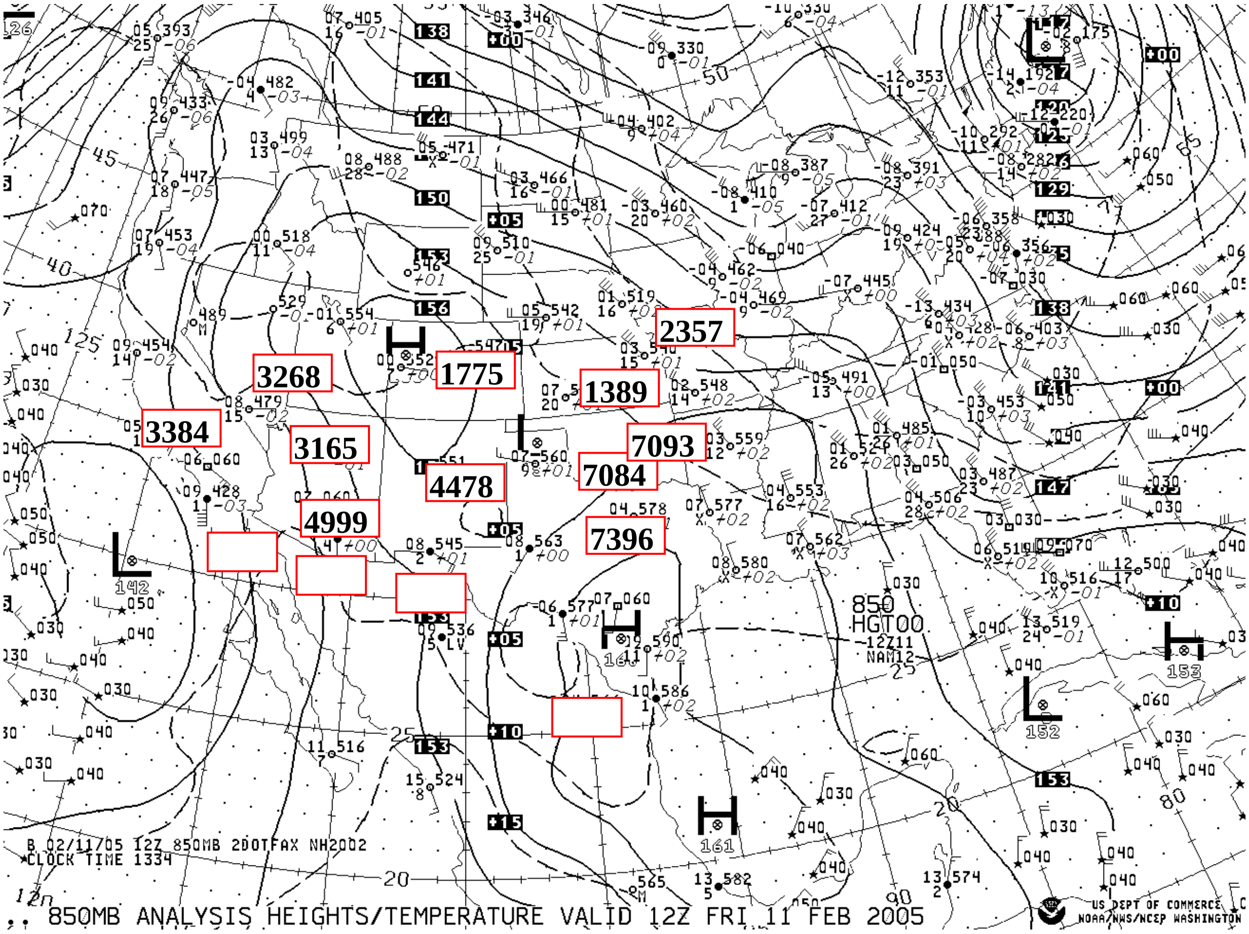

Figure F05-c-Used the 02/13/2005 week-ending data for the Feb 11 2005 12 z 850 mb chart. Courtesy of NOAA NWS. Blank Rectangles indicate no reports.

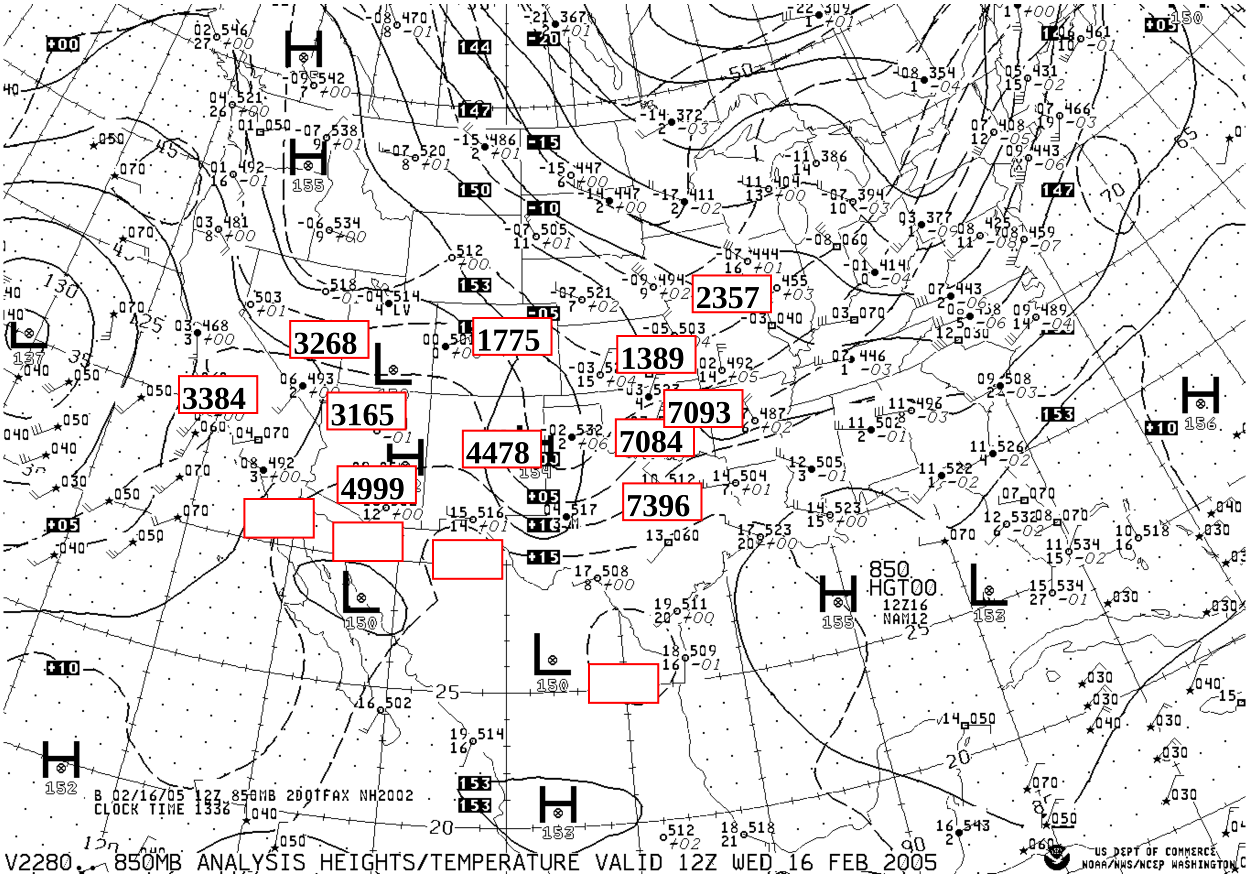

Figure F05-d-Used the 02/13/2005 week-ending data for the Feb 16 2005 12 z 850 mb chart. Courtesy of NOAA NWS. Blank Rectangles indicate no reports.

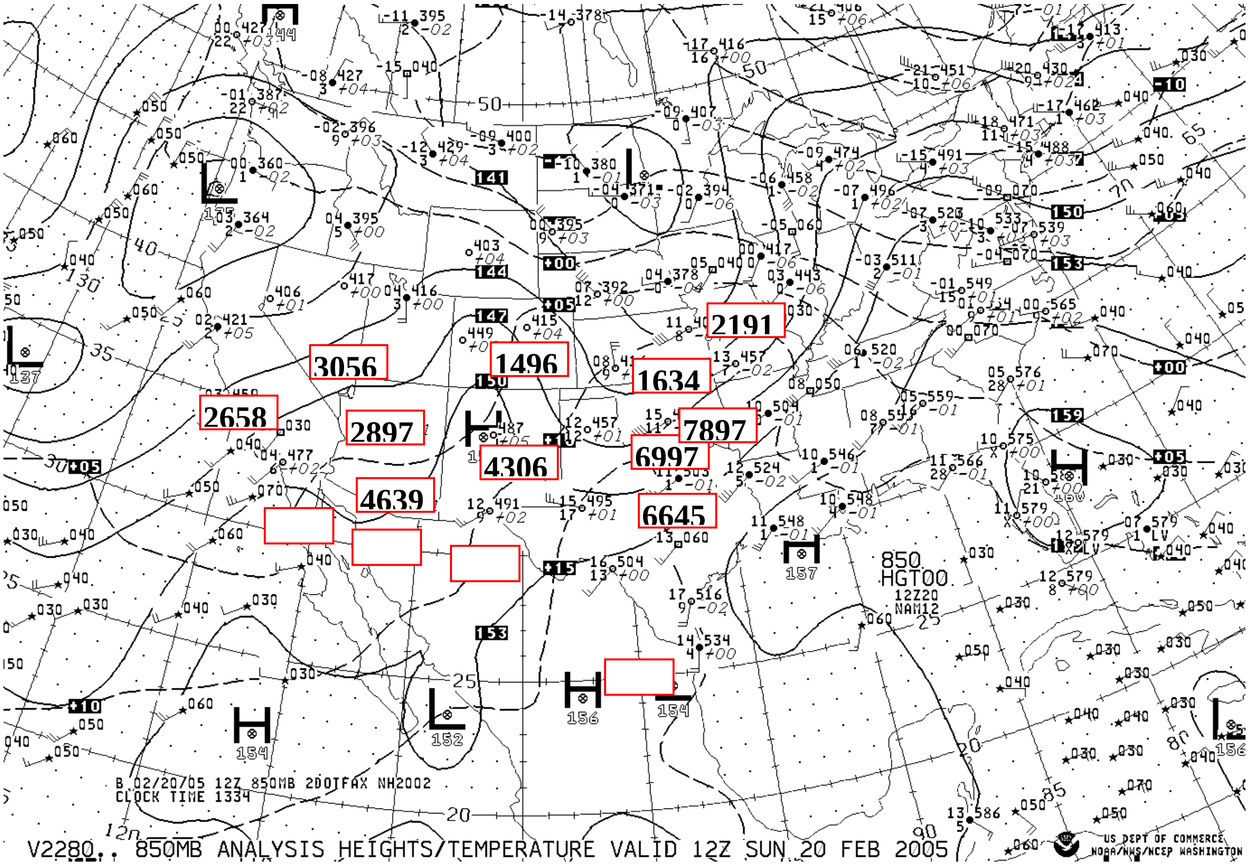

Figure F05-e-Used the 02/20/2005 week-ending data for the Feb 20 2005 12 z 850 mb chart. Courtesy of NOAA NWS. Blank Rectangles indicate no reports.

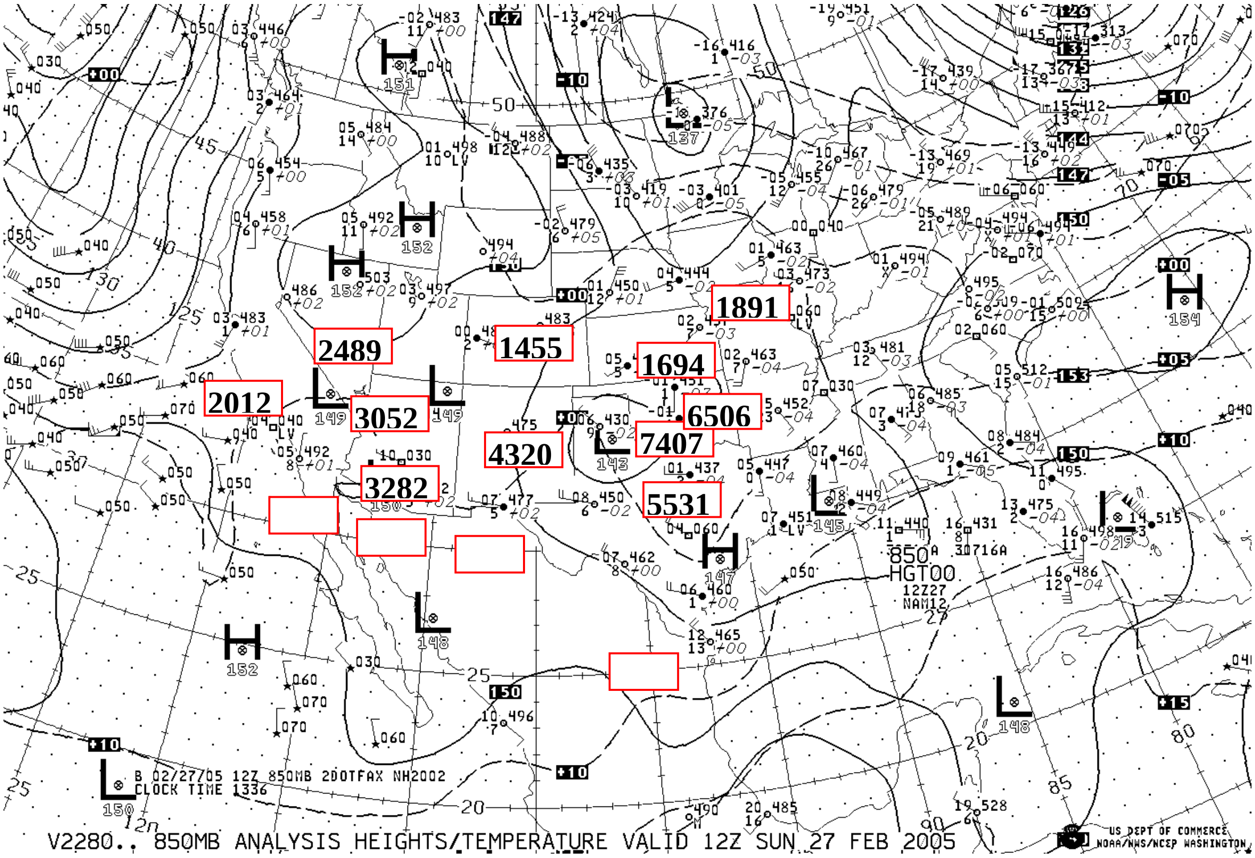

Figure F05-f-Used the 02/27/2005 week-ending data for the Feb 27 2005 12 z 850 mb chart. Courtesy of NOAA NWS. Blank Rectangles indicate no reports.

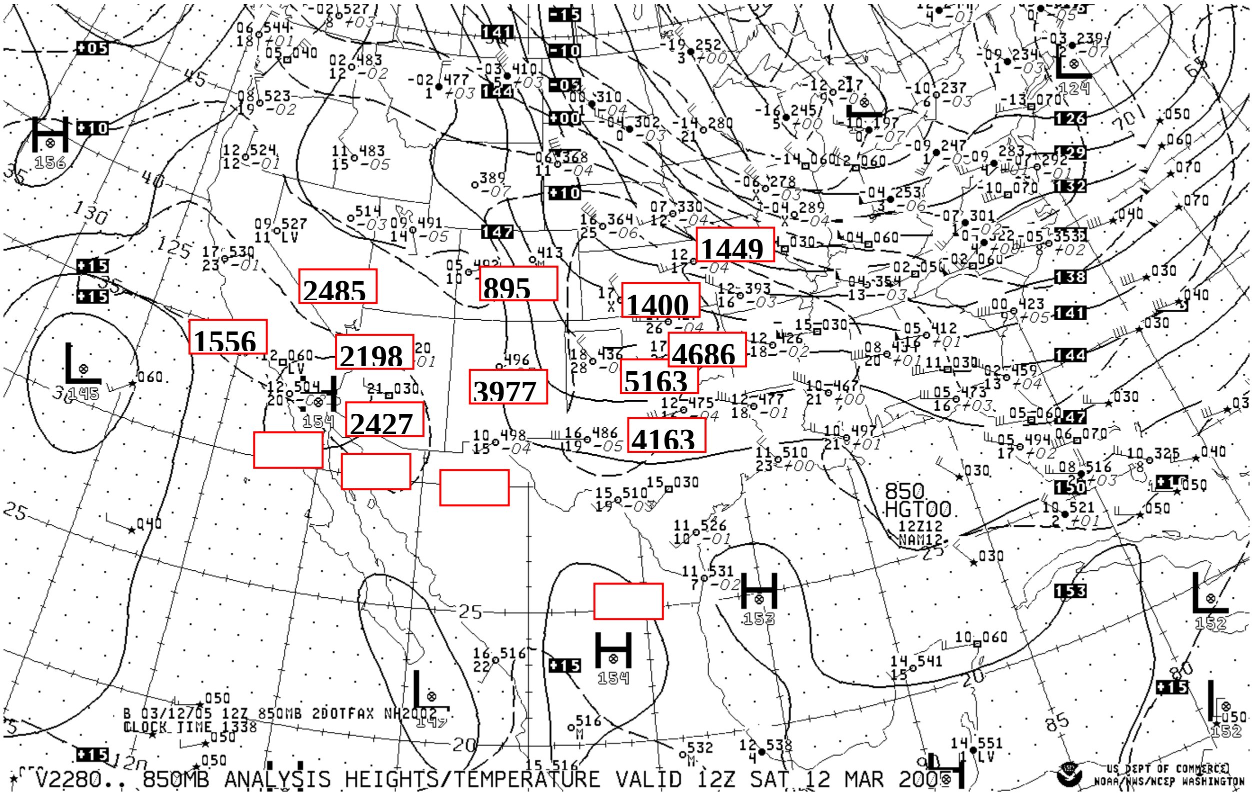

Figure F05-g-Used the 03/13/2005 week-ending data for the Mar 12 2005 12 z 850 mb chart. Courtesy of NOAA NWS. Blank Rectangles indicate no reports.

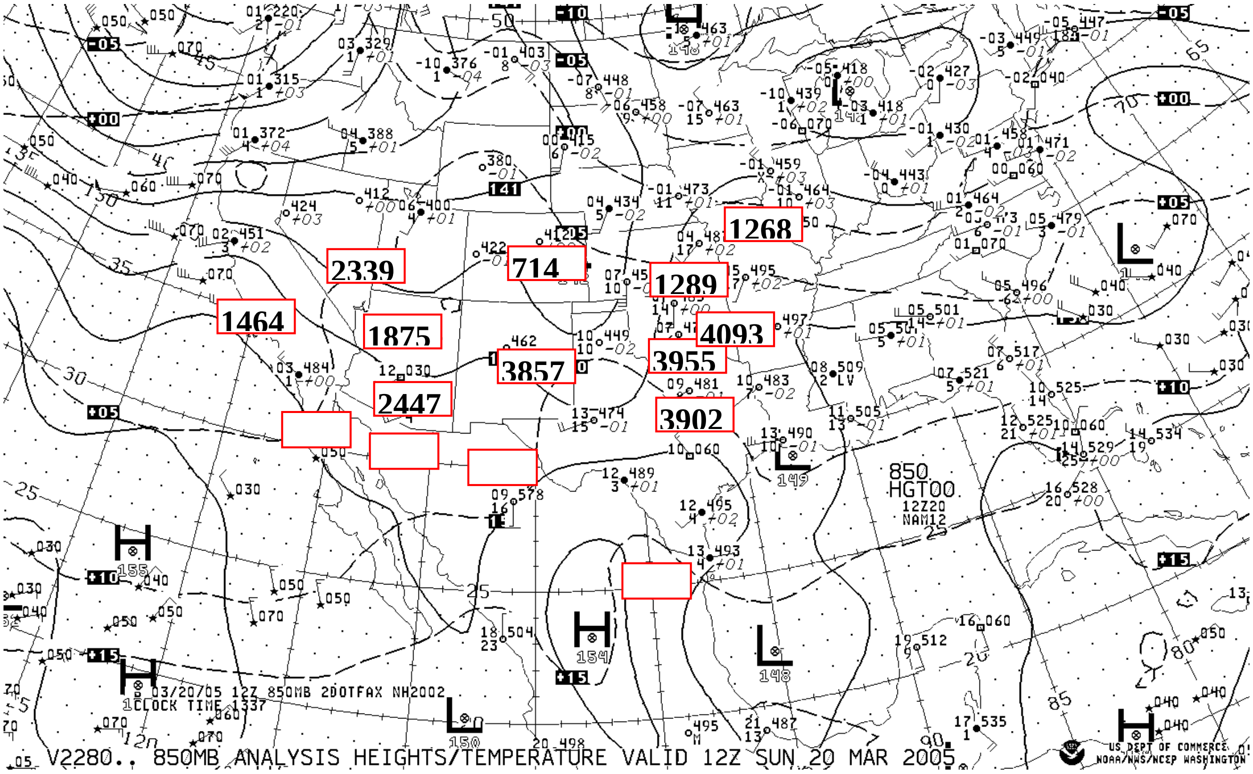

Figure F05-h-Used the 03/20/2005 week-ending data for the Mar 20 2005 12 z 850 mb chart. Courtesy of NOAA NWS. Blank Rectangles indicate no reports.

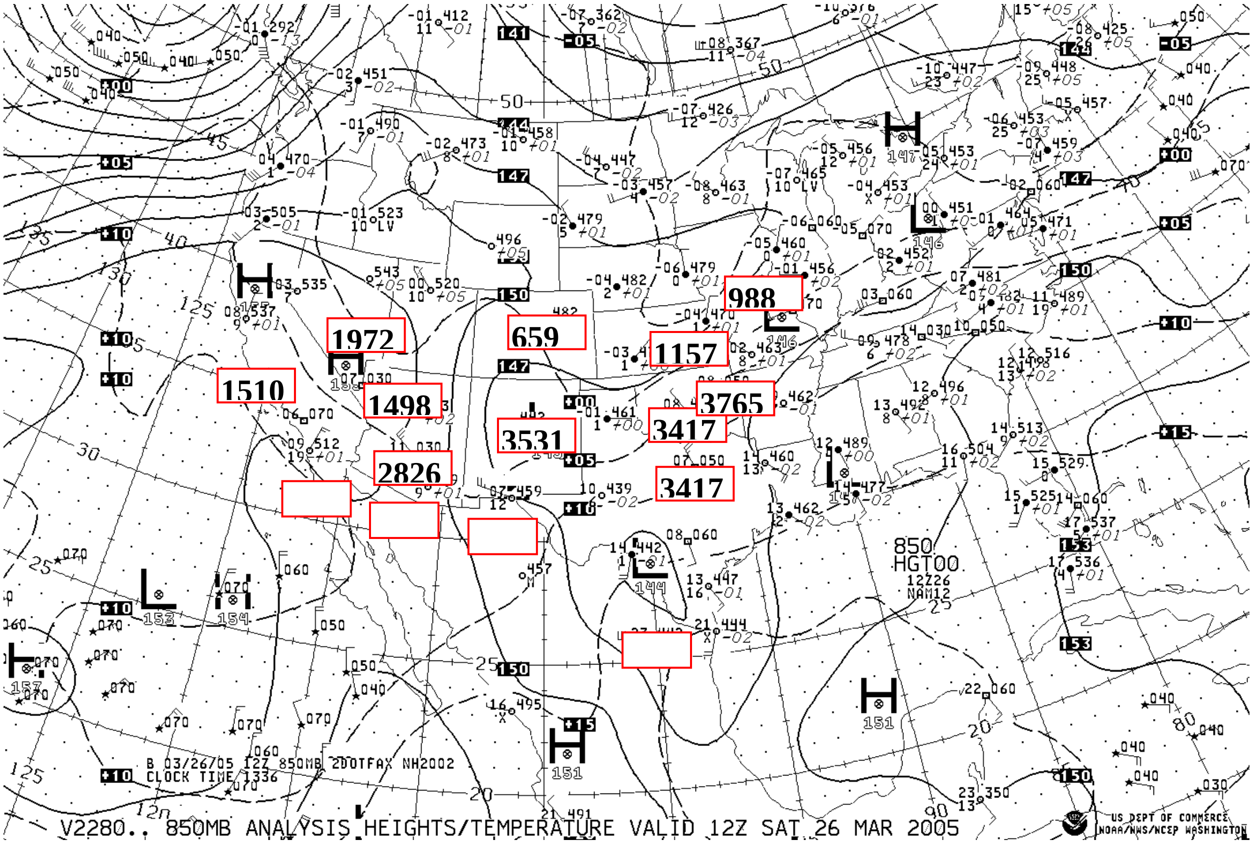

Figure F05-i1-Used the 03/27/2005 week-ending data for the Mar 26 2005 12 z 850 mb chart. Courtesy of NOAA NWS. Blank Rectangles indicate no reports.

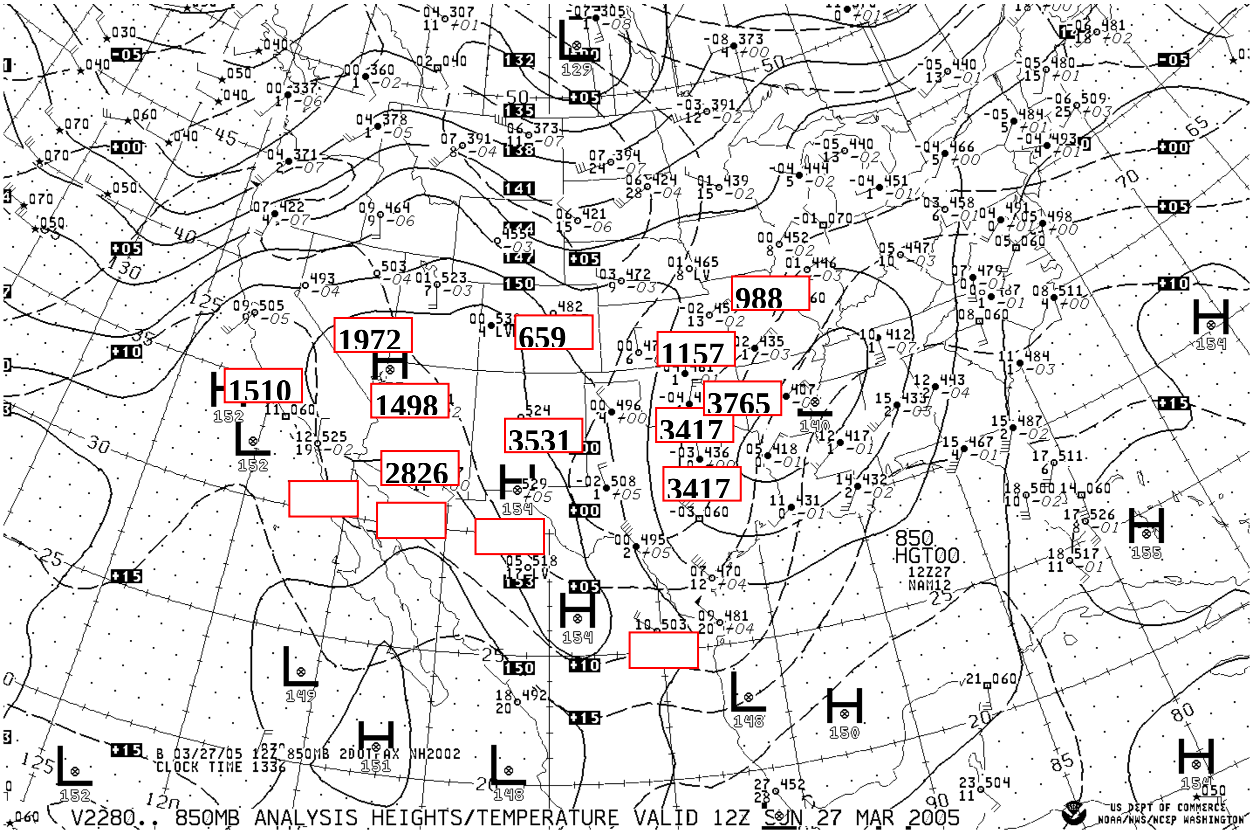

Figure F05-i2-Used the 03/27/2005 week-ending data for the Mar 27 2005 12 z 850 mb chart. Courtesy of NOAA NWS. Blank Rectangles indicate no reports.

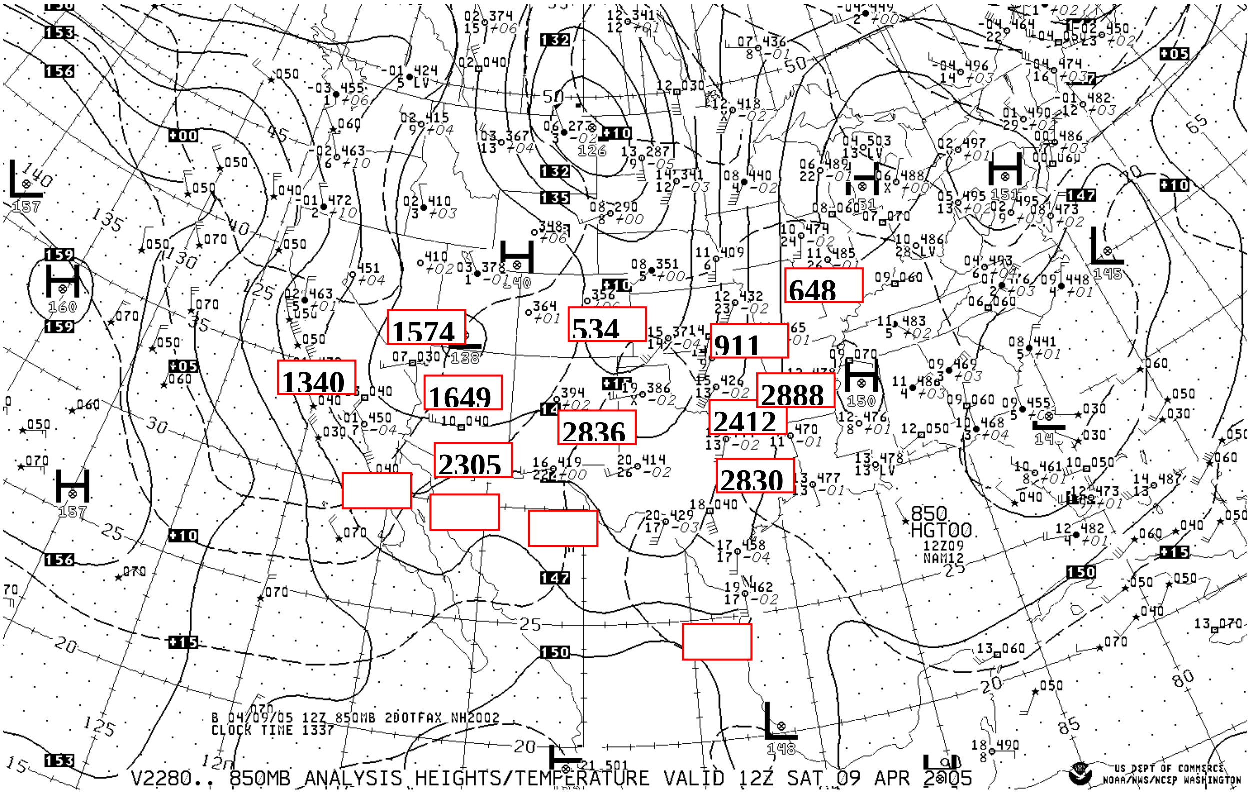

Figure F05-j1-Used the 04/10/2005 week-ending data for the Apr 09 2005 12 z 850 mb chart. Courtesy of NOAA NWS. Blank Rectangles indicate no reports.

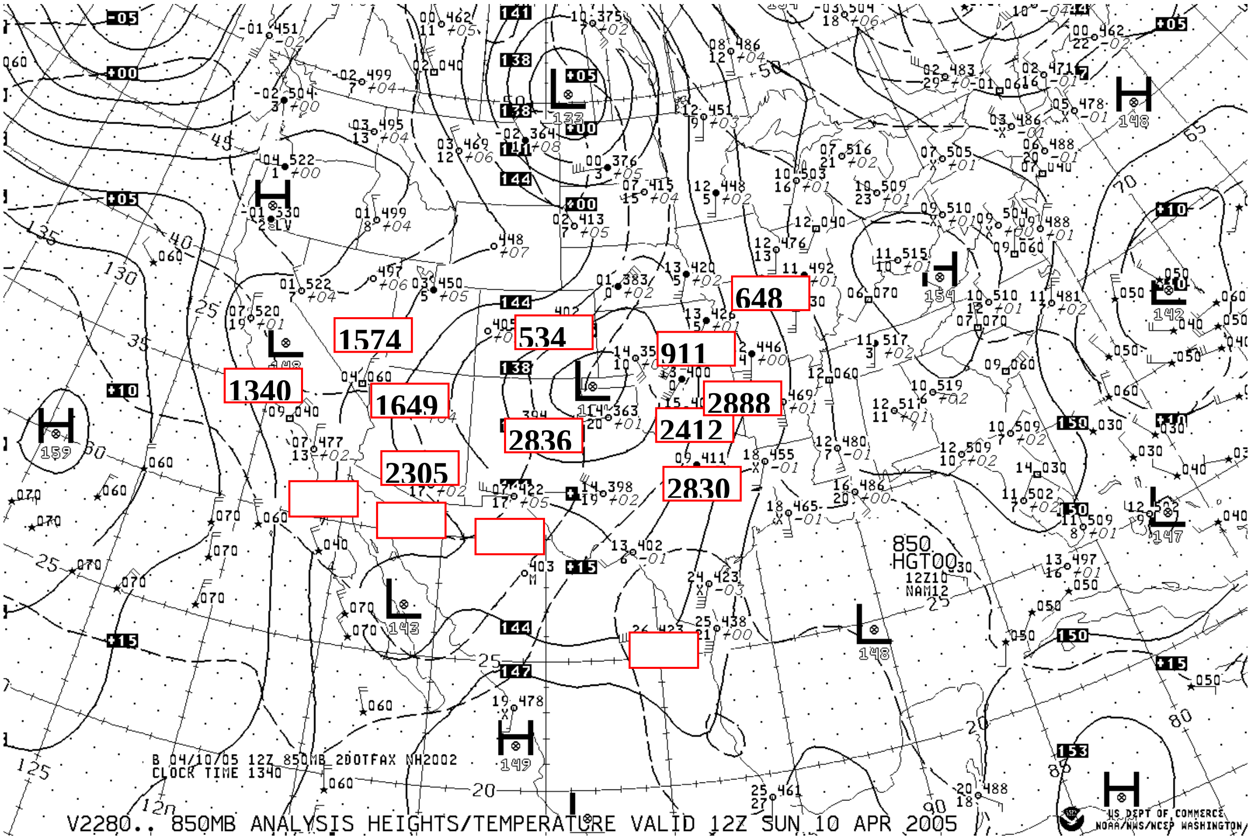

Figure F05-j2-Used the 04/10/2005 week-ending data for the Apr 10 2005 12 z 850 mb chart. Courtesy of NOAA NWS. Blank Rectangles indicate no reports.

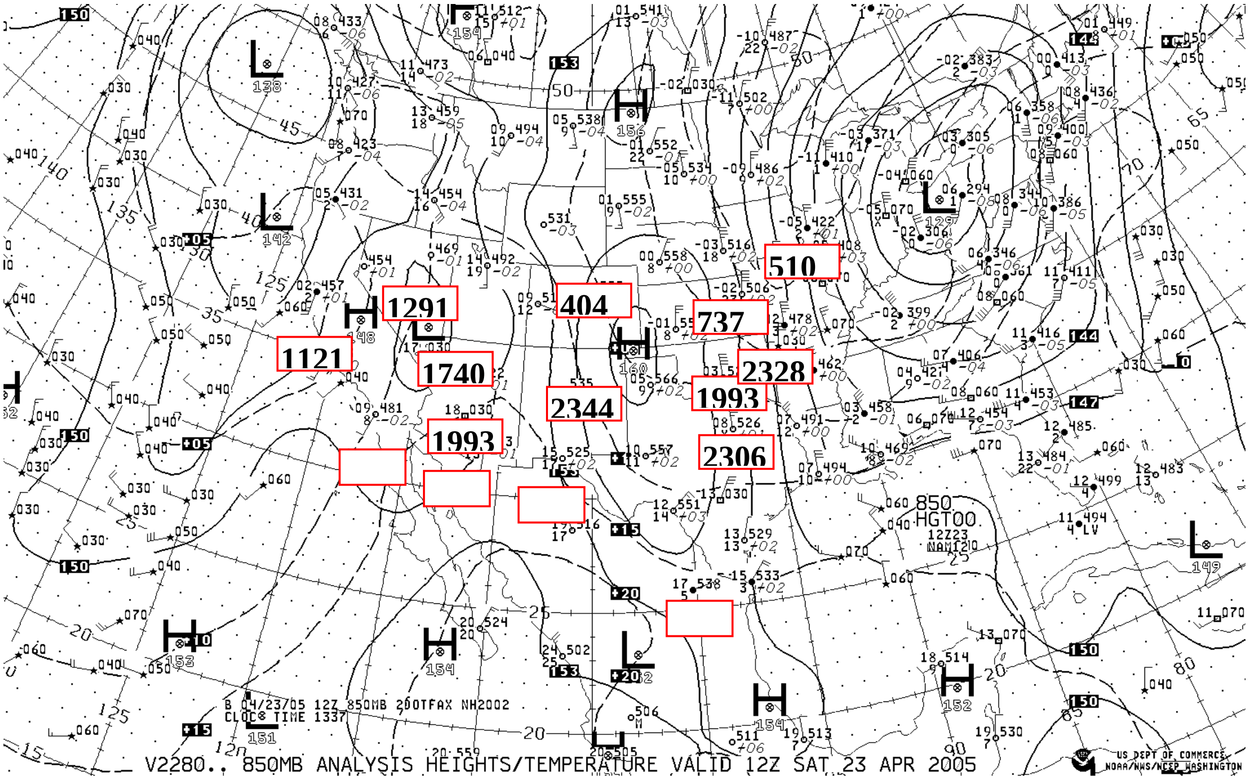

Figure F05-k-Used the 04/24/2005 week-ending data for the Apr 23 2005 12 z 850 mb chart. Courtesy of NOAA NWS. Blank Rectangles indicate no reports.

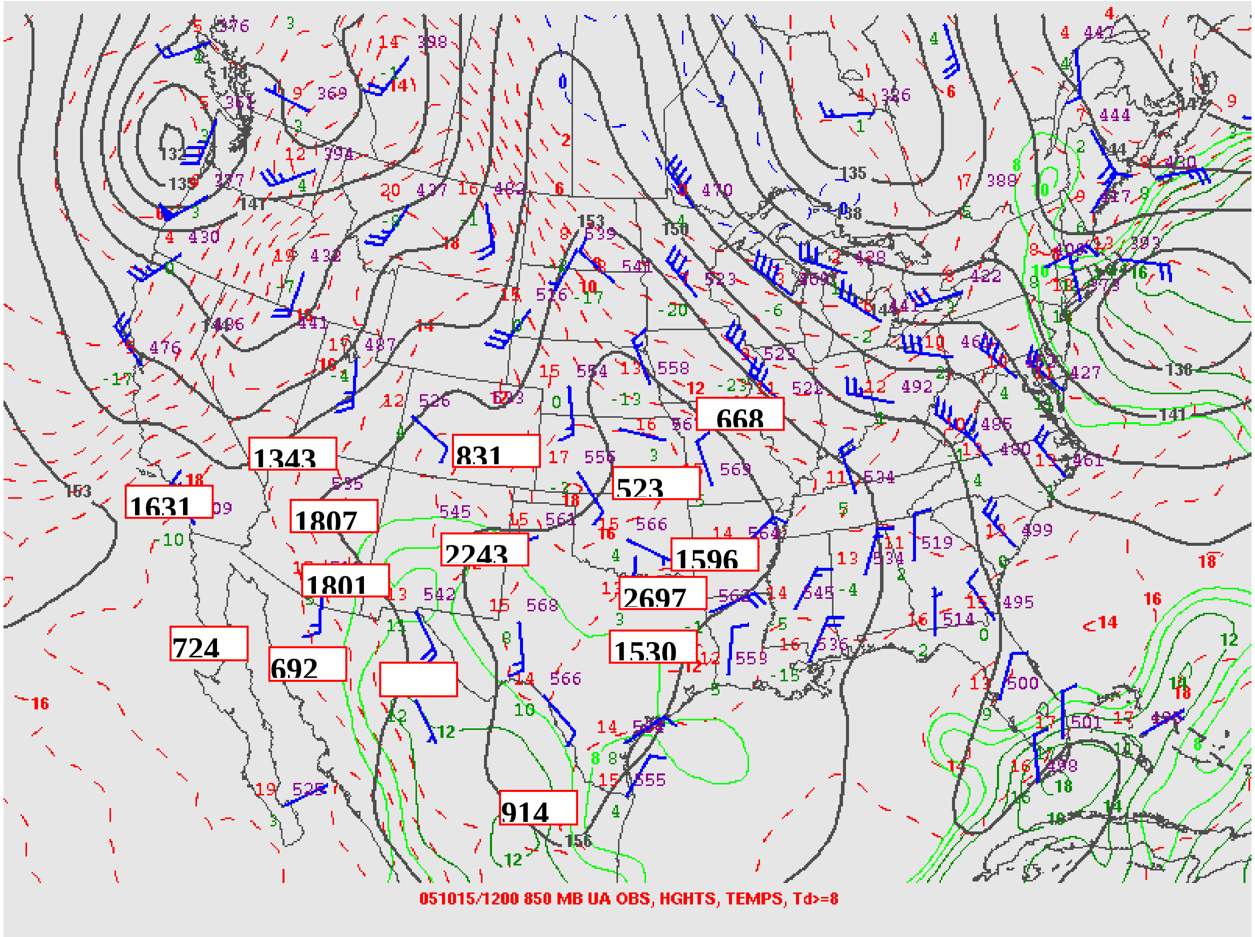

Figure 05G-a-Used the 10/16/2005 week-ending Physician influenza-like illness report data, with the Oct 15 2005 12 z 850 mb chart. (Courtesy of NOAA NWS; Google Flu Trends).

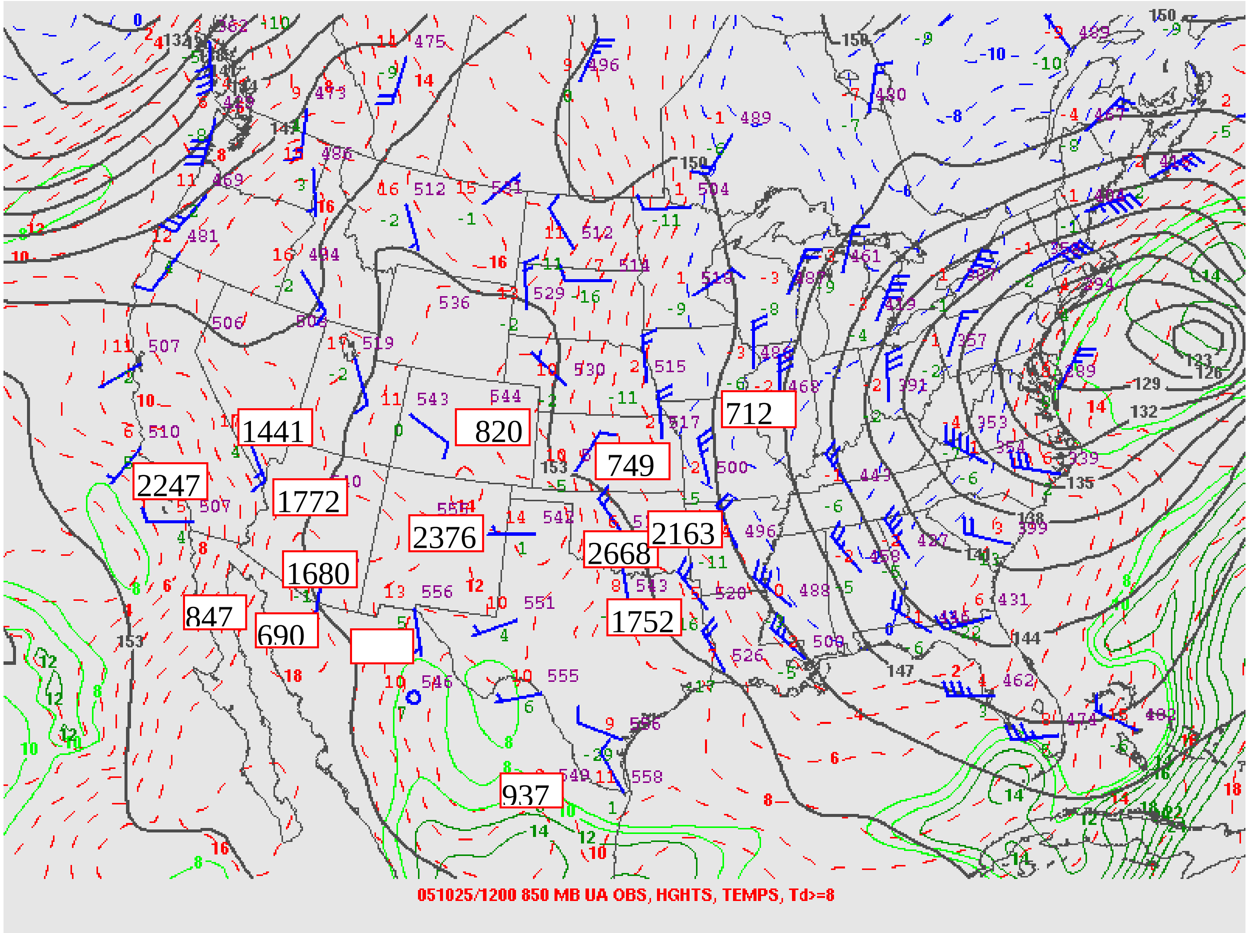

Figure 05G-b-Used the 10/23/2005 week-ending Physician influenza-like illness report data, with the Oct 25 2005 12 z 850 mb chart. (Courtesy of NOAA NWS; Google Flu Trends).

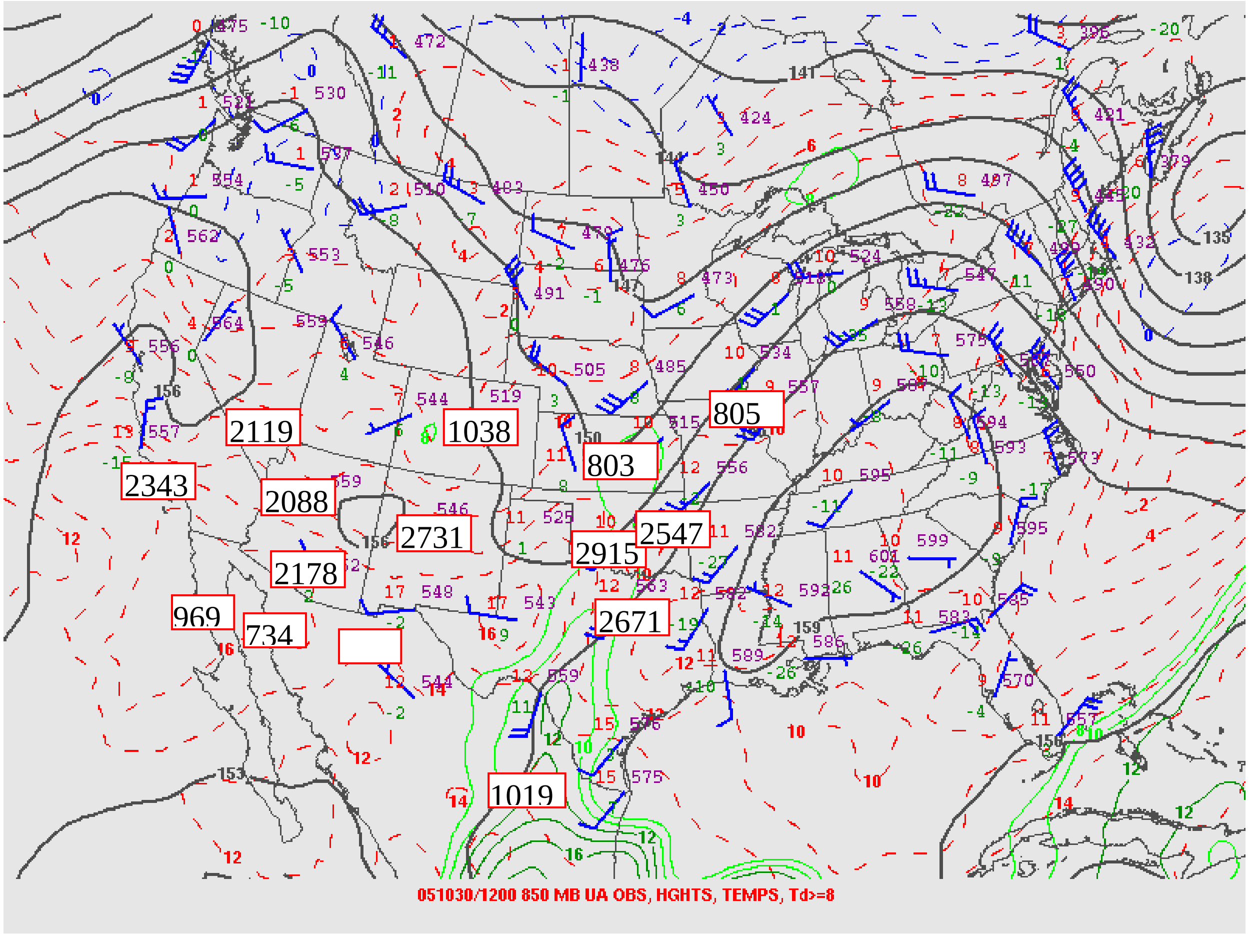

Figure 05G-c-Used the 10/30/2005 week-ending Physician influenza-like illness report data, with the Oct 30 2005 12 z 850 mb chart. (Courtesy of NOAA NWS; Google Flu Trends).

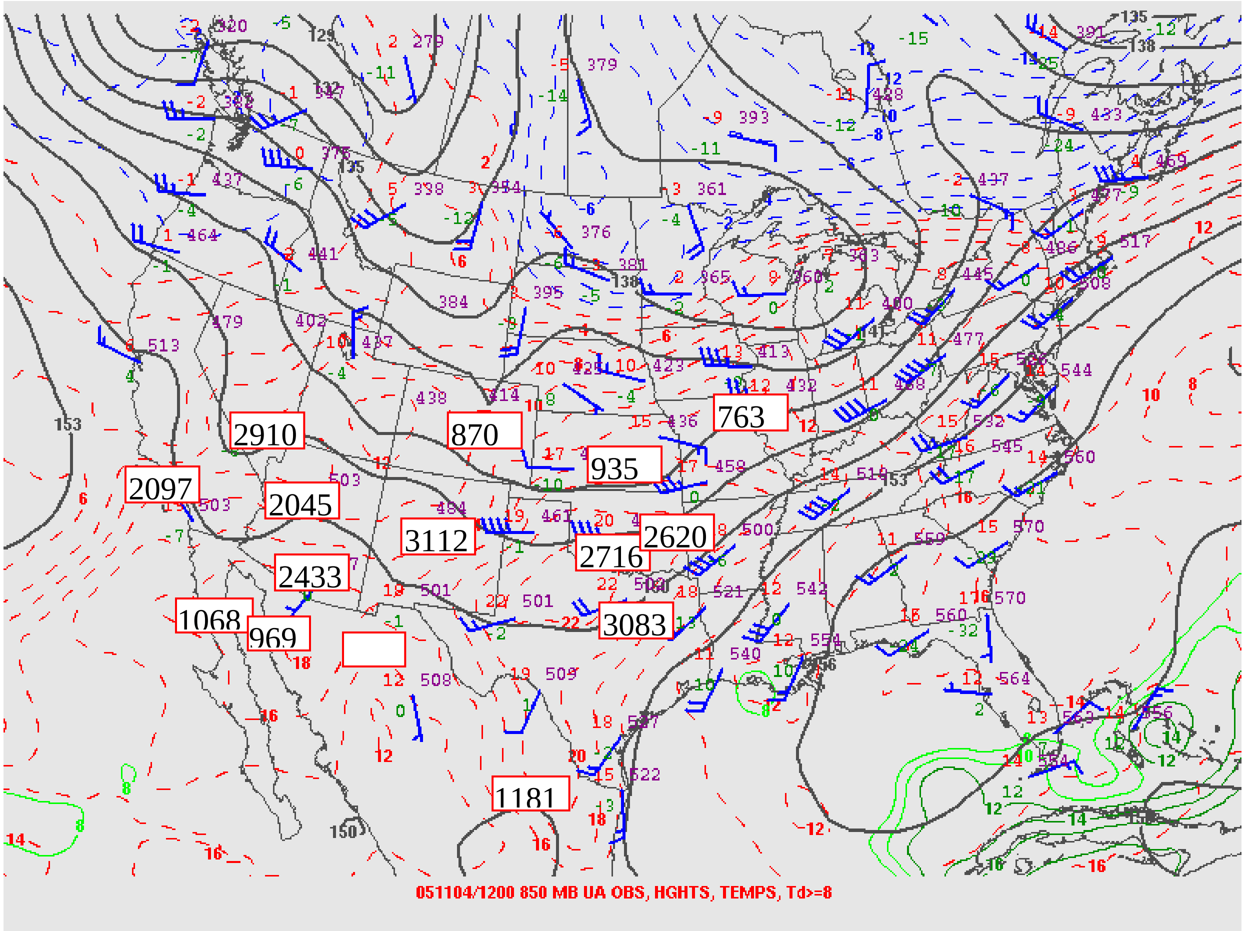

Figure 05G-d-Used the 11/06/2005 week-ending Physician influenza-like illness report data, with the Nov 04 2005 12 z 850 mb chart. (Courtesy of NOAA NWS; Google Flu Trends).

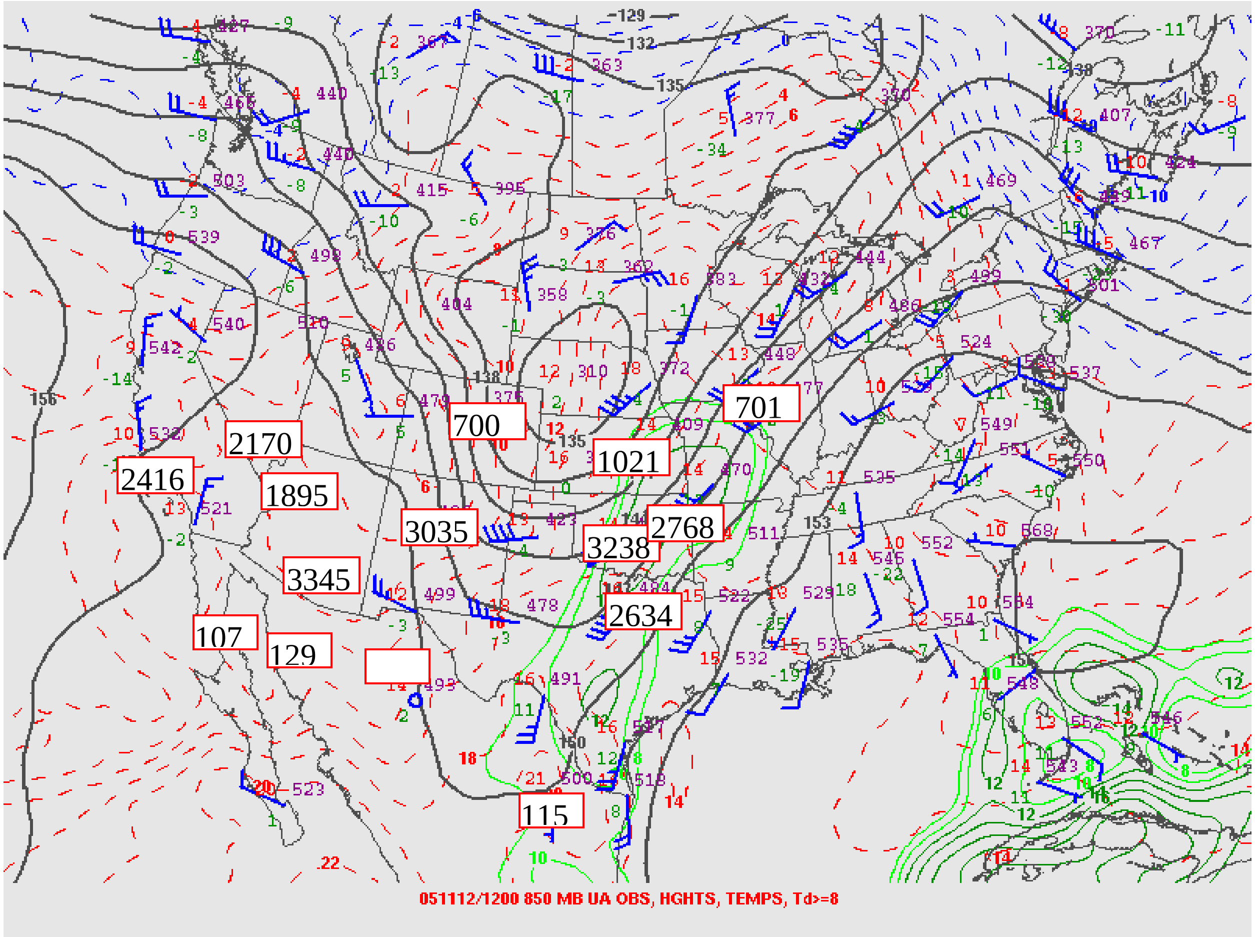

Figure 05G-e-Used the 11/13/2005 week-ending Physician influenza-like illness report data, with the Nov 12 2005 12 z 850 mb chart. (Courtesy of NOAA NWS; Google Flu Trends).

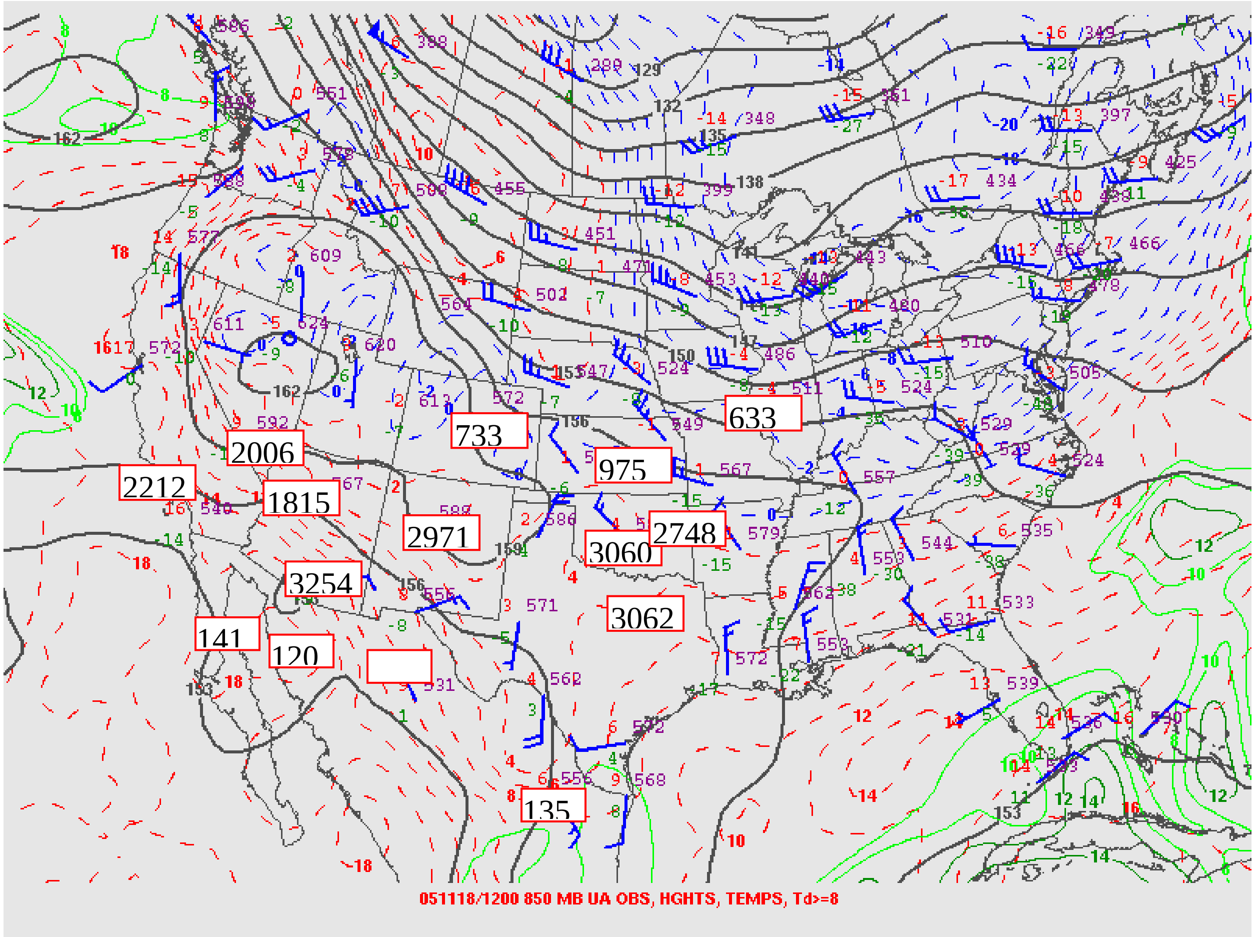

Figure 05G-f-Used the 11/20/2005 week-ending Physician influenza-like illness report data, with the Nov 18 2005 12 z 850 mb chart. (Courtesy of NOAA NWS; Google Flu Trends).

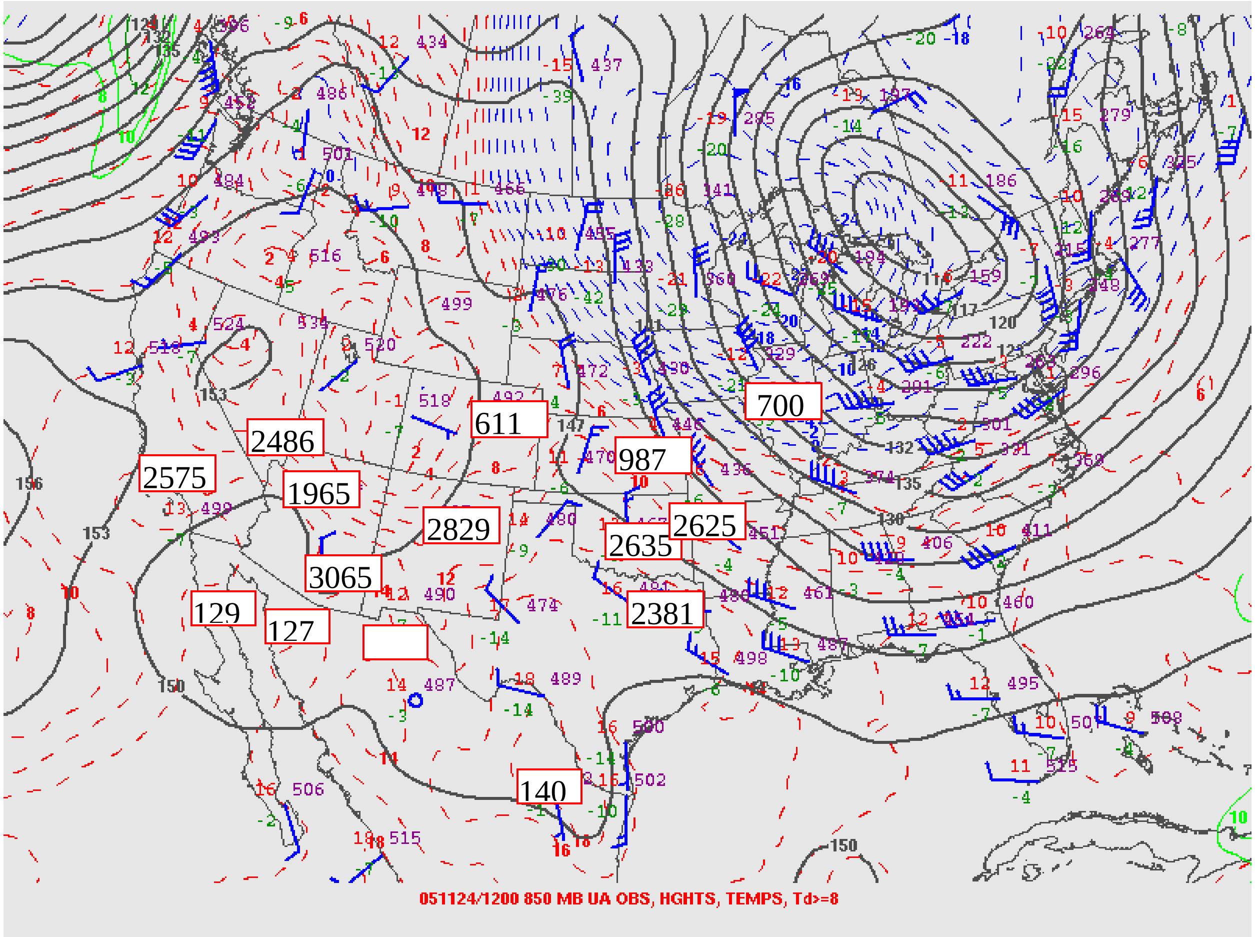

Figure 05G-g-Used the 11/27/2005 week-ending Physician influenza-like illness report data, with the Nov 24 2005 12 z 850 mb chart. (Courtesy of NOAA NWS; Google Flu Trends).

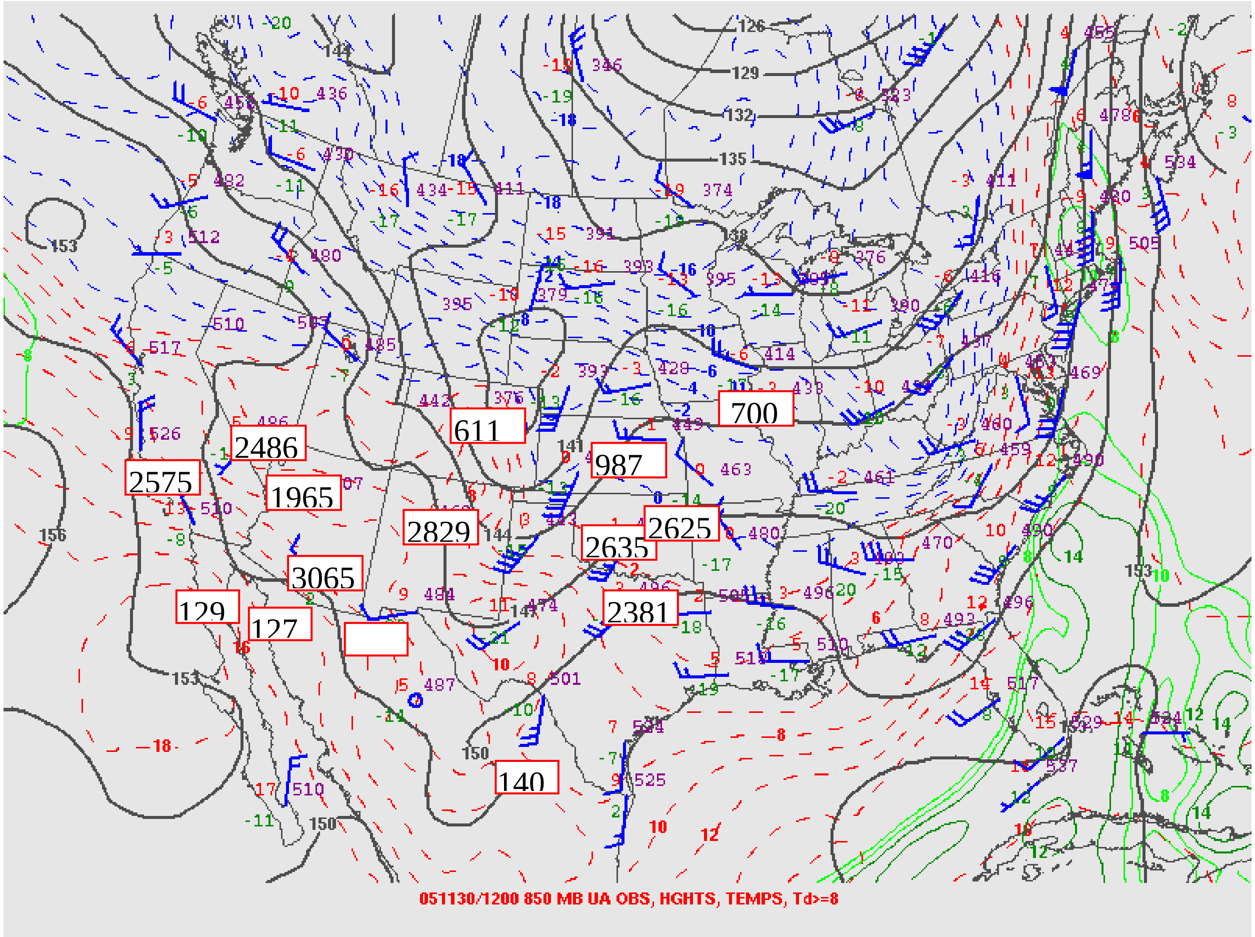

Figure 05G-h-Used the 11/27/2005 week-ending Physician influenza-like illness report data, with the Nov 30 2005 12 z 850 mb chart. (Courtesy of NOAA NWS; Google Flu Trends).

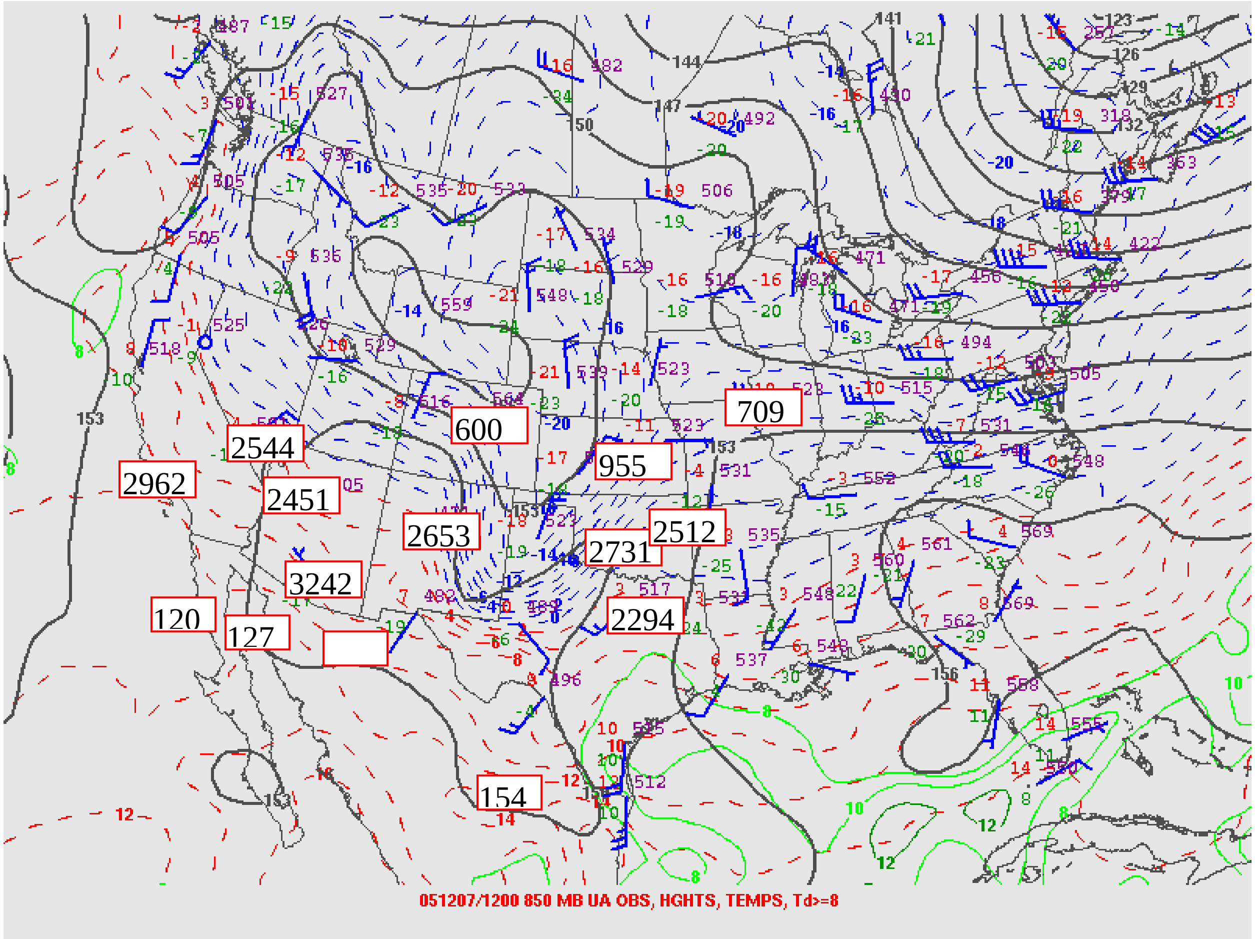

Figure 05G-i-Used the 12/04/2005 week-ending Physician influenza-like illness report data, with the Dec 07 2005 12 z 850 mb chart. (Courtesy of NOAA NWS; Google Flu Trends).

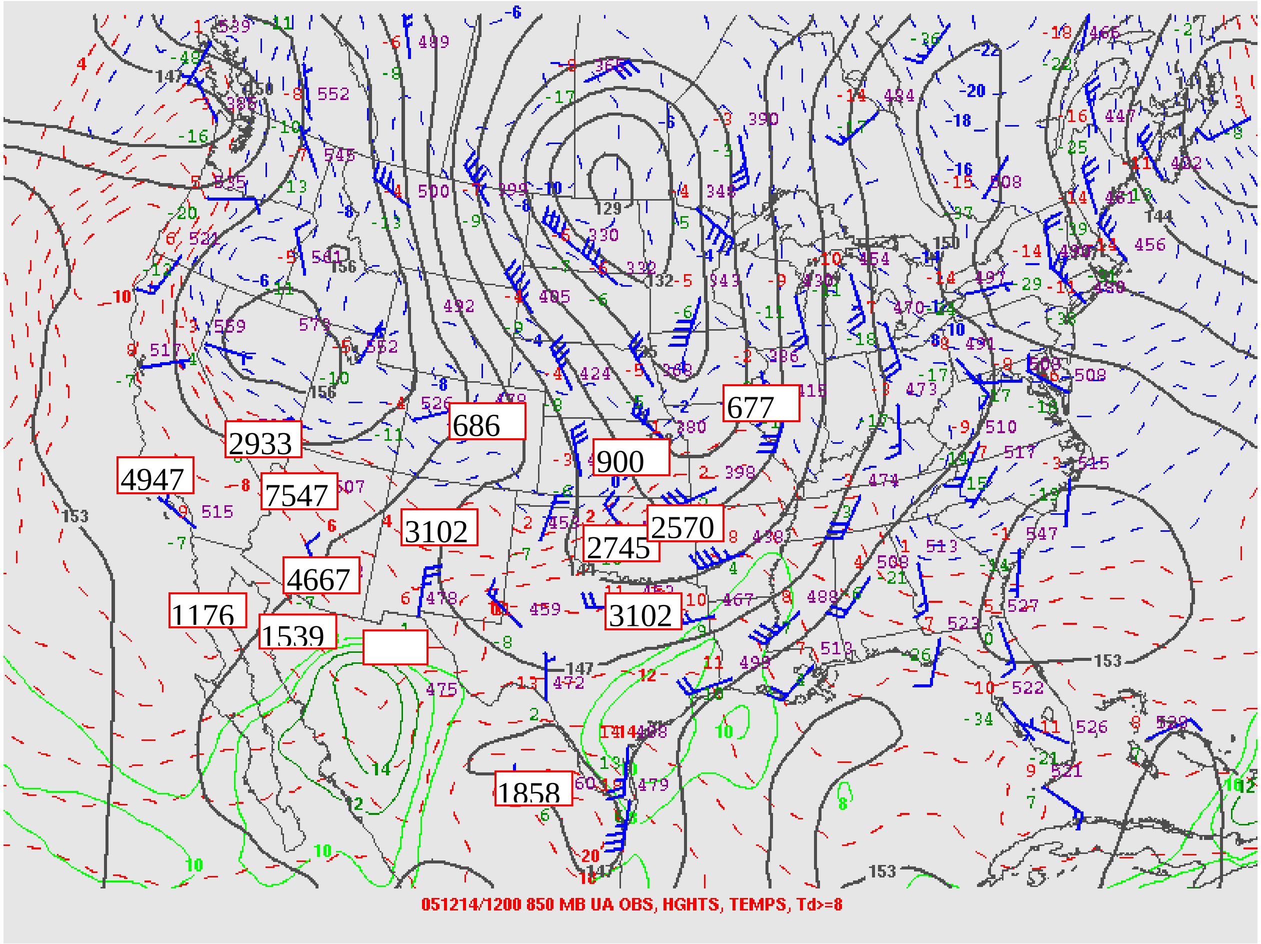

Figure 05G-j-Used the 12/11/2005 week-ending Physician influenza-like illness report data, with the Dec 14 2005 12 z 850 mb chart. (Courtesy of NOAA NWS; Google Flu Trends).

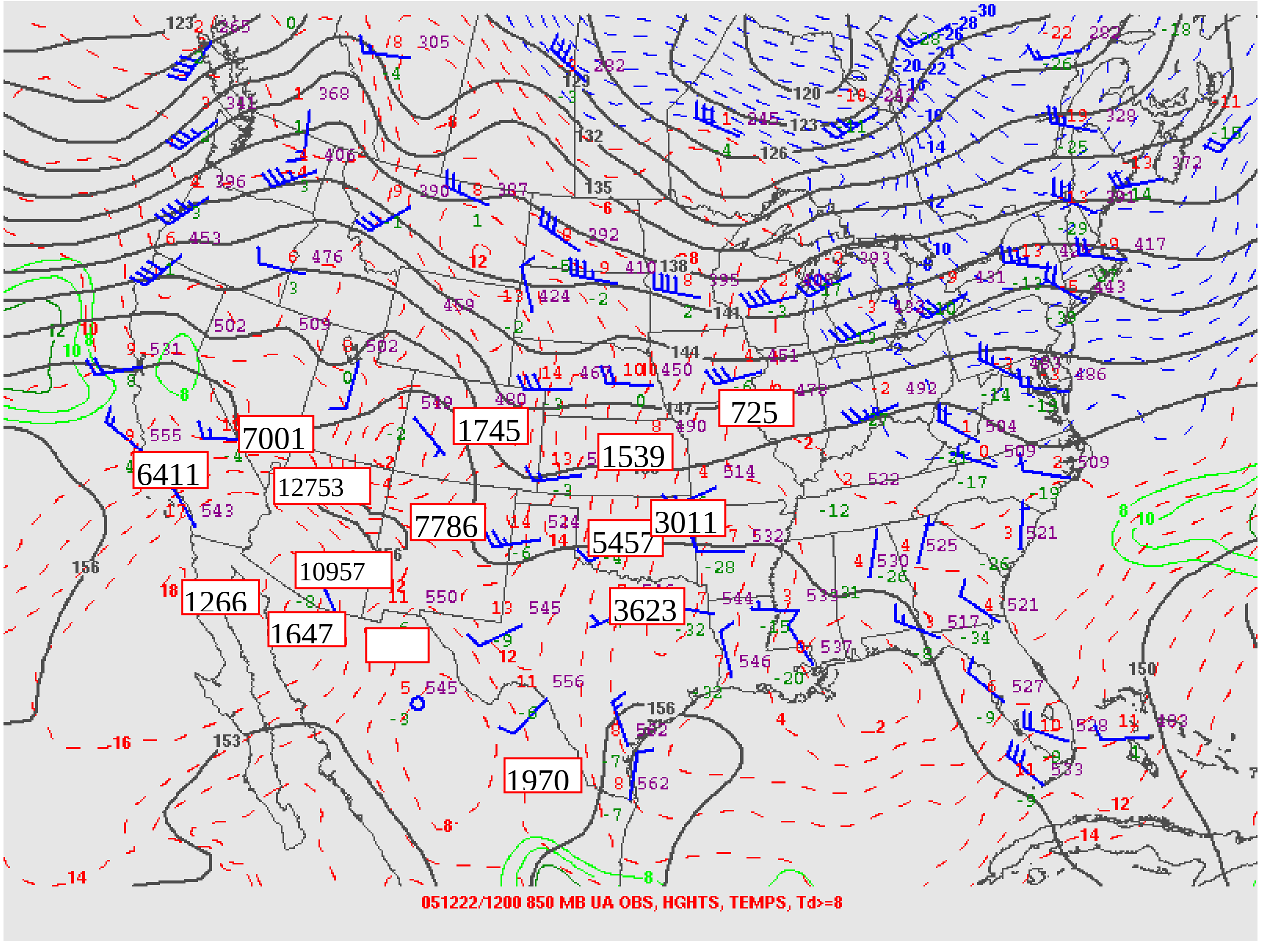

Figure 05G-k-Used the 12/25/2005 week-ending Physician influenza-like illness report data, with the Dec 22 2005 12 z 850 mb chart. (Courtesy of NOAA NWS; Google Flu Trends).

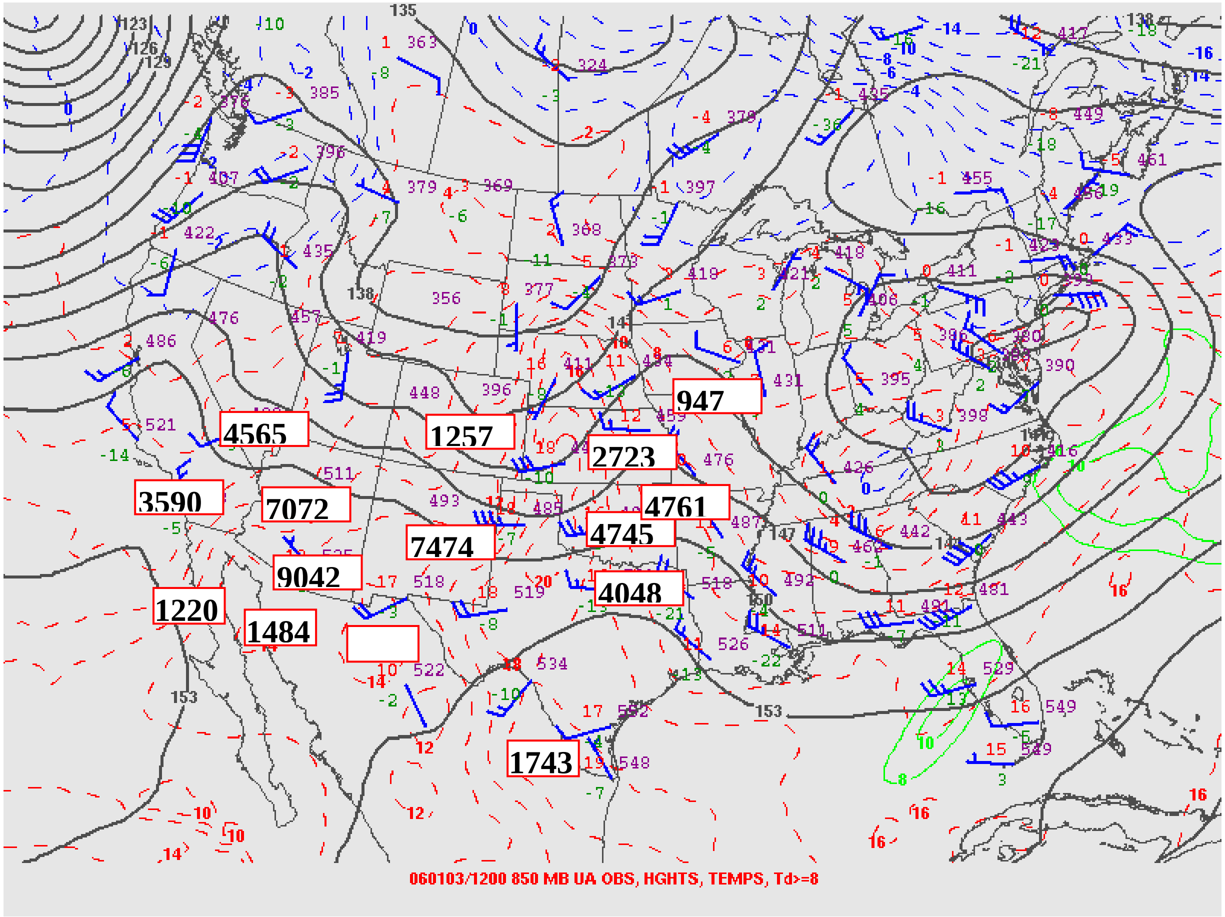

Figure F06-a-Used the 01/01/2006 week-ending Physician influenza-like illness report data, with the Jan 03 2006 12 z 850 mb chart. (Courtesy of NOAA NWS; Google Flu Trends).

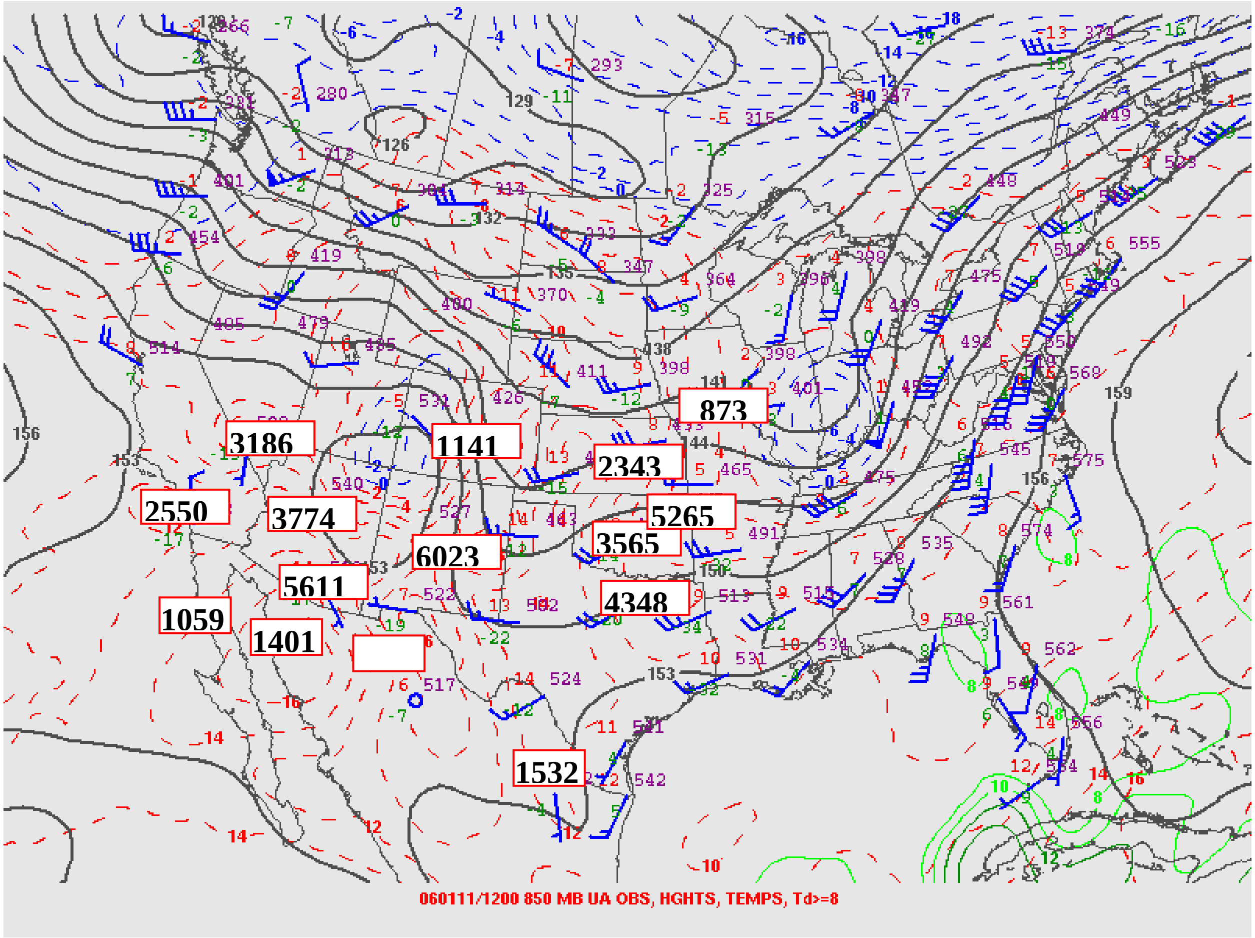

Figure F06-b-Used the 01/08/2006 week-ending Physician influenza-like illness report data, with the Jan 11 2006 12 z 850 mb chart. (Courtesy of NOAA NWS; Google Flu Trends).

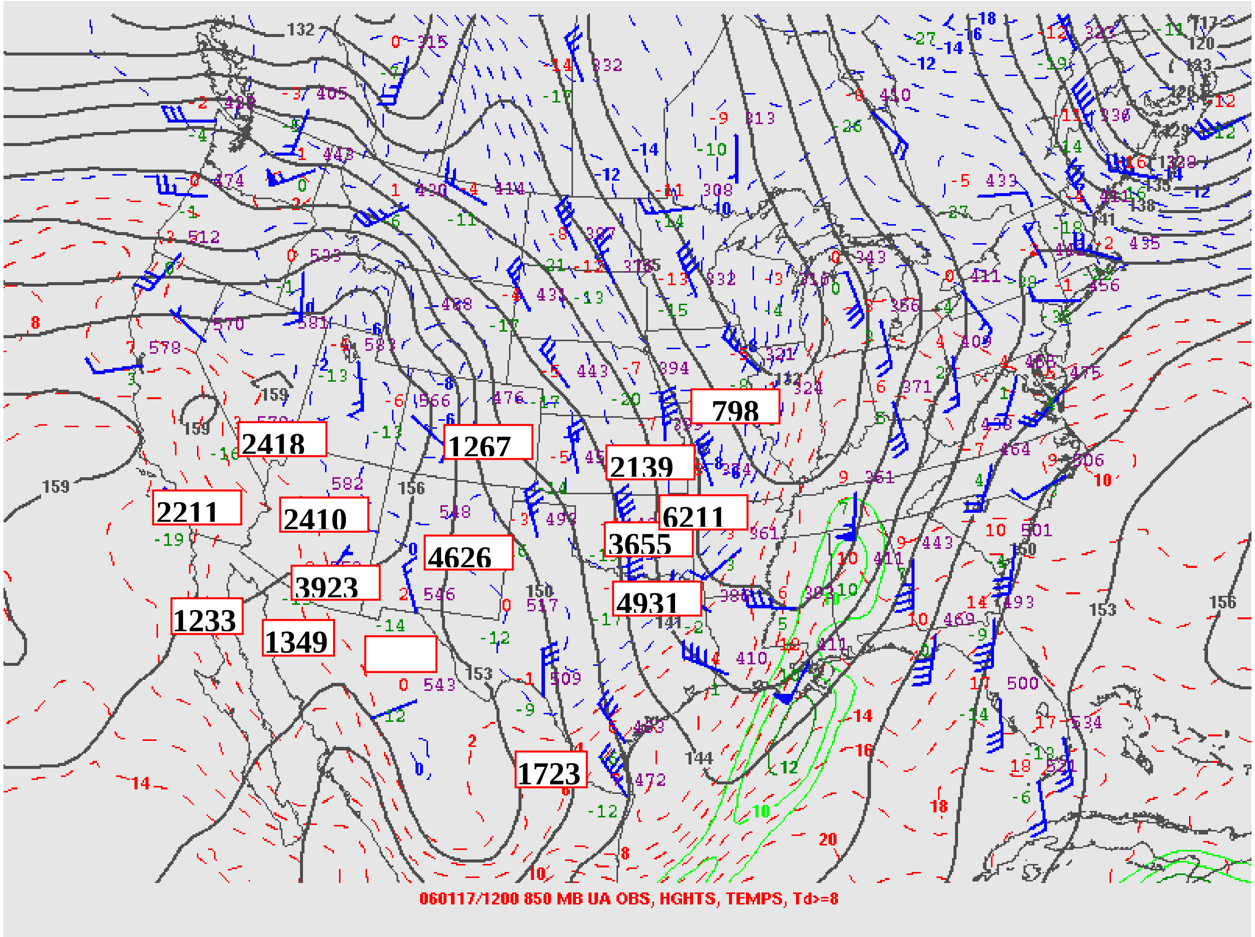

Figure F06-c-Used the 01/15/2006 week-ending Physician influenza-like illness report data, with the Jan 17 2006 12 z 850 mb chart. (Courtesy of NOAA NWS; Google Flu Trends).

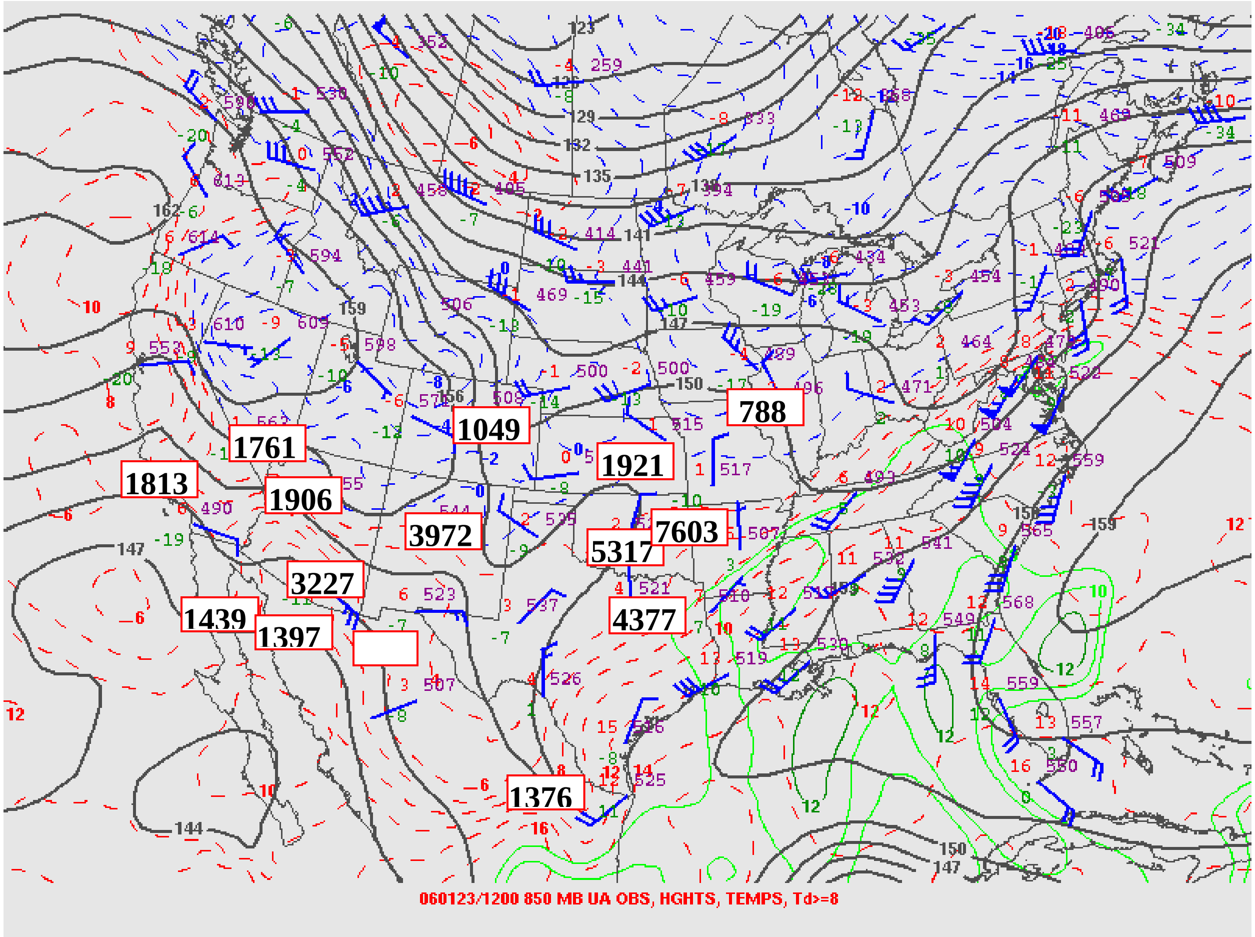

Figure F06-d-Used the 01/22/2006 week-ending Physician influenza-like illness report data, with the Jan 23 2006 12 z 850 mb chart. (Courtesy of NOAA NWS; Google Flu Trends).

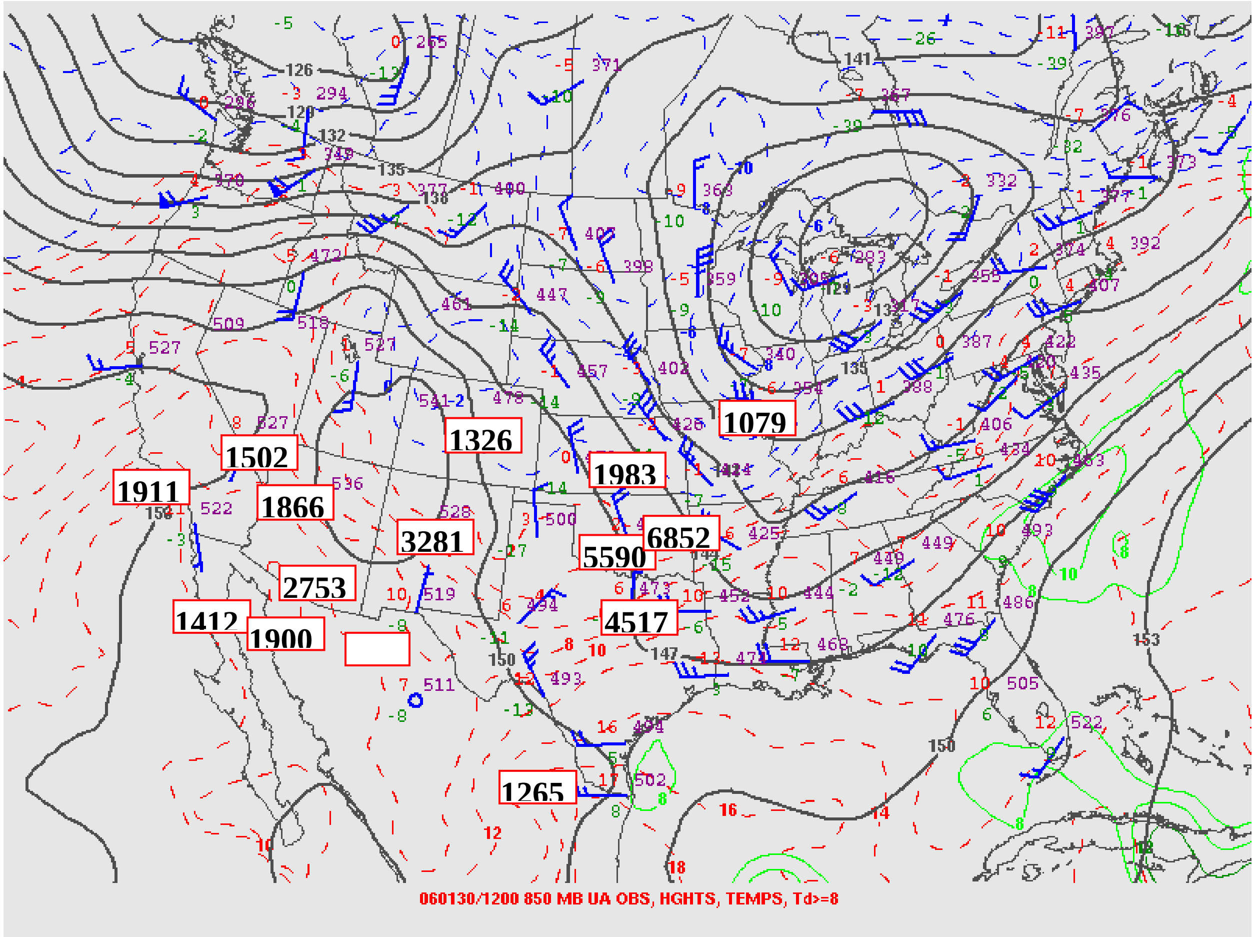

Figure F06-e-Used the 01/29/2006 week-ending Physician influenza-like illness report data, with the Jan 30 2006 12 z 850 mb chart. (Courtesy of NOAA NWS; Google Flu Trends).

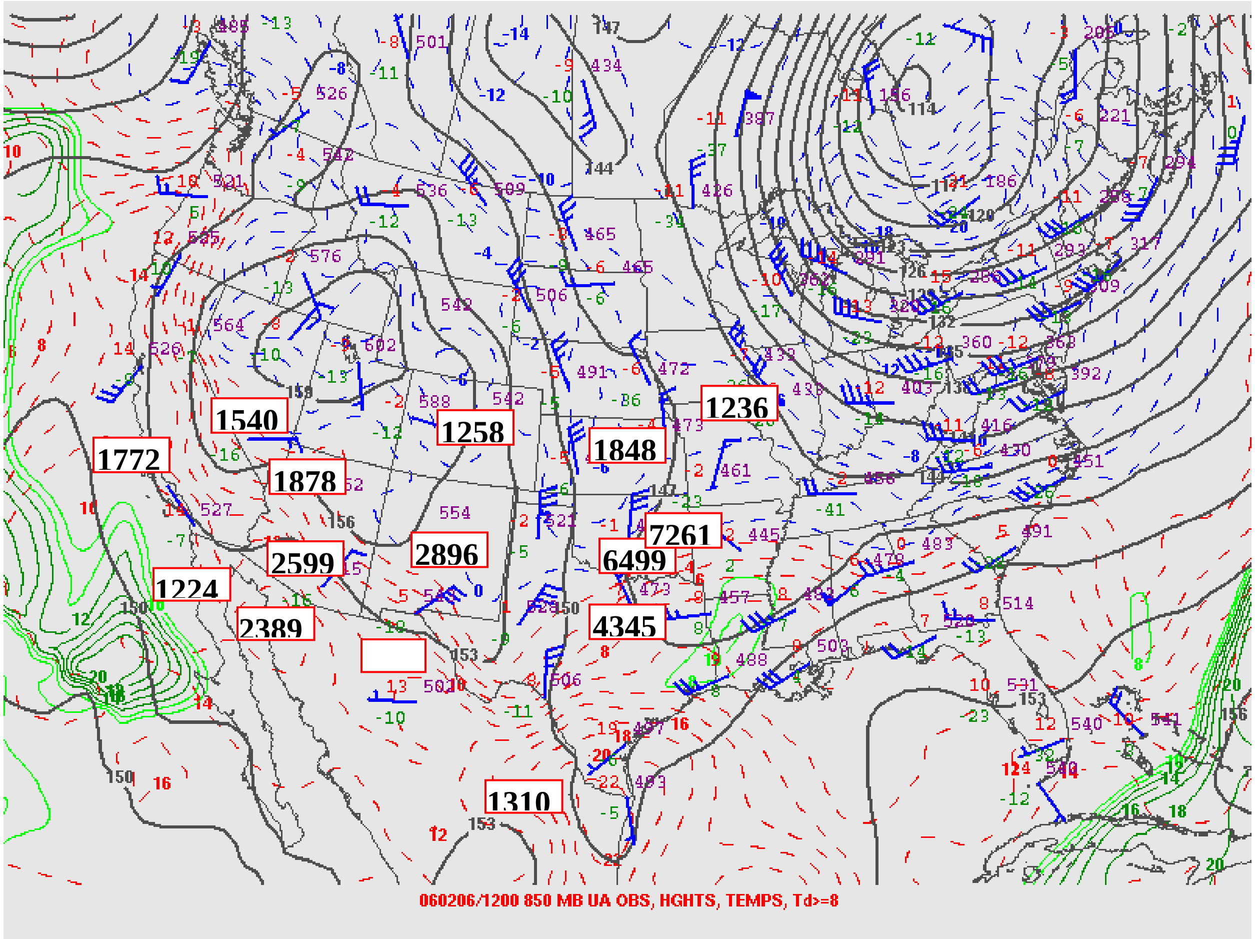

Figure F06-f-Used the 02/05/2006 week-ending Physician influenza-like illness report data, with the Feb 06 2006 12 z 850 mb chart. (Courtesy of NOAA NWS; Google Flu Trends).

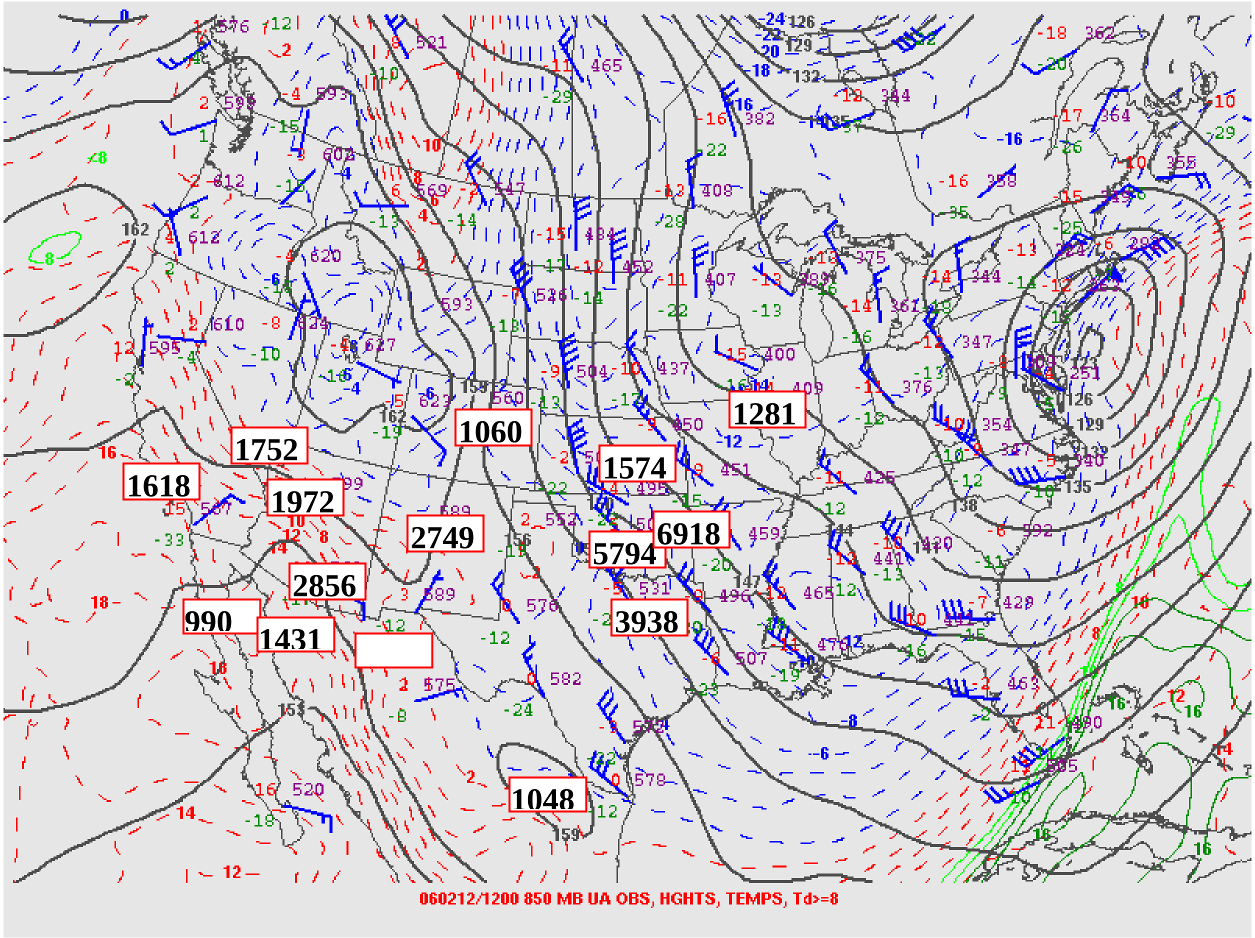

Figure F06-g-Used the 02/12/2006 week-ending Physician influenza-like illness report data, with the Feb 12 2006 12 z 850 mb chart. (Courtesy of NOAA NWS; Google Flu Trends).

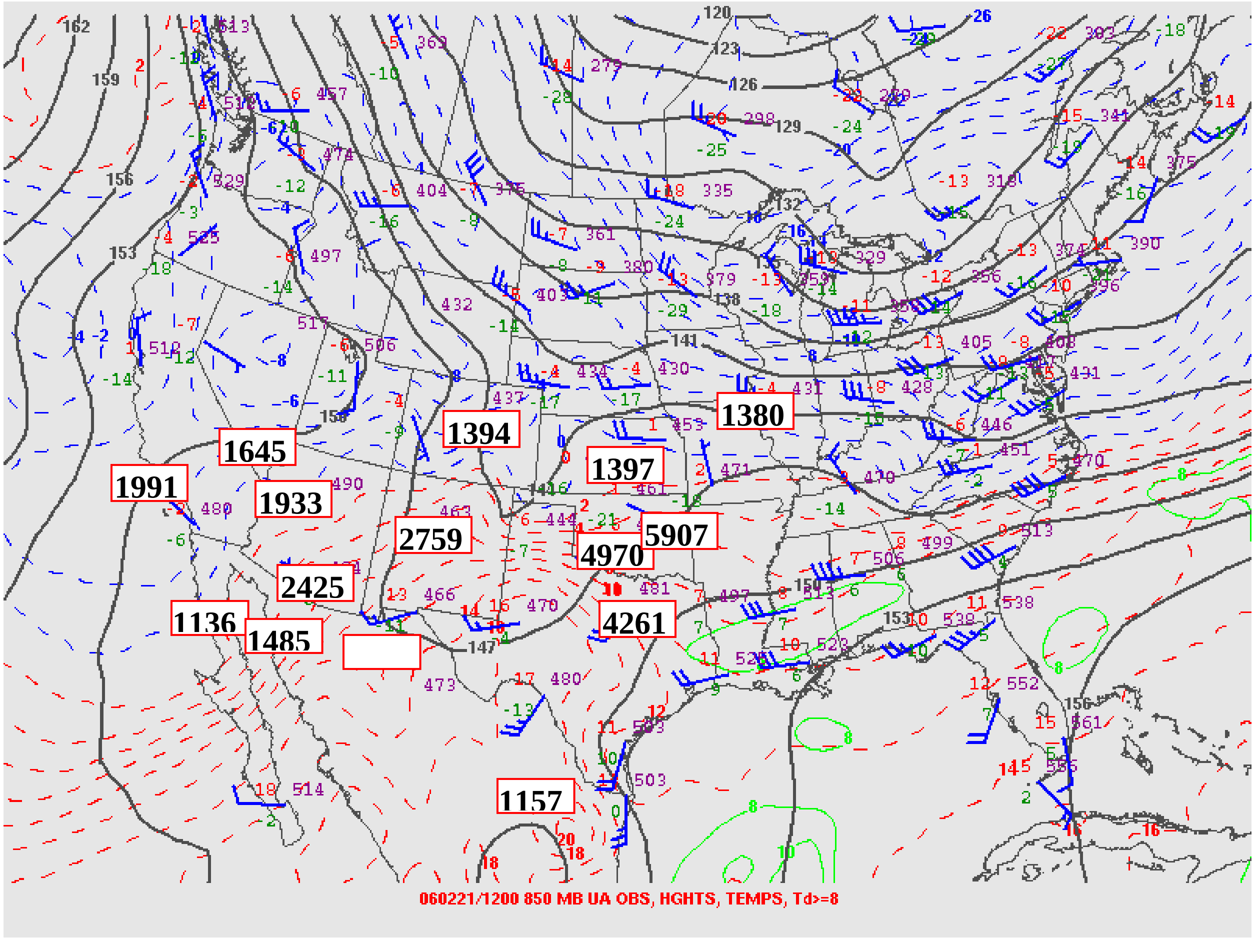

Figure F06-h-Used the 02/19/2006 week-ending Physician influenza-like illness report data, with the Feb 21 2006 12 z 850 mb chart. (Courtesy of NOAA NWS; Google Flu Trends).

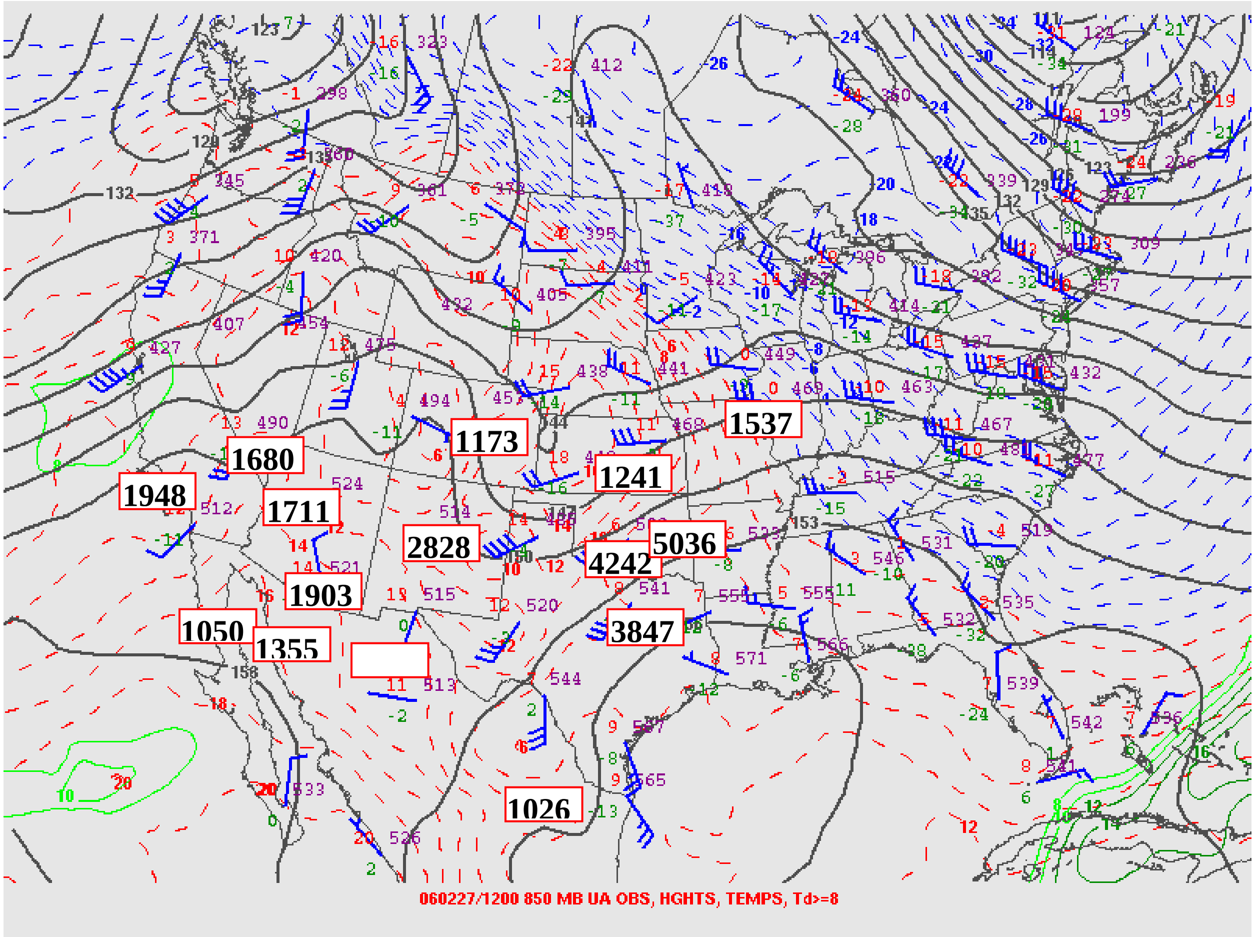

Figure F06-i-Used the 02/26/2006 week-ending Physician influenza-like illness report data, with the Feb 27 2006 12 z 850 mb chart. (Courtesy of NOAA NWS; Google Flu Trends).

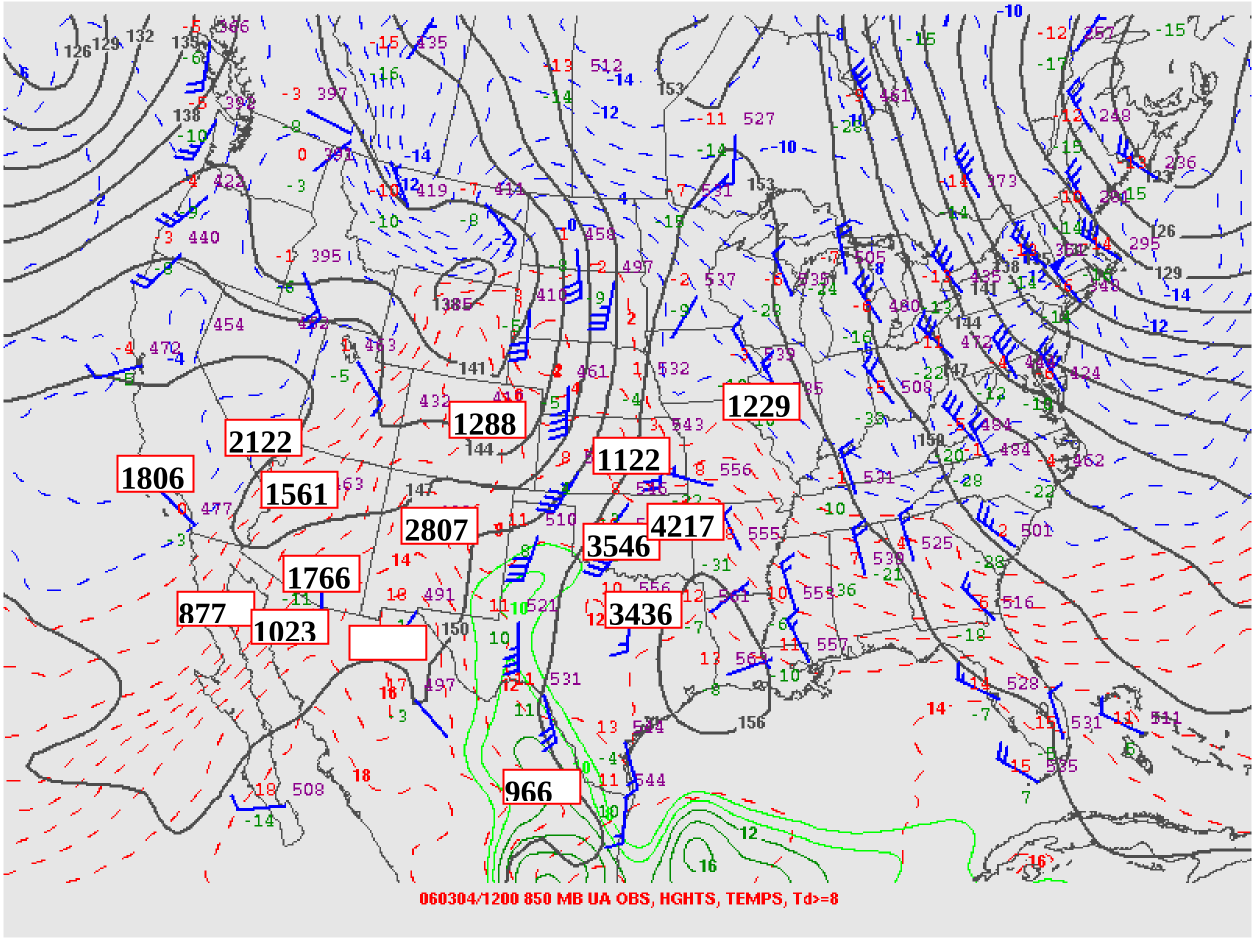

Figure F06-j1-Used the 03/05/2006 week-ending Physician influenza-like illness report data, with the Mar 04 2006 12 z 850 mb chart. (Courtesy of NOAA NWS; Google Flu Trends).

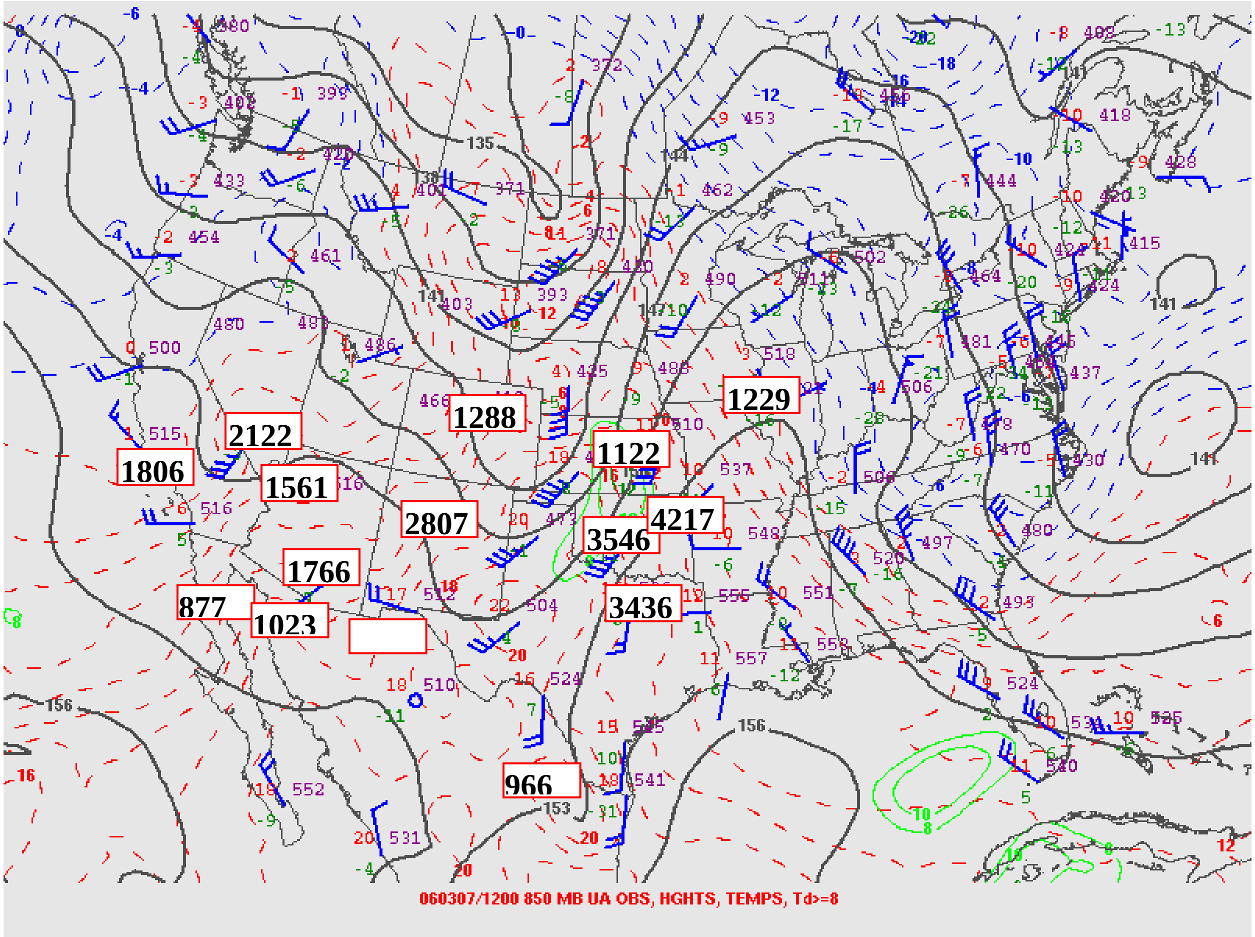

Figure F06-j2-Used the 03/05/2006 week-ending Physician influenza-like illness report data, with the Mar 07 2006 12 z 850 mb chart. (Courtesy of NOAA NWS; Google Flu Trends).

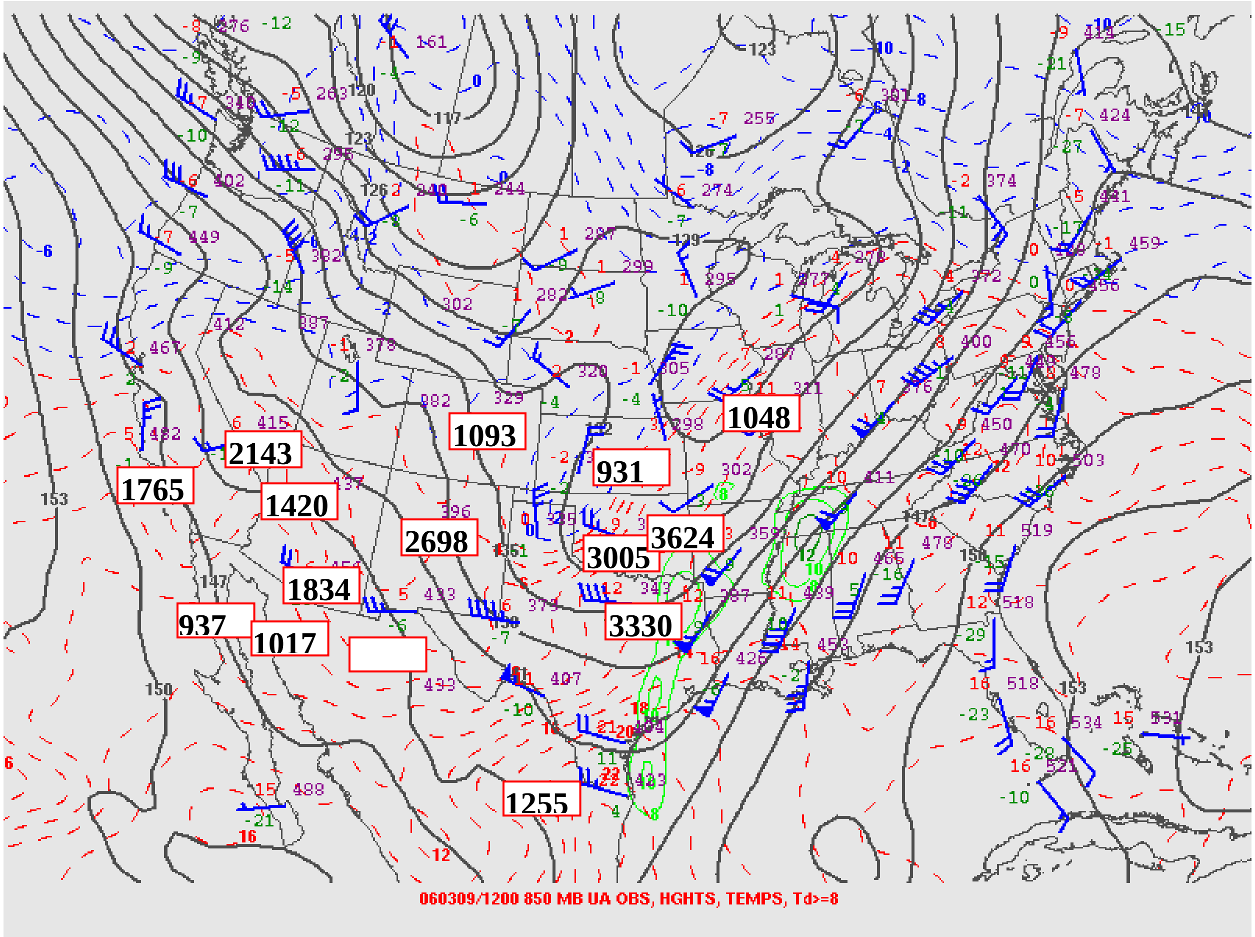

Figure F06-k1-Used the 03/12/2006 week-ending Physician influenza-like illness report data, with the Mar 09 2006 12 z 850 mb chart. (Courtesy of NOAA NWS; Google Flu Trends).

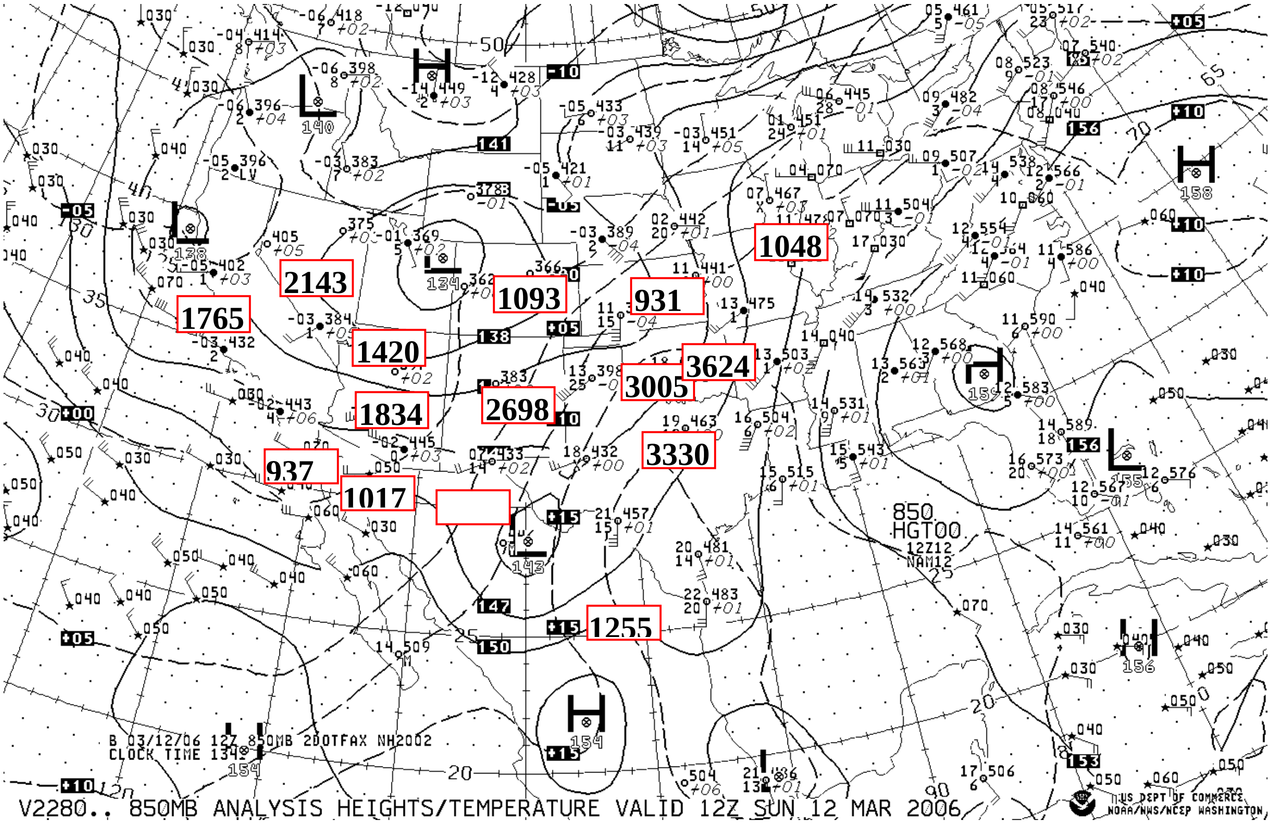

Figure F06-k2-Used the 03/12/2006 week-ending Physician influenza-like illness report data, with the Mar 12 2006 12 z 850 mb chart. (Courtesy of NOAA NWS; Google Flu Trends).

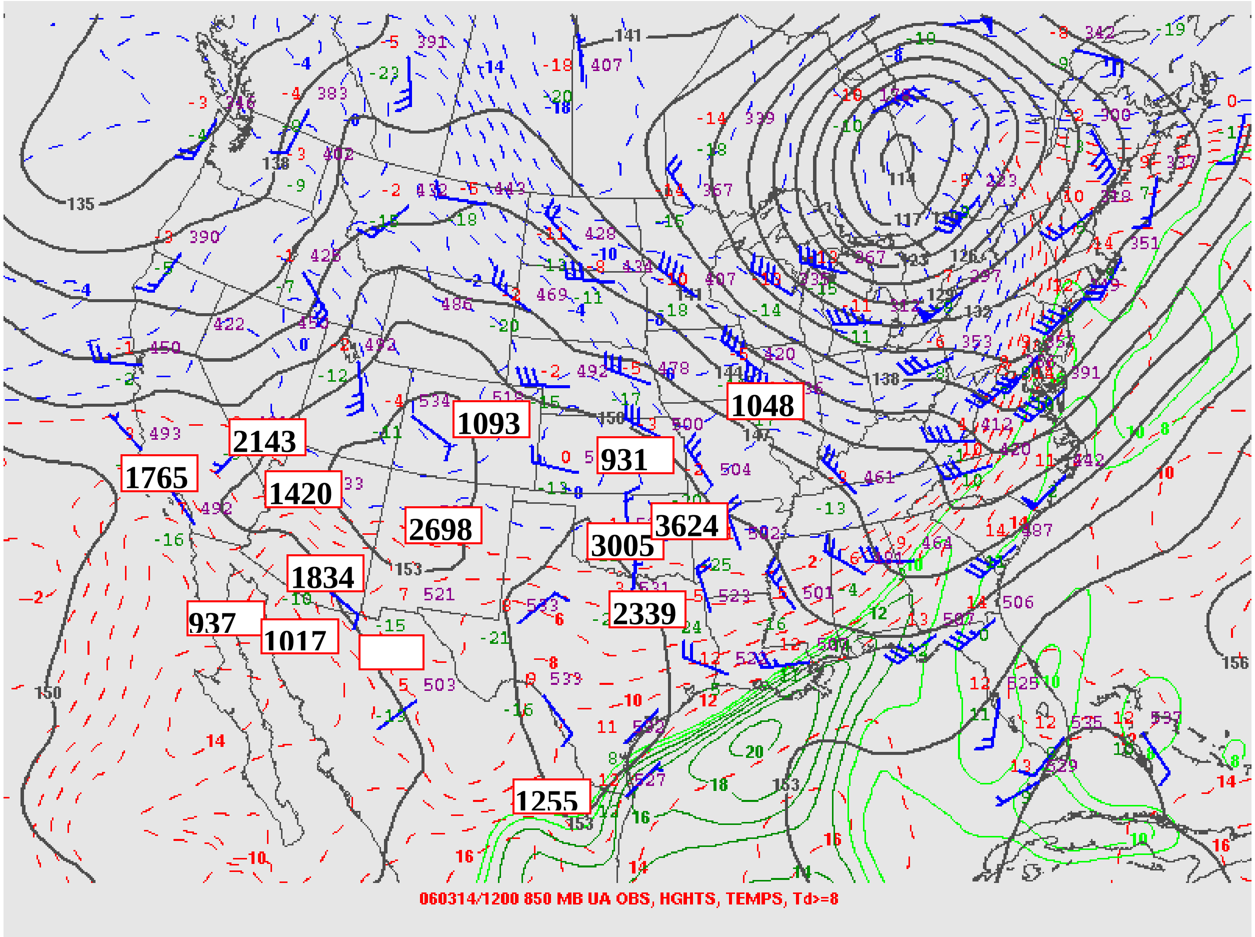

Figure F06-k3-Used the 03/12/2006 week-ending Physician influenza-like illness report data, with the Mar 14 2006 12 z 850 mb chart. (Courtesy of NOAA NWS; Google Flu Trends).

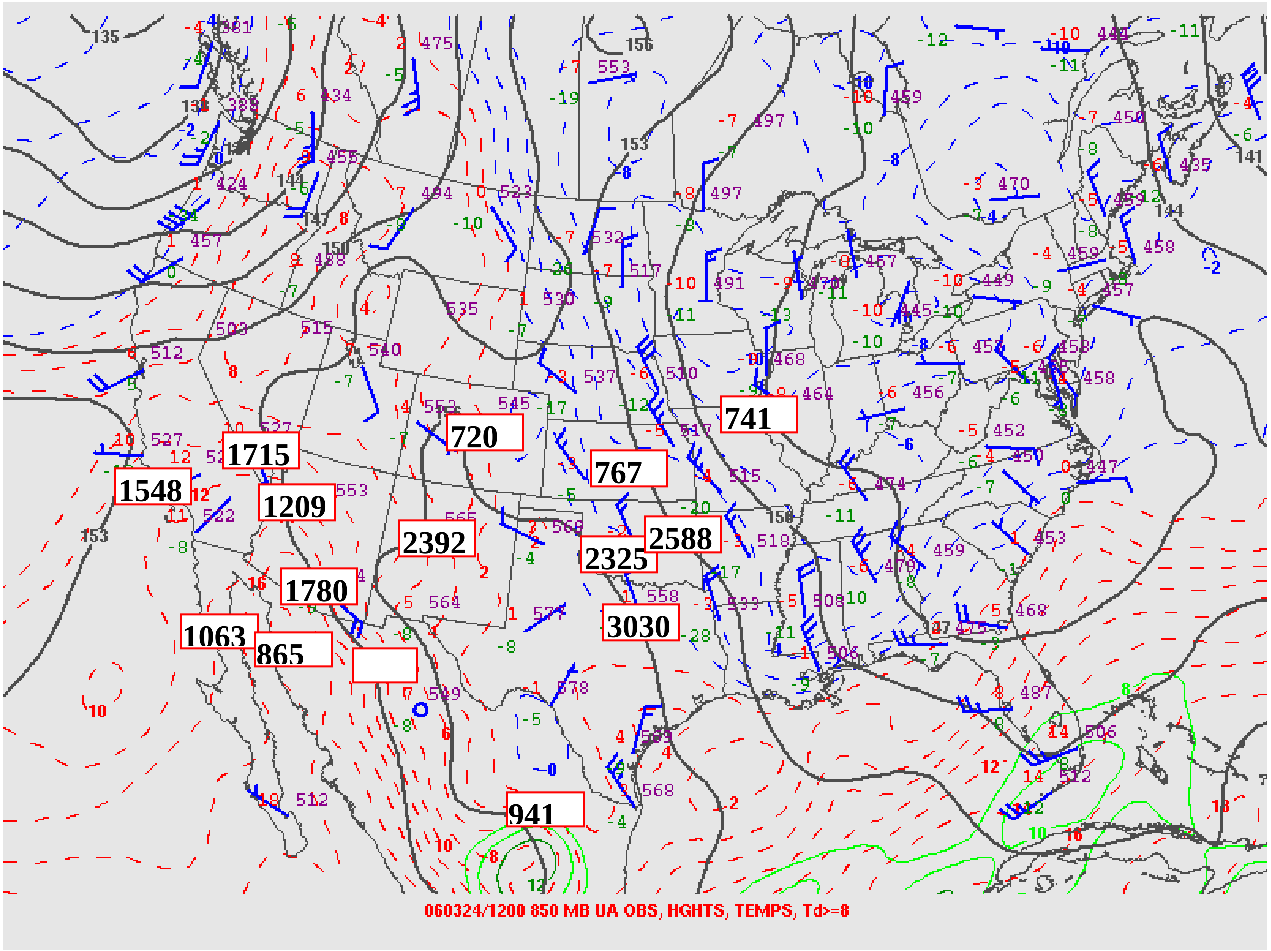

Figure F06-l-Used the 03/26/2006 week-ending Physician influenza-like illness report data, with the Mar 24 2006 12 z 850 mb chart. (Courtesy of NOAA NWS; Google Flu Trends).

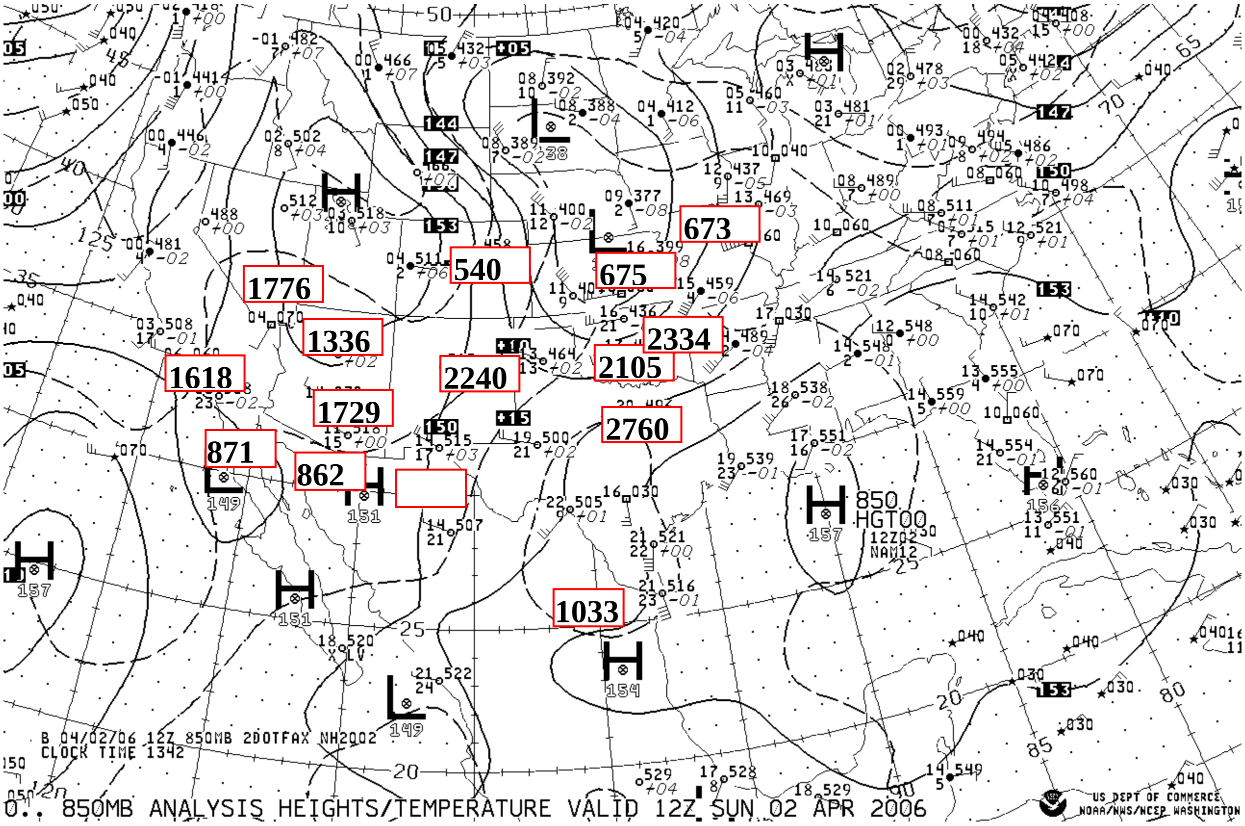

Figure F06-m-Used the 04/02/2006 week-ending Physician influenza-like illness report data, with the Apr 02 2006 12 z 850 mb chart. (Courtesy of NOAA NWS; Google Flu Trends).

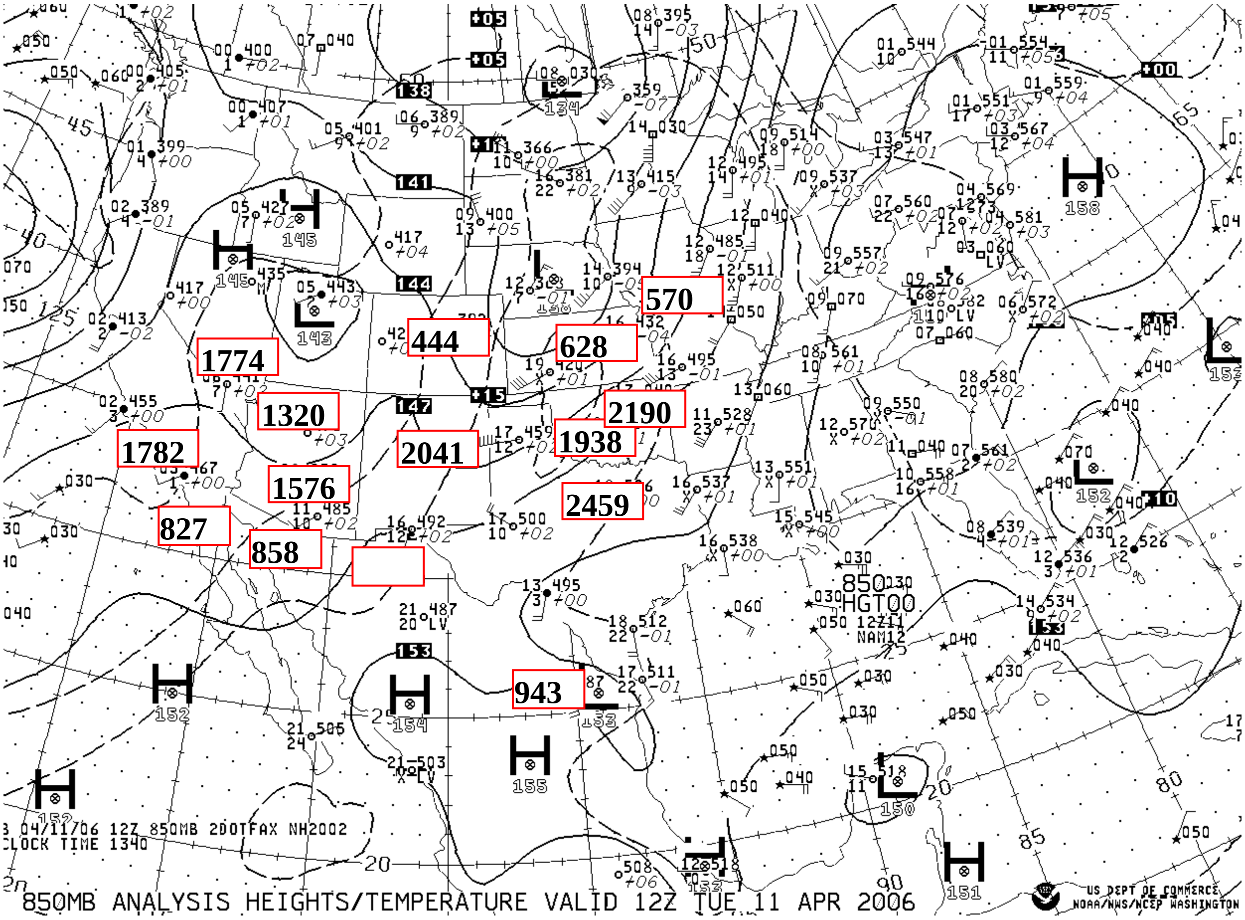

Figure F06-n-Used the 04/09/2006 week-ending Physician influenza-like illness report data, with the Apr 11 2006 12 z 850 mb chart. (Courtesy of NOAA NWS; Google Flu Trends).

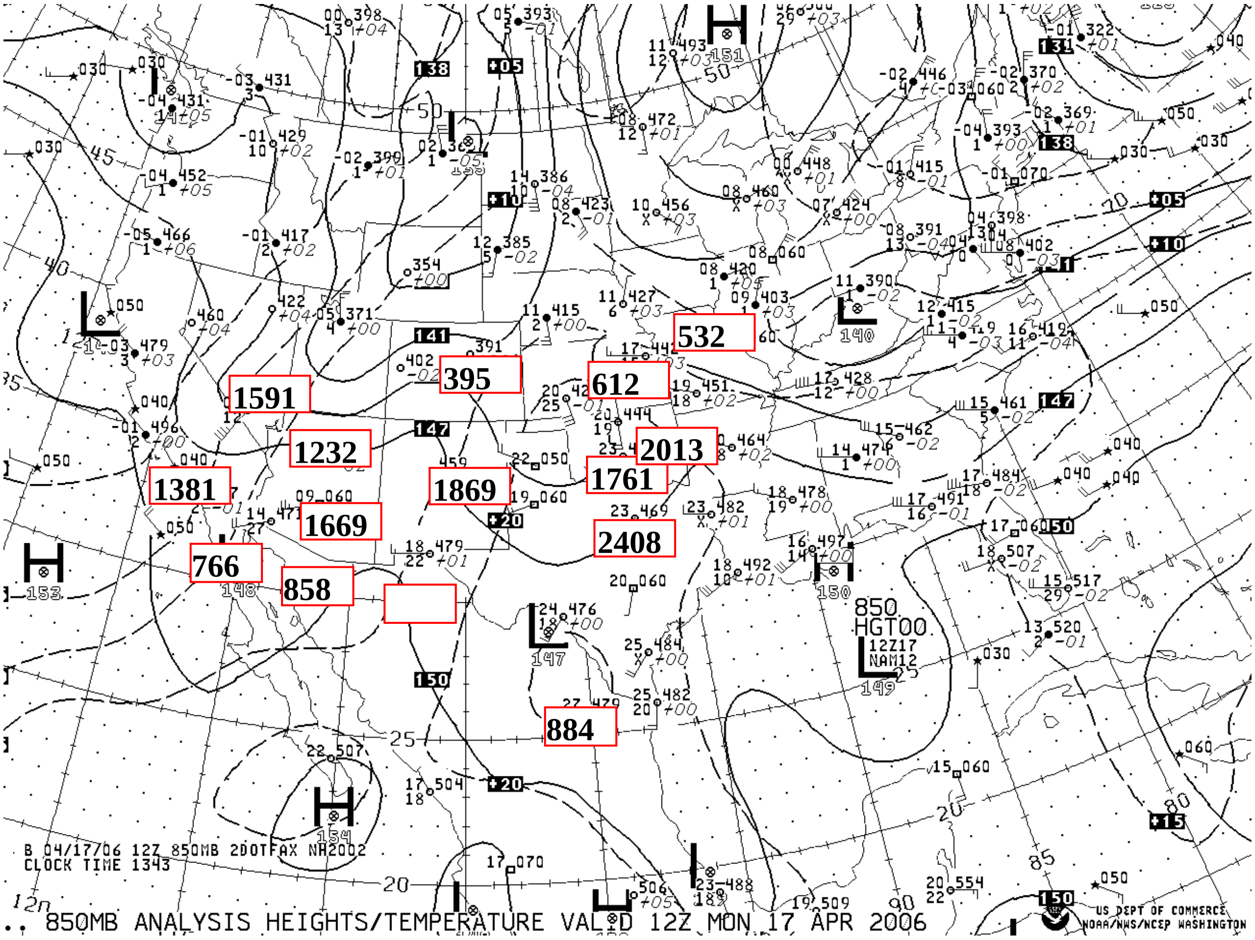

Figure F06-o-Used the 04/16/2006 week-ending Physician influenza-like illness report data, with the Apr 17 2006 12 z 850 mb chart. (Courtesy of NOAA NWS; Google Flu Trends).

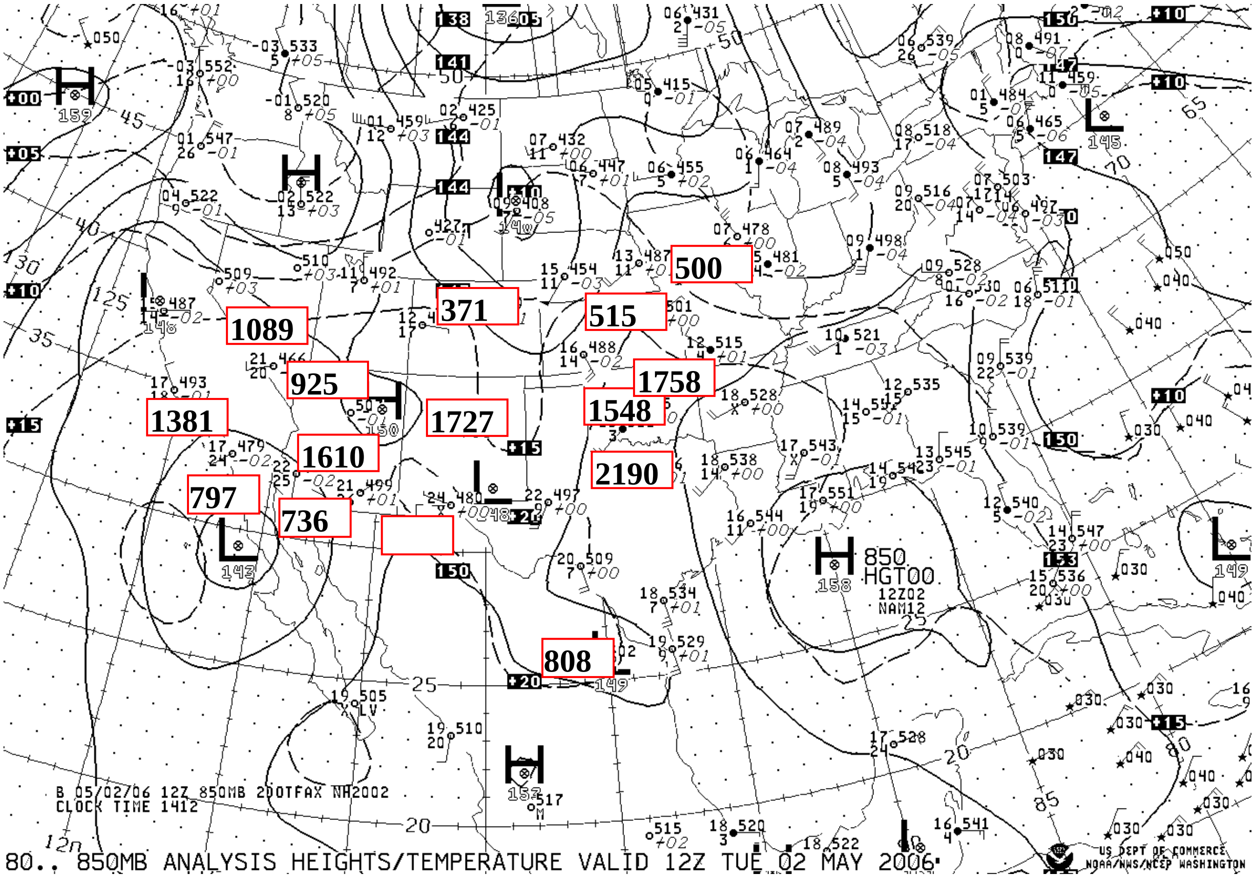

Figure F06-p-Used the 04/30/2006 week-ending Physician influenza-like illness report data, with the May 02 2006 12 z 850 mb chart. (Courtesy of NOAA NWS; Google Flu Trends).

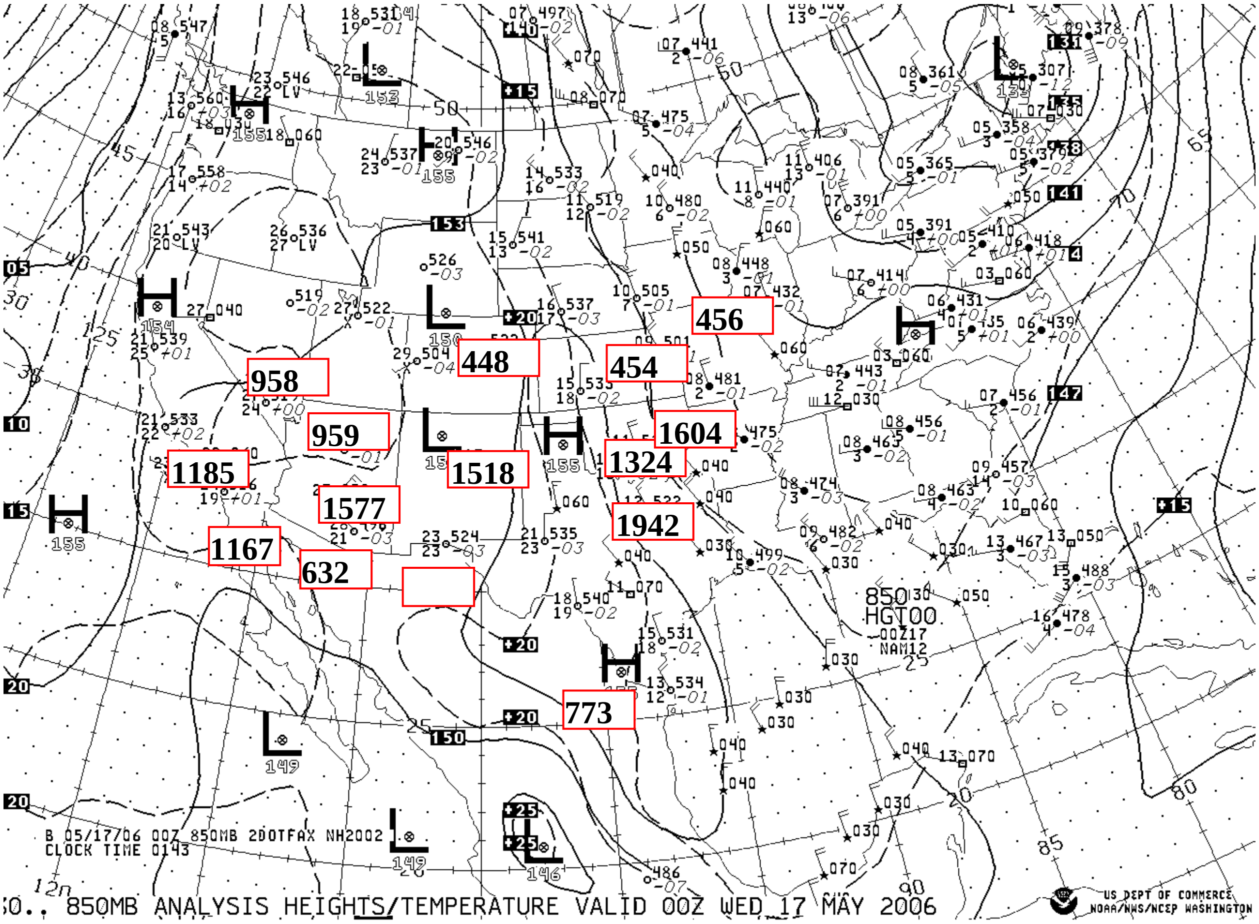

Figure F06-q-Used the 05/14/2006 week-ending Physician influenza-like illness report data, with the May 16 2006 12 z 850 mb chart. (Courtesy of NOAA NWS; Google Flu Trends).

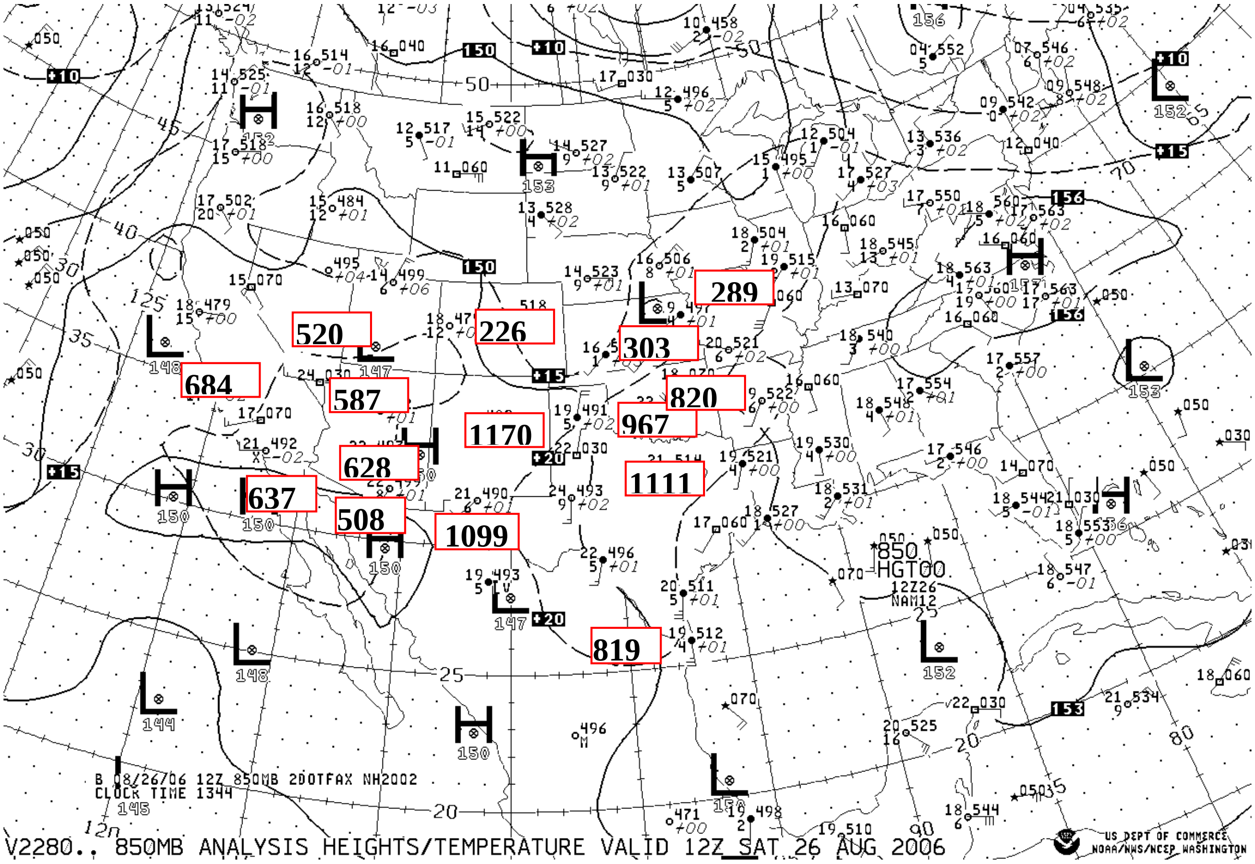

Figure 06G-a-Used the 08/27/2006 week-ending Physician influenza-like illness report data, with the Aug 26 2006 12 z 850 mb chart. (Courtesy of NOAA NWS; Google Flu Trends).

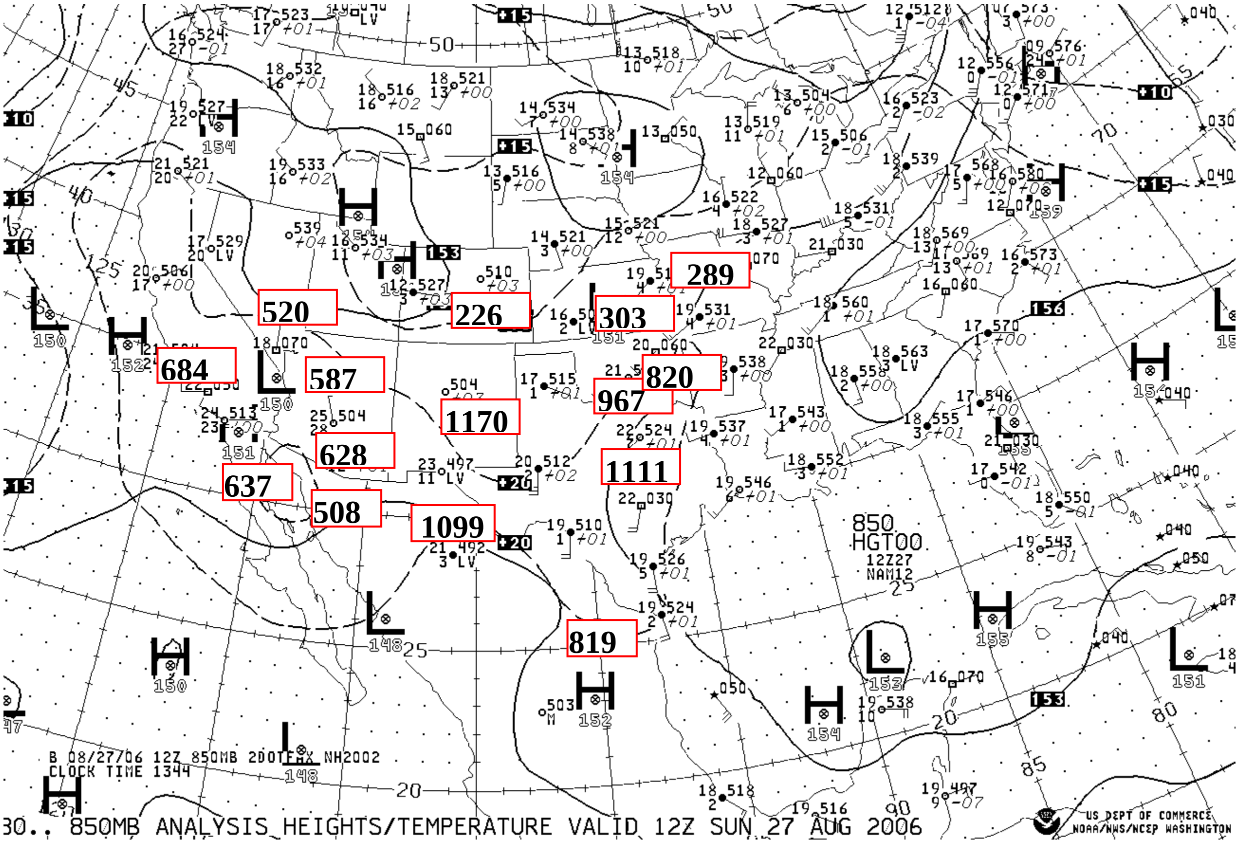

Figure 06G-b-Used the 08/27/2006 week-ending Physician influenza-like illness report data, with the Aug 27 2006 12 z 850 mb chart. (Courtesy of NOAA NWS; Google Flu Trends).

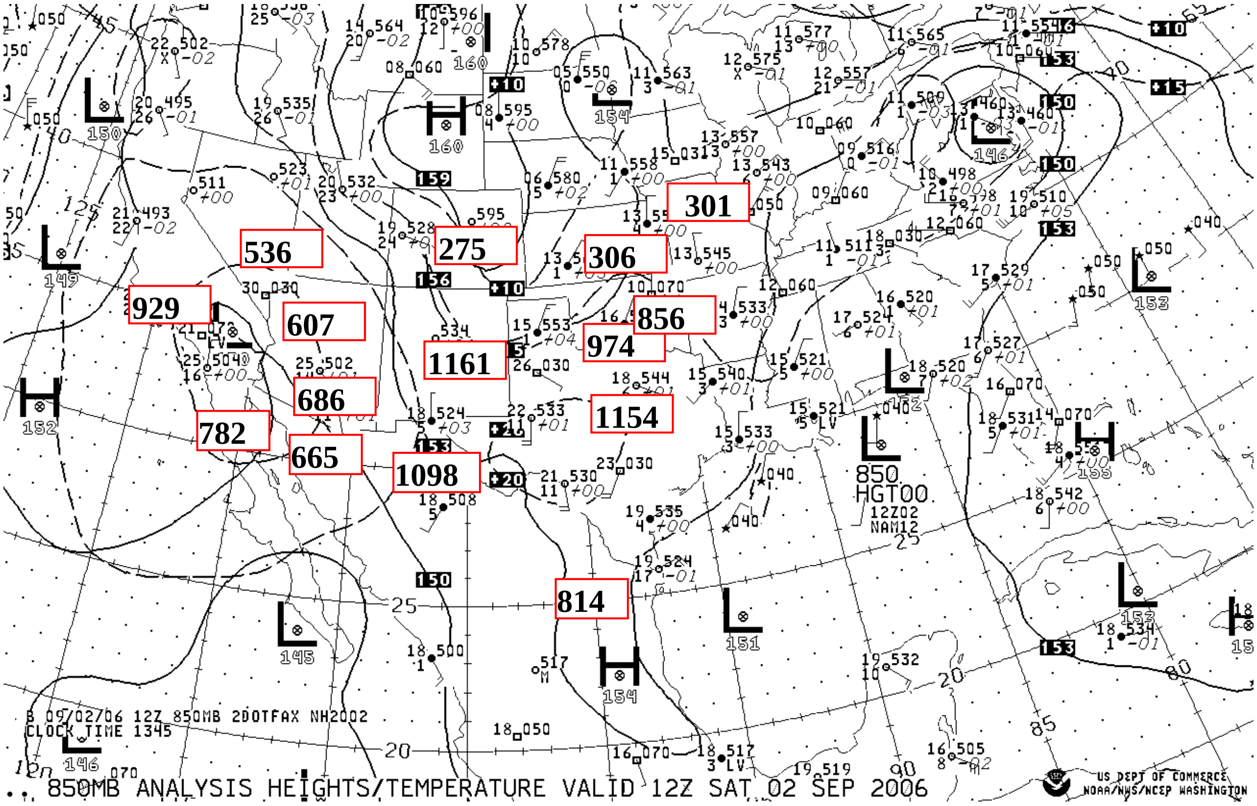

Figure 06G-c-Used the 09/03/2006 week-ending Physician influenza-like illness report data, with the Sep 02 2006 12 z 850 mb chart. (Courtesy of NOAA NWS; Google Flu Trends).

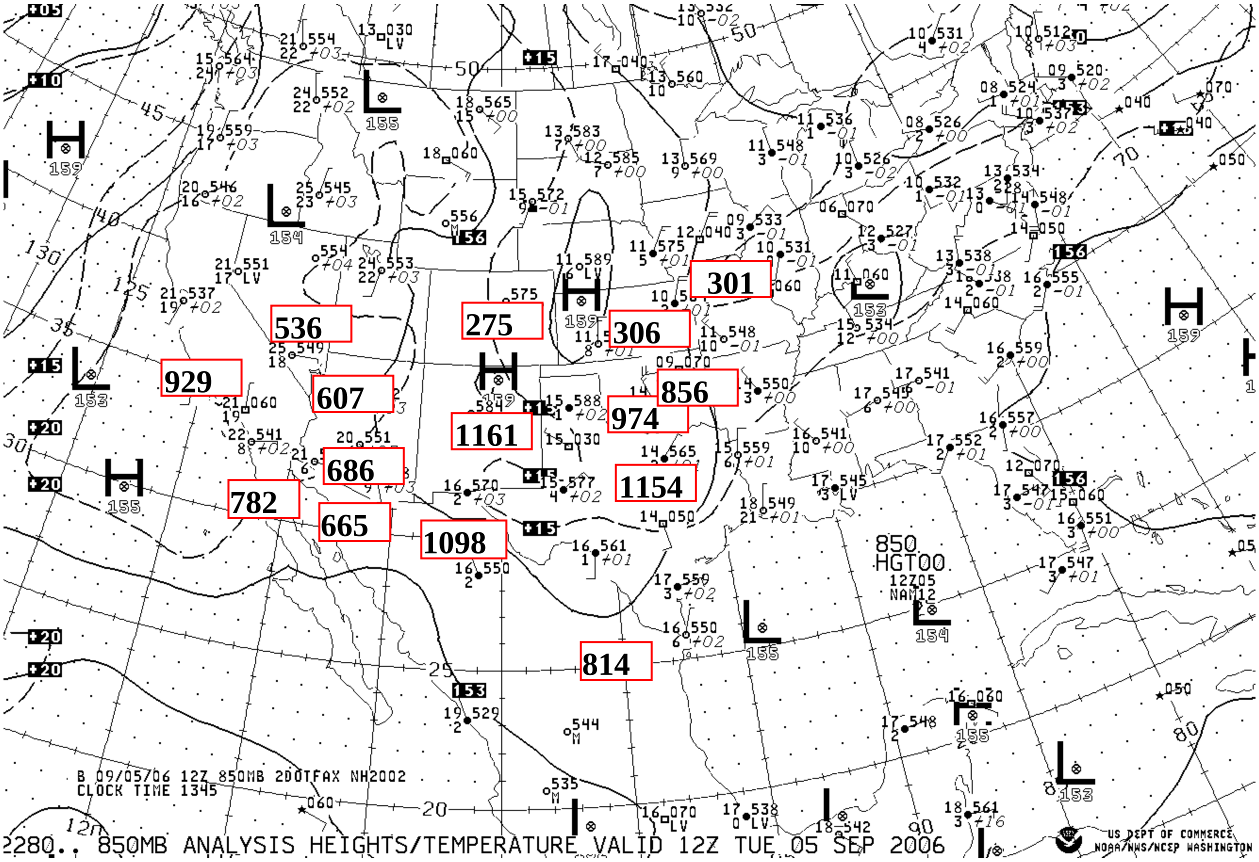

Figure 06G-d-Used the 09/03/2006 week-ending Physician influenza-like illness report data, with the Sep 05 2006 12 z 850 mb chart. (Courtesy of NOAA NWS; Google Flu Trends).

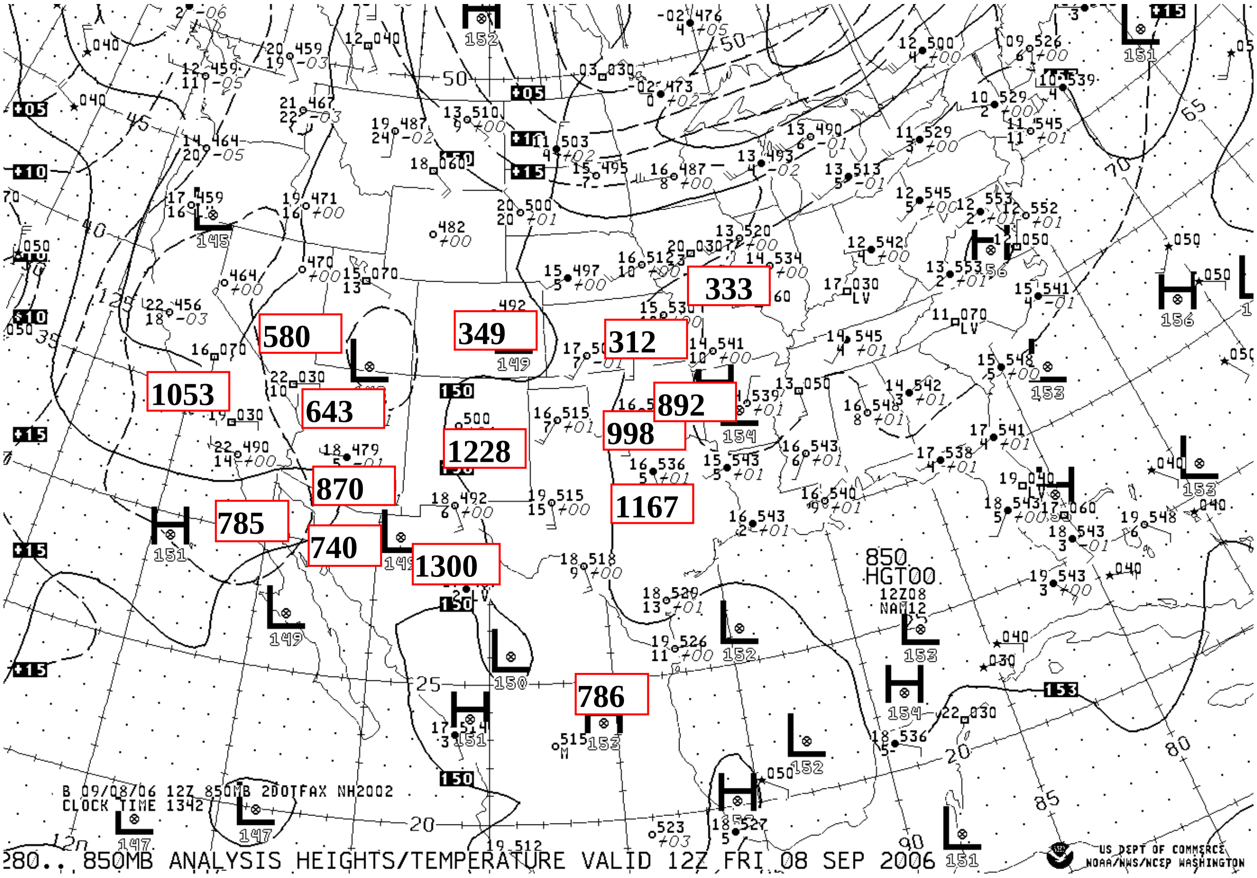

Figure 06G-e-Used the 09/10/2006 week-ending Physician influenza-like illness report data, with the Sep 08 2006 12 z 850 mb chart. (Courtesy of NOAA NWS; Google Flu Trends).

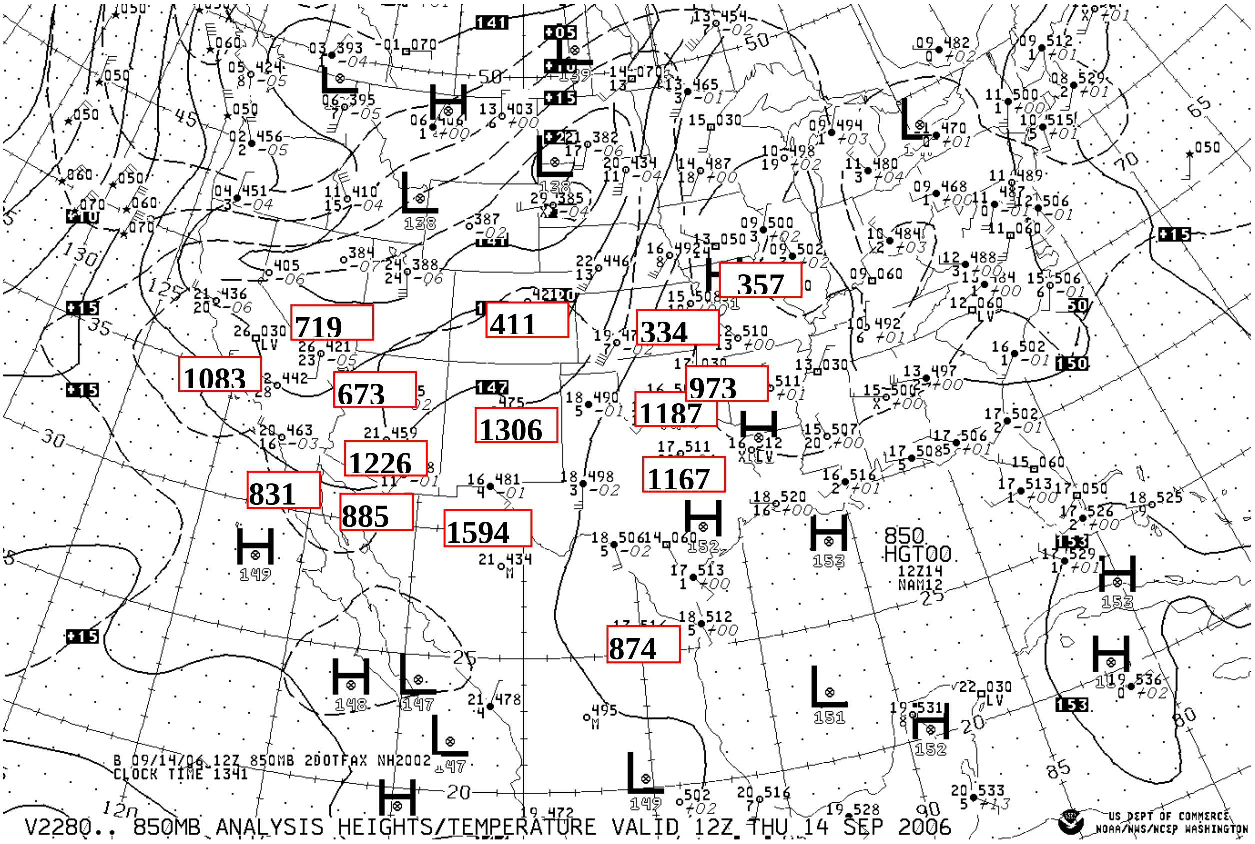

Figure 06G-f-Used the 09/17/2006 week-ending Physician influenza-like illness report data, with the Sep 14 2006 12 z 850 mb chart. (Courtesy of NOAA NWS; Google Flu Trends).

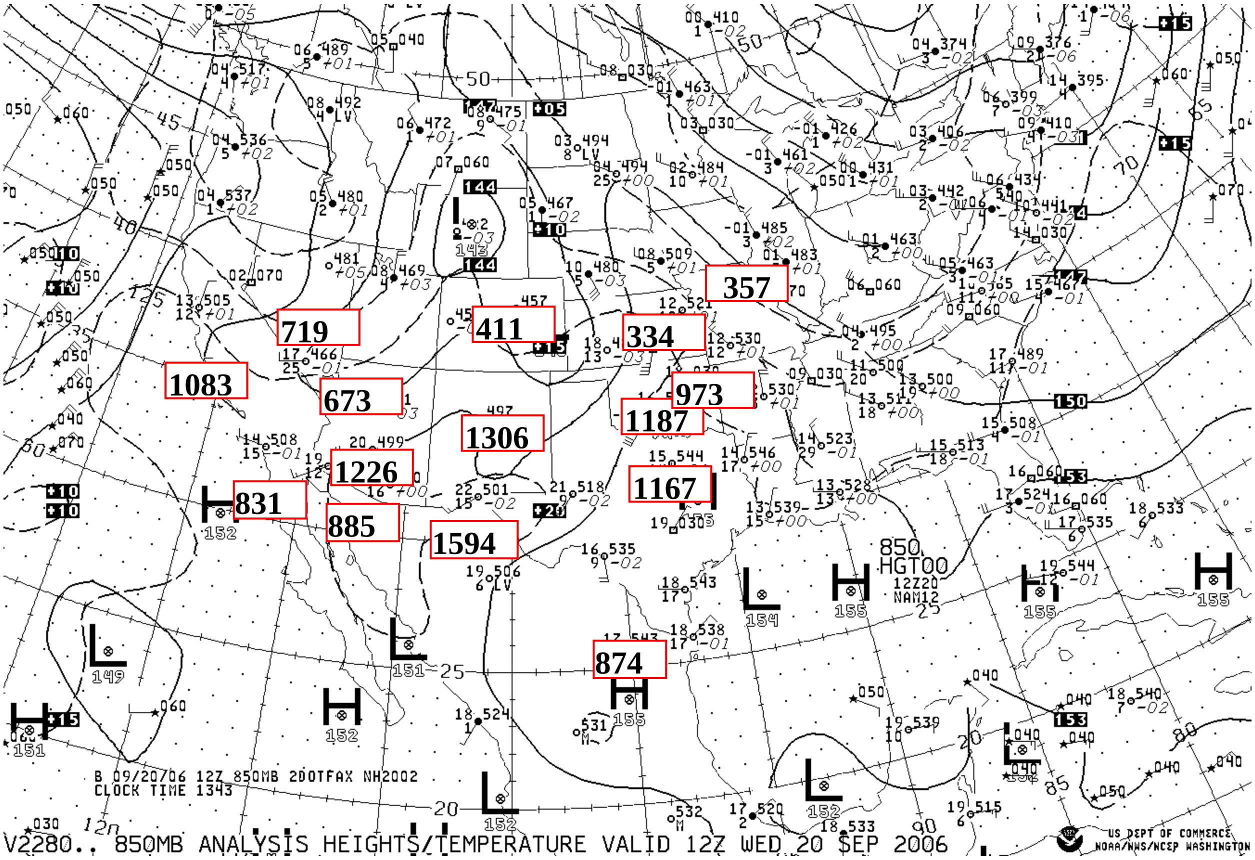

Figure 06G-g-Used the 09/17/2006 week-ending Physician influenza-like illness report data, with the Sep 20 2006 12 z 850 mb chart. (Courtesy of NOAA NWS; Google Flu Trends).

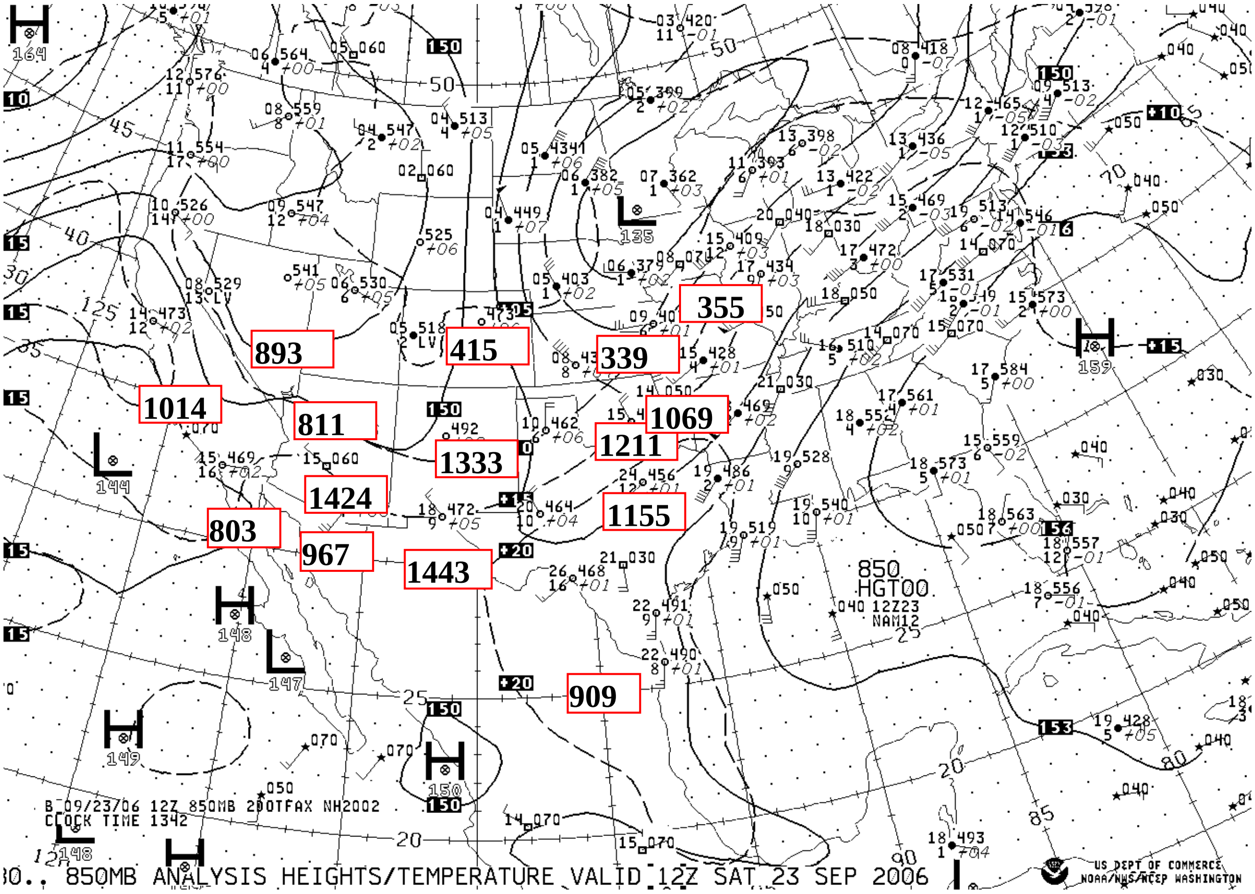

Figure 06G-h-Used the 09/24/2006 week-ending Physician influenza-like illness report data, with the Sep 23 2006 12 z 850 mb chart. (Courtesy of NOAA NWS; Google Flu Trends).

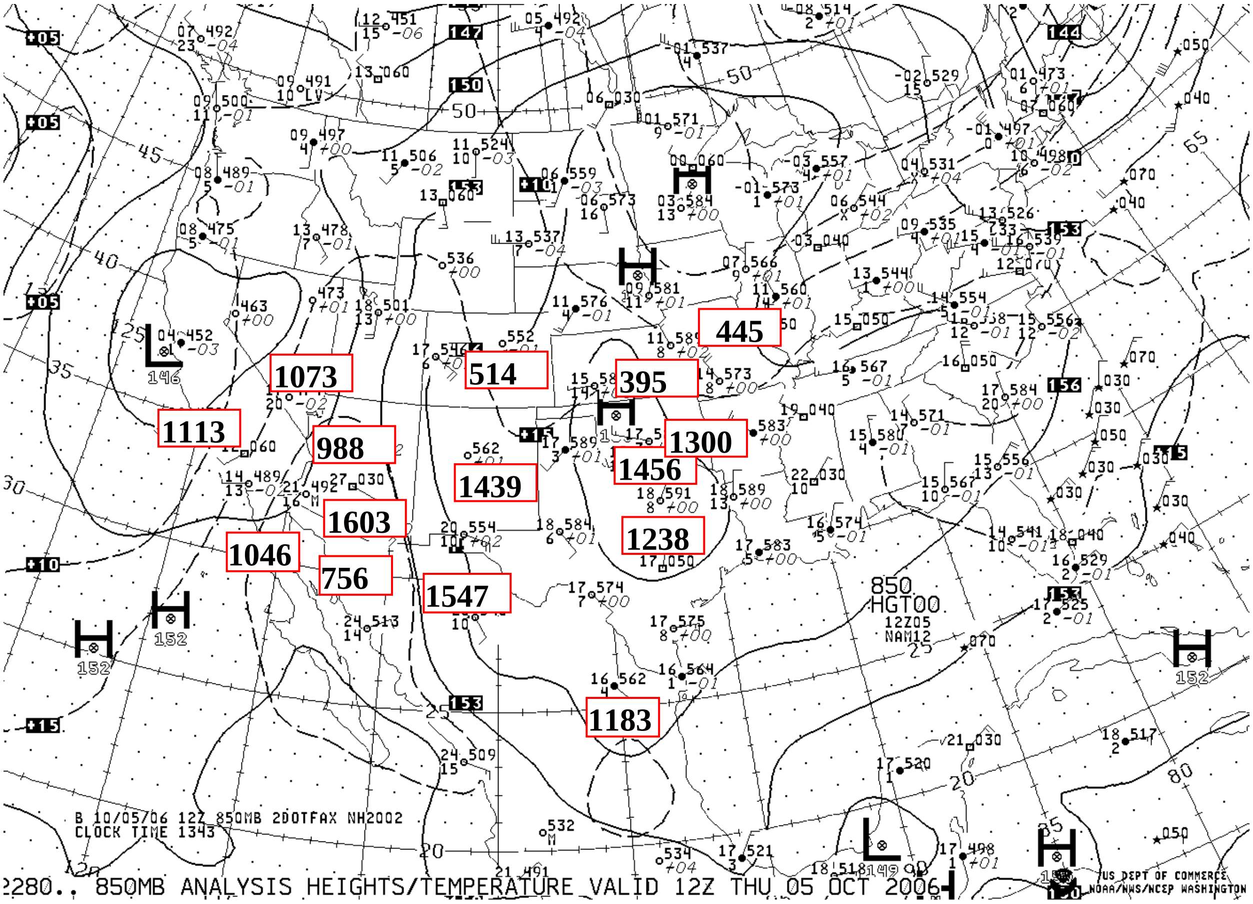

Figure 06G-i1-Used the 10/08/2006 week-ending Physician influenza-like illness report data, with the Oct 05 2006 12 z 850 mb chart. (Courtesy of NOAA NWS; Google Flu Trends).

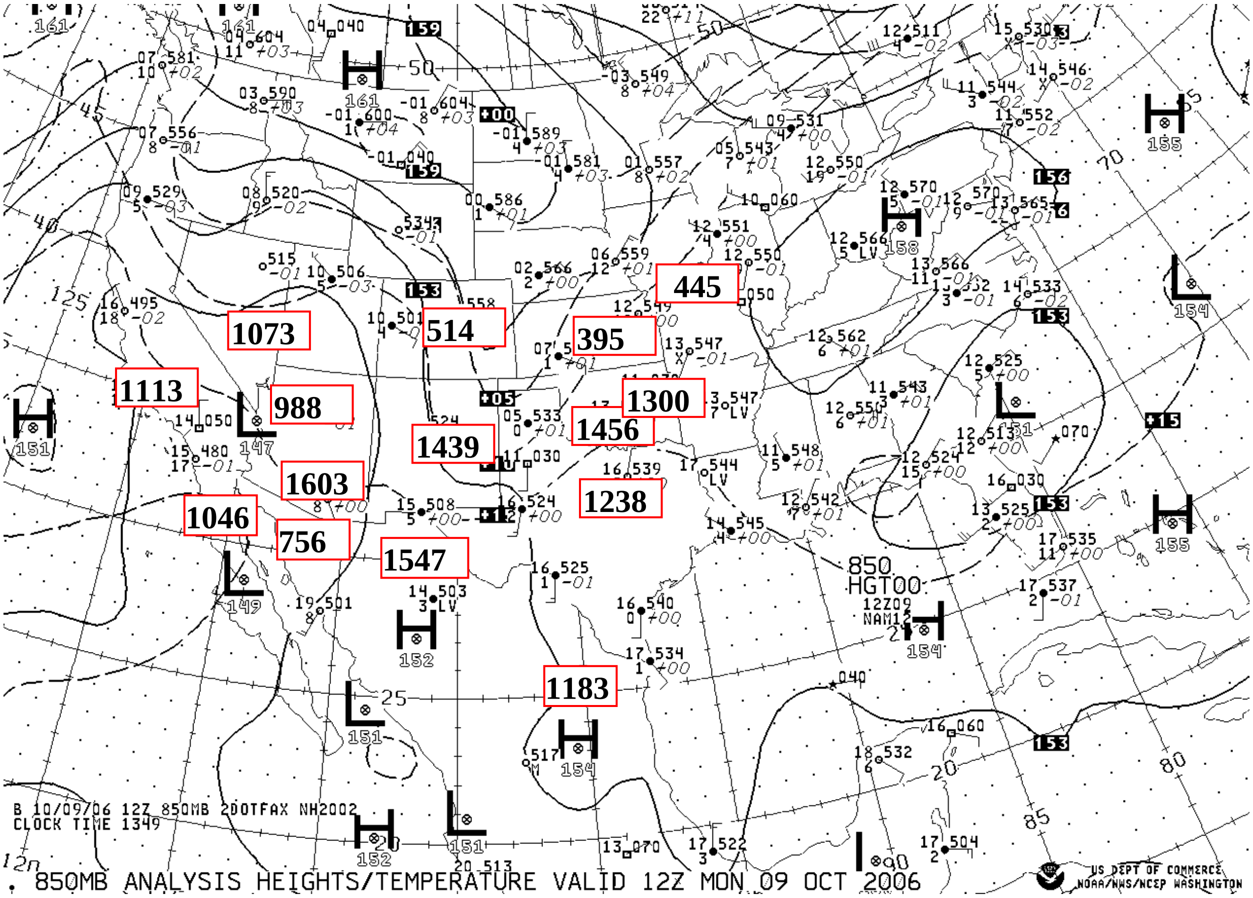

Figure 06G-i2-Used the 10/08/2006 week-ending Physician influenza-like illness report data, with the Oct 09 2006 12 z 850 mb chart. (Courtesy of NOAA NWS; Google Flu Trends).

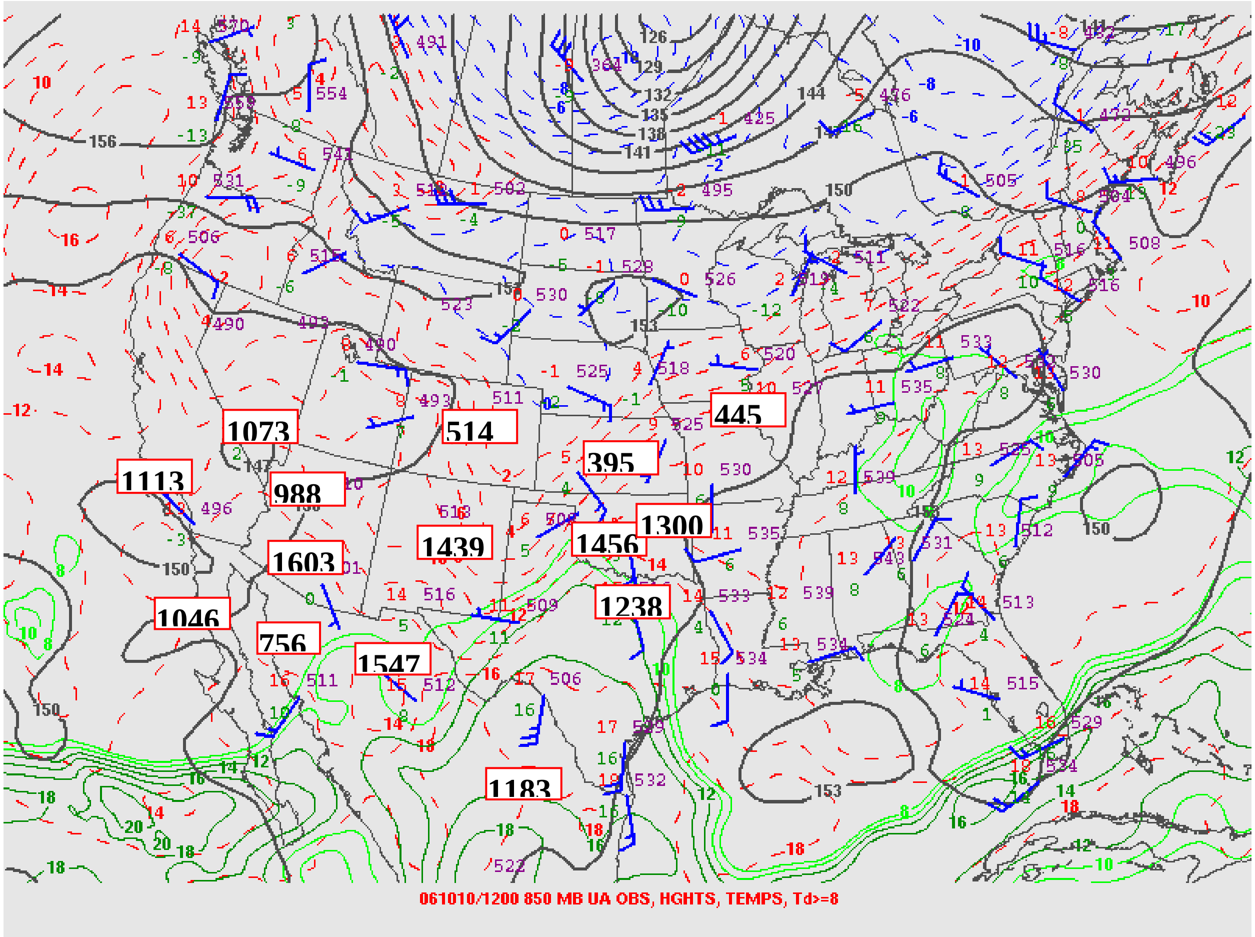

Figure 06G-i3-Used the 10/08/2006 week-ending Physician influenza-like illness report data, with the Oct 10 2006 12 z 850 mb chart. (Courtesy of NOAA NWS; Google Flu Trends).

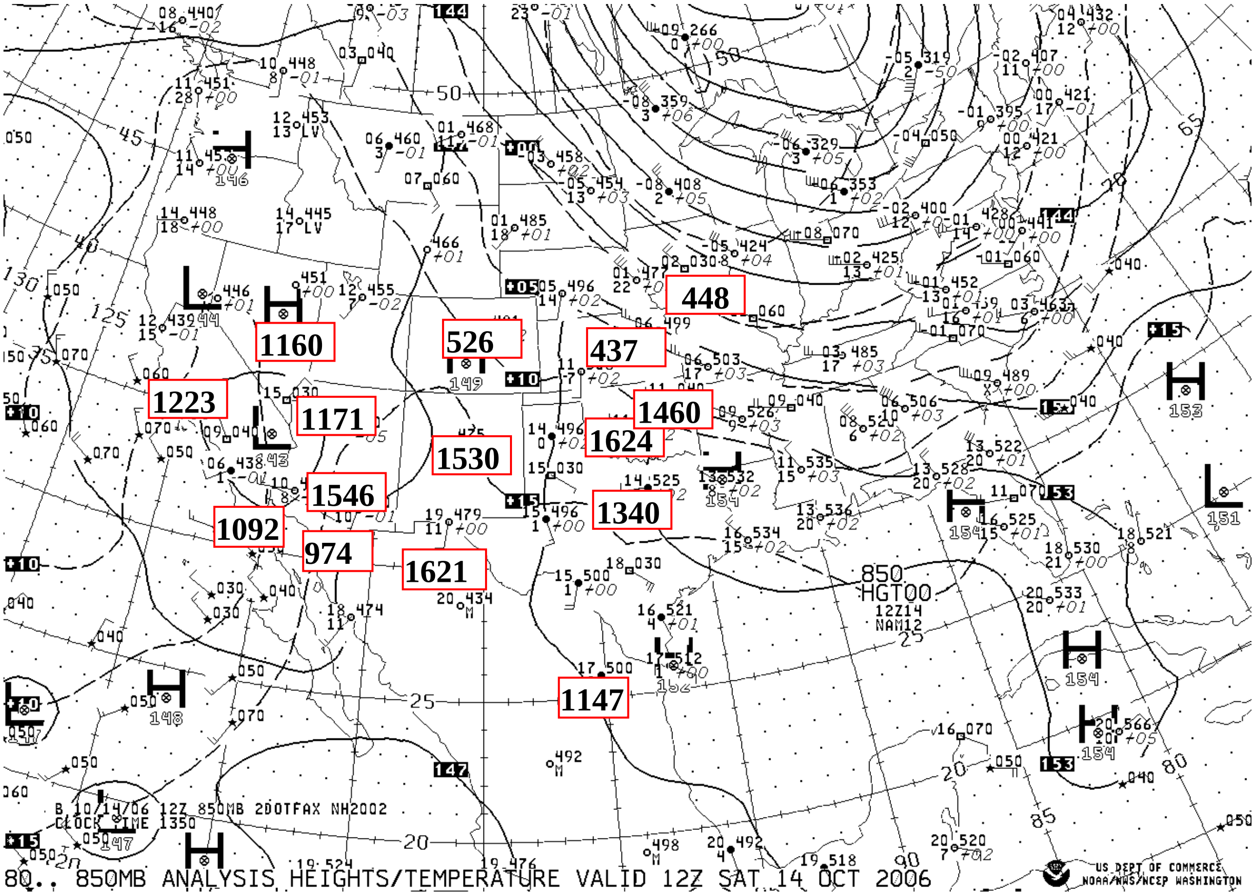

Figure 06G-j-Used the 10/15/2006 week-ending Physician influenza-like illness report data, with the Oct 14 2006 12 z 850 mb chart. (Courtesy of NOAA NWS; Google Flu Trends).

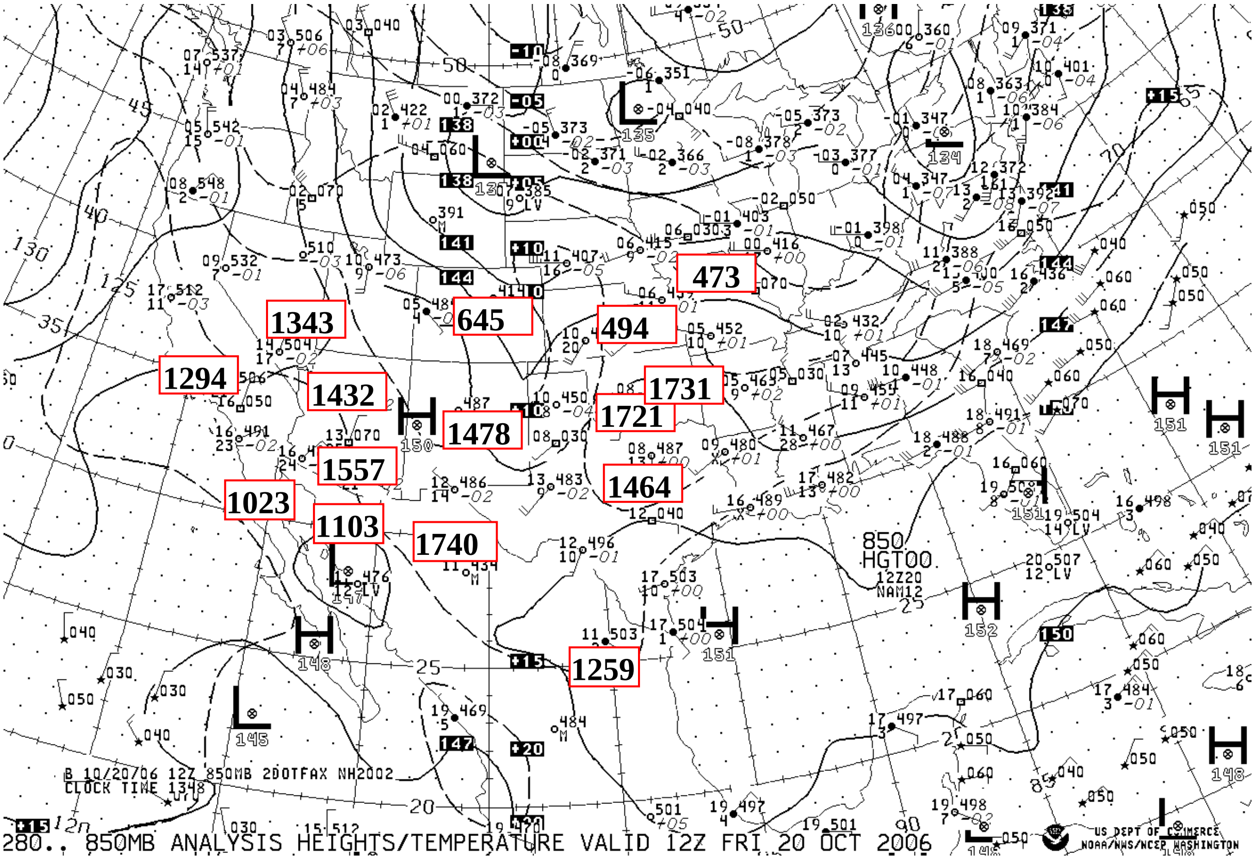

Figure 06G-k-Used the 10/22/2006 week-ending Physician influenza-like illness report data, with the Oct 20 2006 12 z 850 mb chart. (Courtesy of NOAA NWS; Google Flu Trends).

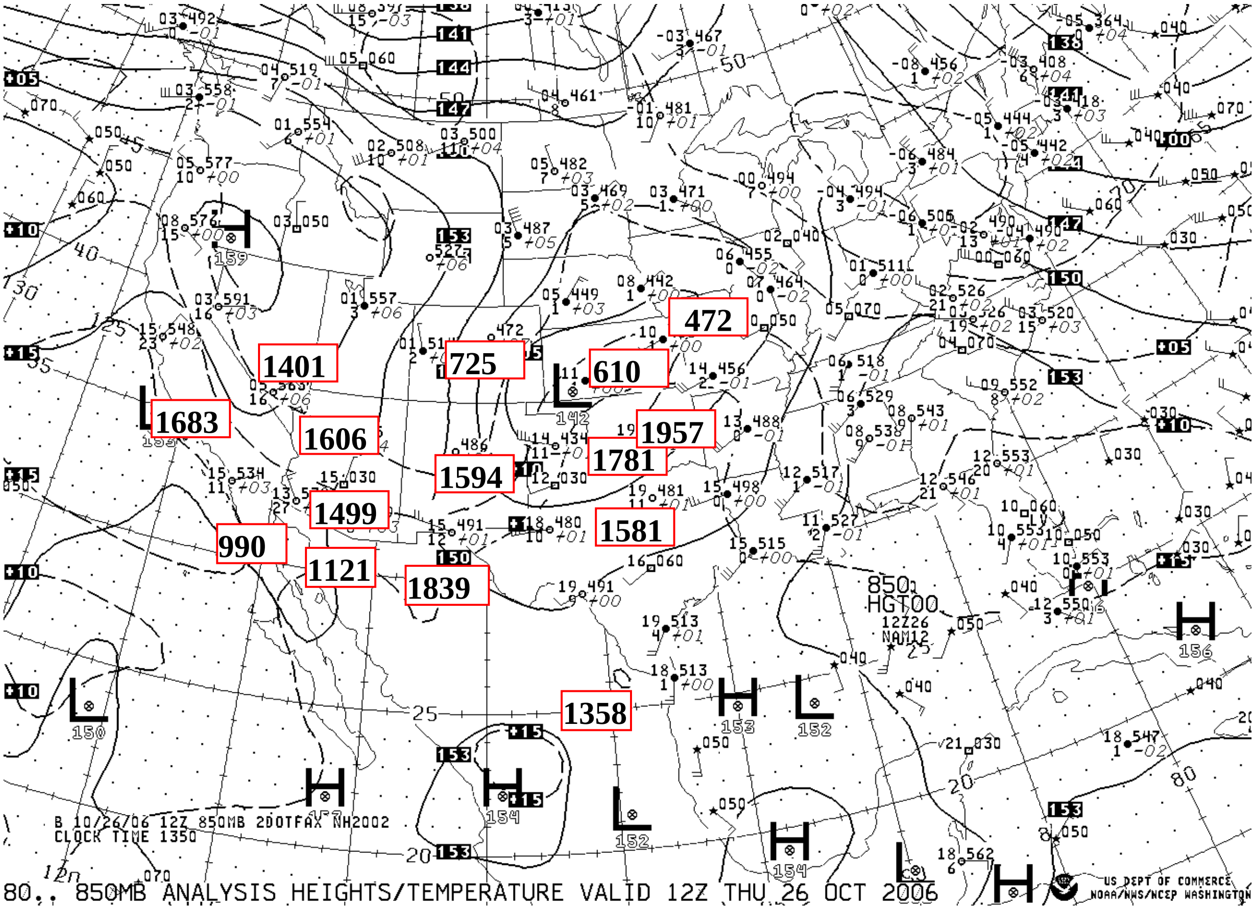

Figure 06G-l1-Used the 10/29/2006 week-ending Physician influenza-like illness report data, with the Oct 26 2006 12 z 850 mb chart. (Courtesy of NOAA NWS; Google Flu Trends).

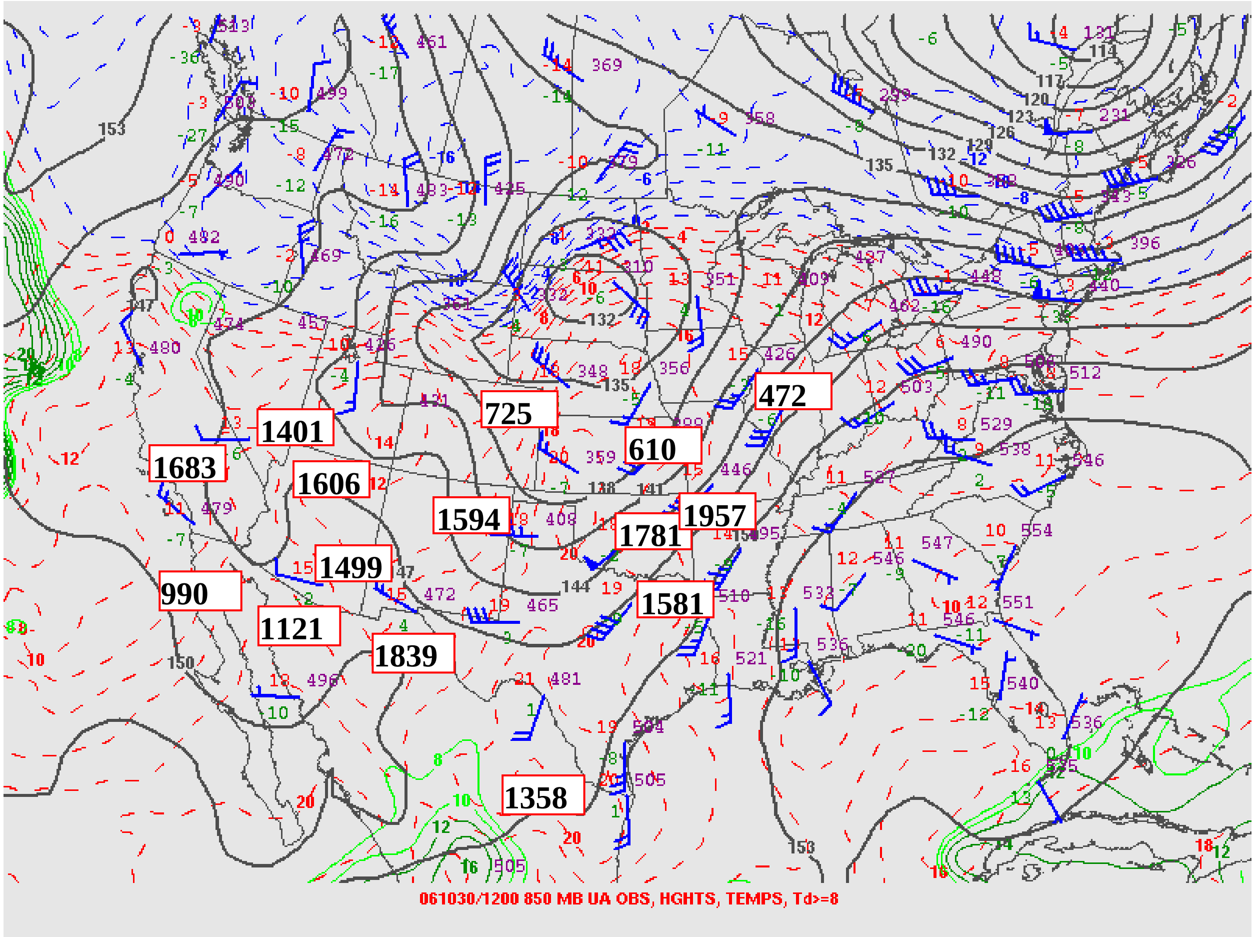

Figure 06G-l2-Used the 10/29/2006 week-ending Physician influenza-like illness report data, with the Oct 30 2006 12 z 850 mb chart. (Courtesy of NOAA NWS; Google Flu Trends).

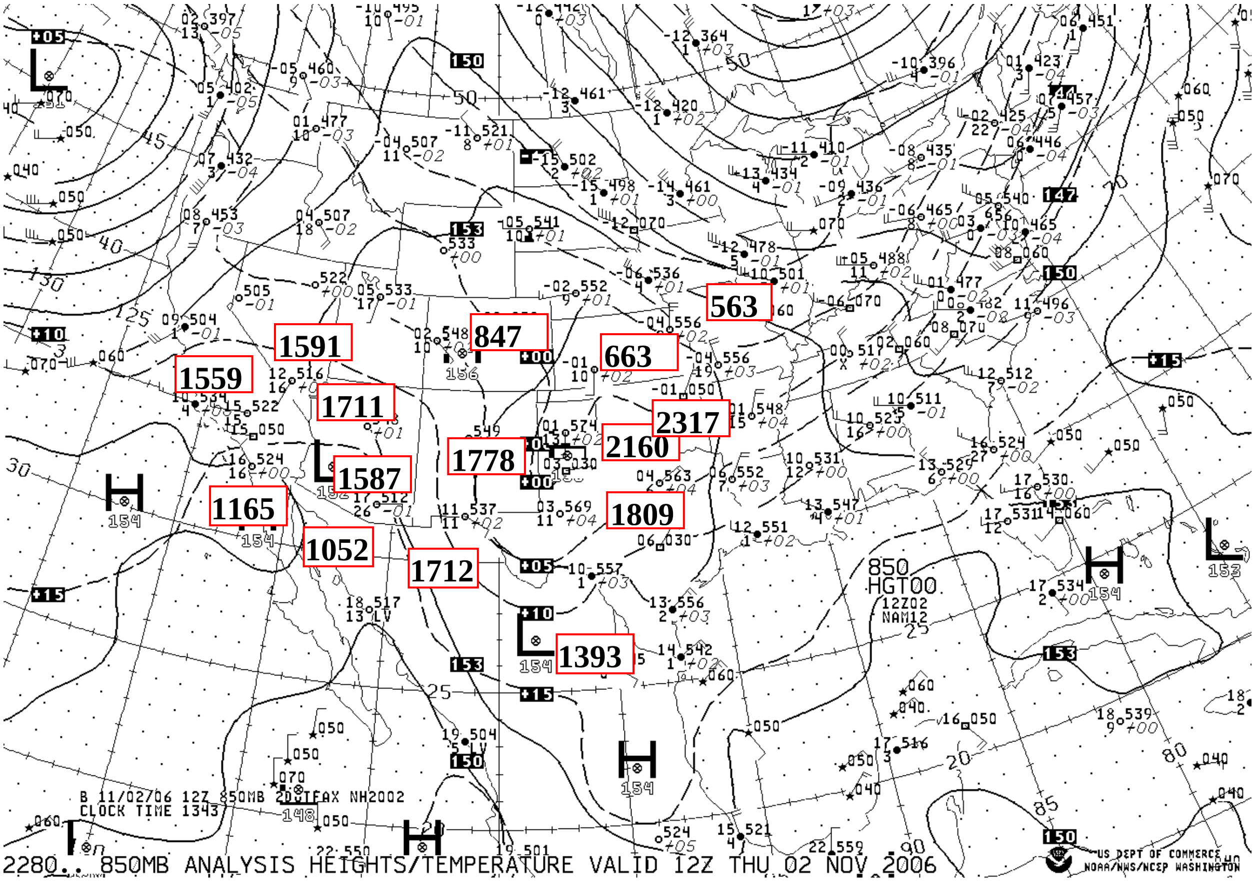

Figure 06G-m-Used the 11/05/2006 week-ending Physician influenza-like illness report data, with the Nov 02 2006 12 z 850 mb chart. (Courtesy of NOAA NWS; Google Flu Trends).

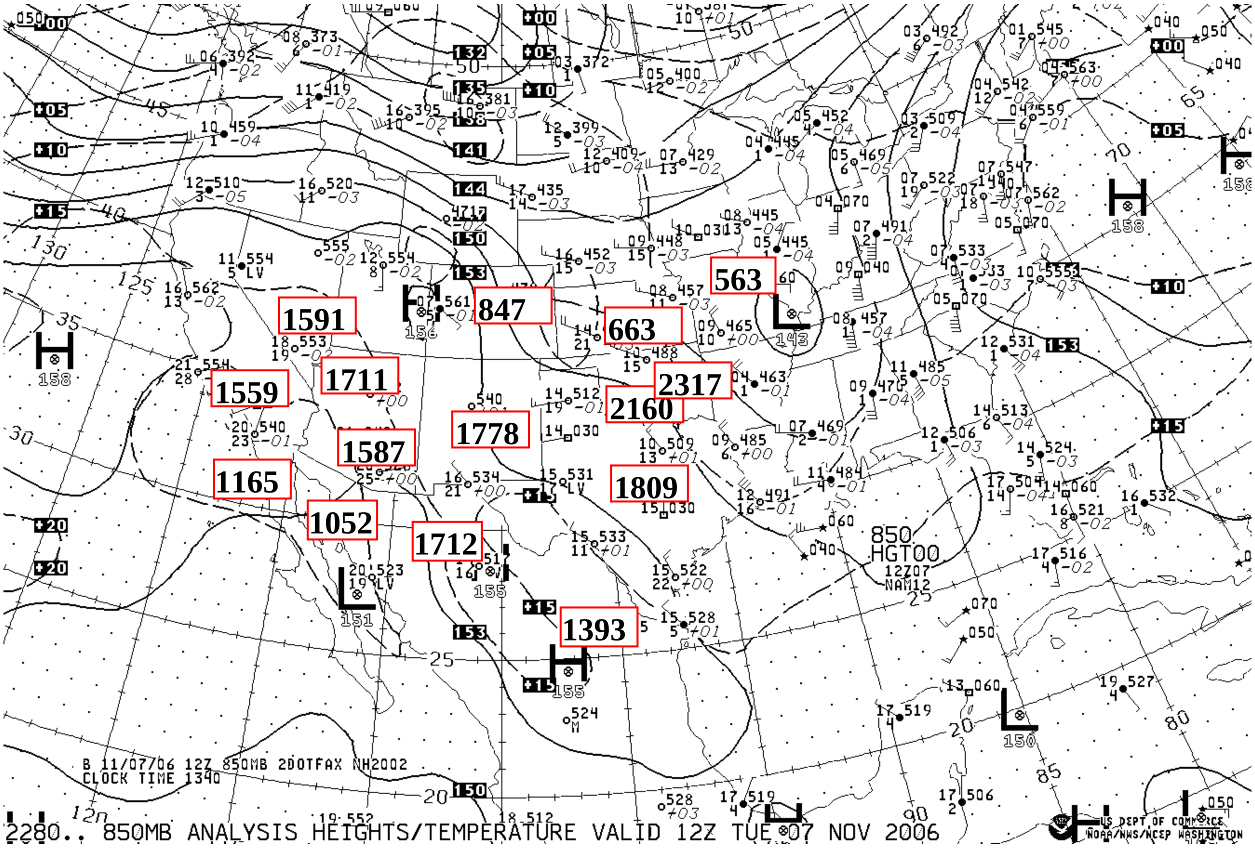

Figure 06G-n-Used the 11/05/2006 week-ending Physician influenza-like illness report data, with the Nov 07 2006 12 z 850 mb chart. (Courtesy of NOAA NWS; Google Flu Trends).

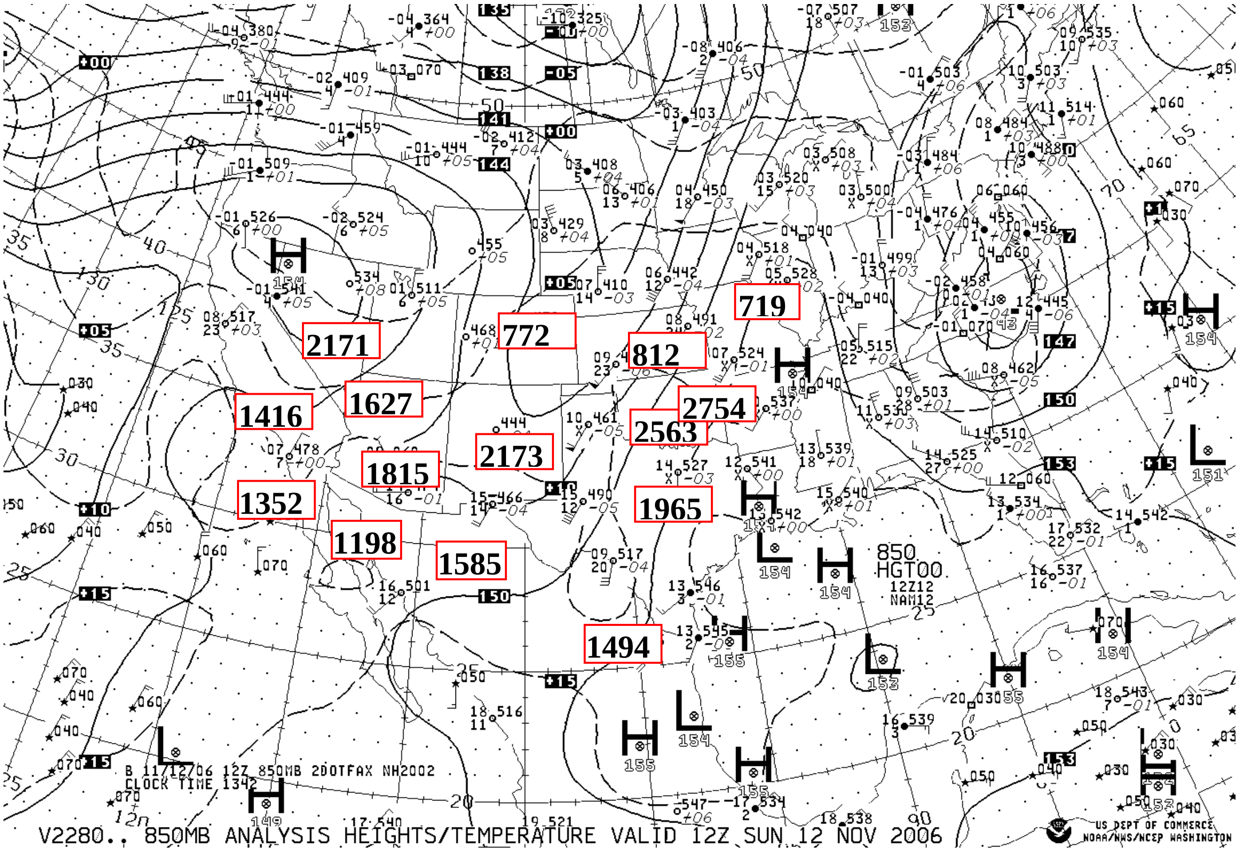

Figure 06G-o-Used the 11/12/2006 week-ending Physician influenza-like illness report data, with the Nov 12 2006 12 z 850 mb chart. (Courtesy of NOAA NWS; Google Flu Trends).

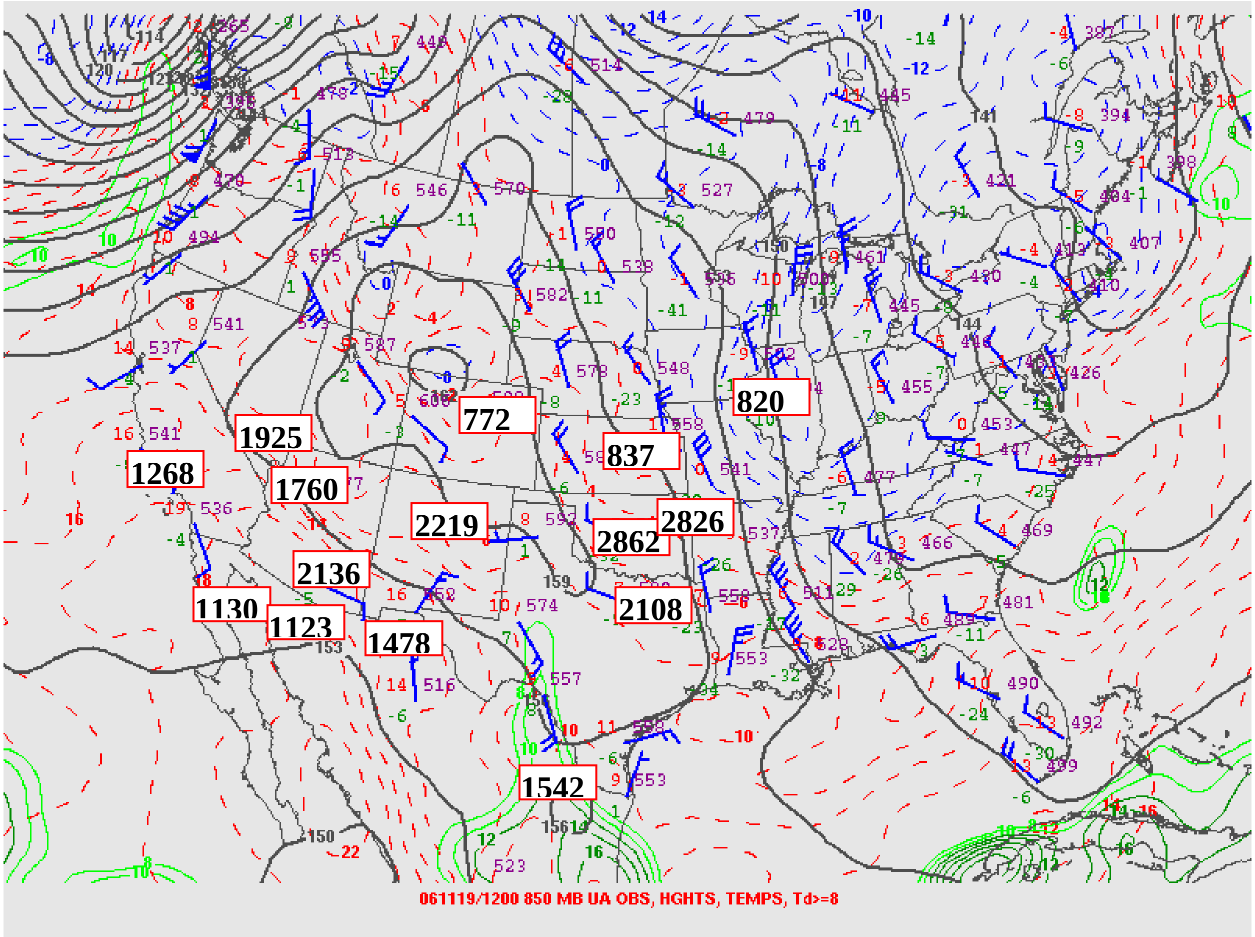

Figure 06G-p-Used the 11/19/2006 week-ending Physician influenza-like illness report data, with the Nov 19 2006 12 z 850 mb chart. (Courtesy of NOAA NWS; Google Flu Trends).

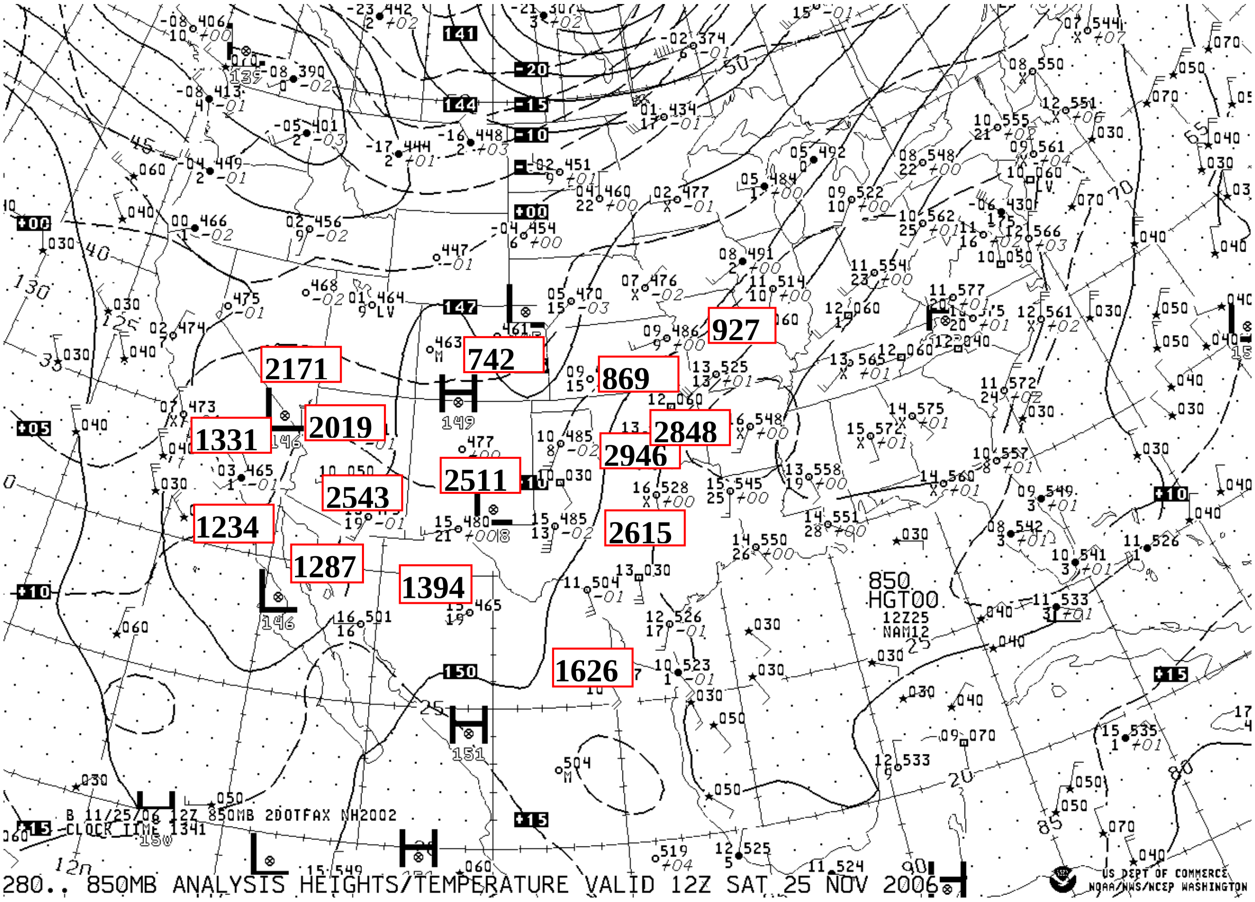

Figure 06G-q1-Used the 11/26/2006 week-ending Physician influenza-like illness report data, with the Nov 25 2006 12 z 850 mb chart. (Courtesy of NOAA NWS; Google Flu Trends).

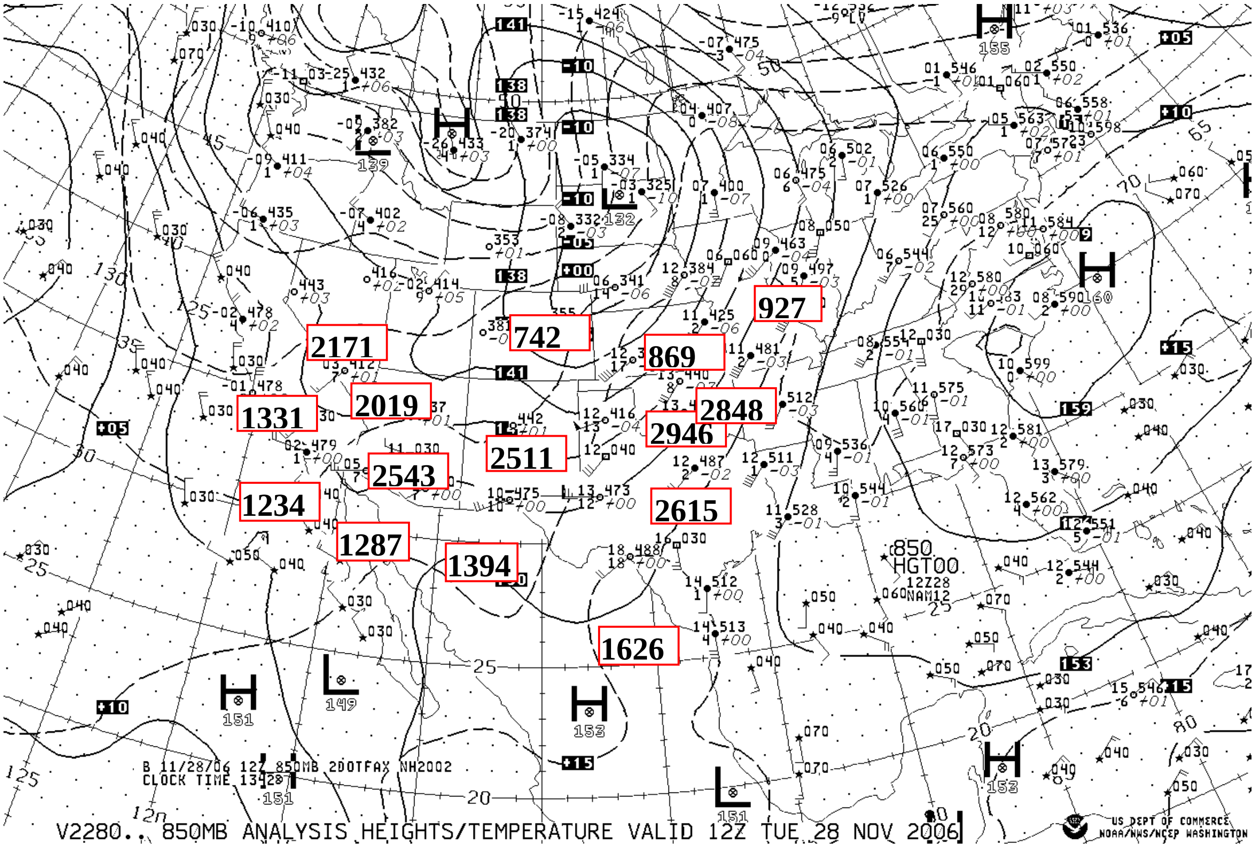

Figure 06G-q2-Used the 11/26/2006 week-ending Physician influenza-like illness report data, with the Nov 28 2006 12 z 850 mb chart. (Courtesy of NOAA NWS; Google Flu Trends).

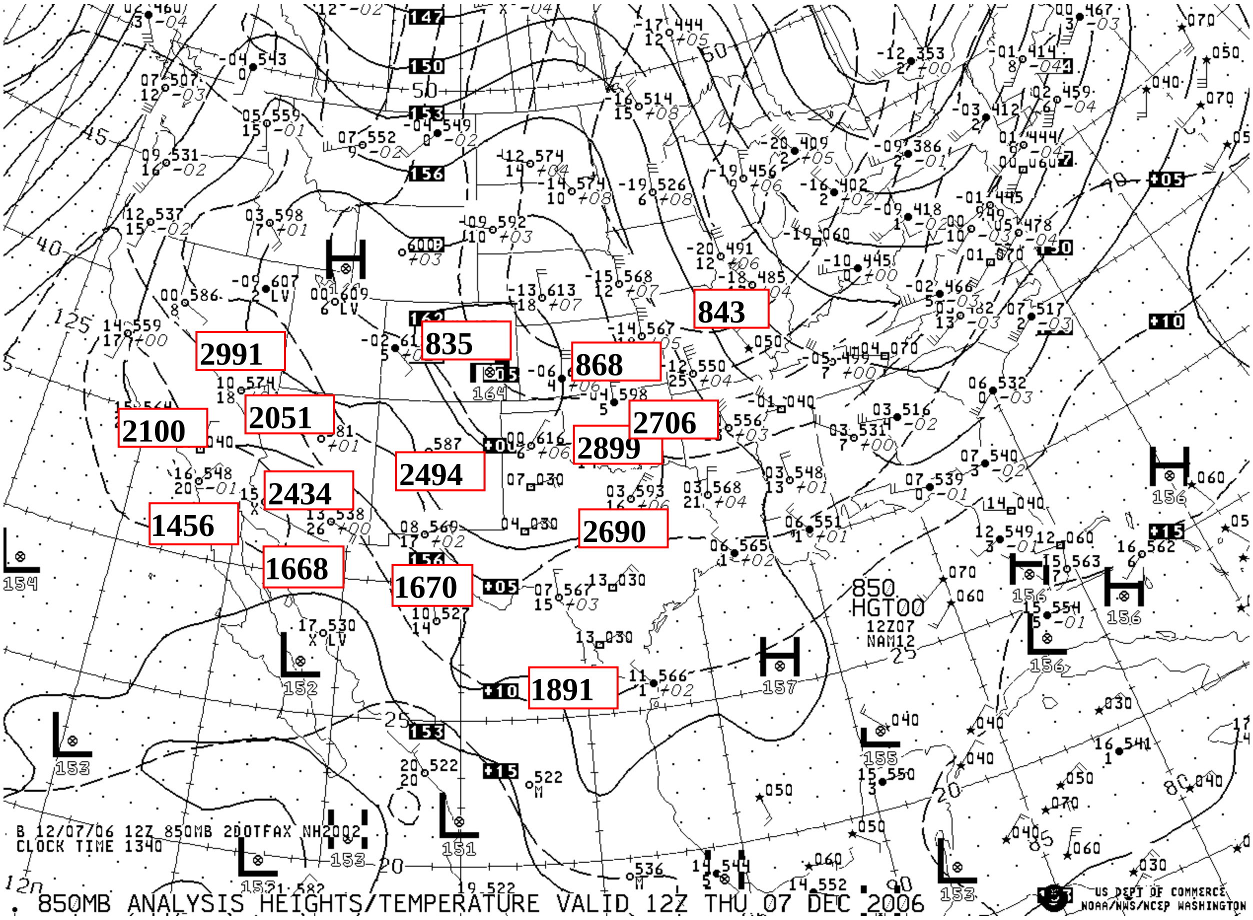

Figure 06G-r1-Used the 12/10/2006 week-ending Physician influenza-like illness report data, with the Dec 07 2006 12 z 850 mb chart. (Courtesy of NOAA NWS; Google Flu Trends).

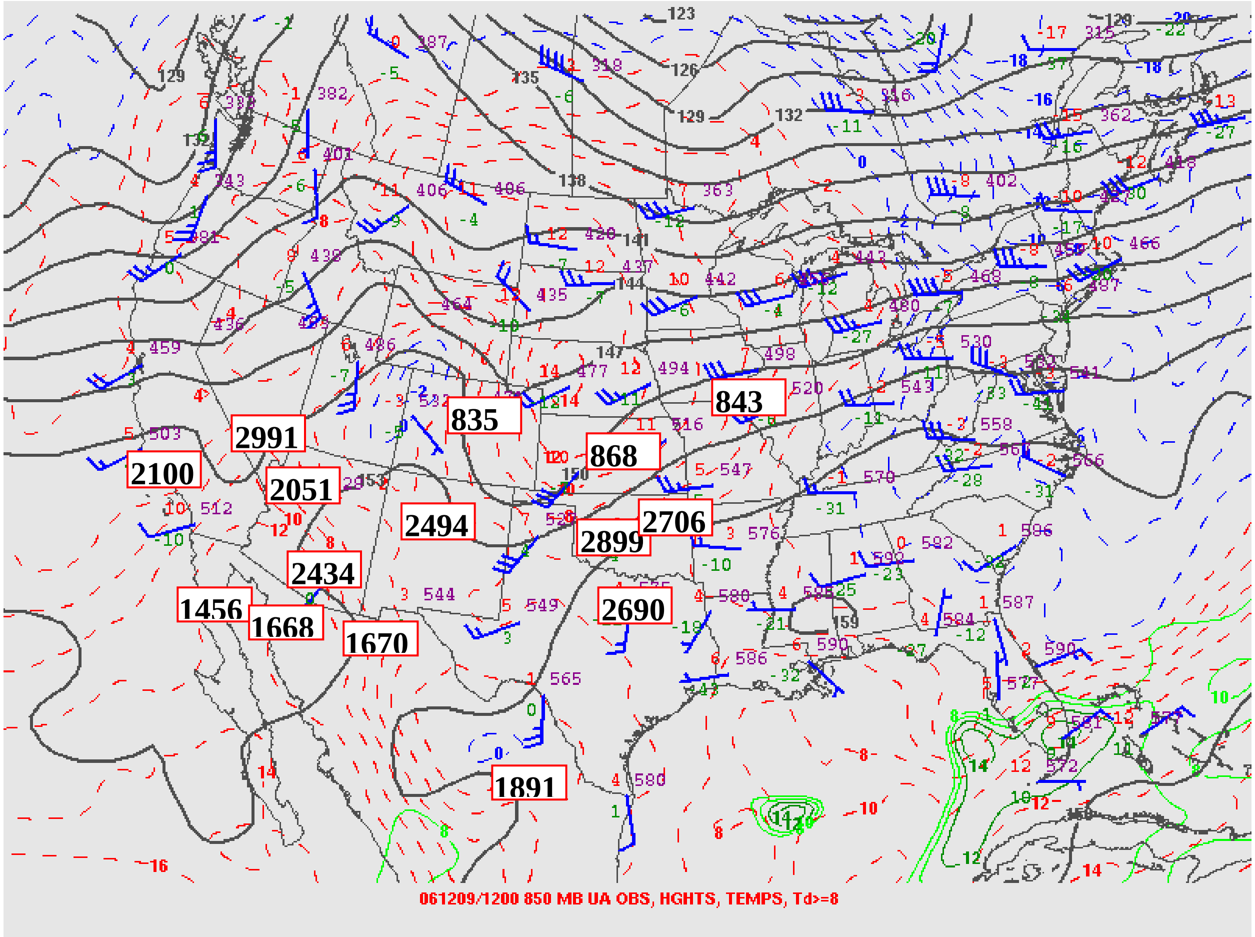

Figure 06G-r2-Used the 12/10/2006 week-ending Physician influenza-like illness report data, with the Dec 09 2006 12 z 850 mb chart. (Courtesy of NOAA NWS; Google Flu Trends).

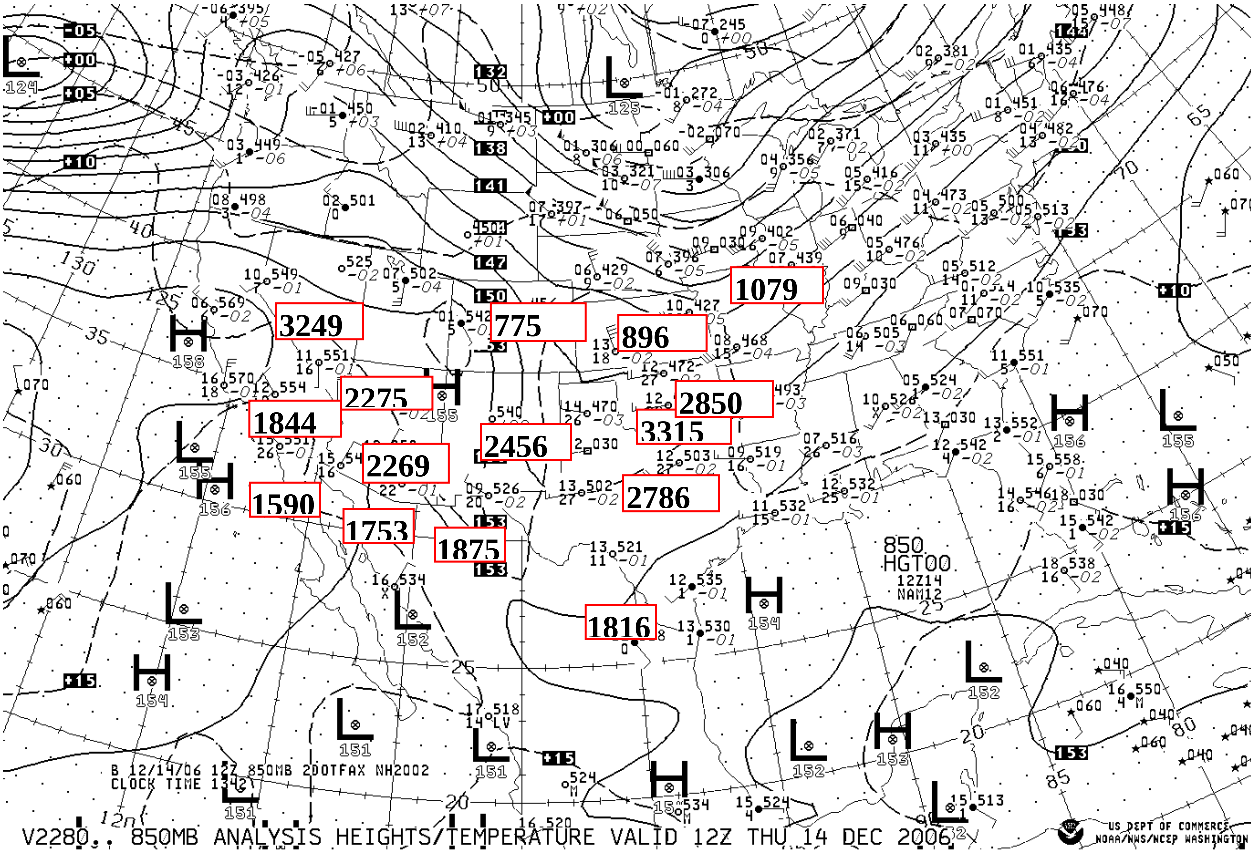

Figure 06G-s1-Used the 12/17/2006 week-ending Physician influenza-like illness report data, with the Dec 14 2006 12 z 850 mb chart. (Courtesy of NOAA NWS; Google Flu Trends).

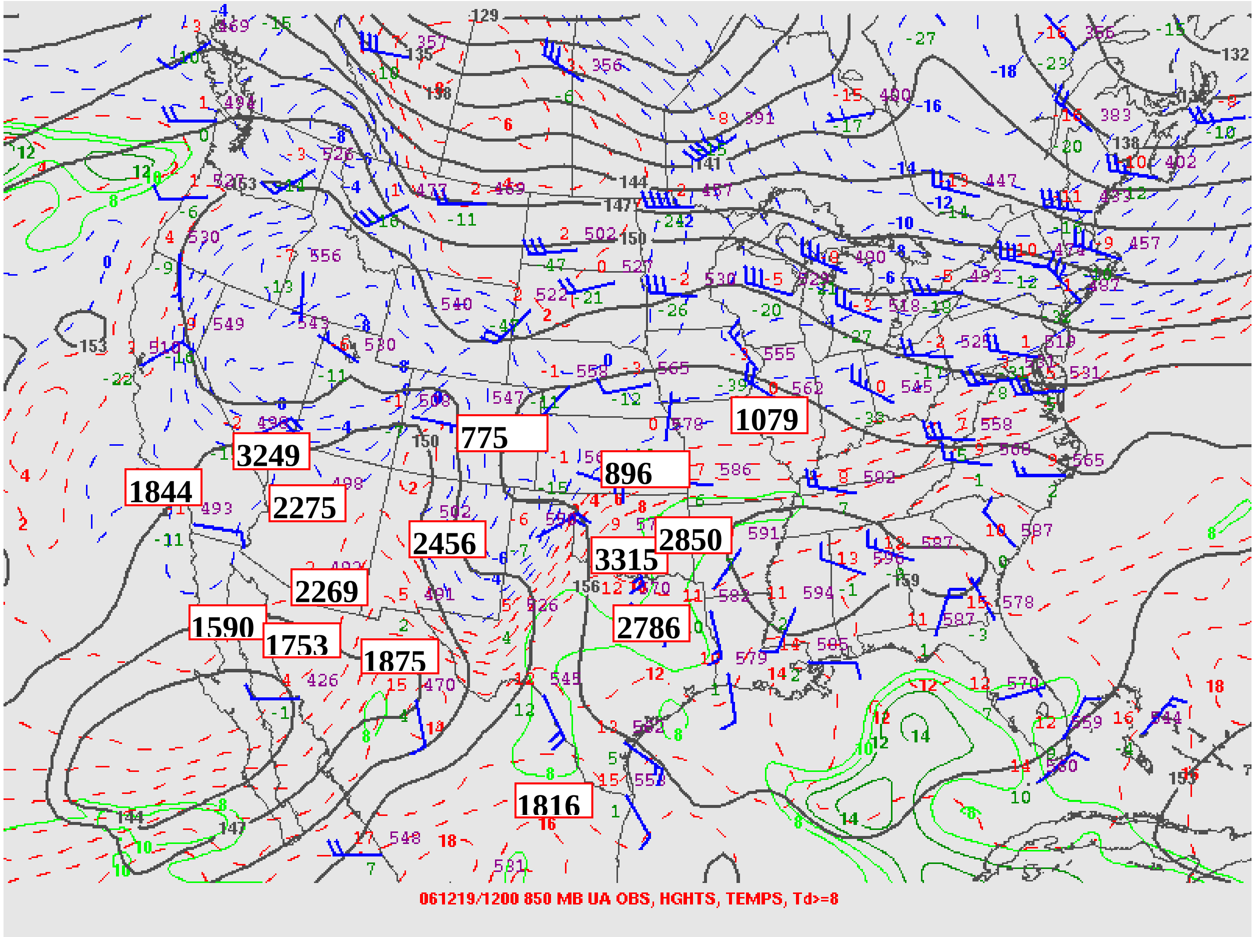

Figure 06G-s2-Used the 12/17/2006 week-ending Physician influenza-like illness report data, with the Dec 19 2006 12 z 850 mb chart. (Courtesy of NOAA NWS; Google Flu Trends).

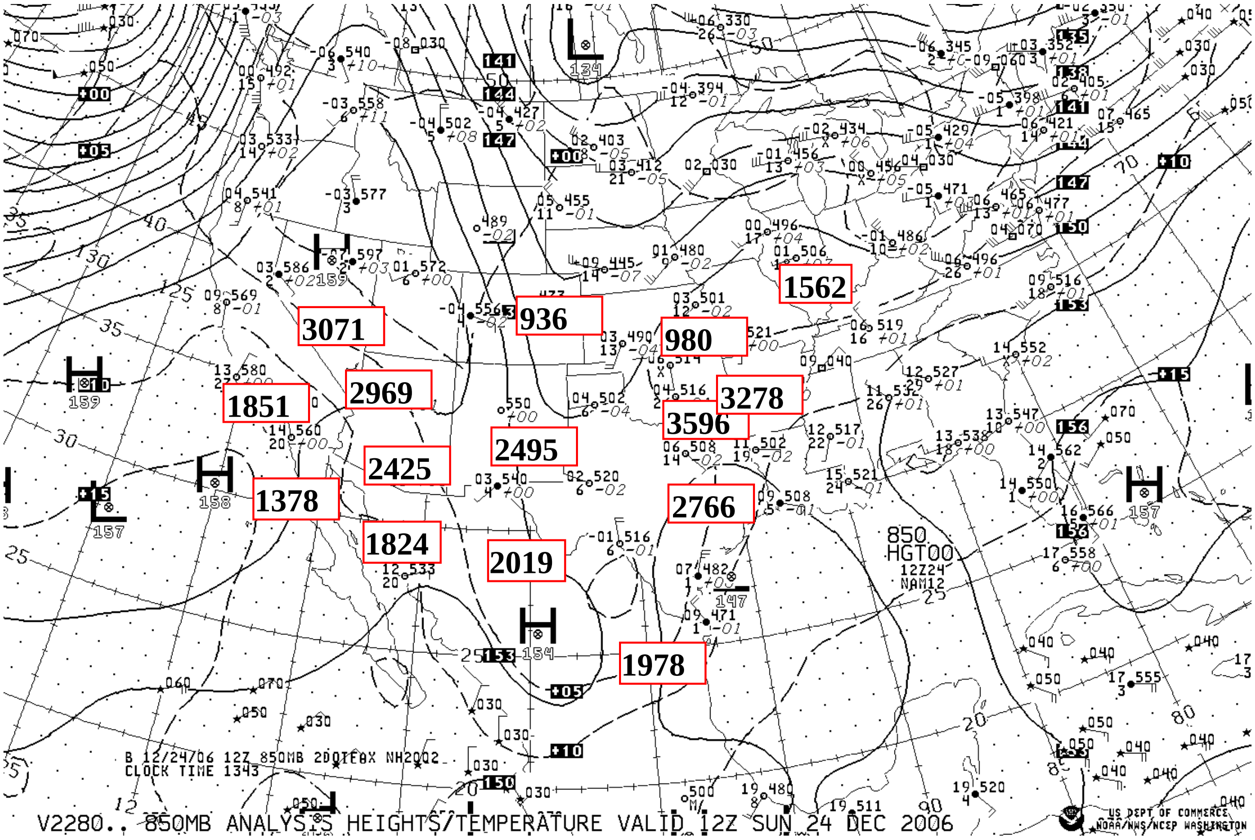

Figure 06G-t-Used the 12/24/2006 week-ending Physician influenza-like illness report data, with the Dec 24 2006 12 z 850 mb chart. (Courtesy of NOAA NWS; Google Flu Trends).

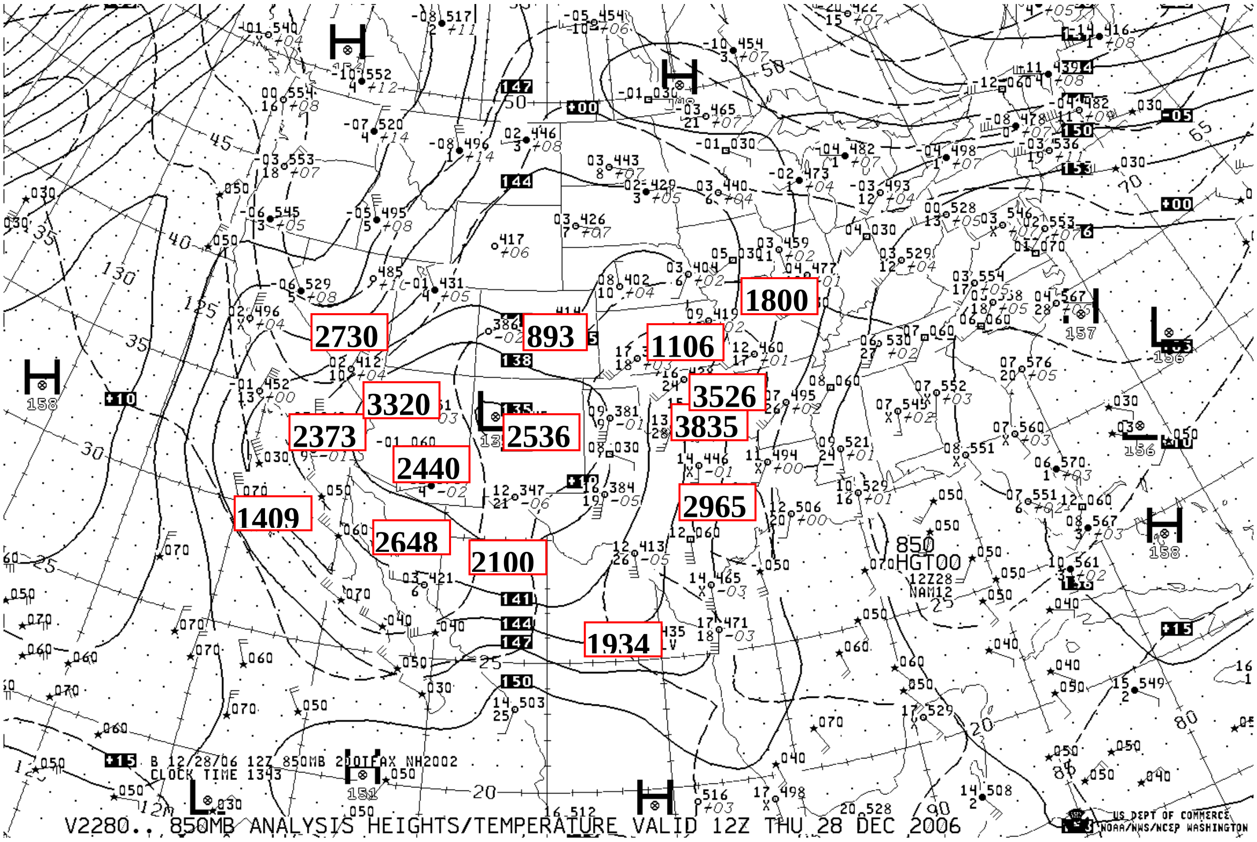

Figure 06G-u1-Used the 12/31/2006 week-ending Physician influenza-like illness report data, with the Dec 28 2006 12 z 850 mb chart. (Courtesy of NOAA NWS; Google Flu Trends).

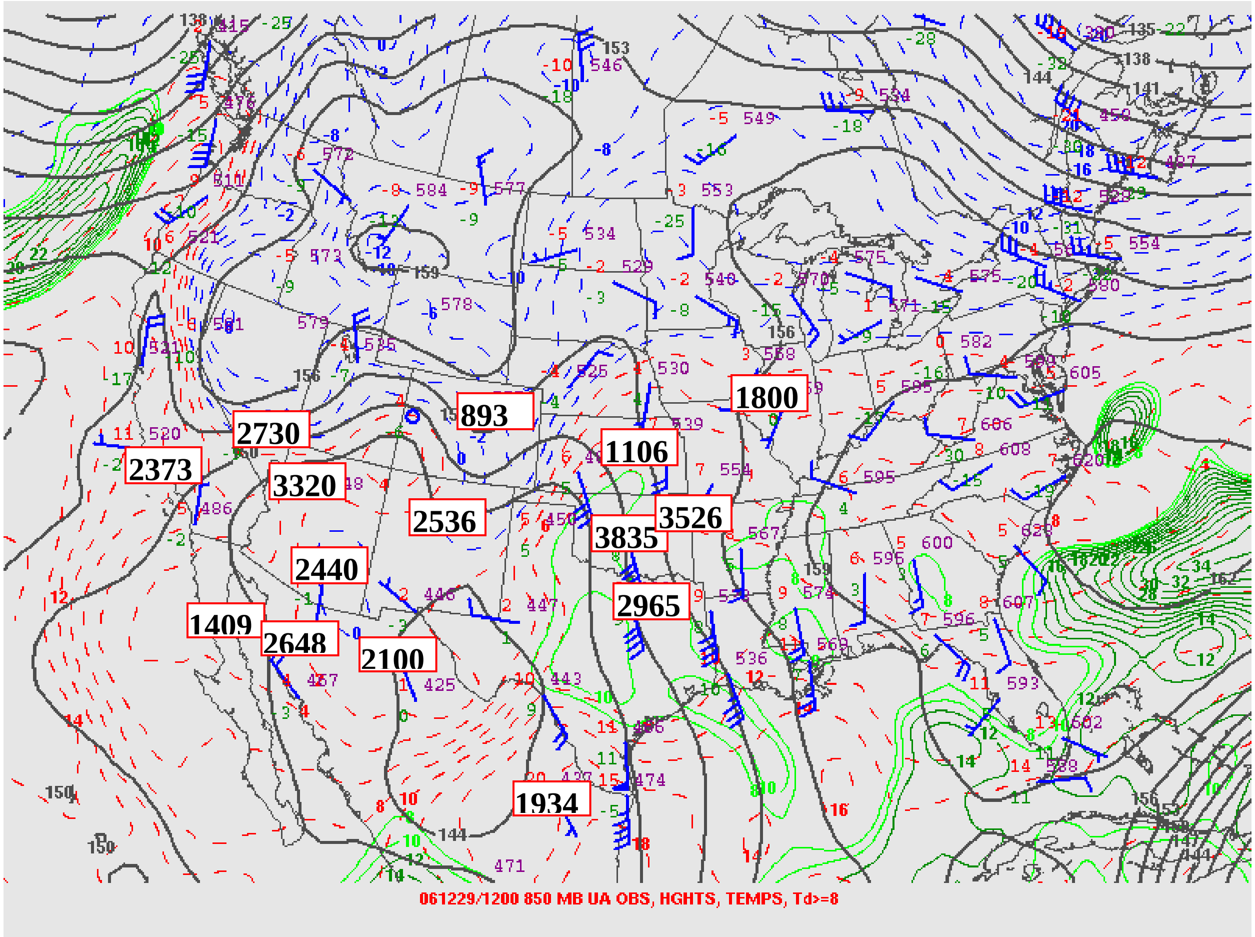

Figure 06G-u2-Used the 12/31/2006 week-ending Physician influenza-like illness report data, with the Dec 29 2006 12 z 850 mb chart. (Courtesy of NOAA NWS; Google Flu Trends).

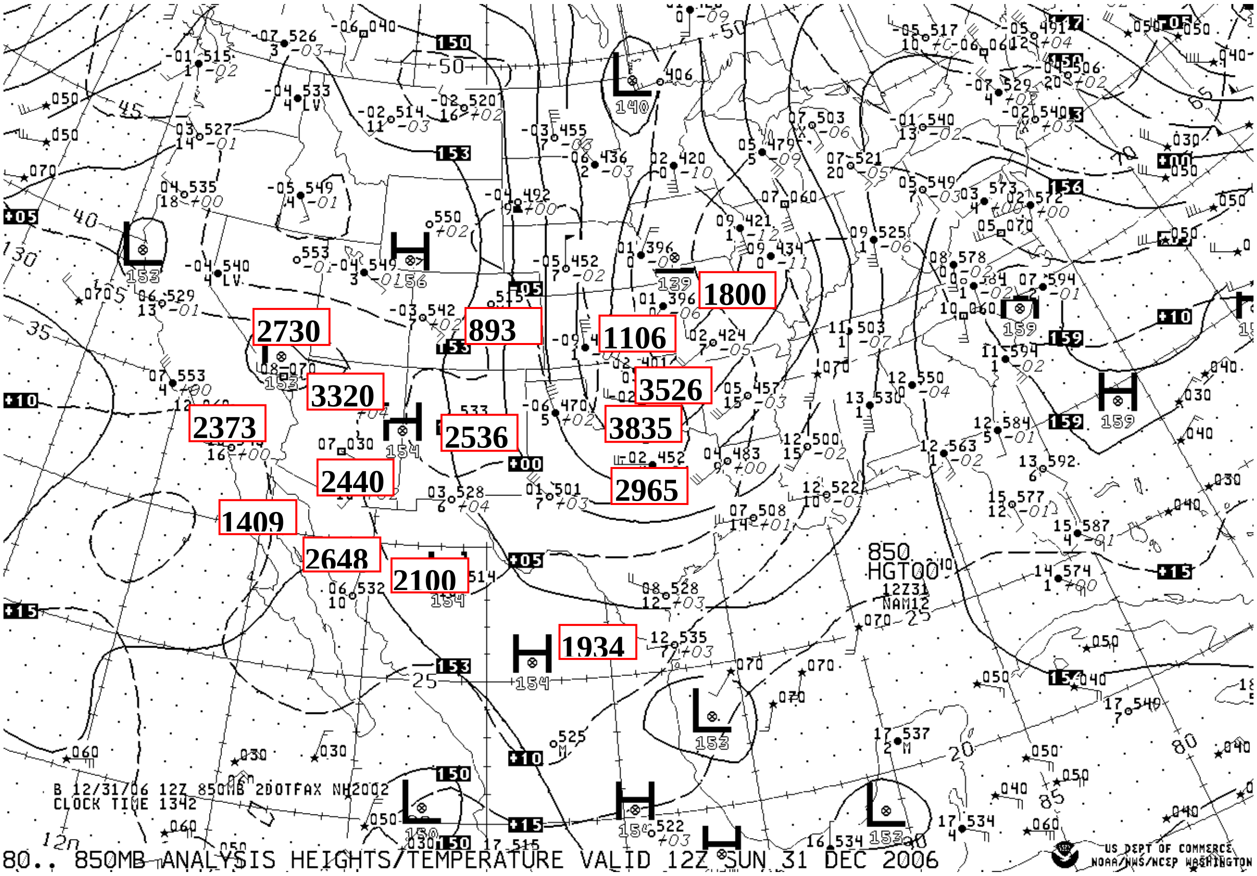

Figure 06G-u3-Used the 12/31/2006 week-ending Physician influenza-like illness report data, with the Dec 31 2006 12 z 850 mb chart. (Courtesy of NOAA NWS; Google Flu Trends).

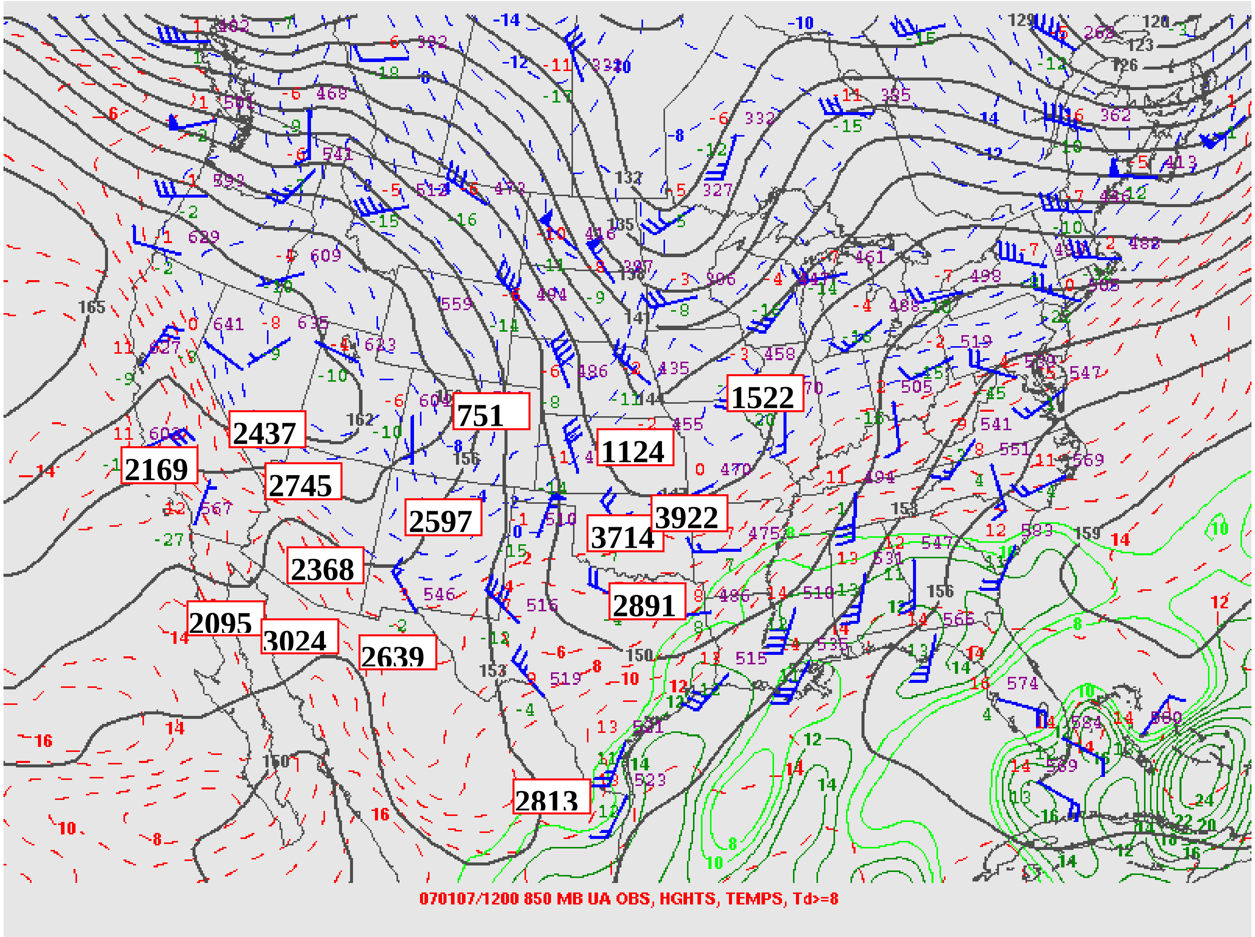

Figure F07-a-Used the 01/07/2007 week-ending Physician influenza-like illness report data, with the Jan 07 2007 12 z 850 mb chart. (Courtesy of NOAA NWS; Google Flu Trends).

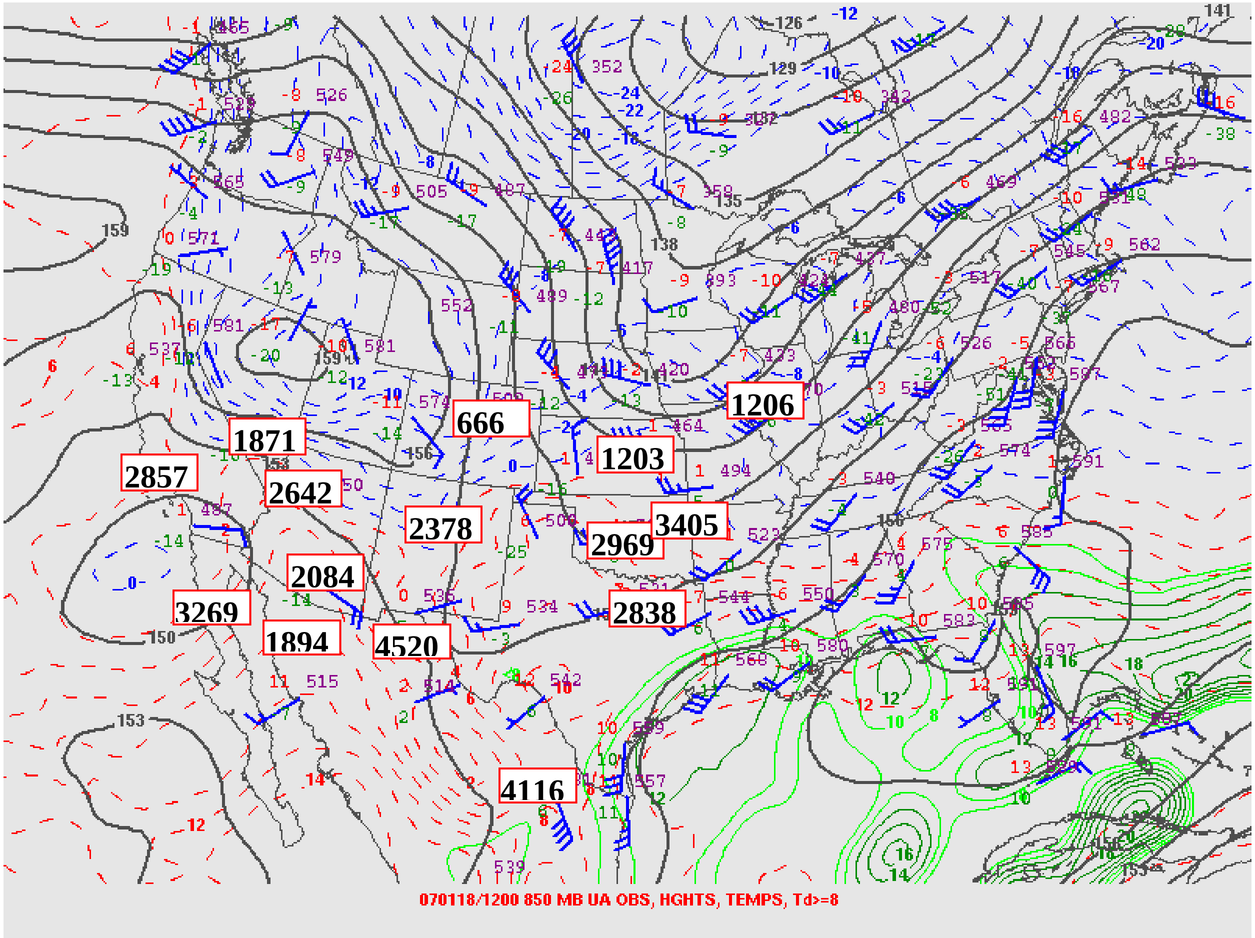

Figure F07-b-Used the 01/21/2007 week-ending Physician influenza-like illness report data, with the Jan 18 2007 12 z 850 mb chart. (Courtesy of NOAA NWS; Google Flu Trends).

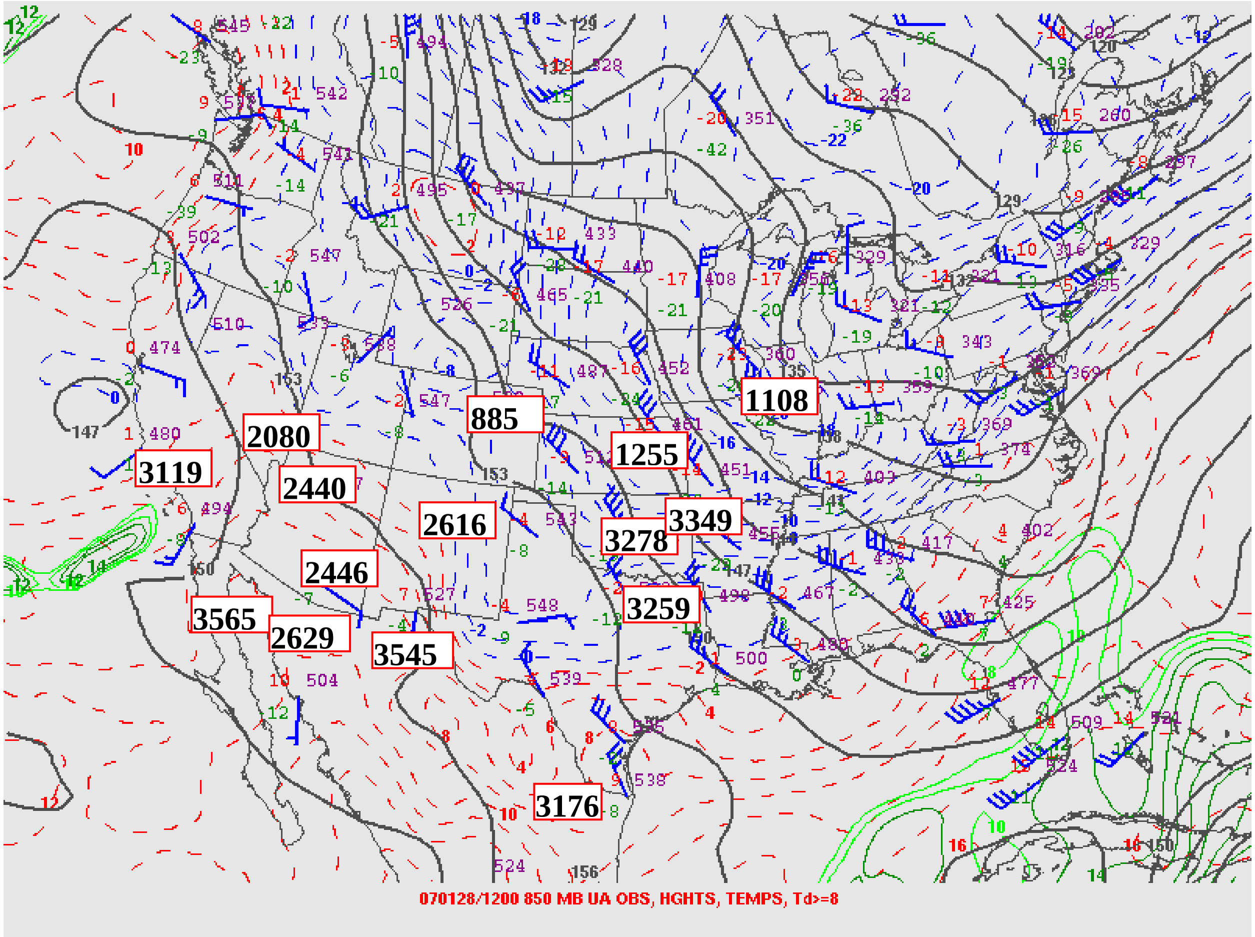

Figure F07-c-Used the 01/28/2007 week-ending Physician influenza-like illness report data, with the Jan 28 2007 12 z 850 mb chart. (Courtesy of NOAA NWS; Google Flu Trends).

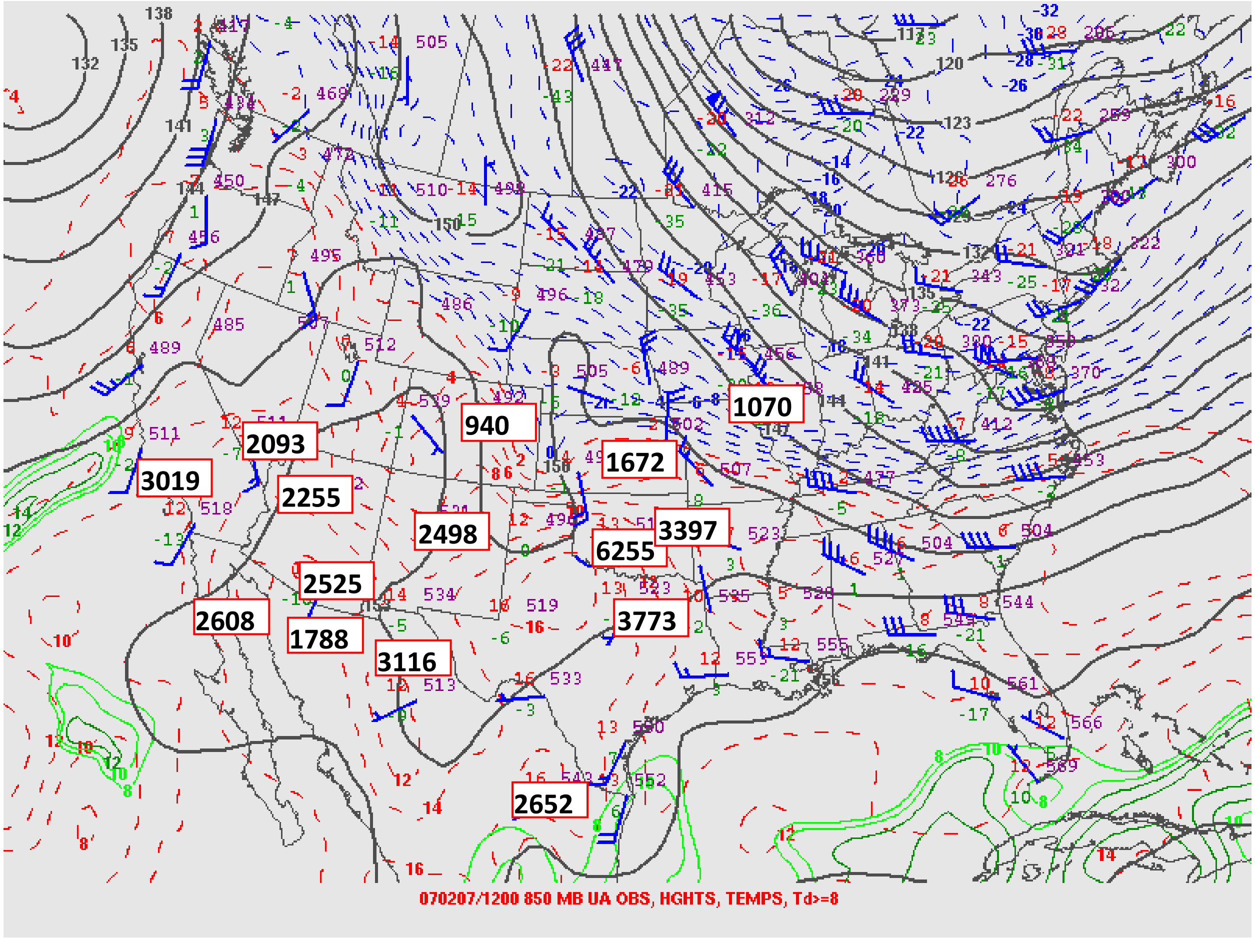

Figure F07-d-Used the 02/04/2007 week-ending Physician influenza-like illness report data, with the Feb 7 2007 12 z 850 mb chart. (Courtesy of NOAA NWS; Google Flu Trends).

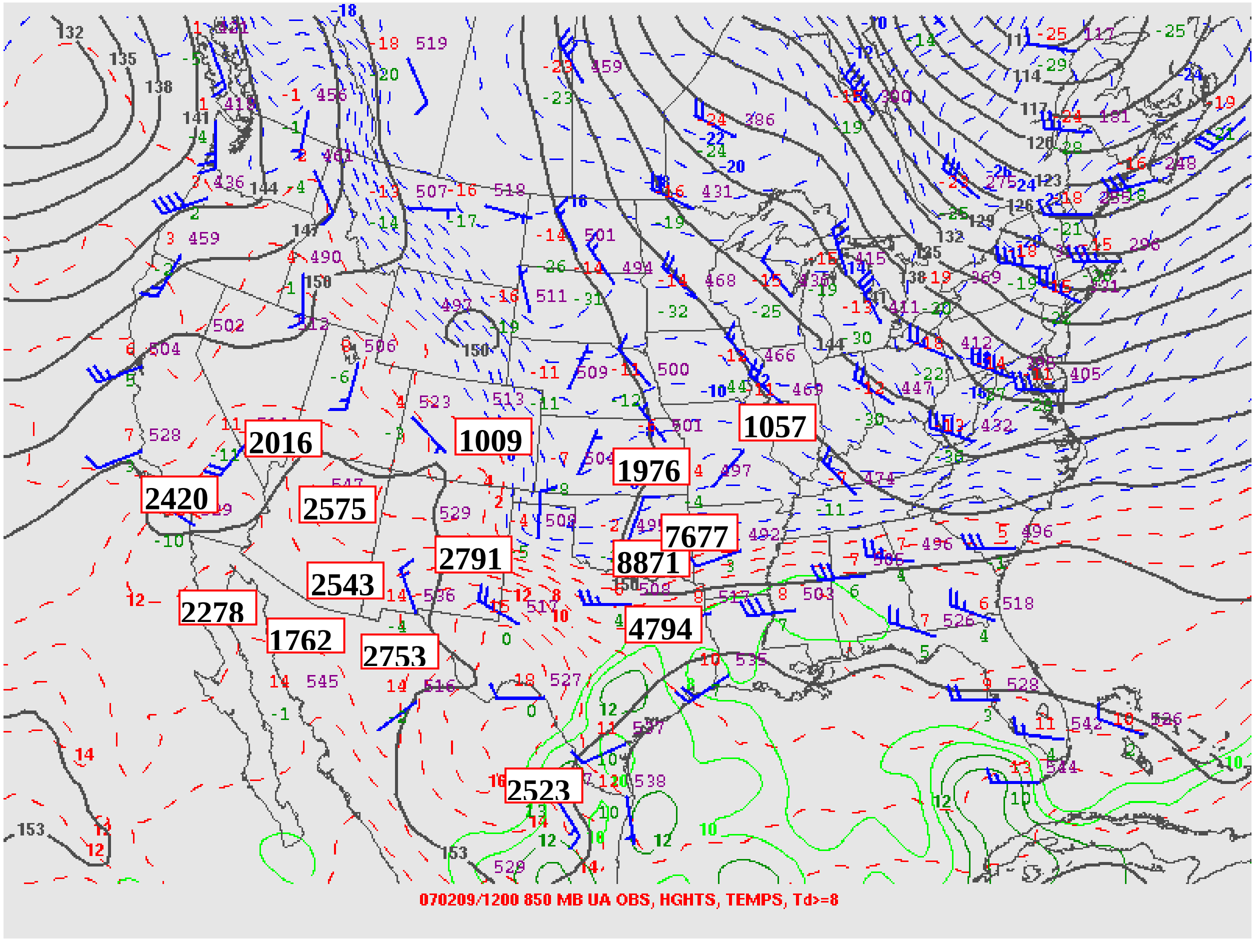

Figure F07-e-Used the 02/11/2007 week-ending Physician influenza-like illness report data, with the Feb 09 2007 12 z 850 mb chart. (Courtesy of NOAA NWS; Google Flu Trends).

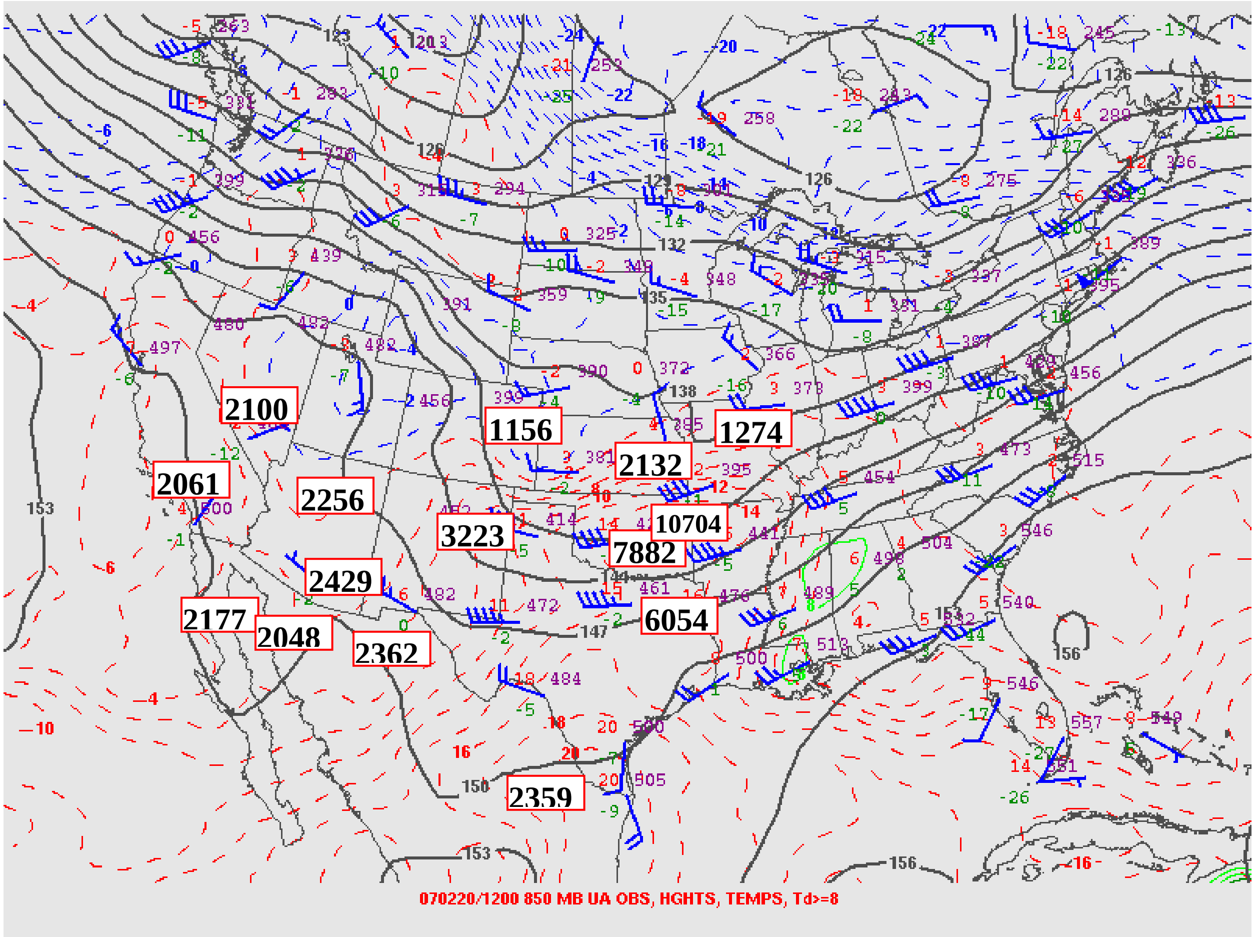

Figure F07-f-Used the 02/18/2007 week-ending Physician influenza-like illness report data, with the Feb 20 2007 12 z 850 mb chart. (Courtesy of NOAA NWS; Google Flu Trends).

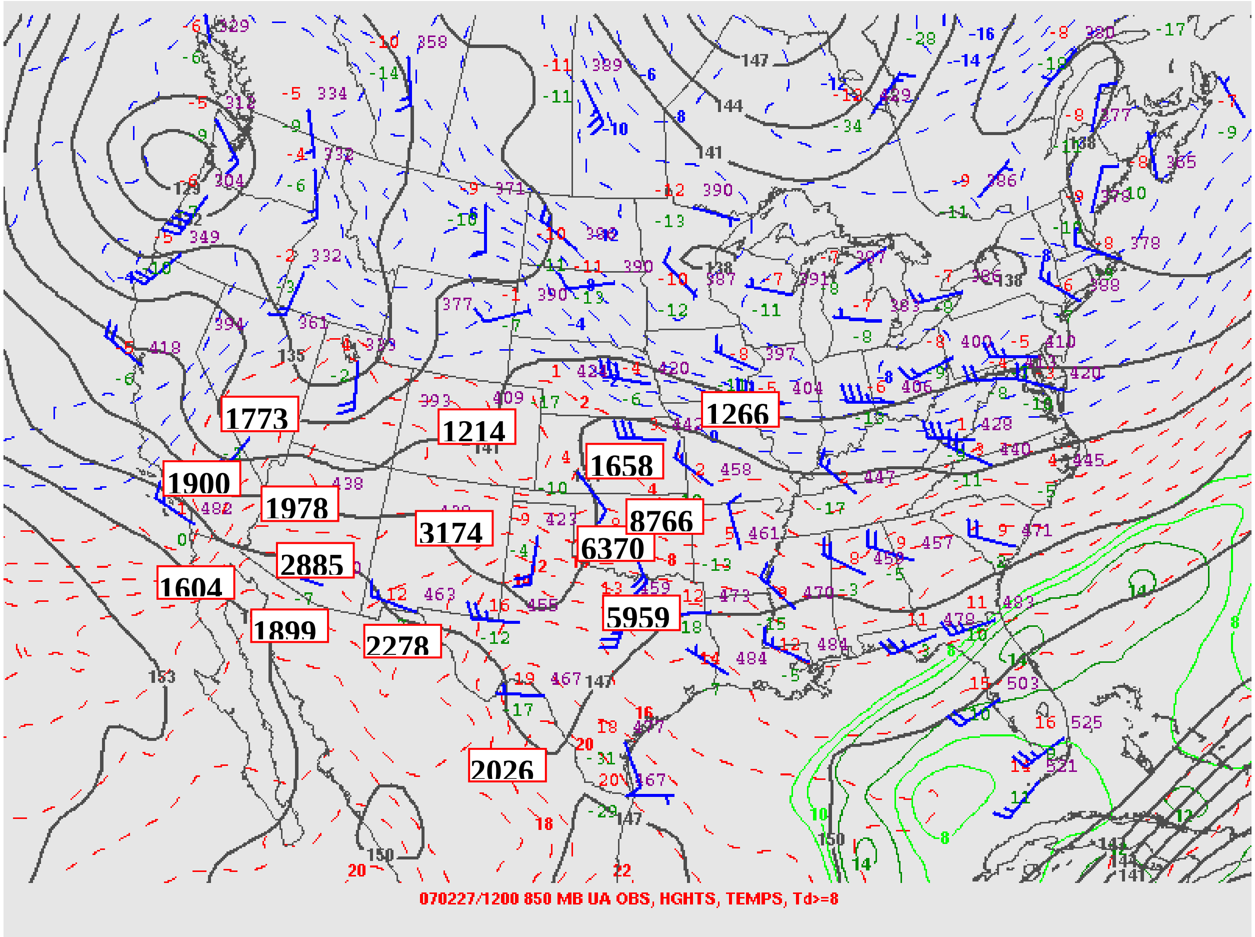

Figure F07-g-Used the 02/25/2007 week-ending Physician influenza-like illness report data, with the Feb 27 2007 12 z 850 mb chart. (Courtesy of NOAA NWS; Google Flu Trends).

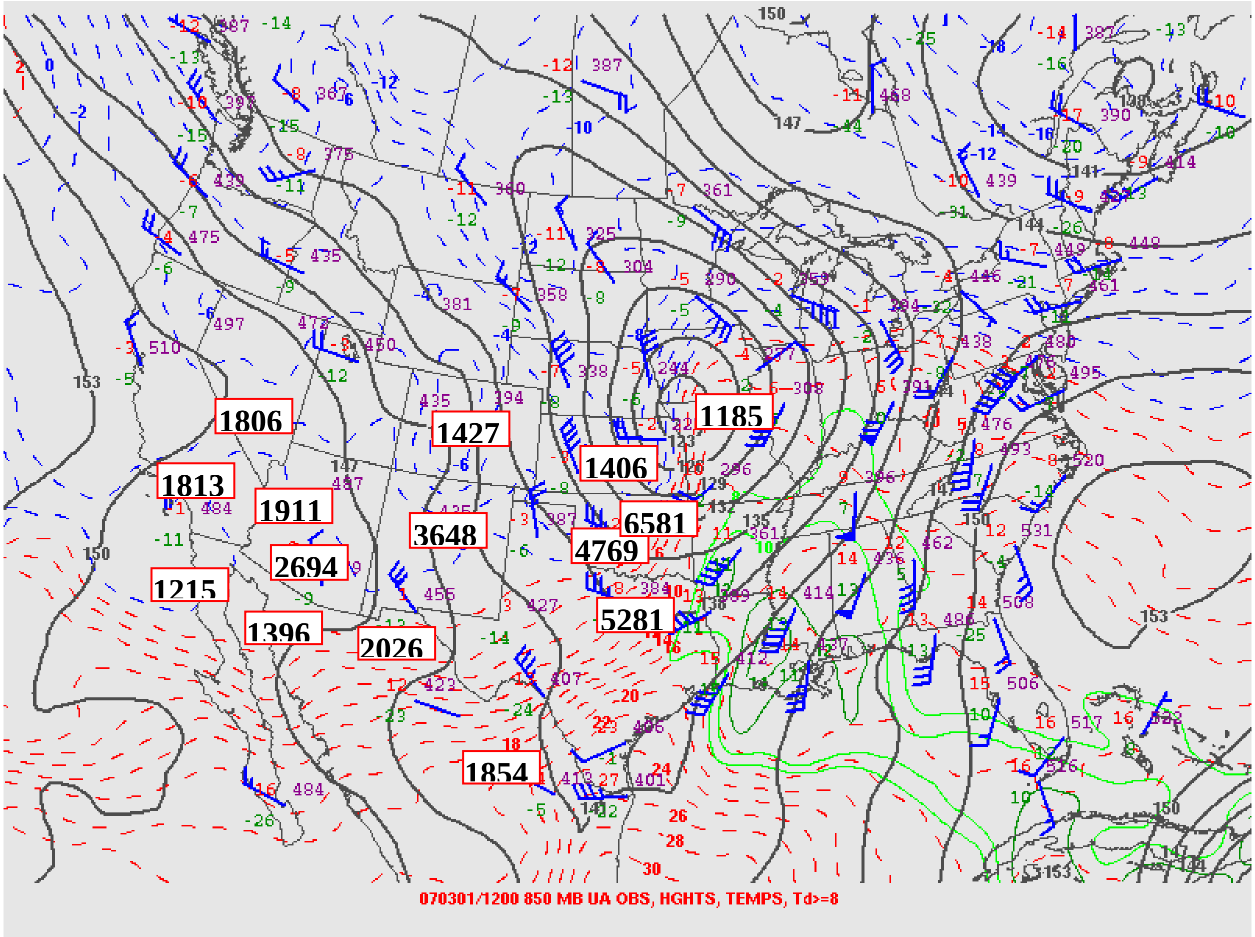

Figure F07-h-Used the 03/04/2007 week-ending Physician influenza-like illness report data, with the Mar 01 2007 12 z 850 mb chart. (Courtesy of NOAA NWS; Google Flu Trends).

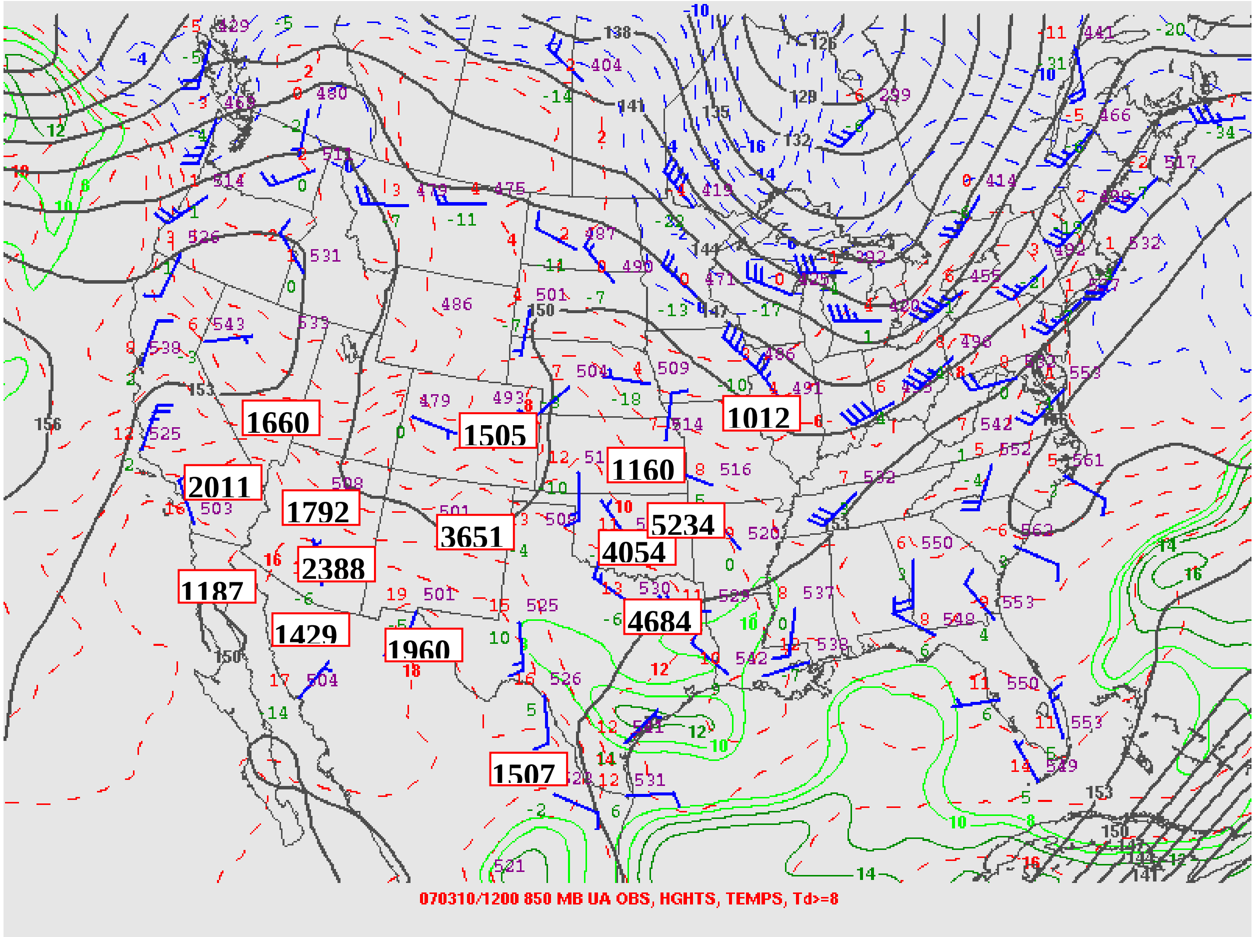

Figure F07-i-Used the 03/11/2007 week-ending Physician influenza-like illness report data, with the Mar 10 2007 12 z 850 mb chart. (Courtesy of NOAA NWS; Google Flu Trends).

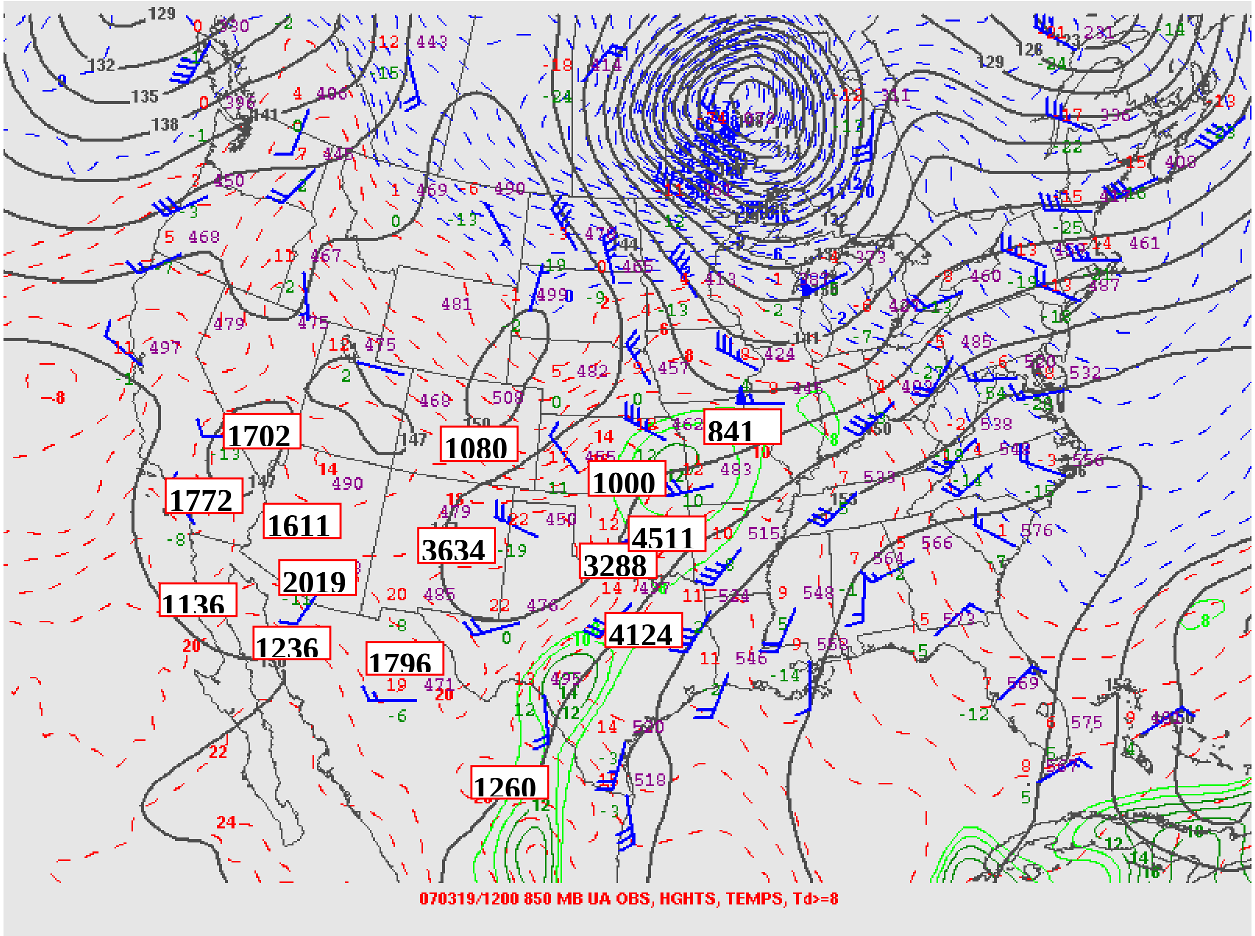

Figure F07-j-Used the 03/18/2007 week-ending Physician influenza-like illness report data, with the Mar 19 2007 12 z 850 mb chart. (Courtesy of NOAA NWS; Google Flu Trends).

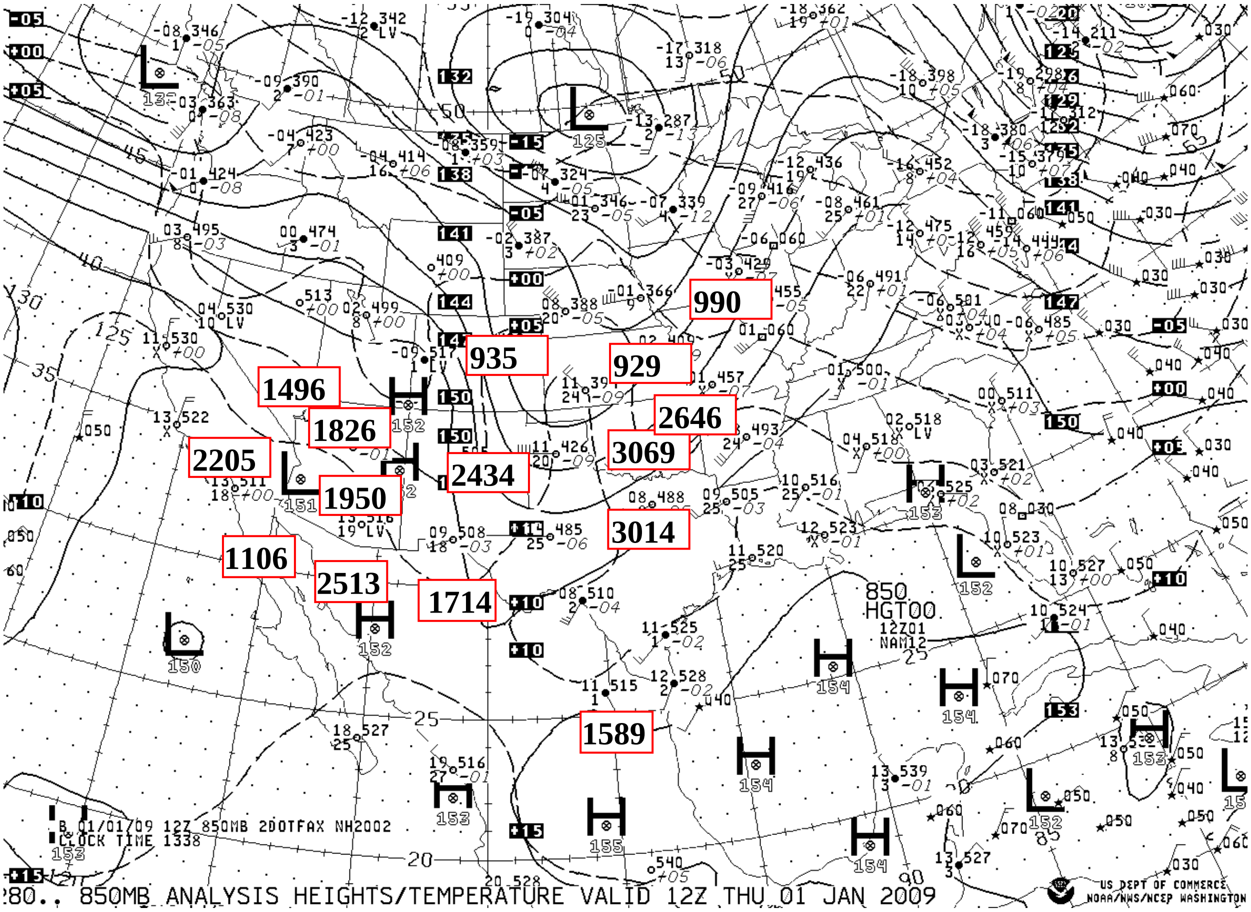

Figure F09-a1-Used the 01/04/2009 week-ending Physician influenza-like illness report data, with the Jan 01 2009 12 z 850 mb chart. (Courtesy of NOAA NWS; Google Flu Trends).

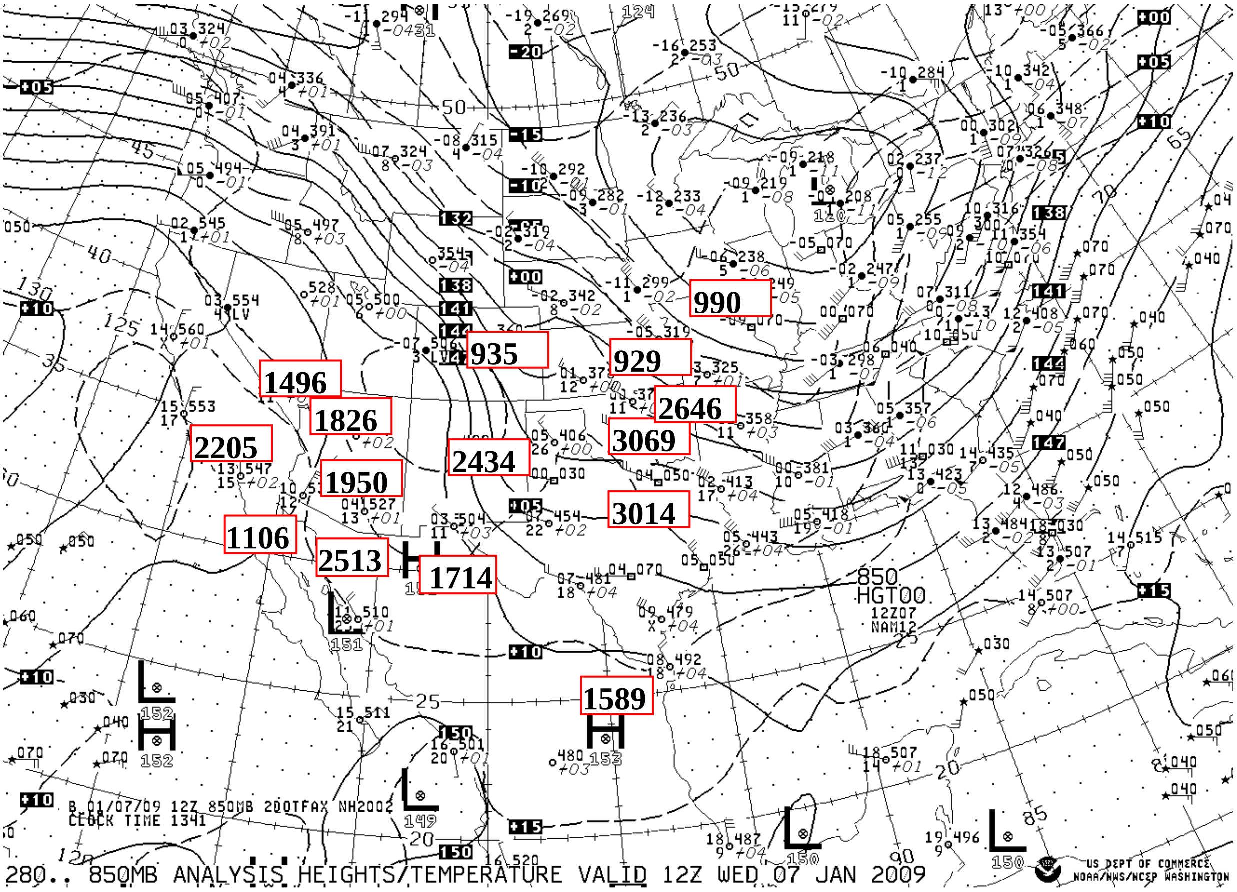

Figure F09-a2-Used the 01/04/2009 week-ending Physician influenza-like illness report data, with the Jan 07 2009 12 z 850 mb chart. (Courtesy of NOAA NWS; Google Flu Trends).

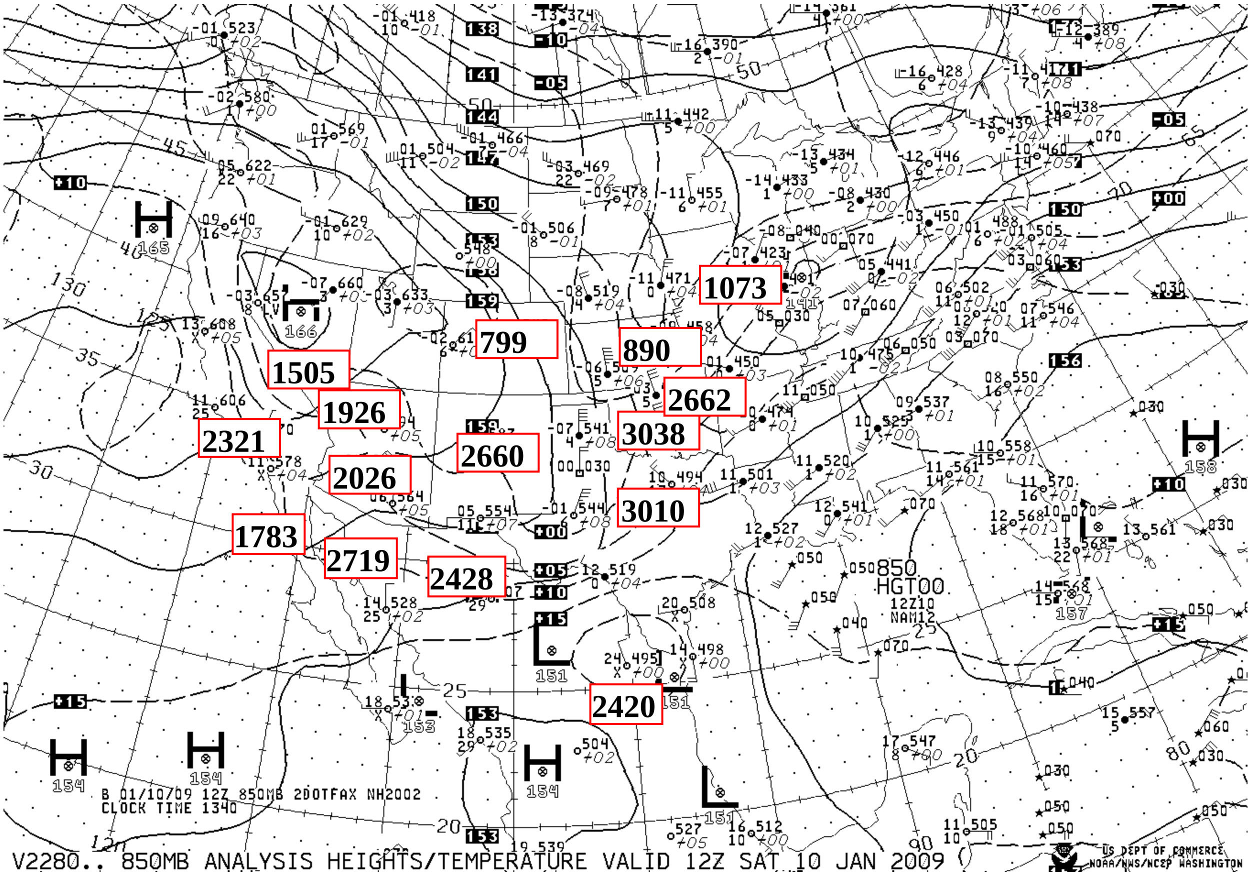

Figure F09-b1-Used the 01/11/2009 week-ending Physician influenza-like illness report data, with the Jan 10 2009 12 z 850 mb chart. (Courtesy of NOAA NWS; Google Flu Trends).

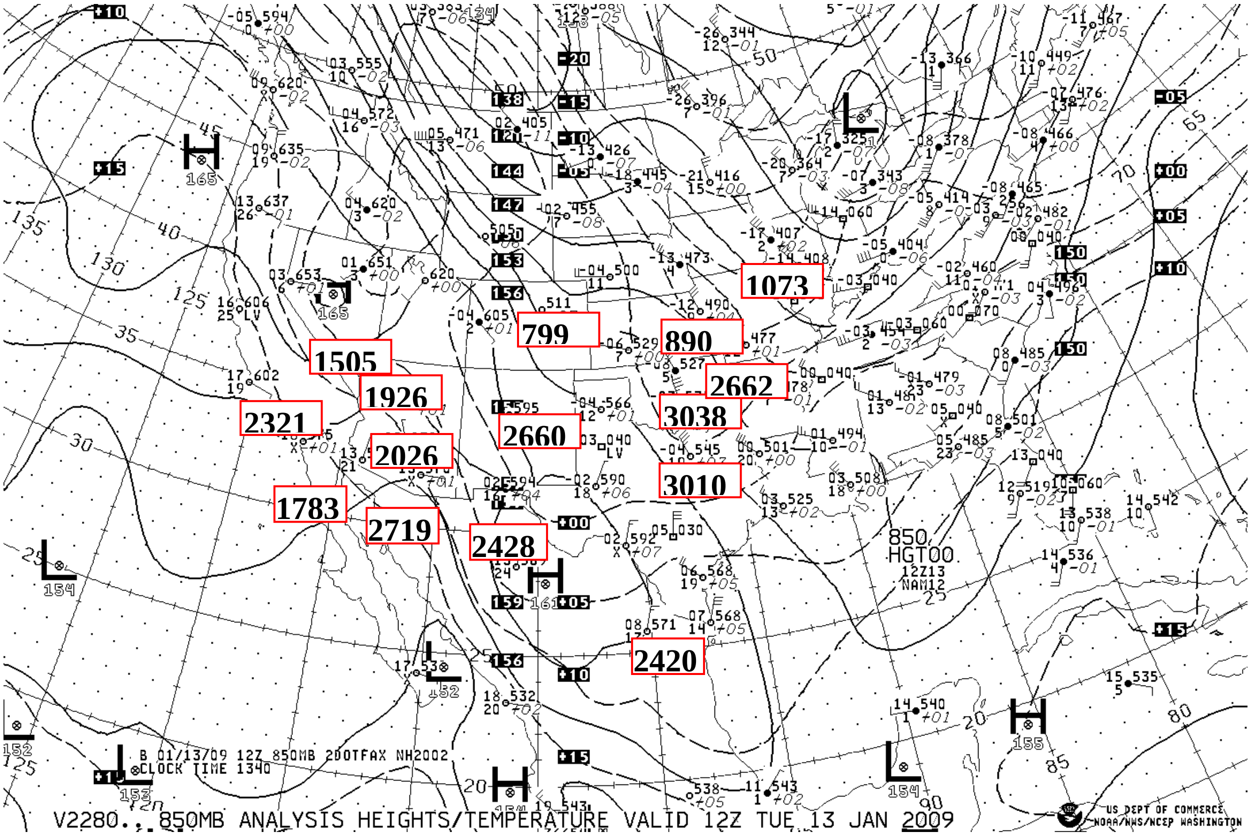

Figure F09-b2-Used the 01/11/2009 week-ending Physician influenza-like illness report data, with the Jan 13 2009 12 z 850 mb chart. (Courtesy of NOAA NWS; Google Flu Trends).

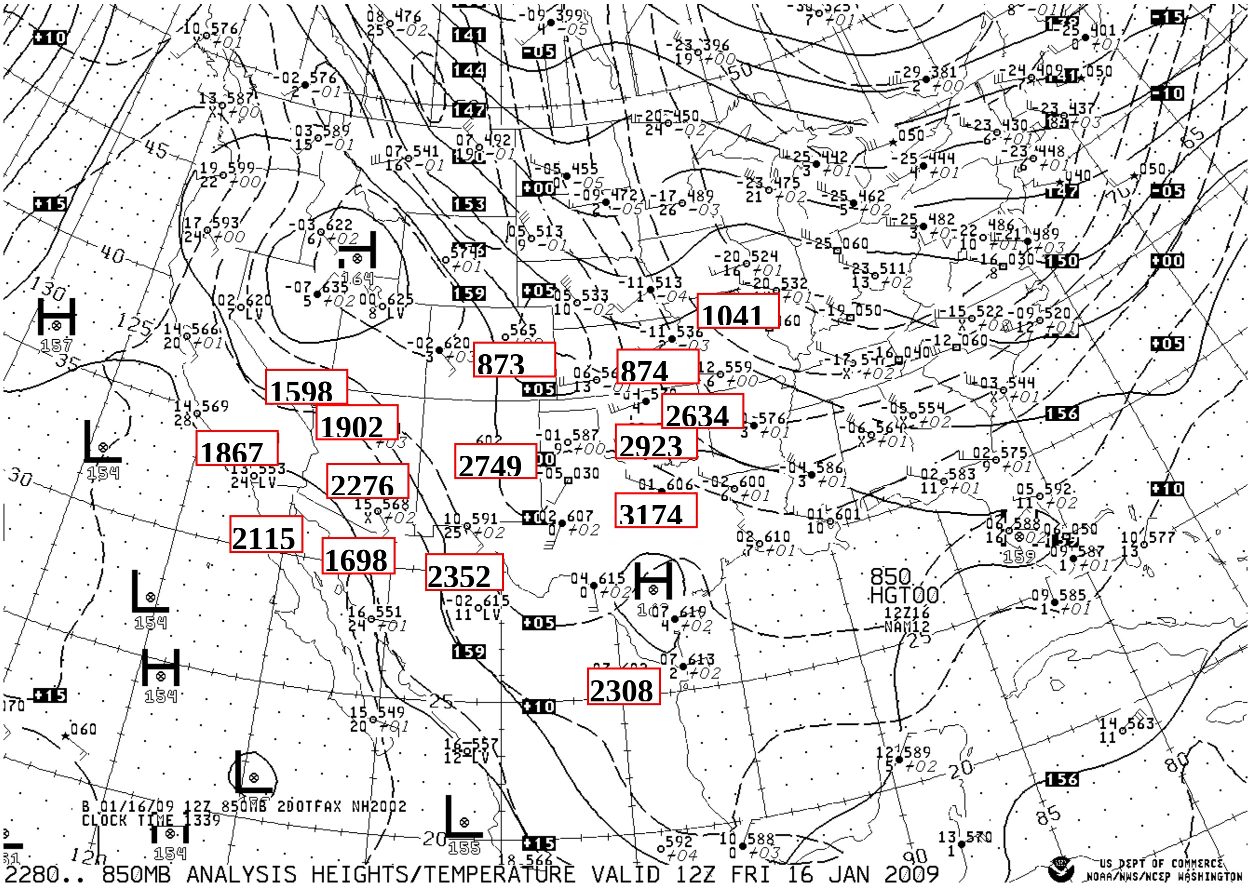

Figure F09-c1-Used the 01/18/2009 week-ending Physician influenza-like illness report data, with the Jan 16 2009 12 z 850 mb chart. (Courtesy of NOAA NWS; Google Flu Trends).

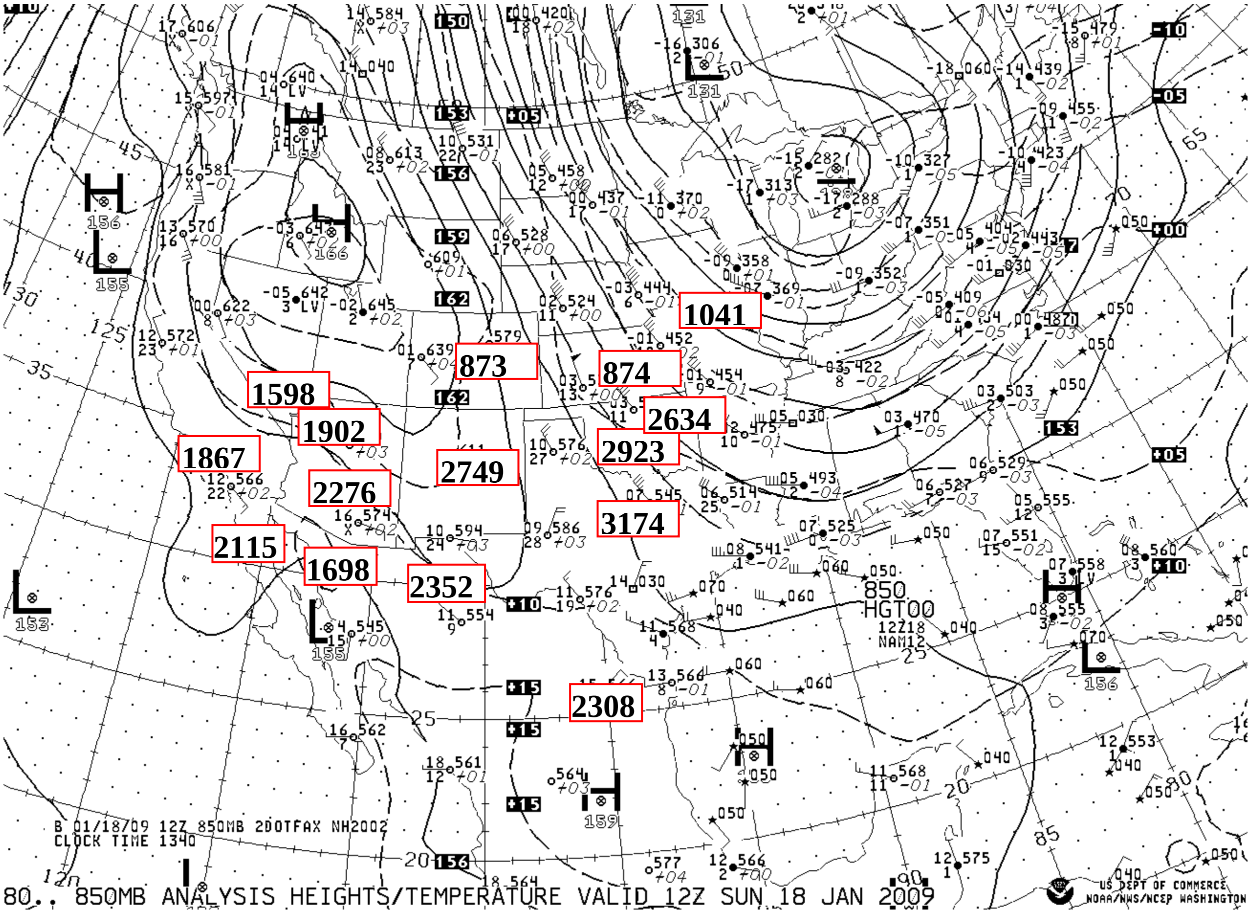

Figure F09-c2-Used the 01/18/2009 week-ending Physician influenza-like illness report data, with the Jan 18 2009 12 z 850 mb chart. (Courtesy of NOAA NWS; Google Flu Trends).

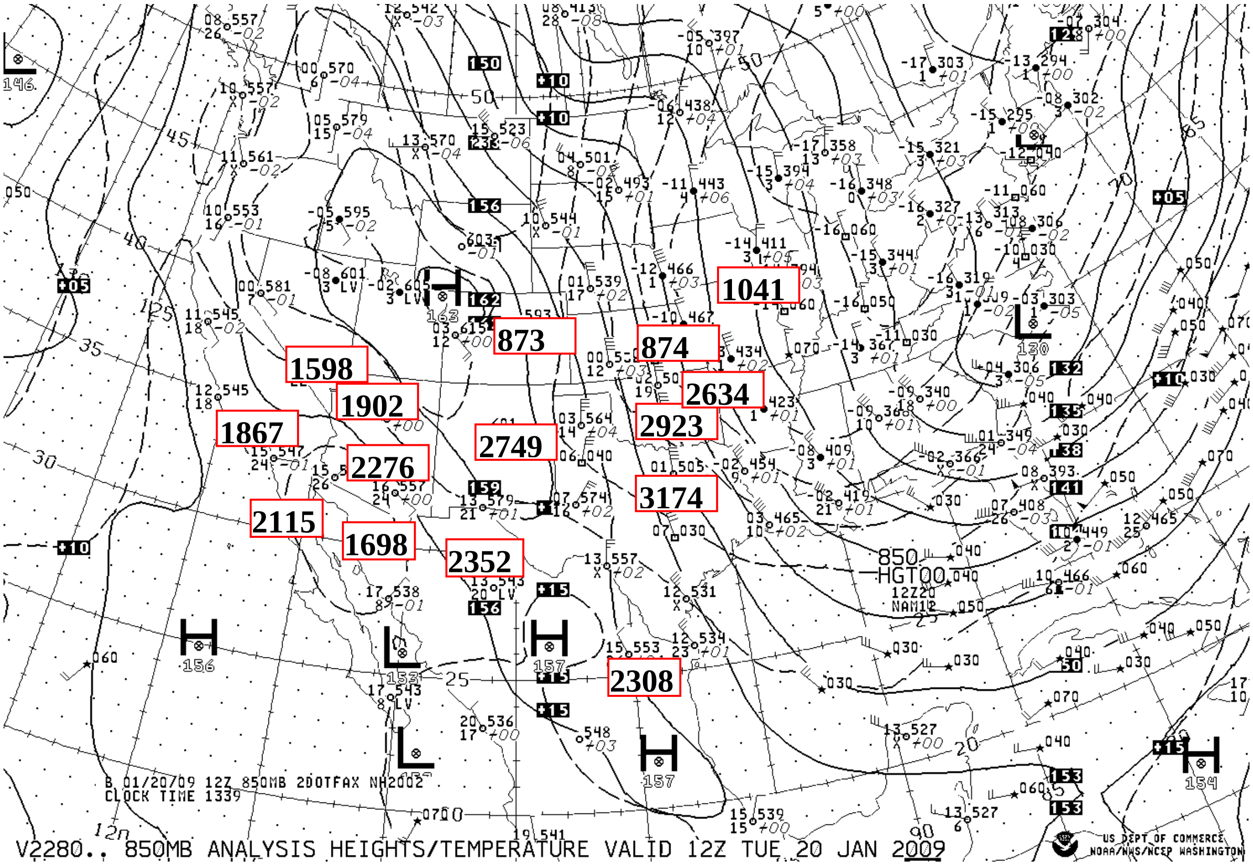

Figure F09-c3-Used the 01/18/2009 week-ending Physician influenza-like illness report data, with the Jan 20 2009 12 z 850 mb chart. (Courtesy of NOAA NWS; Google Flu Trends).

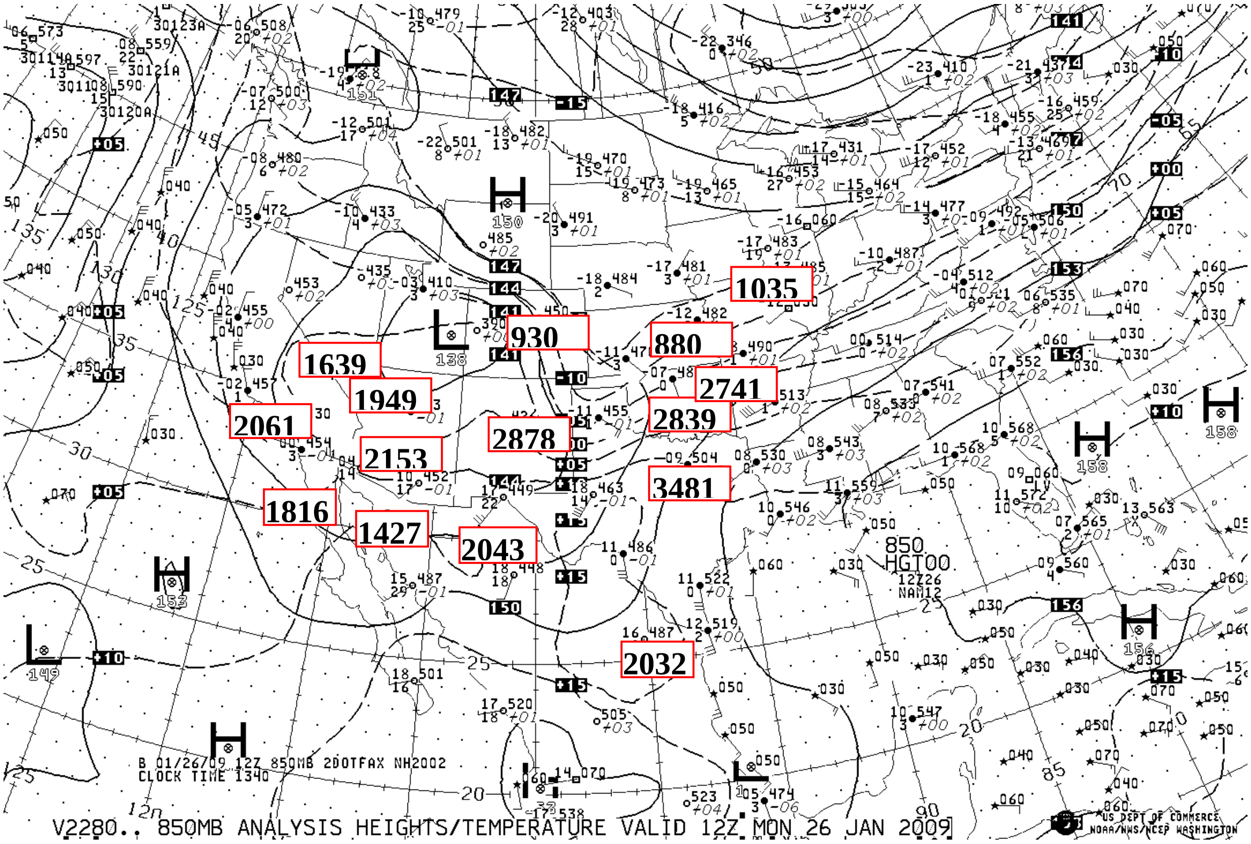

Figure F09-d-Used the 01/25/2009 week-ending Physician influenza-like illness report data, with the Jan 26 2009 12 z 850 mb chart. (Courtesy of NOAA NWS; Google Flu Trends).

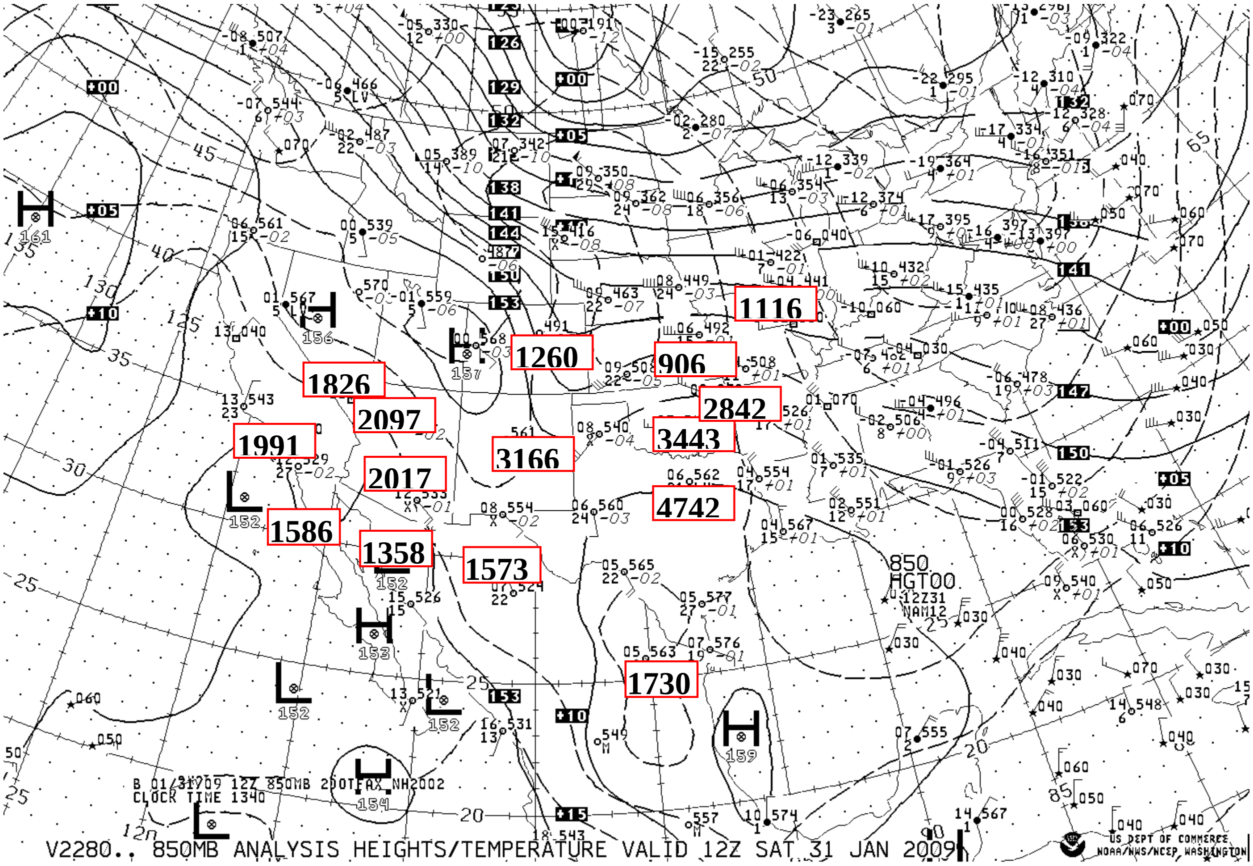

Figure F09-e1-Used the 02/01/2009 week-ending Physician influenza-like illness report data, with the Jan 31 2009 12 z 850 mb chart. (Courtesy of NOAA NWS; Google Flu Trends).

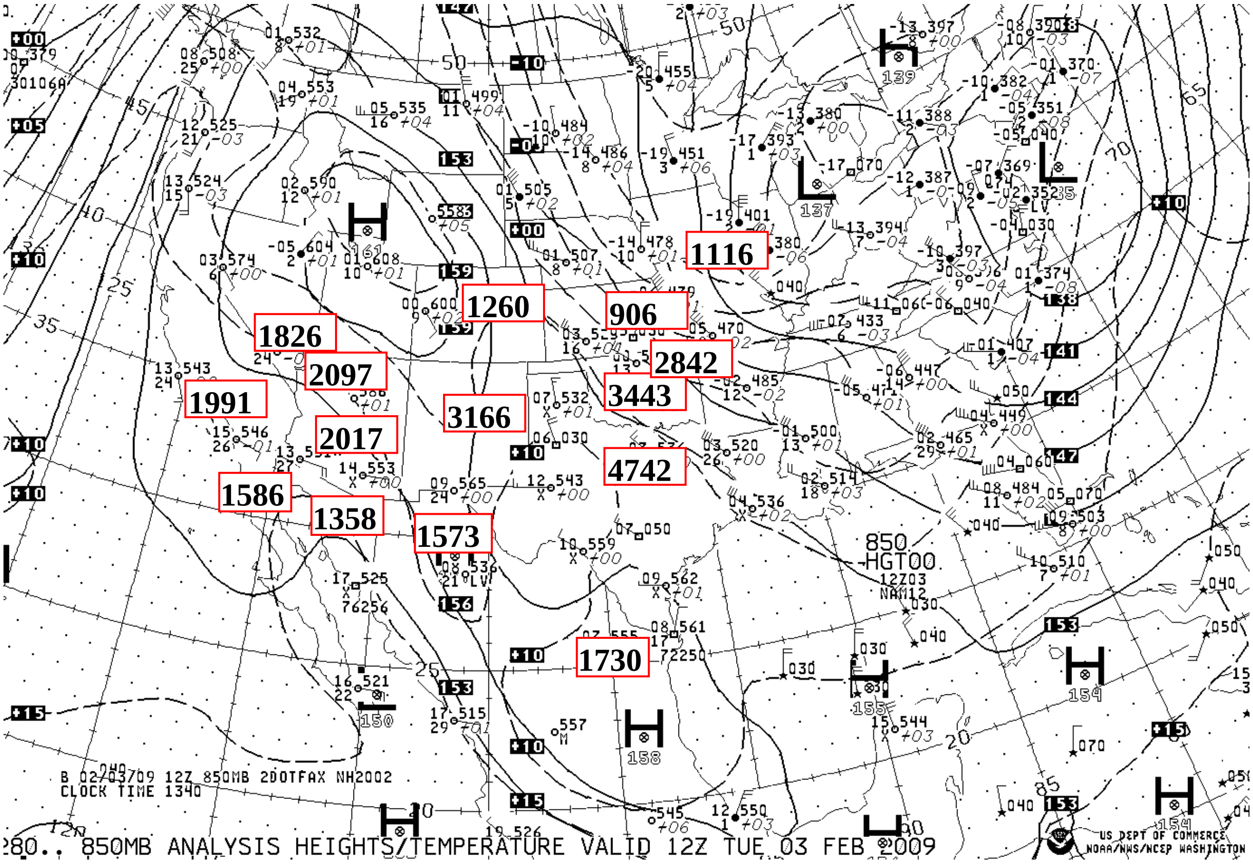

Figure F09- e2-Used the 02/01/2009 week-ending Physician influenza-like illness report data, with the Feb 03 2009 12 z 850 mb chart. (Courtesy of NOAA NWS; Google Flu Trends).

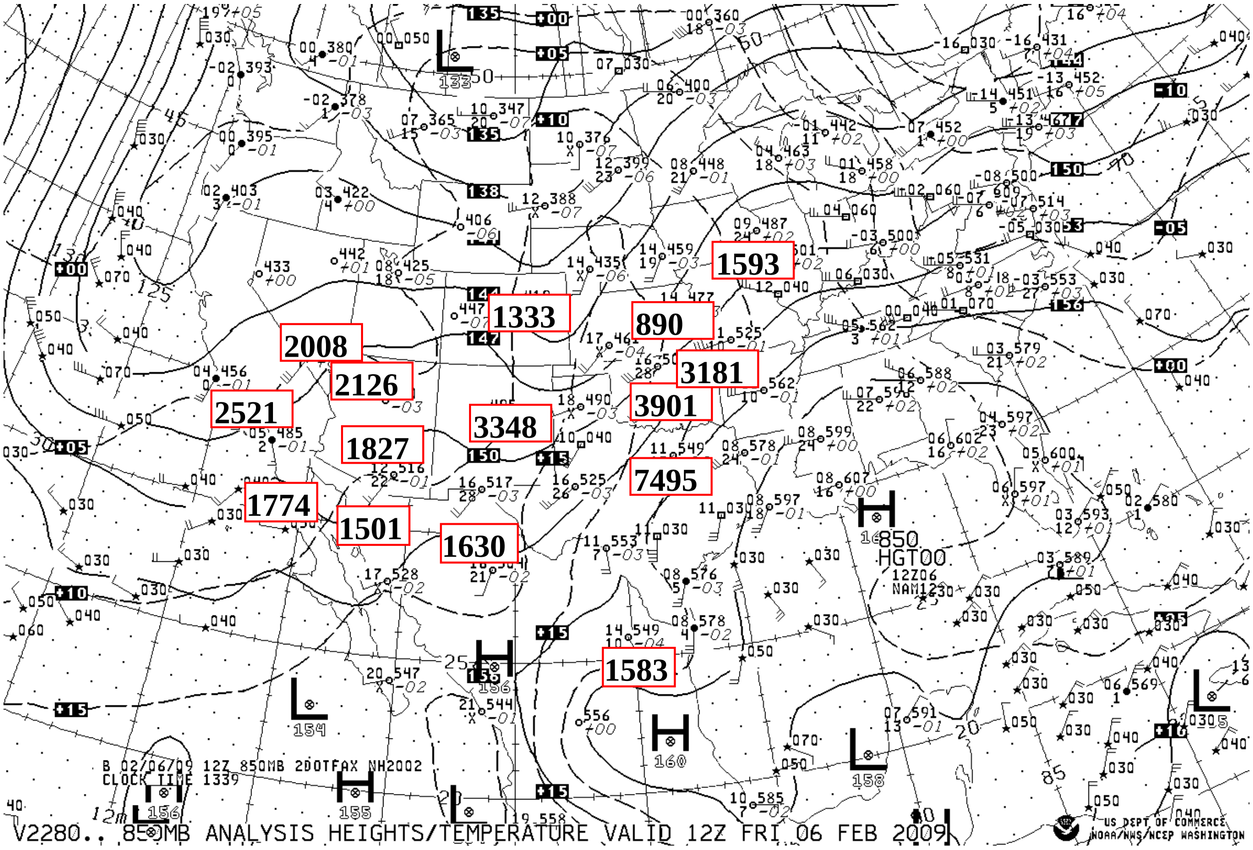

Figure F09-f1-Used the 02/08/2009 week-ending Physician influenza-like illness report data, with the Feb 06 2009 12 z 850 mb chart. (Courtesy of NOAA NWS; Google Flu Trends).

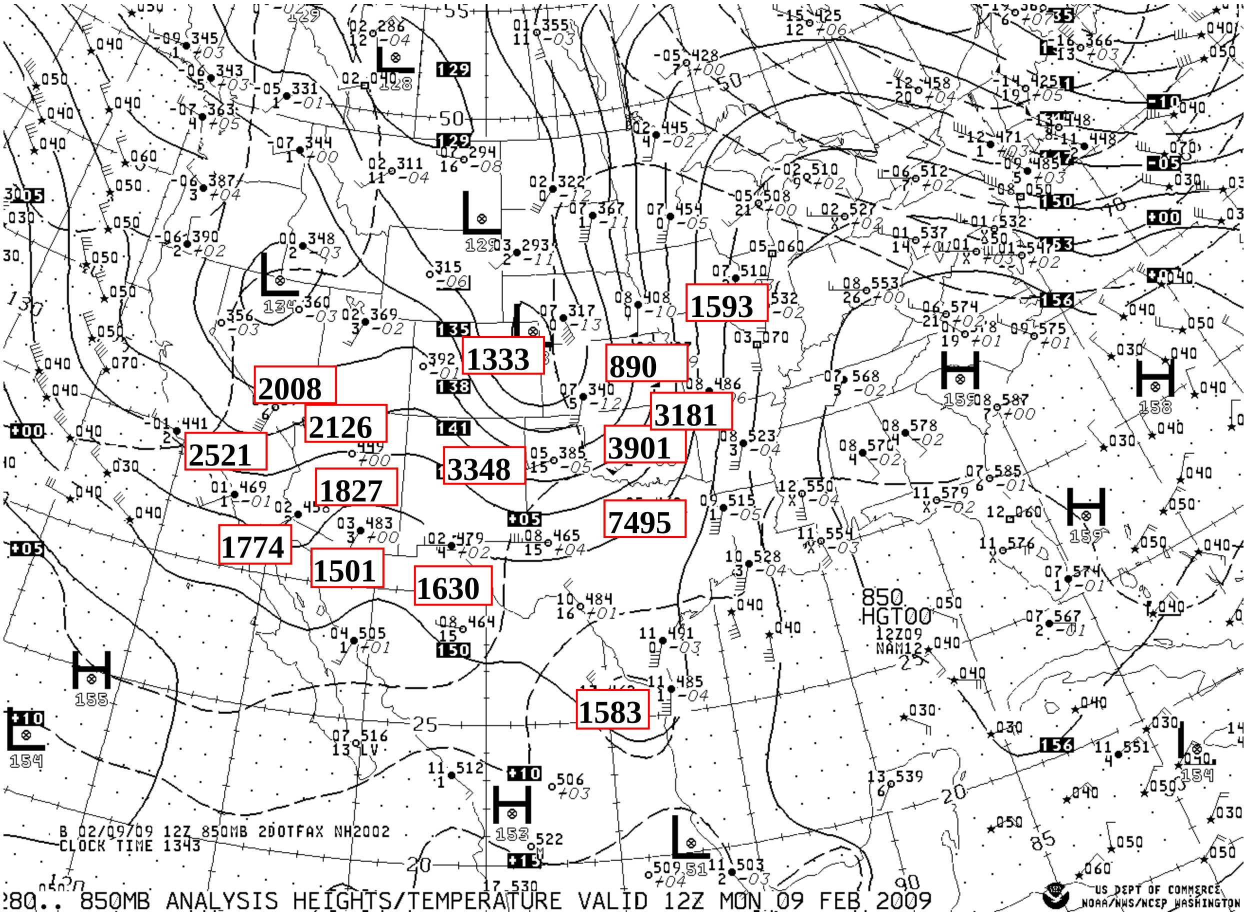

Figure F09-f2-Used the 02/08/2009 week-ending Physician influenza-like illness report data, with the Feb 09 2009 12 z 850 mb chart. (Courtesy of NOAA NWS; Google Flu Trends).

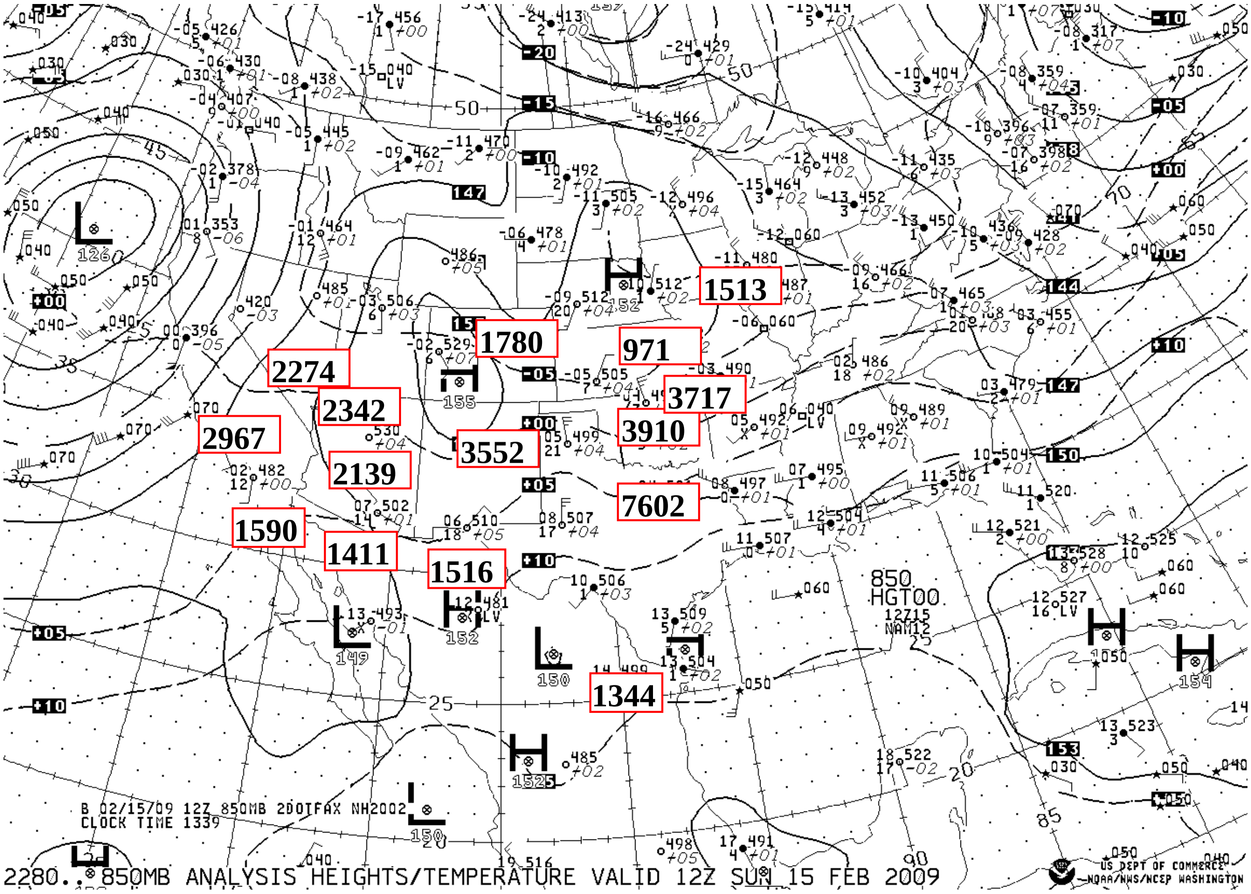

Figure F09-g-Used the 02/15/2009 week-ending Physician influenza-like illness report data, with the Feb 15 2009 12 z 850 mb chart. (Courtesy of NOAA NWS; Google Flu Trends).

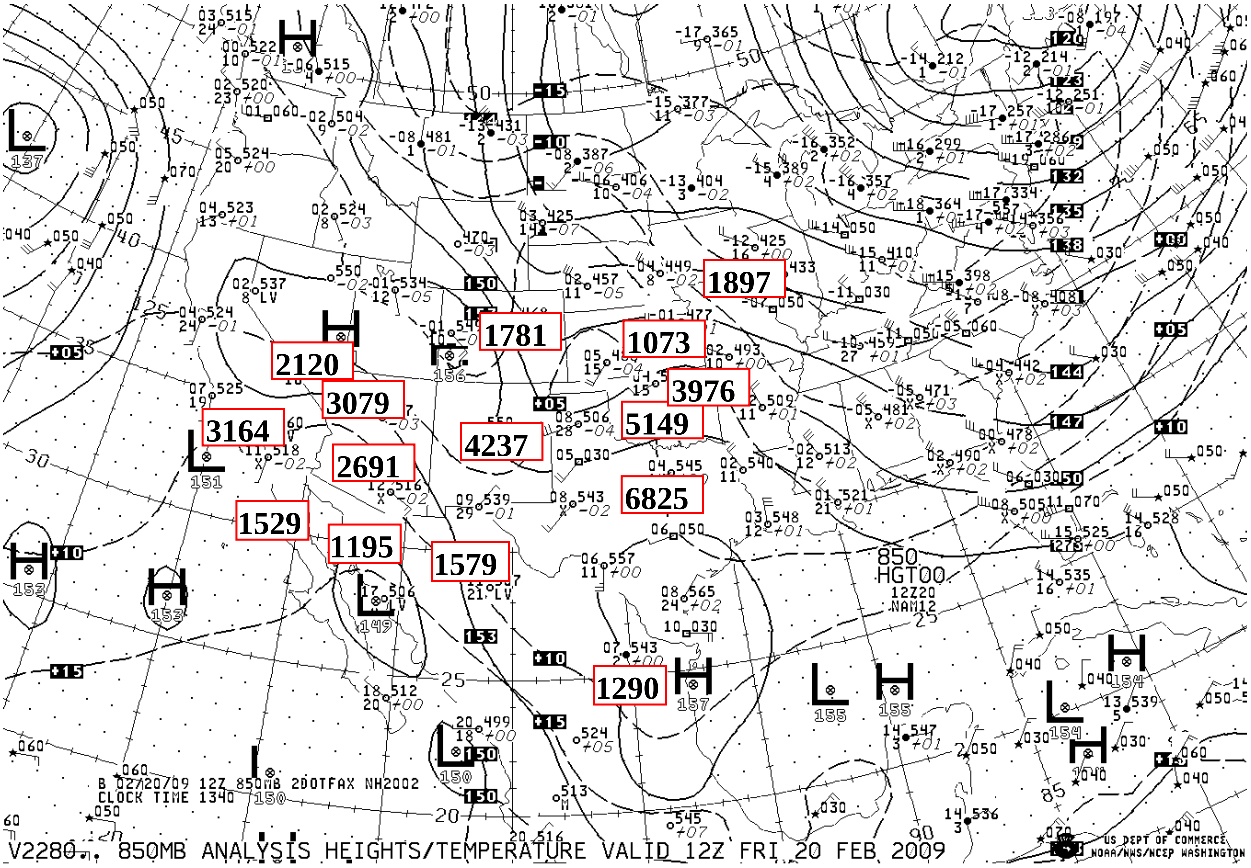

Figure F09-h1-Used the 02/22/2009 week-ending Physician influenza-like illness report data, with the Feb 20 2009 12 z 850 mb chart. (Courtesy of NOAA NWS; Google Flu Trends).

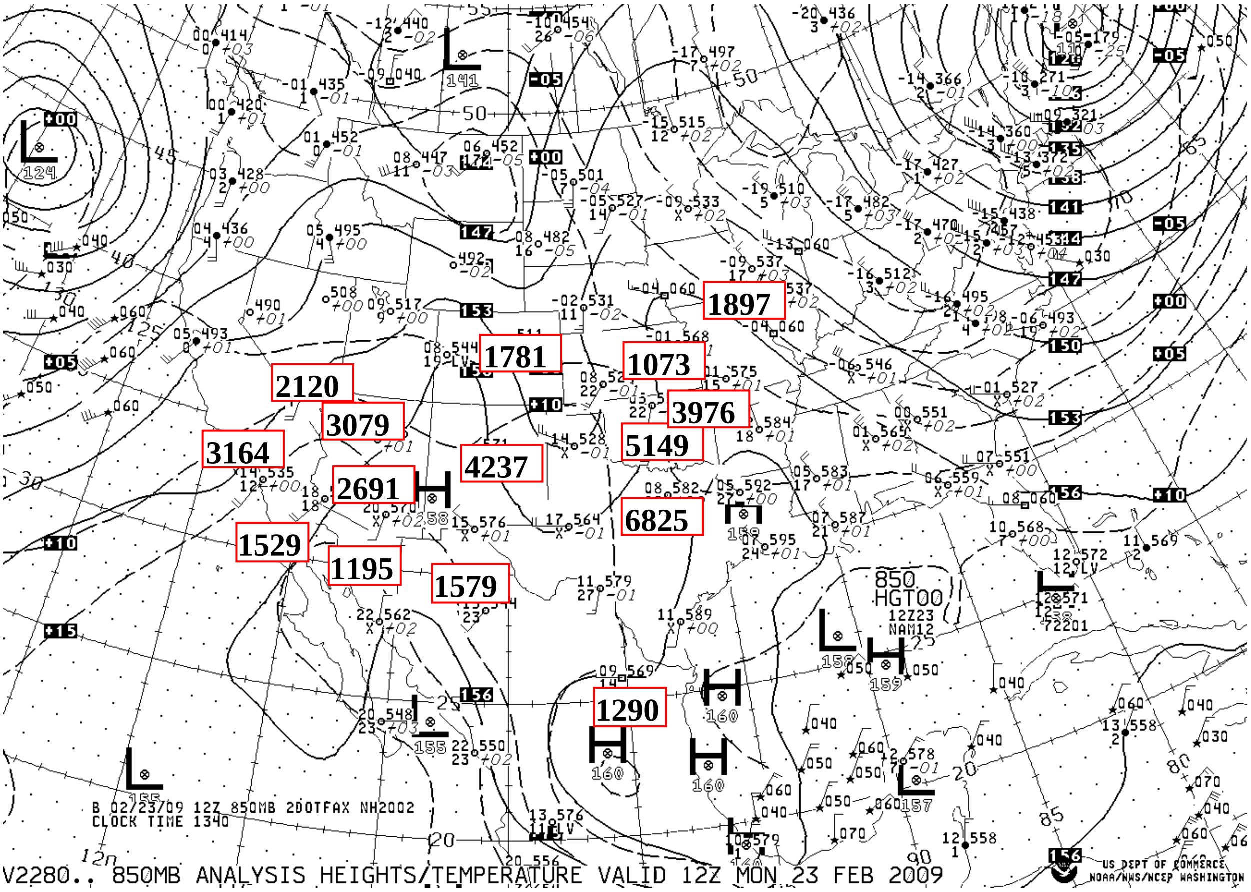

Figure F09-h2-Used the 02/22/2009 week-ending Physician influenza-like illness report data, with the Feb 23 2009 12 z 850 mb chart. (Courtesy of NOAA NWS; Google Flu Trends).

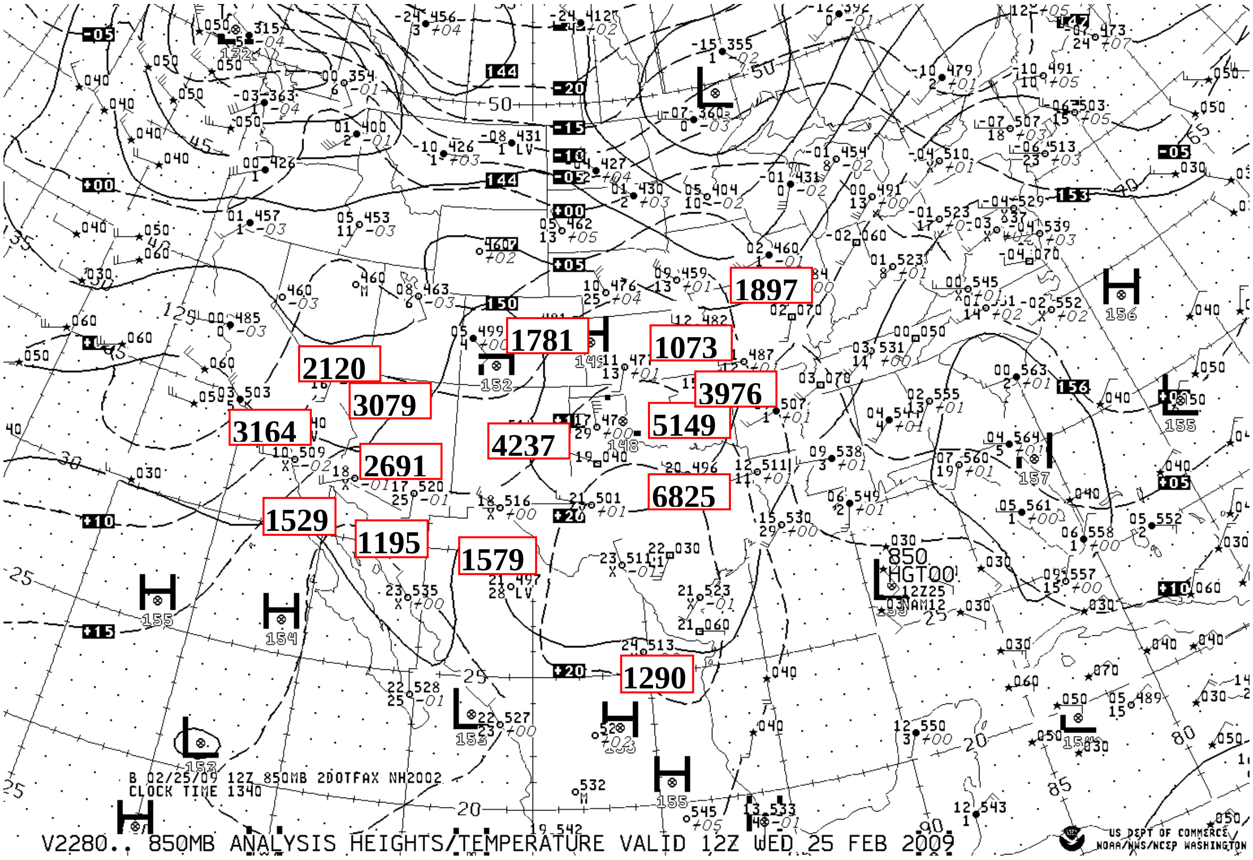

Figure F09-h3-Used the 02/22/2009 week-ending Physician influenza-like illness report data, with the Feb 25 2009 12 z 850 mb chart. (Courtesy of NOAA NWS; Google Flu Trends).

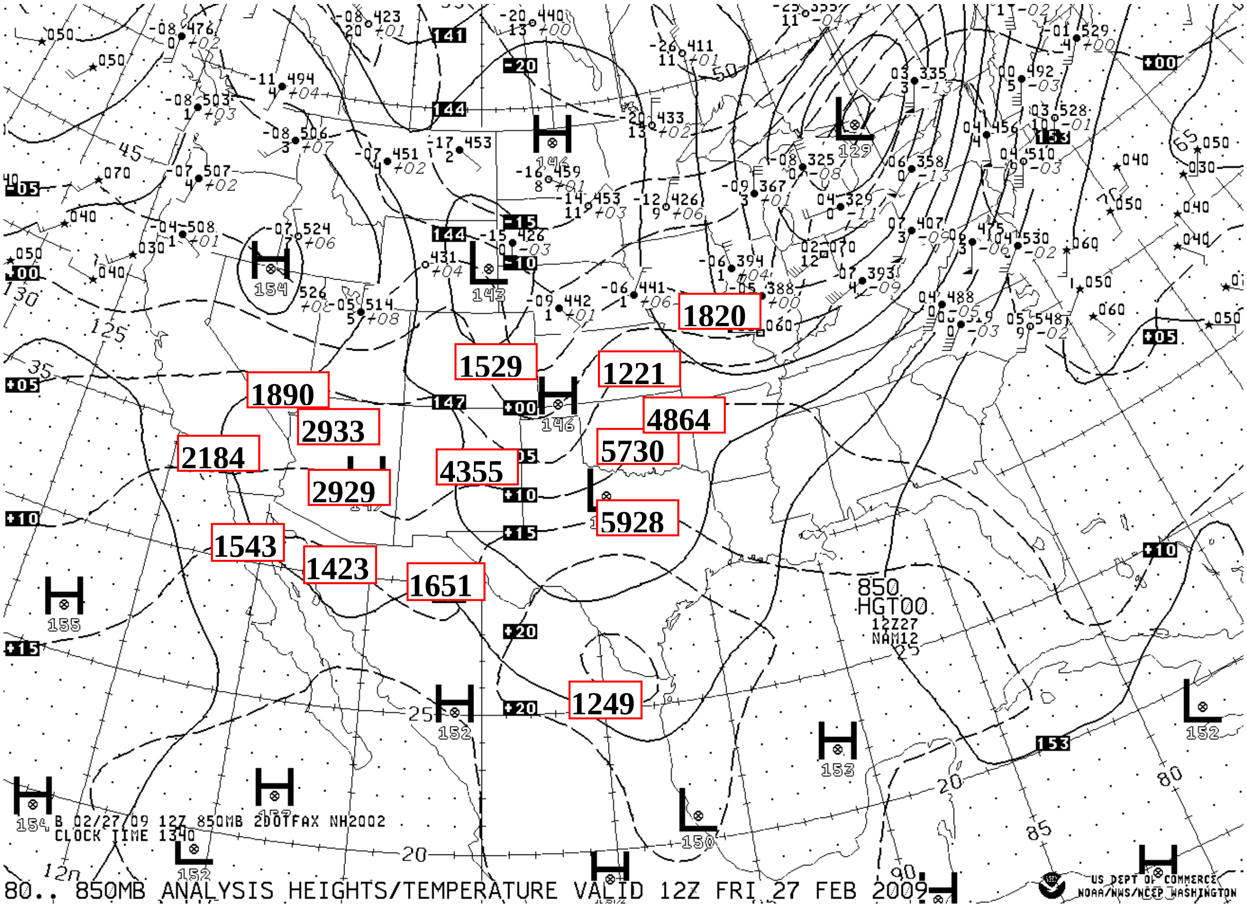

Figure F09-i1-Used the 03/01/2009 week-ending Physician influenza-like illness report data, with the Feb 27 2009 12 z 850 mb chart. (Courtesy of NOAA NWS; Google Flu Trends).

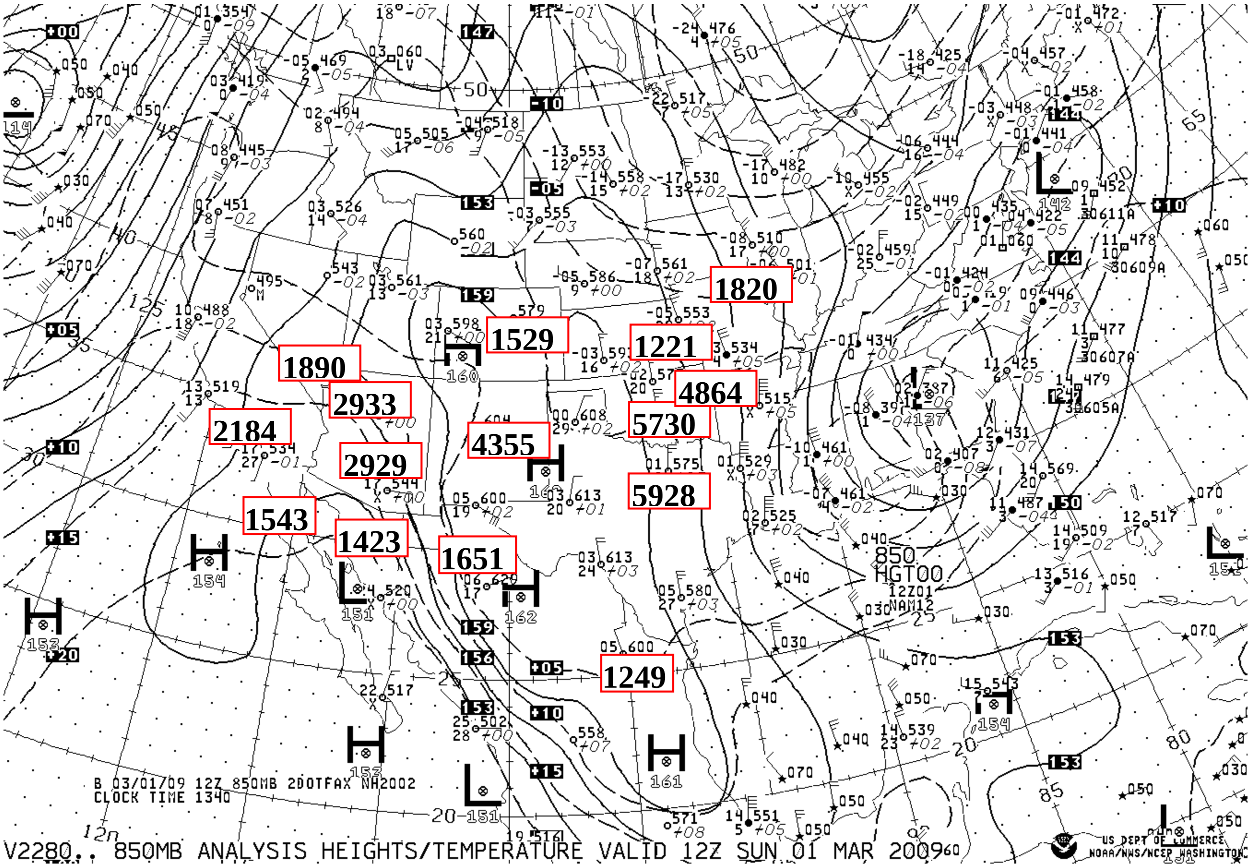

Figure F09-i2-Used the 03/01/2009 week-ending Physician influenza-like illness report data, with the Mar 01 2009 12 z 850 mb chart. (Courtesy of NOAA NWS; Google Flu Trends).

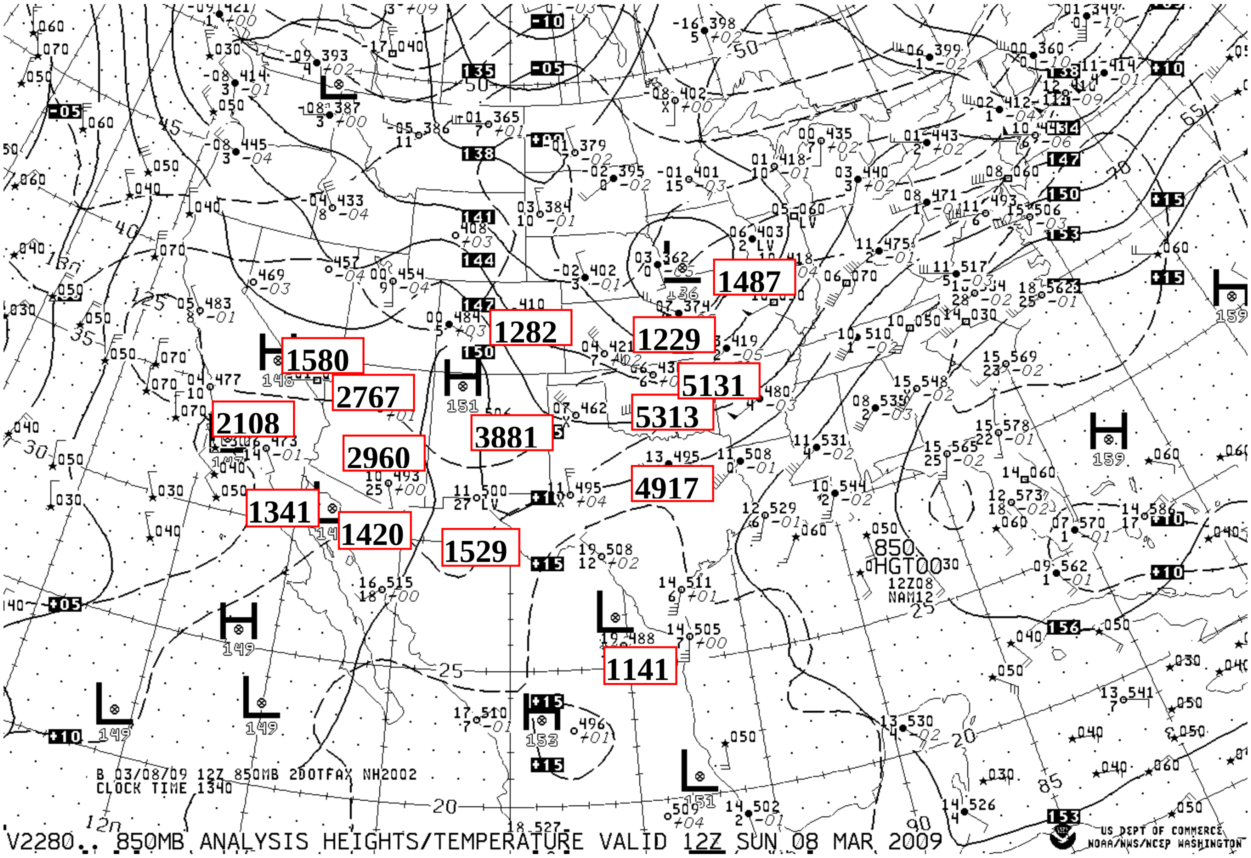

Figure F09-j1-Used the 03/08/2009 week-ending Physician influenza-like illness report data, with the Mar 08 2009 12 z 850 mb chart. (Courtesy of NOAA NWS; Google Flu Trends).

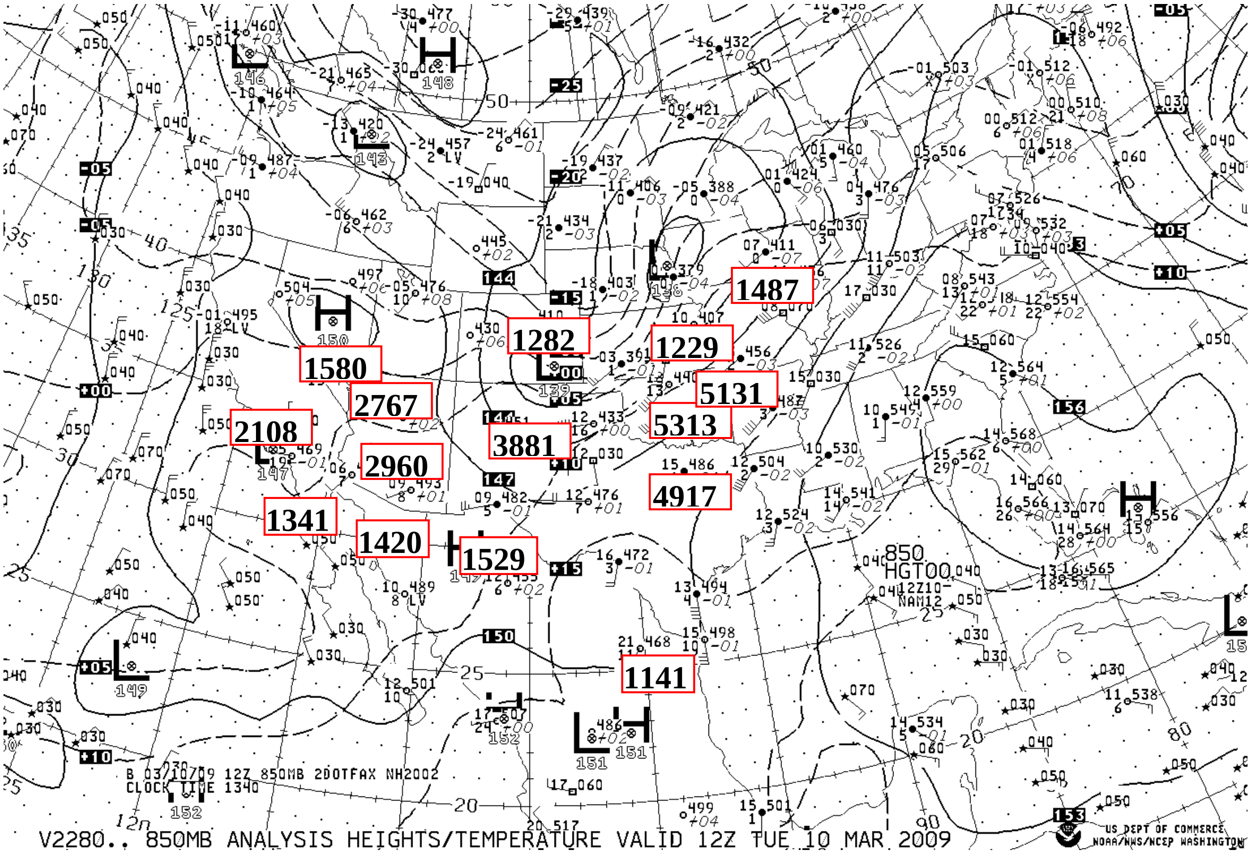

Figure F09-j2-Used the 03/08/2009 week-ending Physician influenza-like illness report data, with the Mar 10 2009 12 z 850 mb chart. (Courtesy of NOAA NWS; Google Flu Trends).

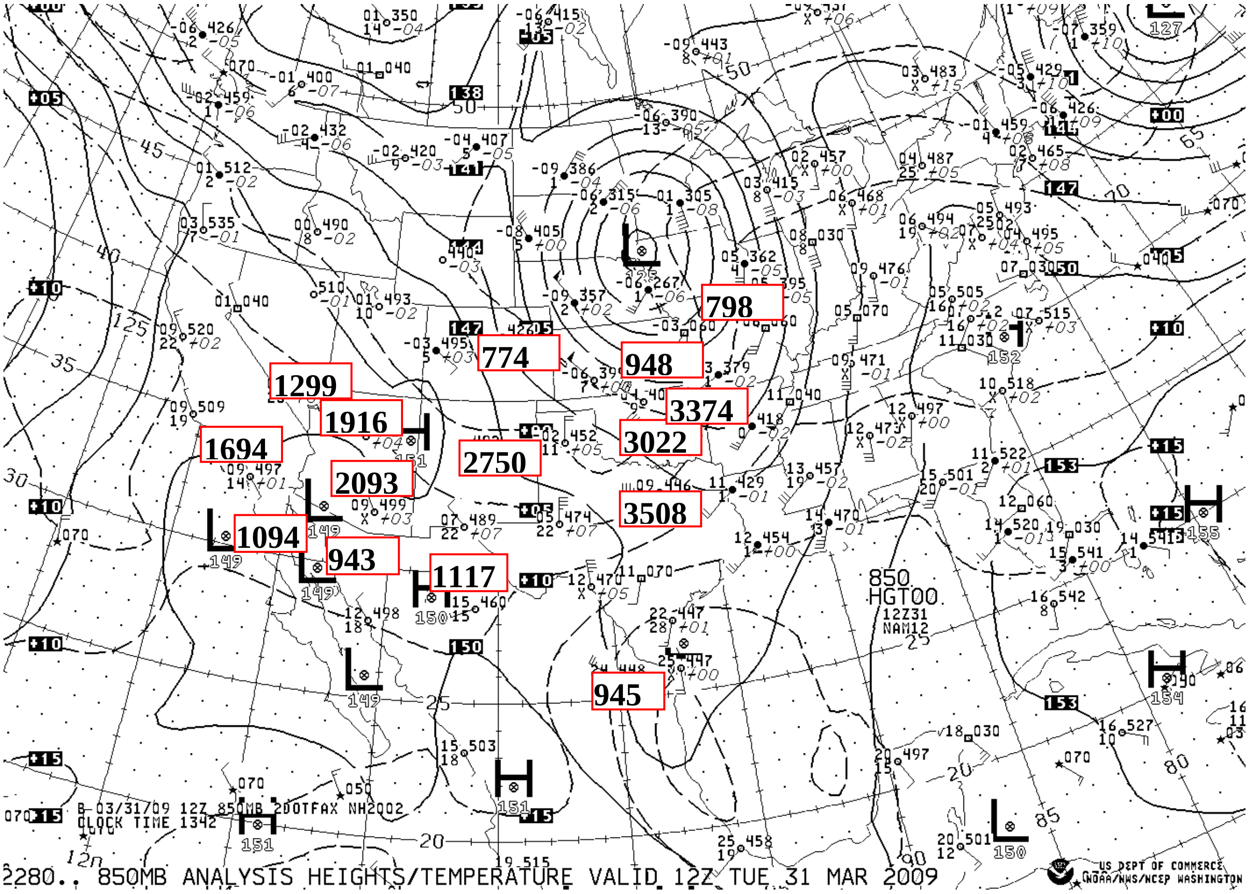

Figure F09-k1-Used the 03/29/2009 week-ending Physician influenza-like illness report data, with the Mar 31 2009 12 z 850 mb chart. (Courtesy of NOAA NWS; Google Flu Trends).

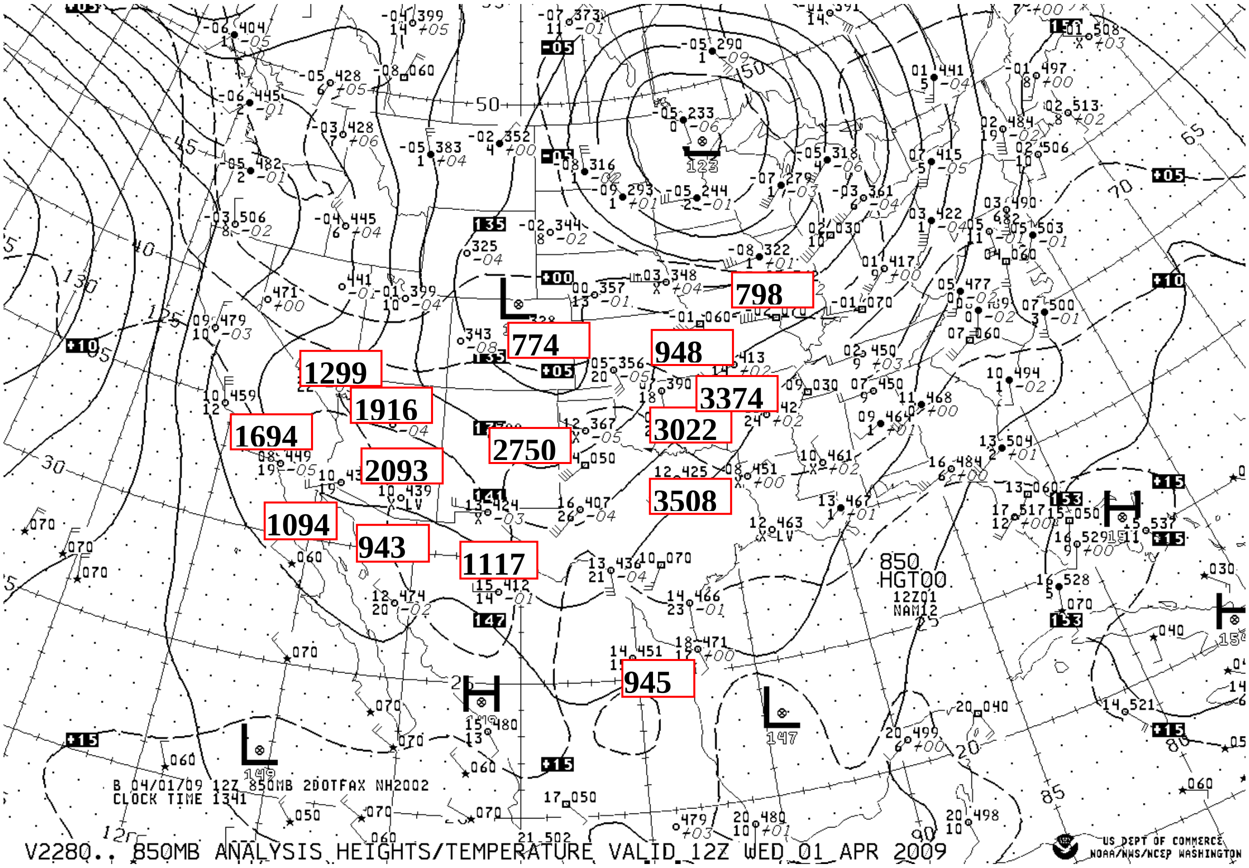

Figure F09-k2-Used the 03/29/2009 week-ending Physician influenza-like illness report data, with the Apr 01 2009 12 z 850 mb chart. (Courtesy of NOAA NWS; Google Flu Trends).

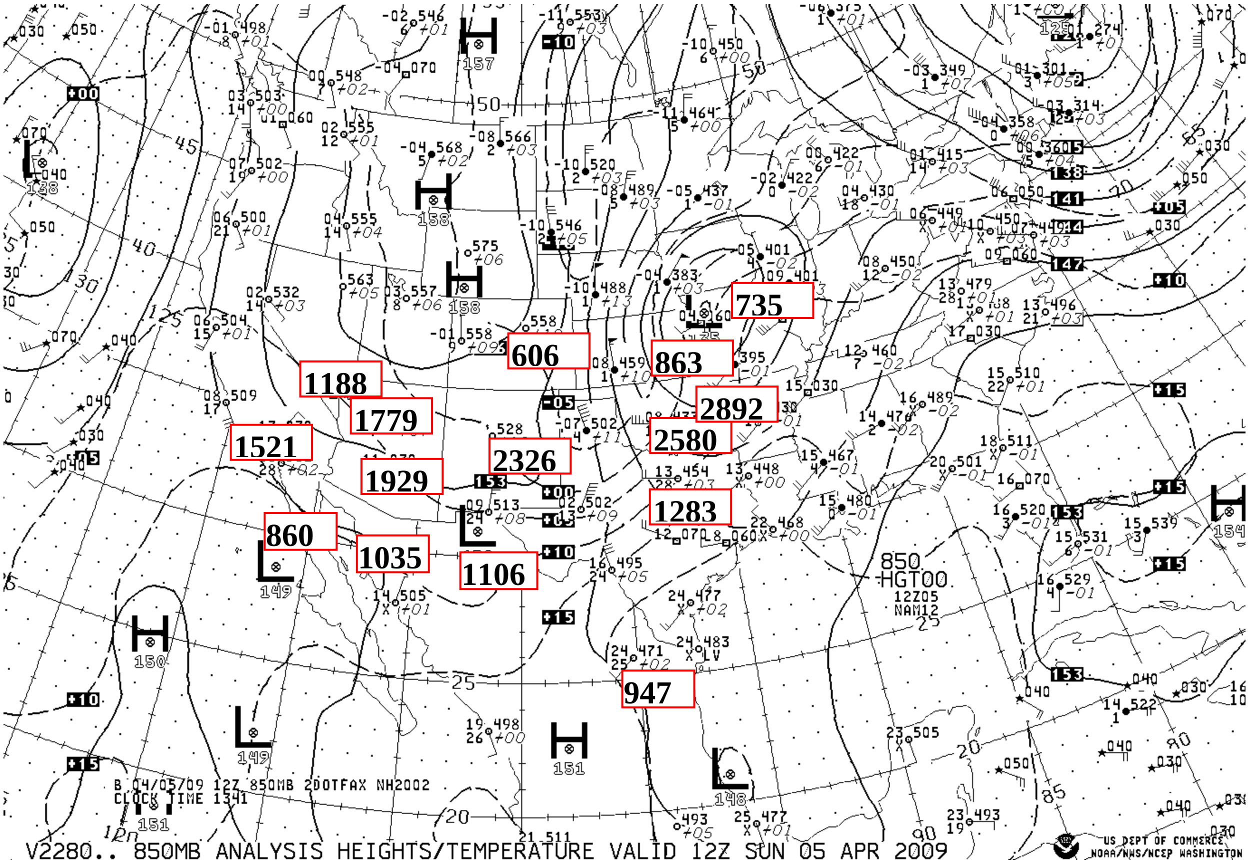

Figure F09-l-Used the 04/05/2009 week-ending Physician influenza-like illness report data, with the Apr 05 2009 12 z 850 mb chart. (Courtesy of NOAA NWS; Google Flu Trends).

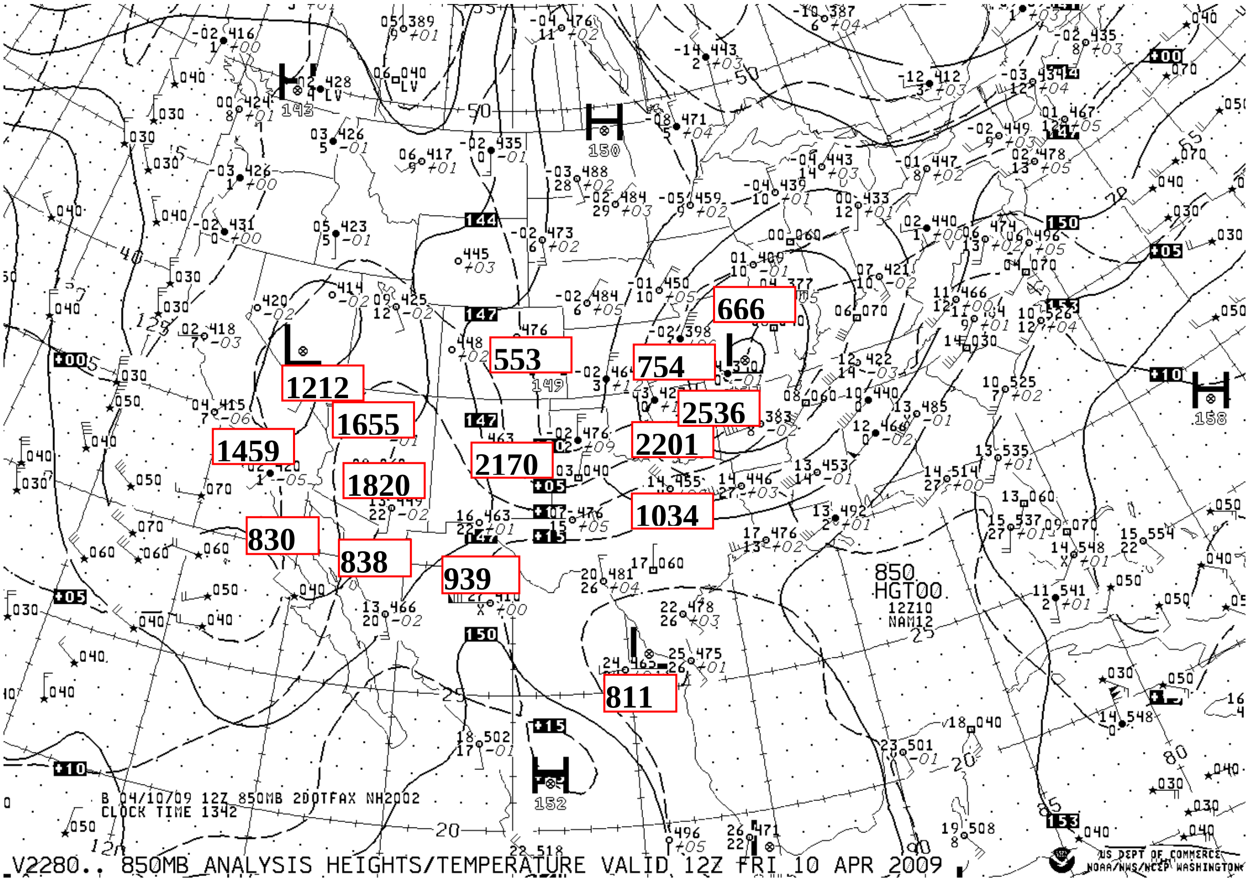

Figure F09-m-Used the 04/12/2009 week-ending Physician influenza-like illness report data, with the Apr 10 2009 12 z 850 mb chart. (Courtesy of NOAA NWS; Google Flu Trends).

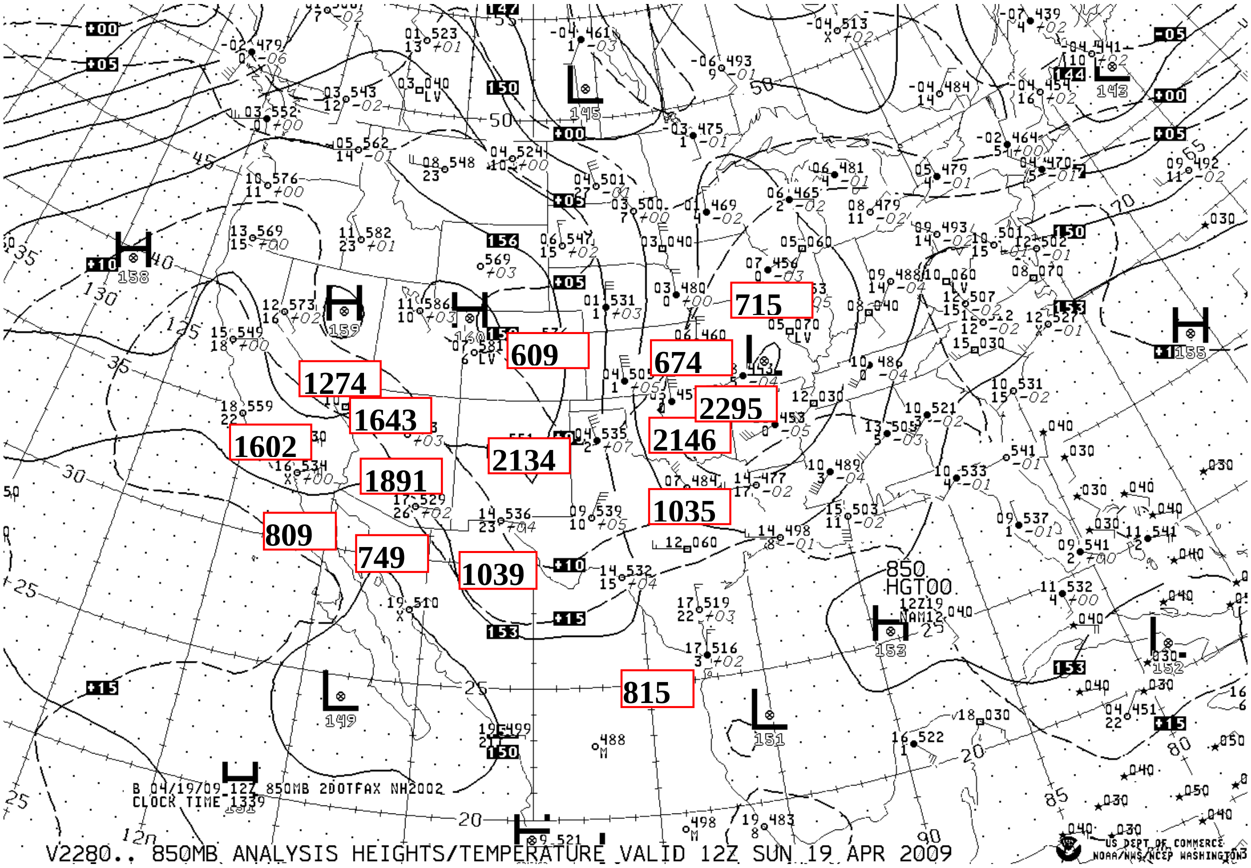

Figure F09-n-Used the 04/19/2009 week-ending Physician influenza-like illness report data, with the Apr 19 2009 12 z 850 mb chart. (Courtesy of NOAA NWS; Google Flu Trends).

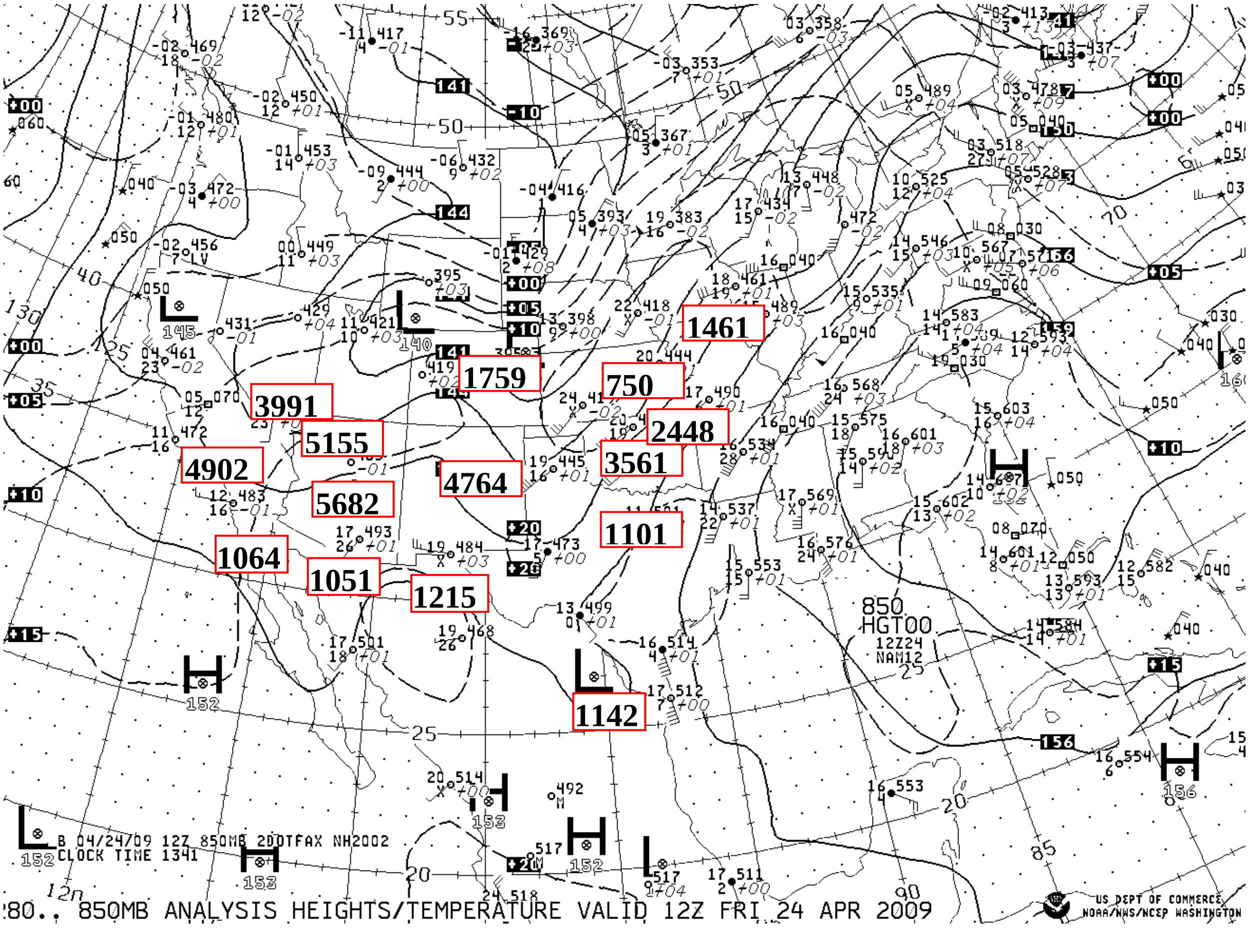

Figure F09-o1-Used the 04/26/2009 week-ending Physician influenza-like illness report data, with the Apr 24 2009 12 z 850 mb chart. (Courtesy of NOAA NWS; Google Flu Trends).

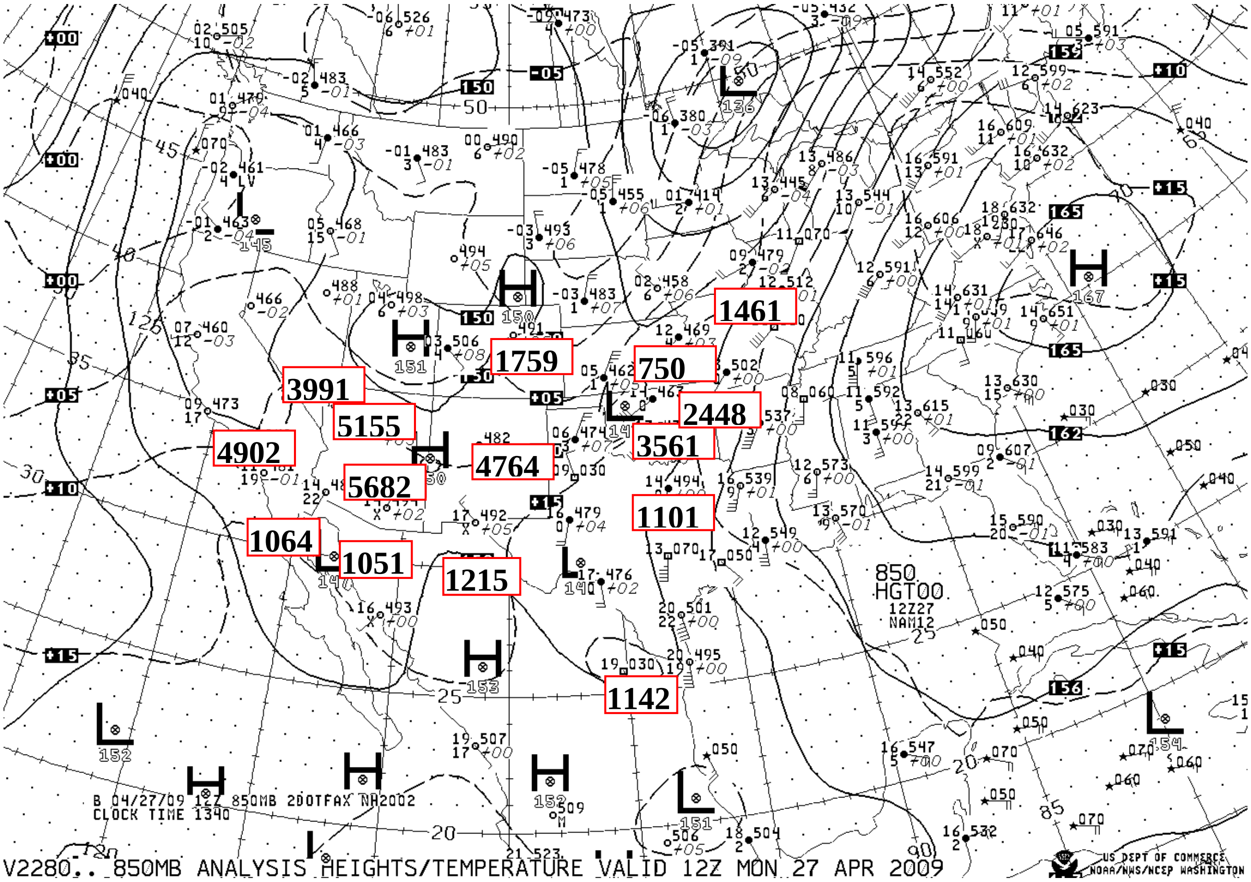

Figure F09-o2-Used the 04/26/2009 week-ending Physician influenza-like illness report data, with the Apr 27 2009 12 z 850 mb chart. (Courtesy of NOAA NWS; Google Flu Trends).

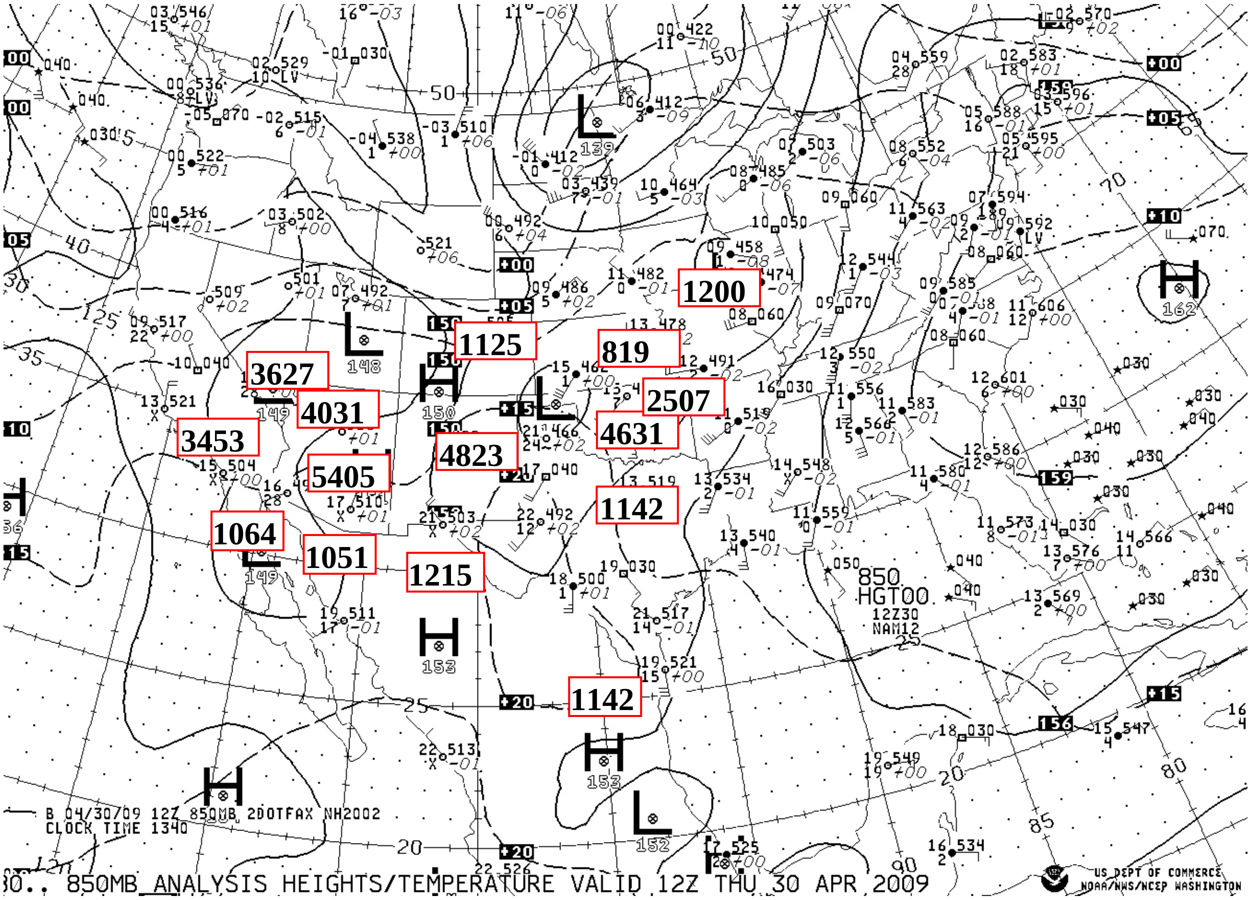

Figure F09-p1-Used the 05/03/2009 week-ending Physician influenza-like illness report data, with the Apr 30 2009 12 z 850 mb chart. (Courtesy of NOAA NWS; Google Flu Trends).

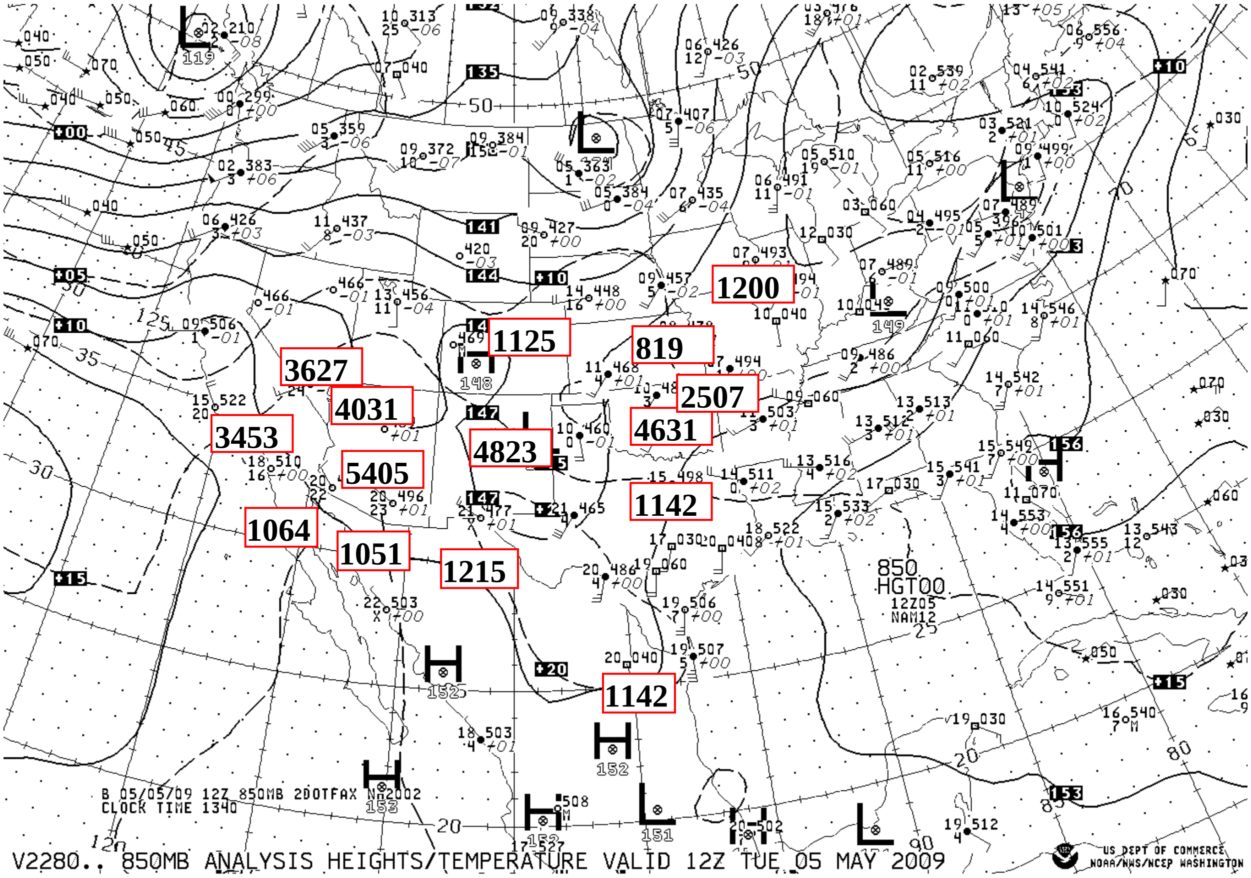

Figure F09-p2-Used the 05/03/2009 week-ending Physician influenza-like illness report data, with the May 05 2009 12 z 850 mb chart. (Courtesy of NOAA NWS; Google Flu Trends).

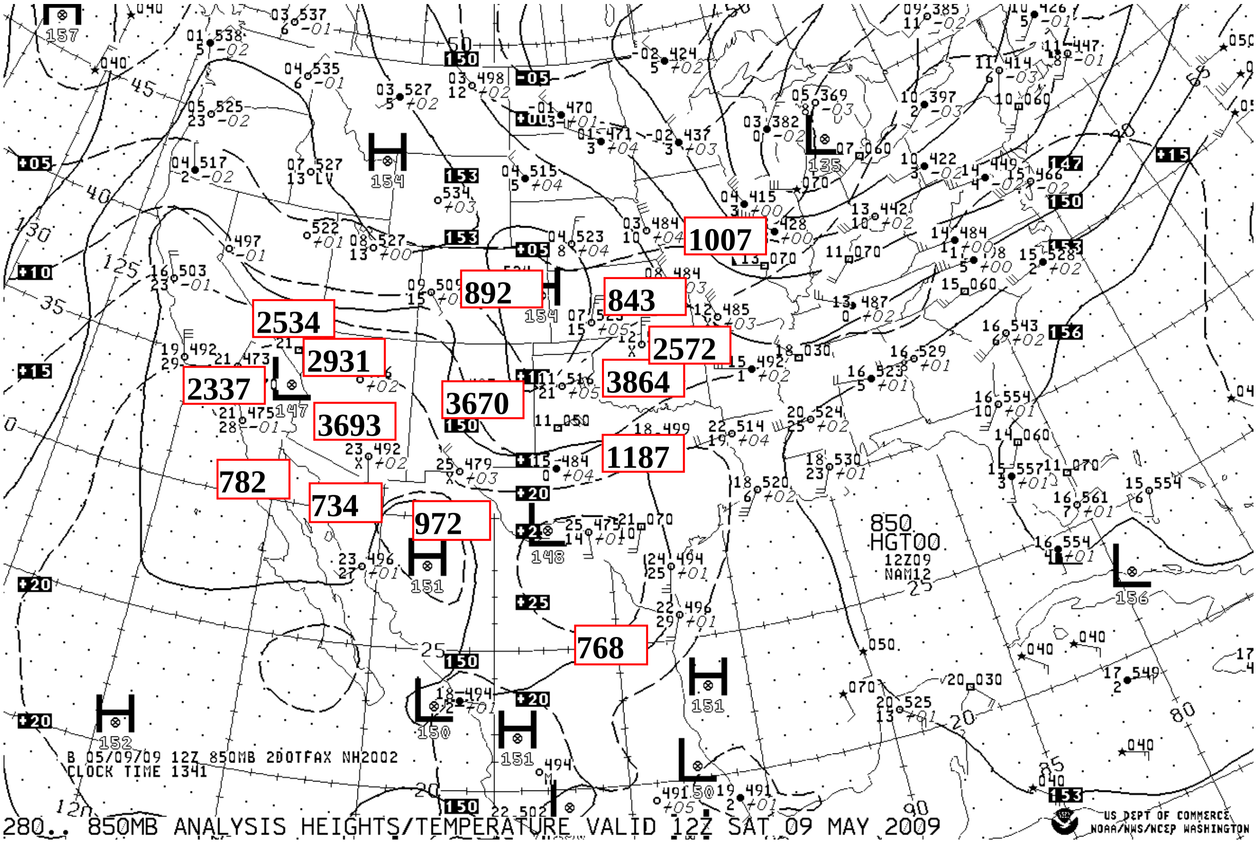

Figure F09-q1-Used the 05/10/2009 week-ending Physician influenza-like illness report data, with the May 09 2009 12 z 850 mb chart. (Courtesy of NOAA NWS; Google Flu Trends).

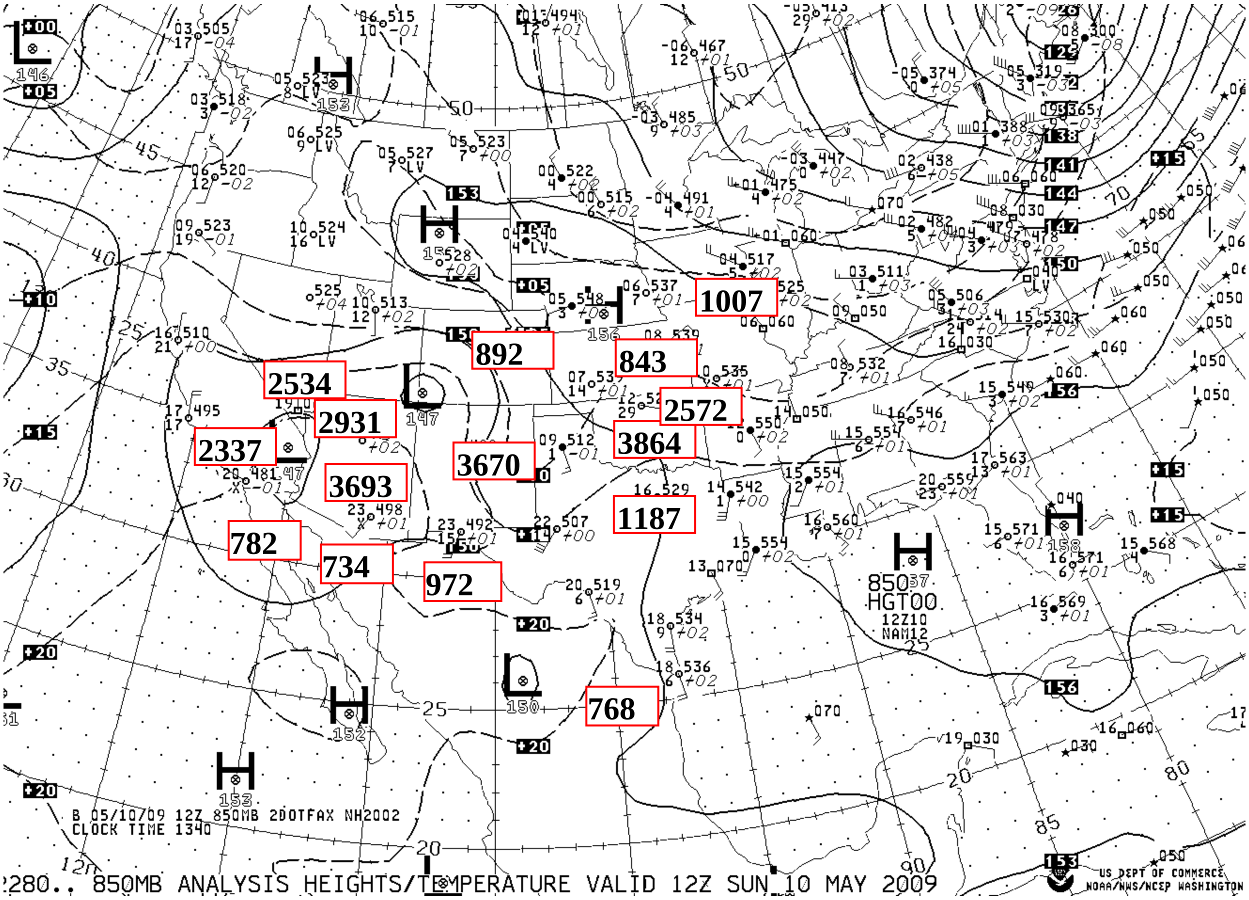

Figure F09-q2-Used the 05/10/2009 week-ending Physician influenza-like illness report data, with the May 10 2009 12 z 850 mb chart. (Courtesy of NOAA NWS; Google Flu Trends).

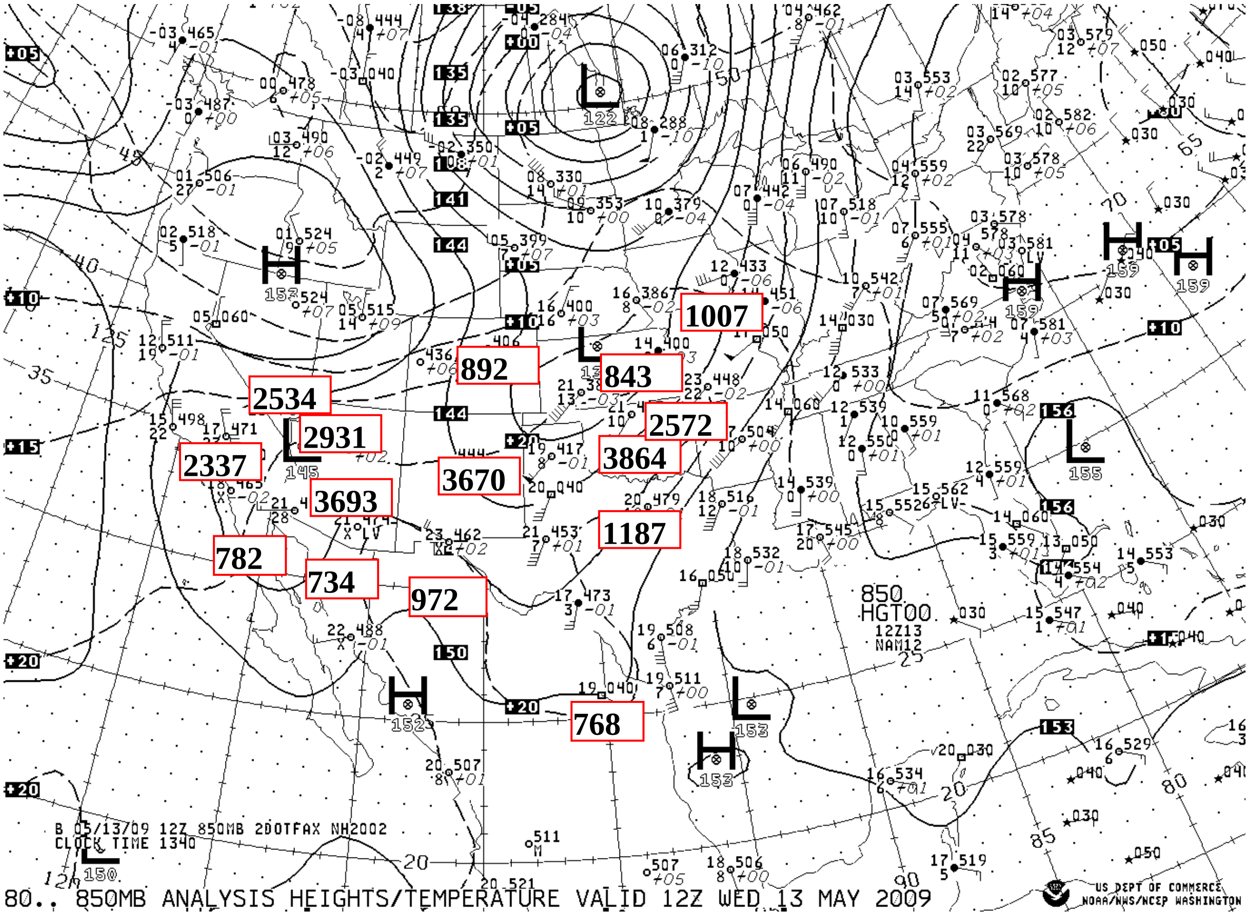

Figure F09-q3-Used the 05/10/2009 week-ending Physician influenza-like illness report data, with the May 13 2009 12 z 850 mb chart. (Courtesy of NOAA NWS; Google Flu Trends).

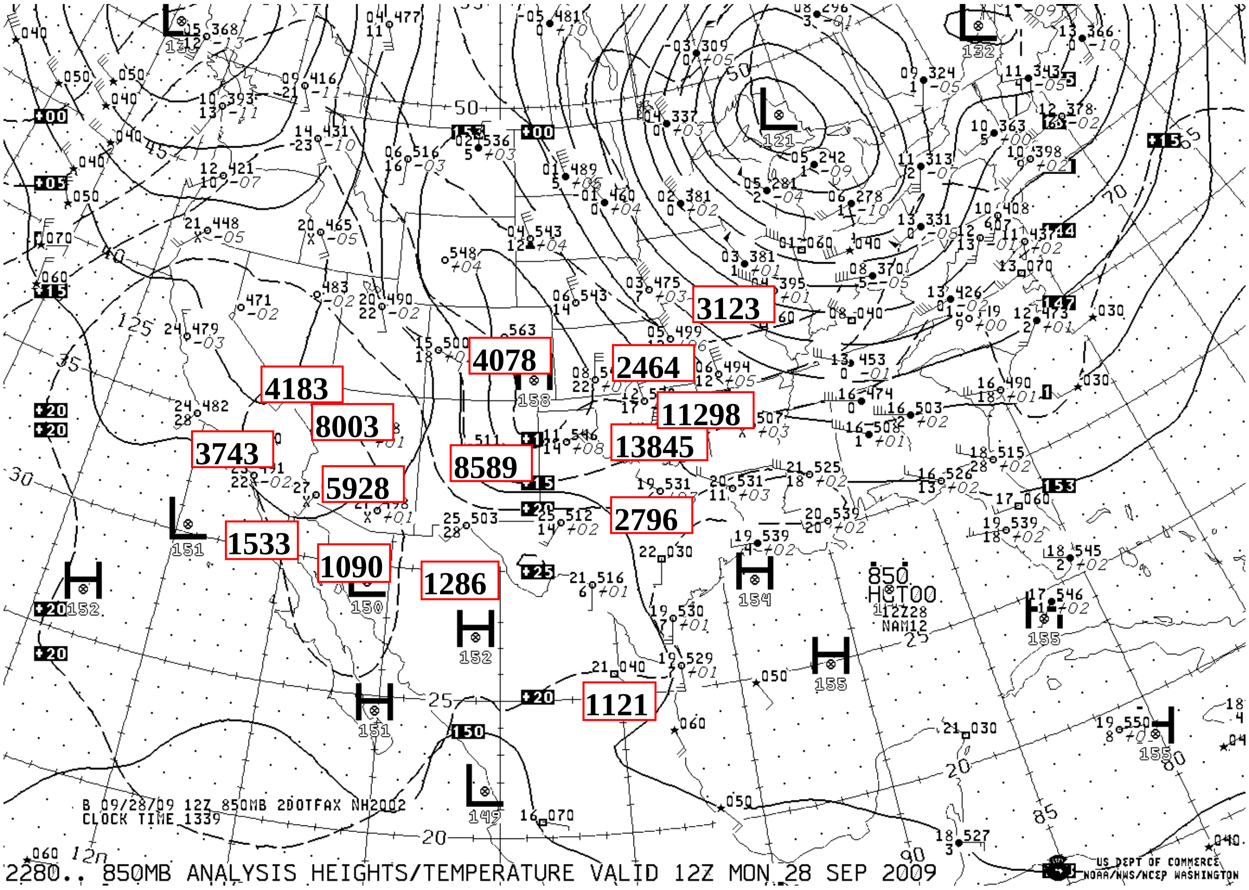

Figure 09G-a-Used the 09/27/2009 week-ending Physician influenza-like illness report data, with the Sep 28 2009 12 z 850 mb chart. (Courtesy of NOAA NWS; Google Flu Trends).

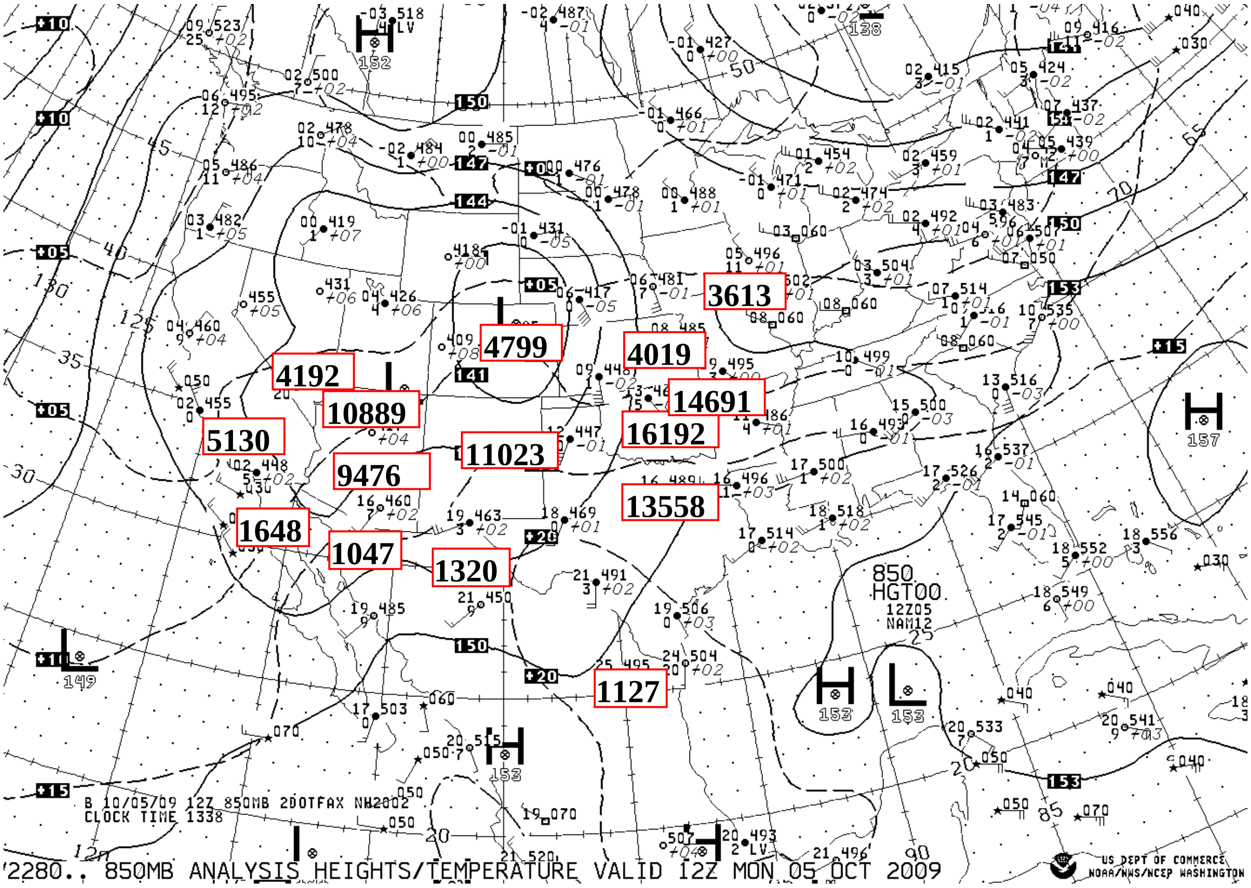

Figure 09G-b-Used the 10/04/2009 week-ending Physician influenza-like illness report data, with the Oct 05 2009 12 z 850 mb chart. (Courtesy of NOAA NWS; Google Flu Trends).

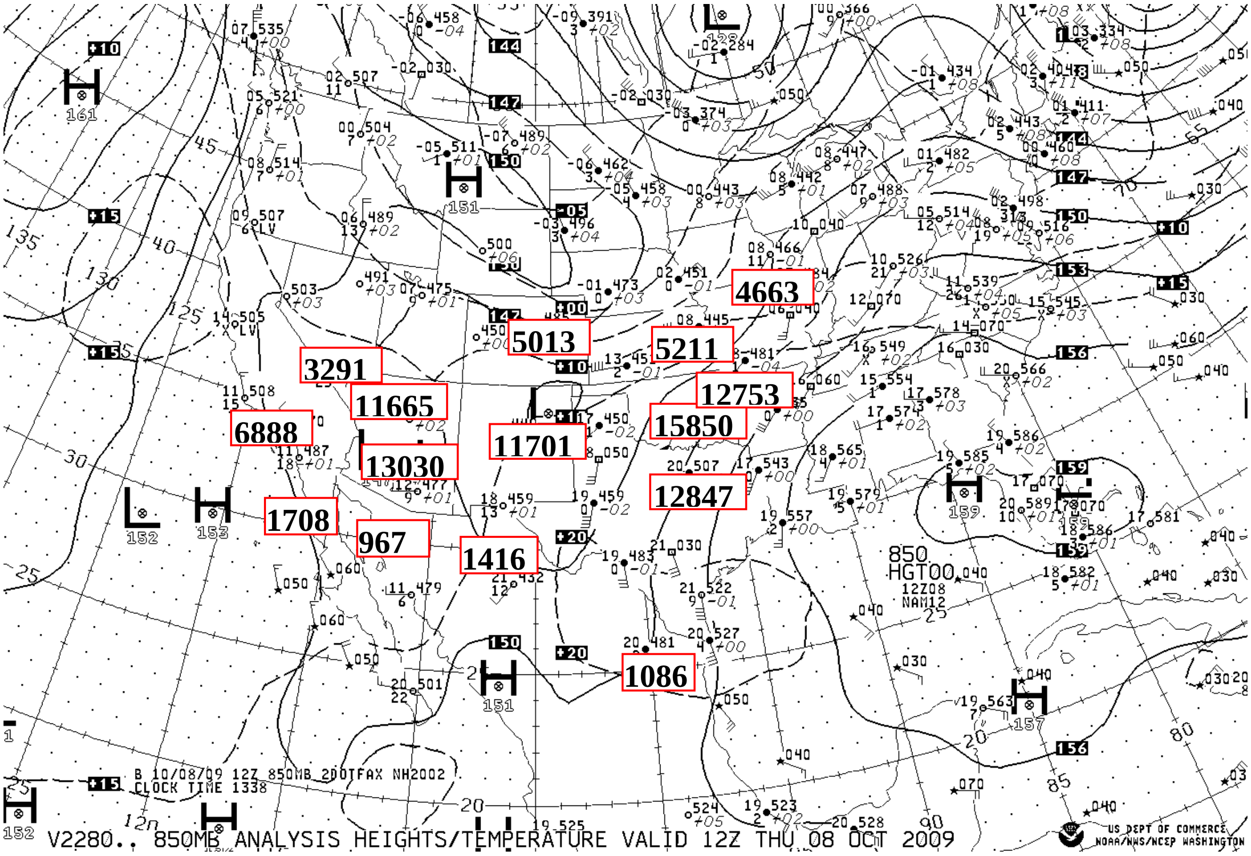

Figure 09G-c1-Used the 10/11/2009 week-ending Physician influenza-like illness report data, with the Oct 08 2009 12 z 850 mb chart. (Courtesy of NOAA NWS; Google Flu Trends).

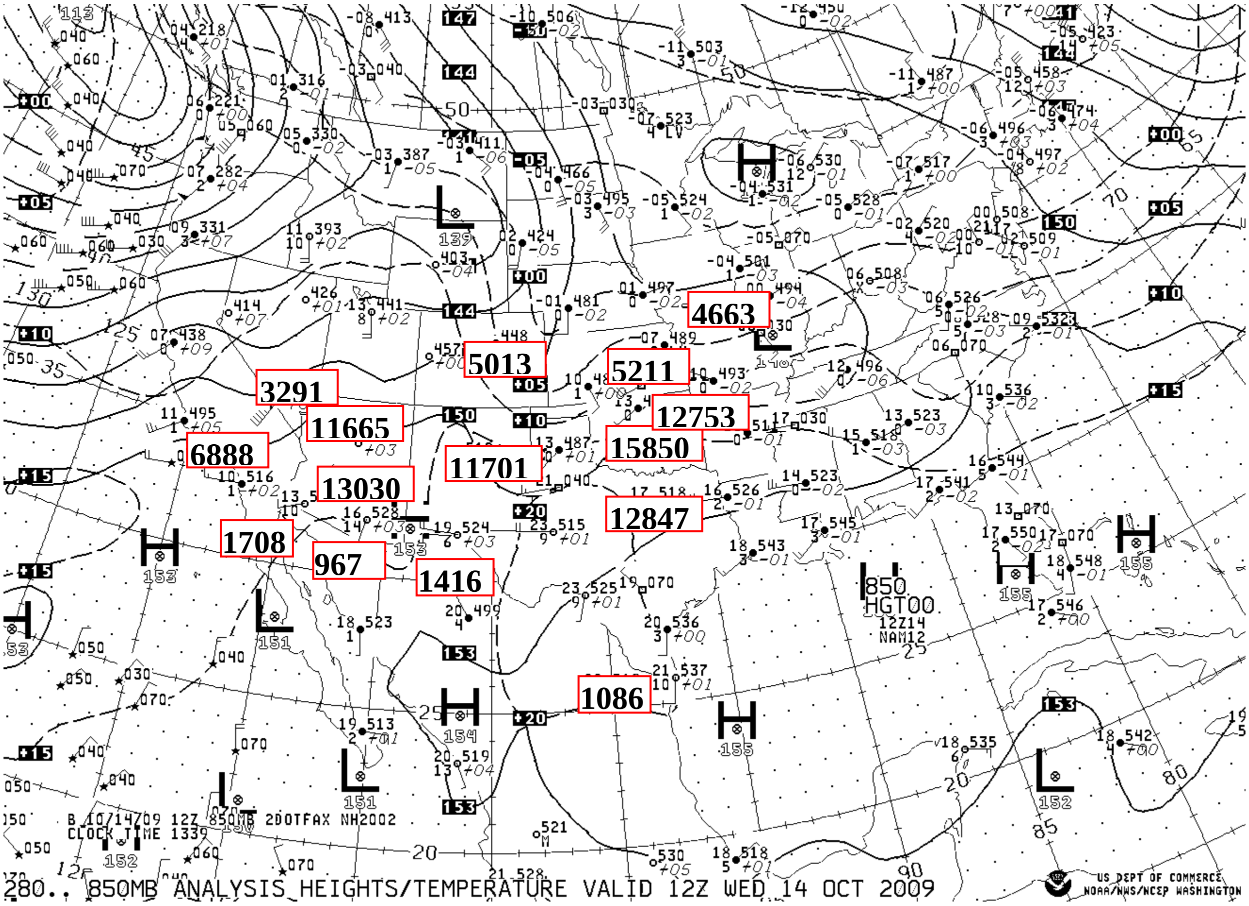

Figure 09G-c2-Used the 10/11/2009 week-ending Physician influenza-like illness report data, with the Oct 14 2009 12 z 850 mb chart. (Courtesy of NOAA NWS; Google Flu Trends).

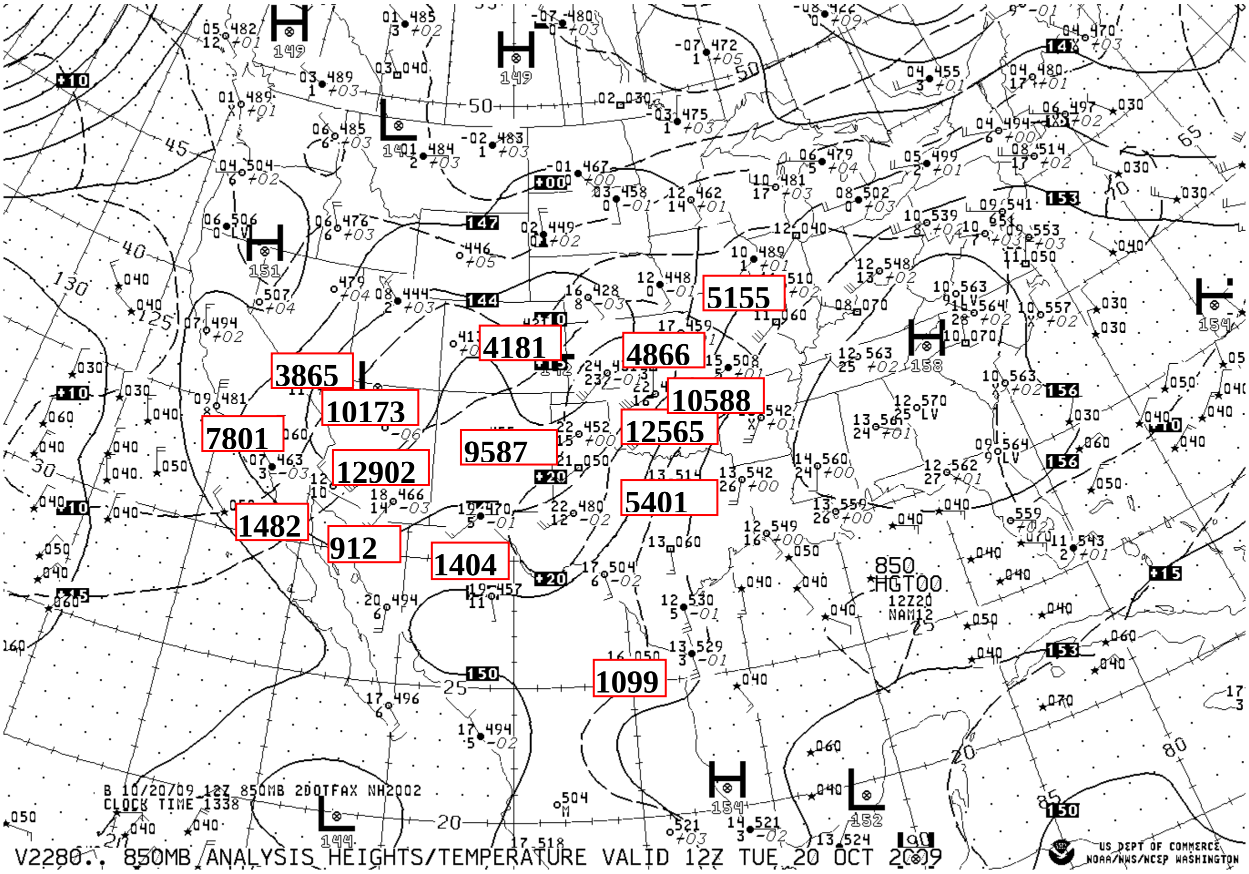

Figure 09G-d-Used the 10/18/2009 week-ending Physician influenza-like illness report data, with the Oct 20 2009 12 z 850 mb chart. (Courtesy of NOAA NWS; Google Flu Trends).

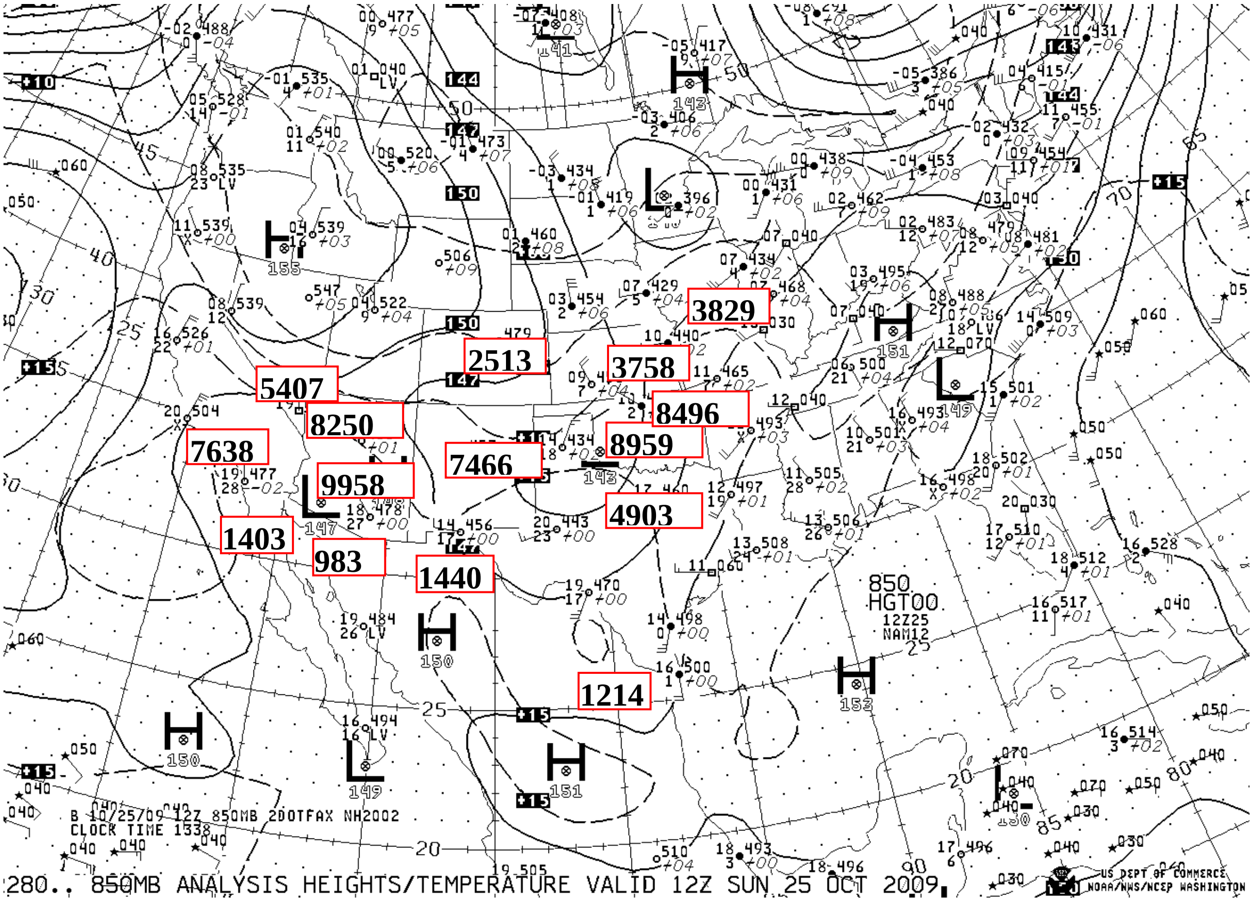

Figure 09G-e-Used the 10/25/2009 week-ending Physician influenza-like illness report data, with the Oct 25 2009 12 z 850 mb chart. (Courtesy of NOAA NWS; Google Flu Trends).

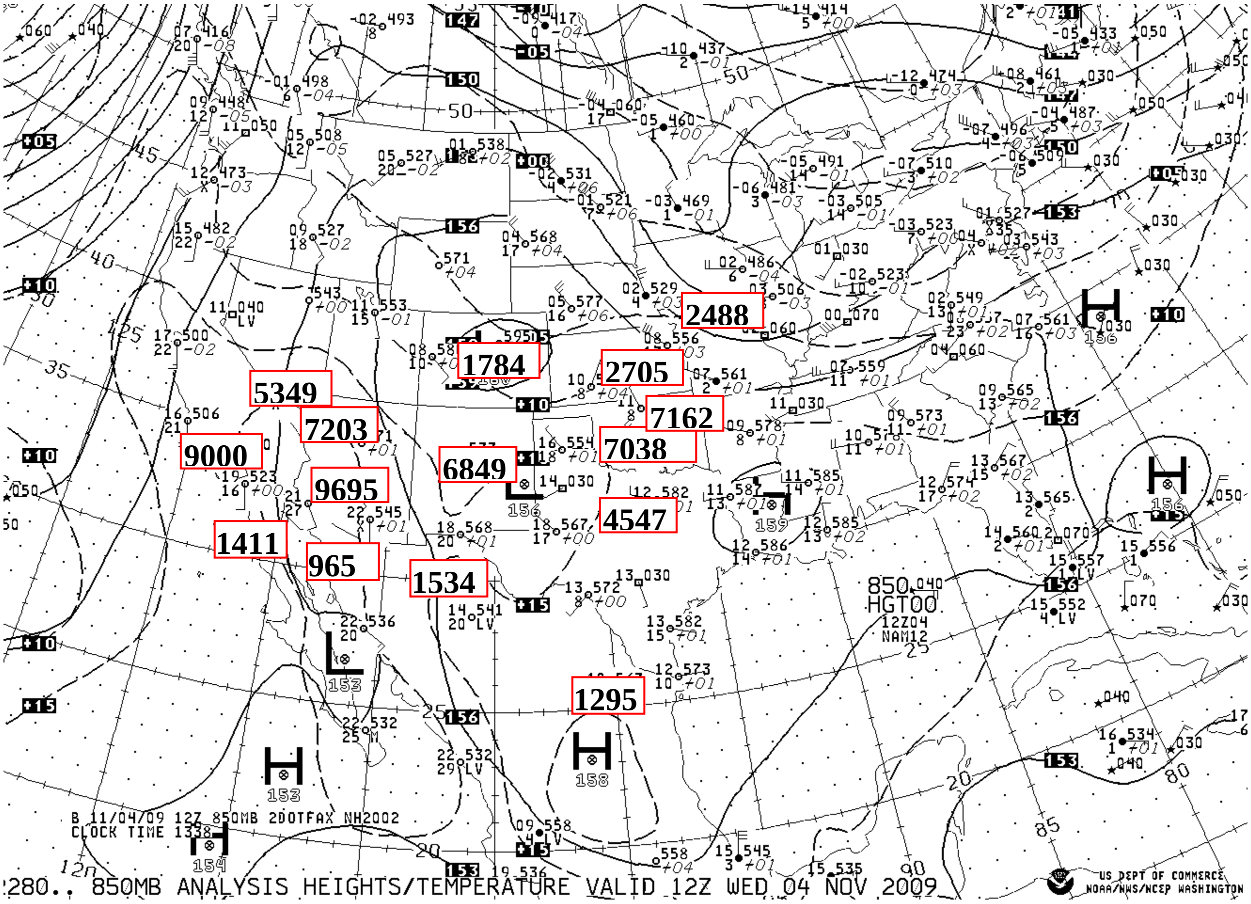

Figure 09G-f-Used the 11/01/2005 week-ending Physician influenza-like illness report data, with the Nov 04 2009 12 z 850 mb chart. (Courtesy of NOAA NWS; Google Flu Trends).

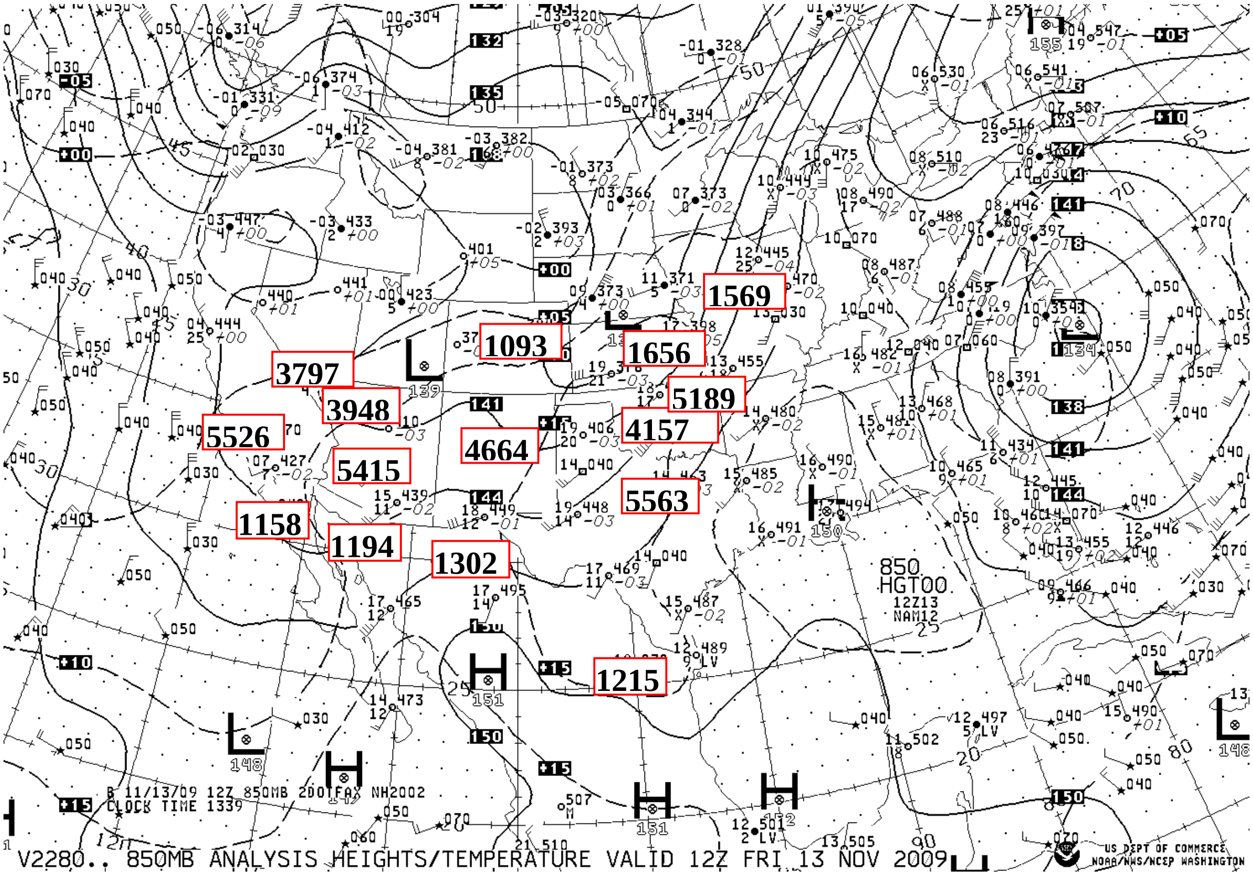

Figure 09G-g-Used the 11/15/2009 week-ending Physician influenza-like illness report data, with the Nov 13 2009 12 z 850 mb chart. (Courtesy of NOAA NWS; Google Flu Trends).

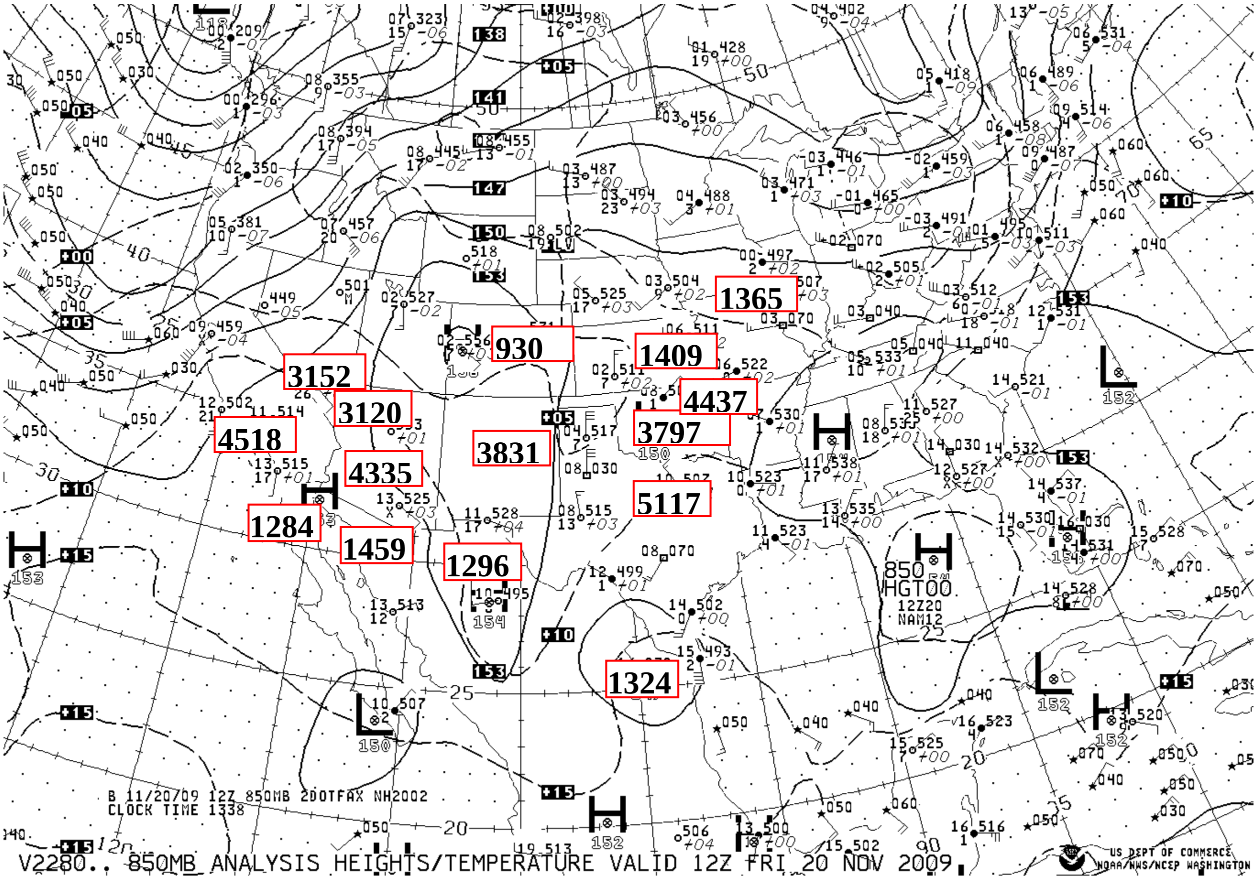

Figure 09G-h1-Used the 11/22/2009 week-ending Physician influenza-like illness report data, with the Nov 20 2009 12 z 850 mb chart. (Courtesy of NOAA NWS; Google Flu Trends).

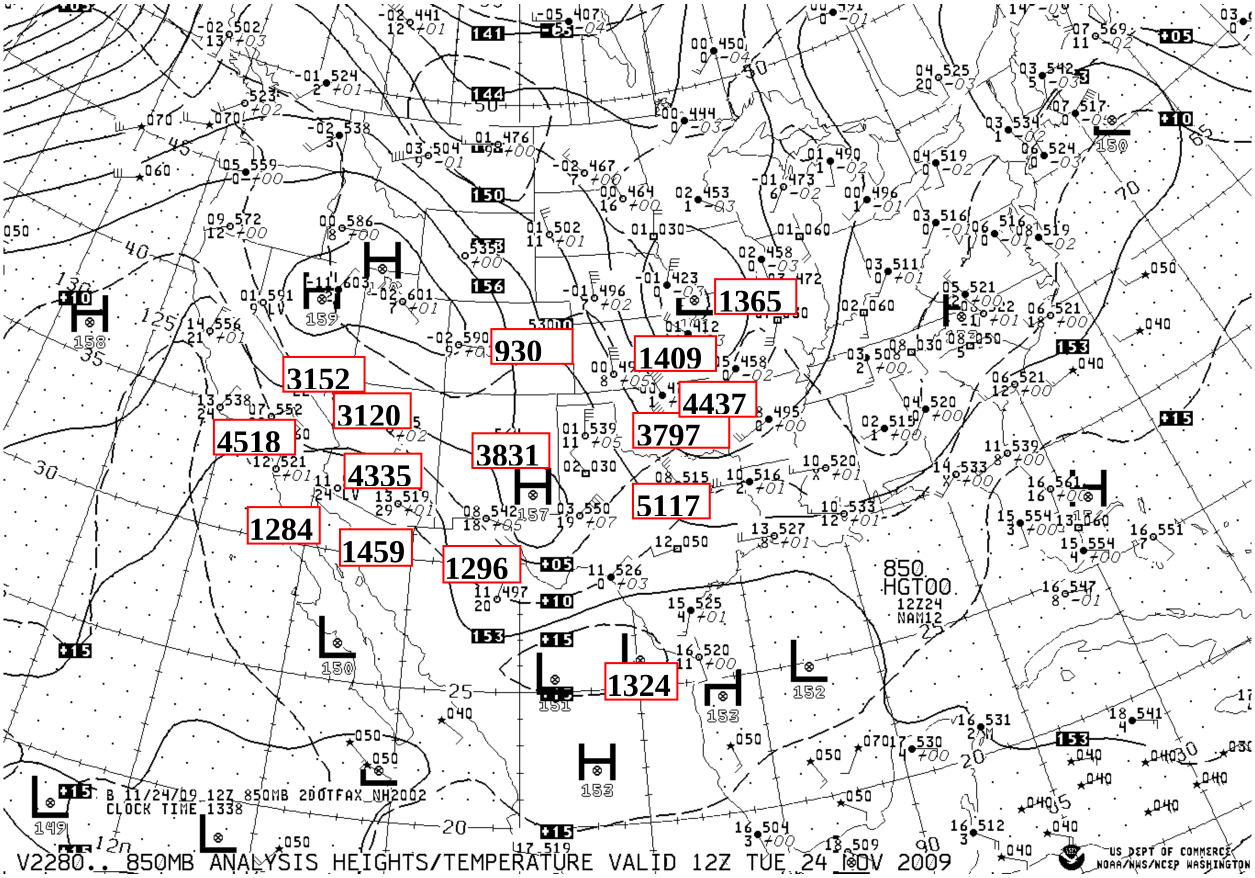

Figure 09G-h2-Used the 11/22/2009 week-ending Physician influenza-like illness report data, with the Nov 24 2009 12 z 850 mb chart. (Courtesy of NOAA NWS; Google Flu Trends).

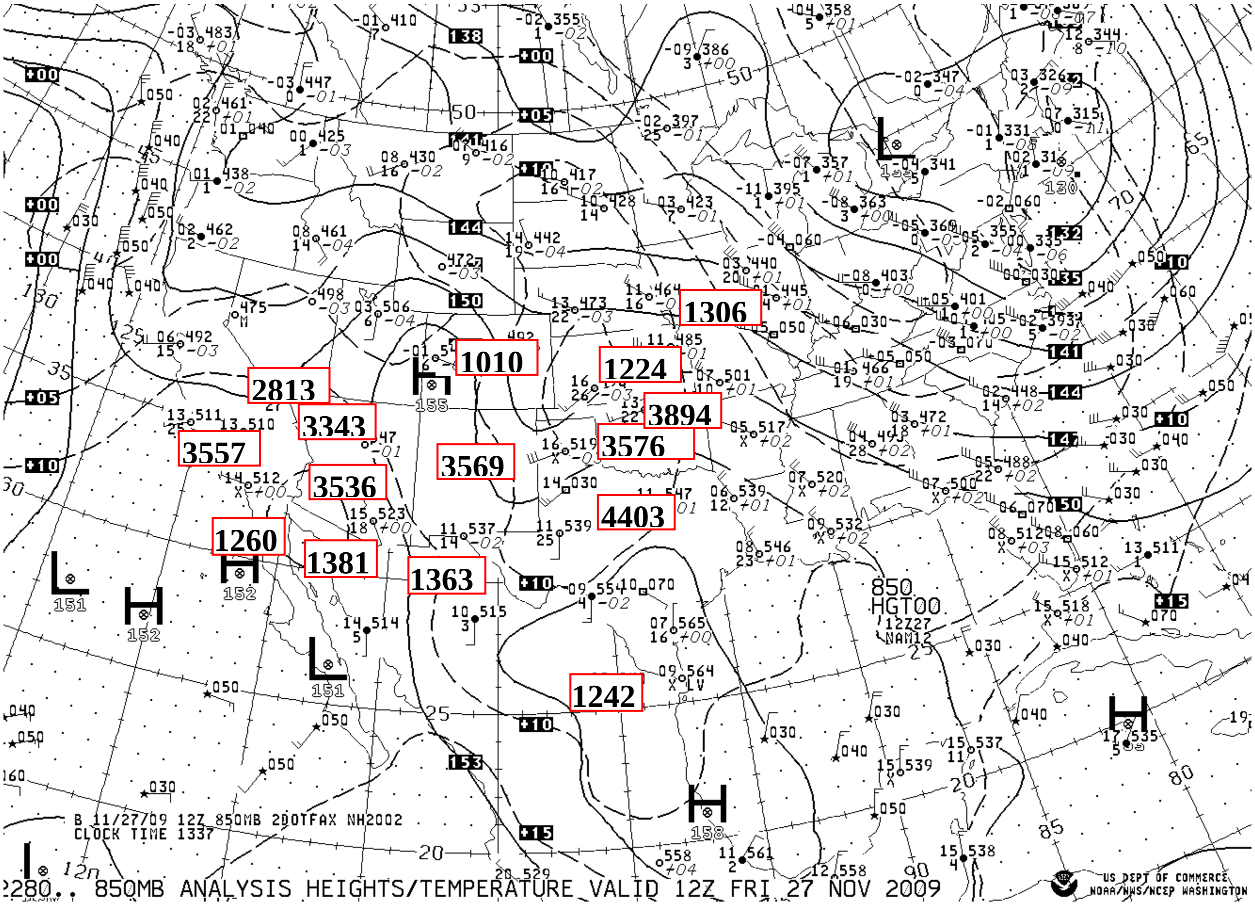

Figure 09G-i1-Used the 11/29/2009 week-ending Physician influenza-like illness report data, with the Nov 27 2009 12 z 850 mb chart. (Courtesy of NOAA NWS; Google Flu Trends).

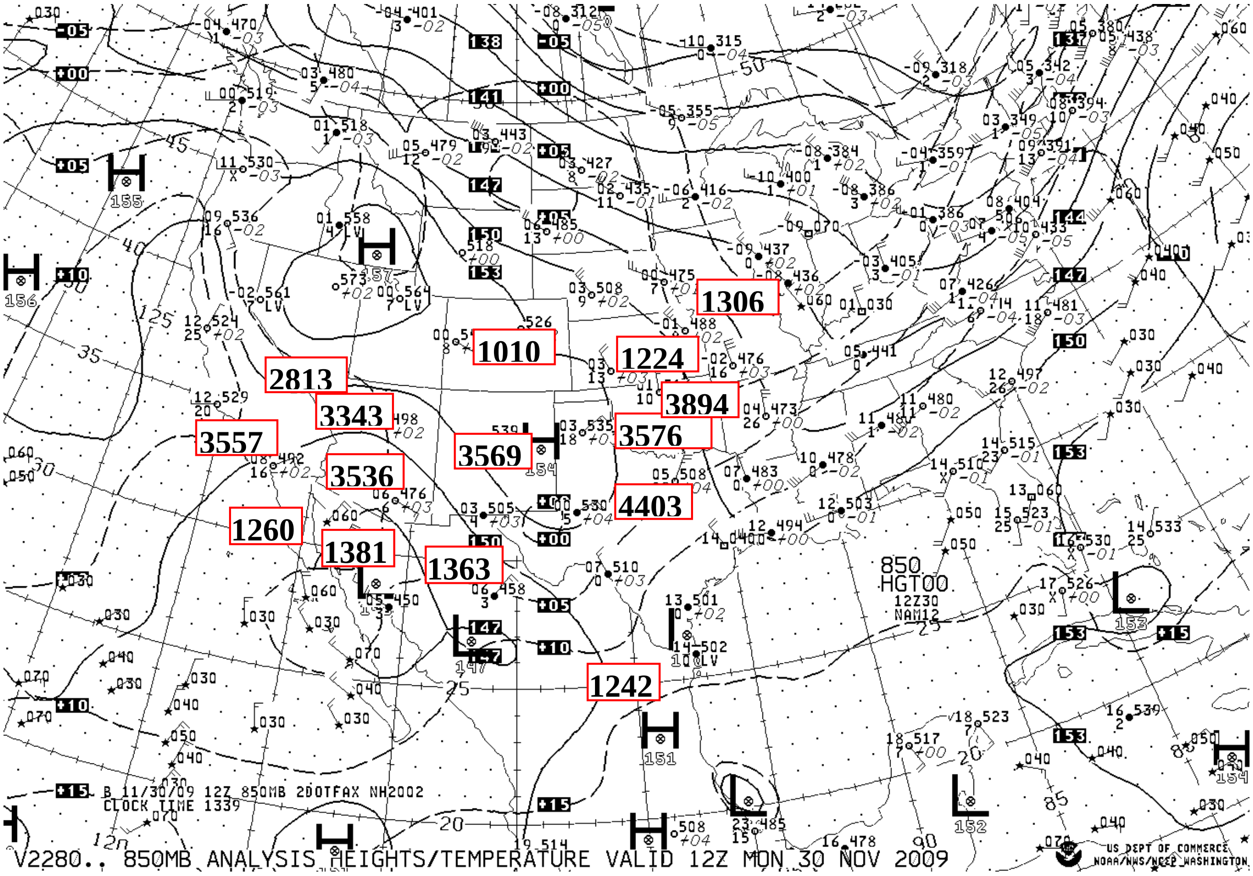

Figure 09G-i2-Used the 11/29/2009 week-ending Physician influenza-like illness report data, with the Nov 30 2009 12 z 850 mb chart. (Courtesy of NOAA NWS; Google Flu Trends).

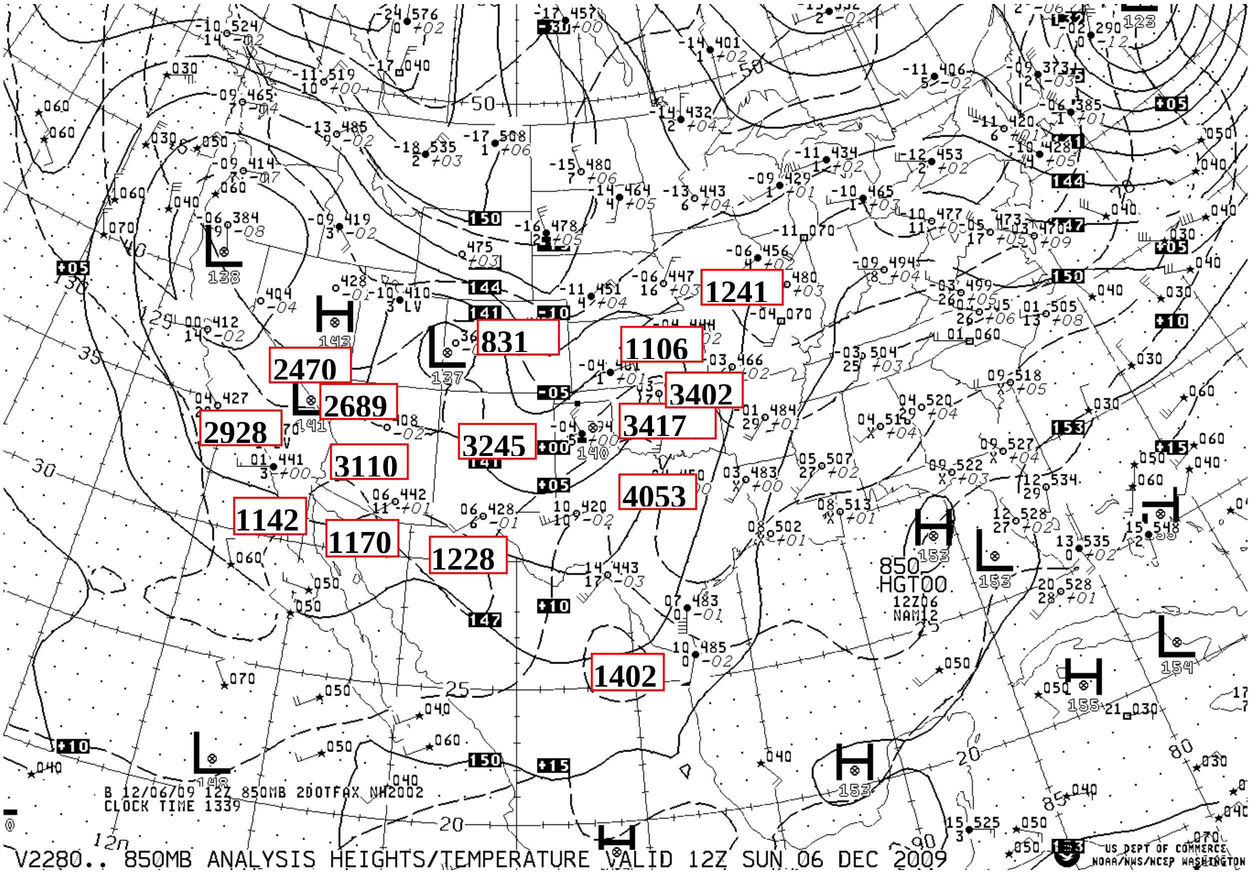

Figure 09G-j-Used the 12/06/2009 week-ending Physician influenza-like illness report data, with the Dec 06 2009 12 z 850 mb chart. (Courtesy of NOAA NWS; Google Flu Trends).

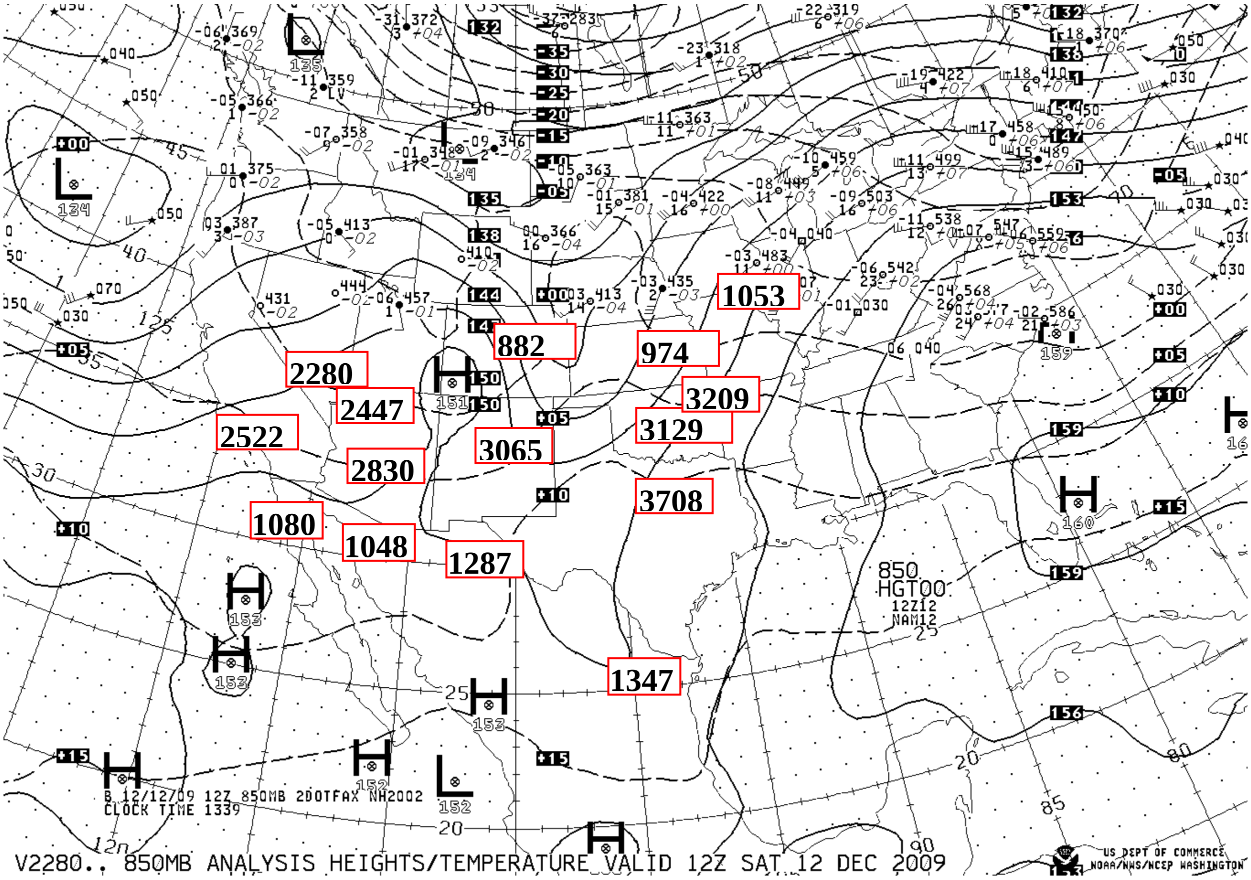

Figure 09G-k-Used the 12/13/2009 week-ending Physician influenza-like illness report data, with the Dec 12 2009 12 z 850 mb chart. (Courtesy of NOAA NWS; Google Flu Trends).

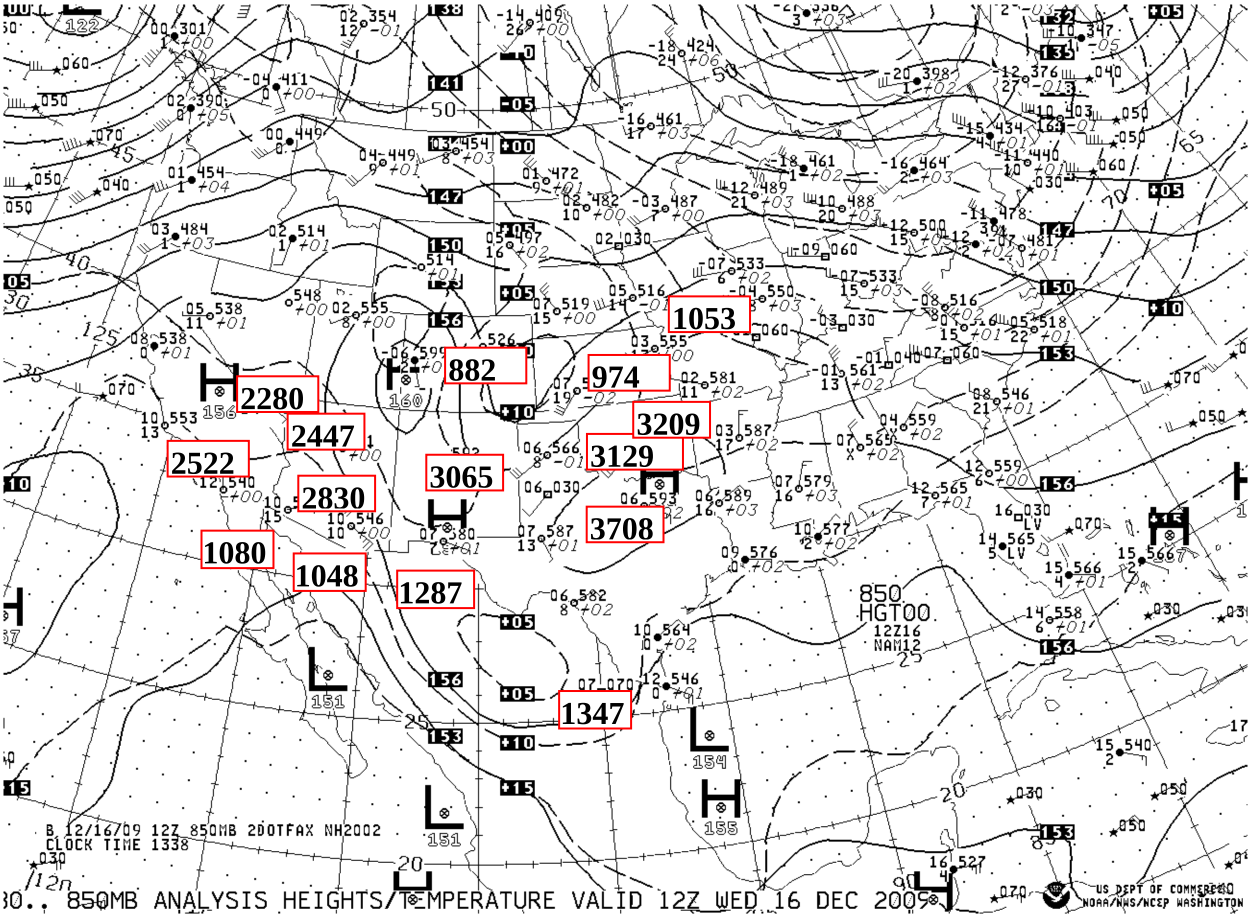

Figure 09G-l-Used the 12/13/2009 week-ending Physician influenza-like illness report data, with the Dec 16 2009 12 z 850 mb chart. (Courtesy of NOAA NWS; Google Flu Trends).

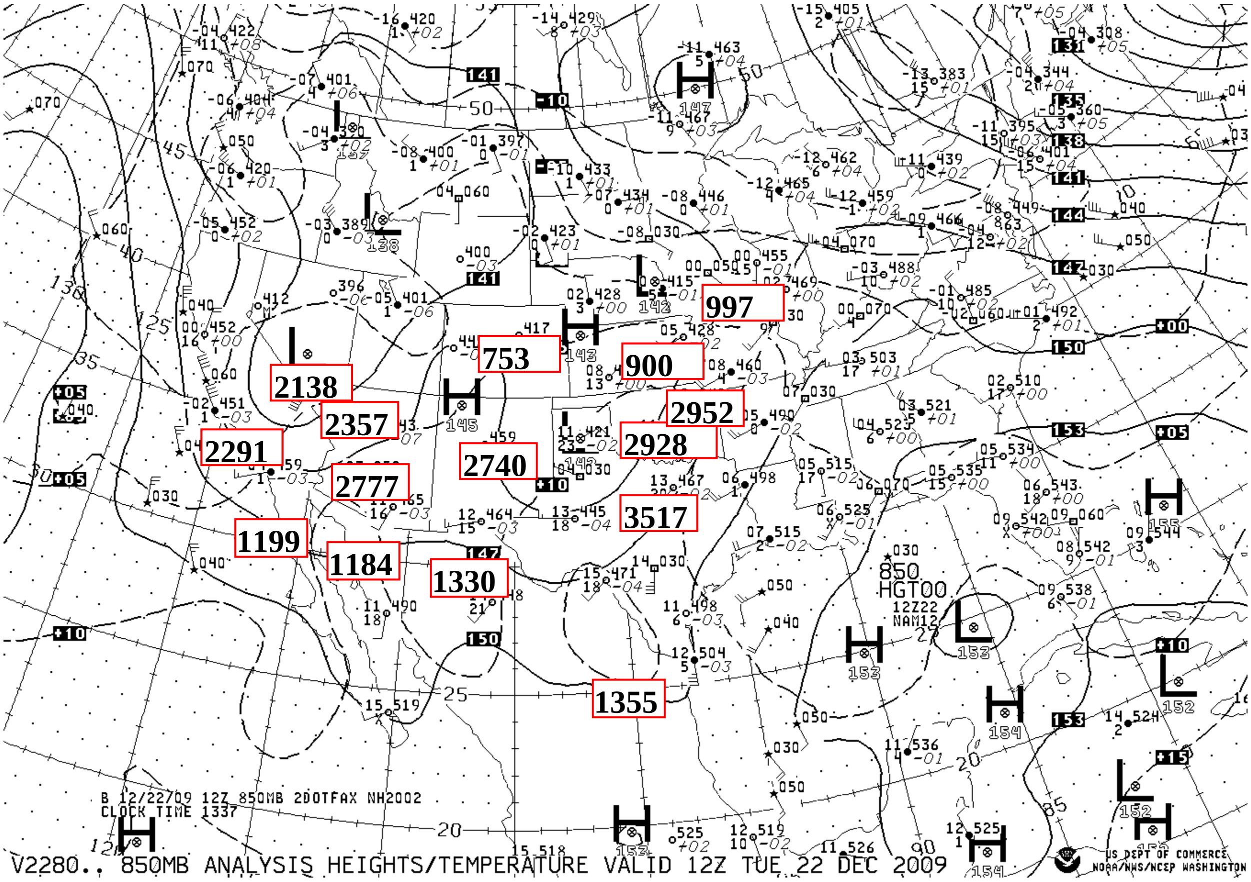

Figure 09G-m-Used the 12/20/2009 week-ending Physician influenza-like illness report data, with the Dec 22 2009 12 z 850 mb chart. (Courtesy of NOAA NWS; Google Flu Trends).

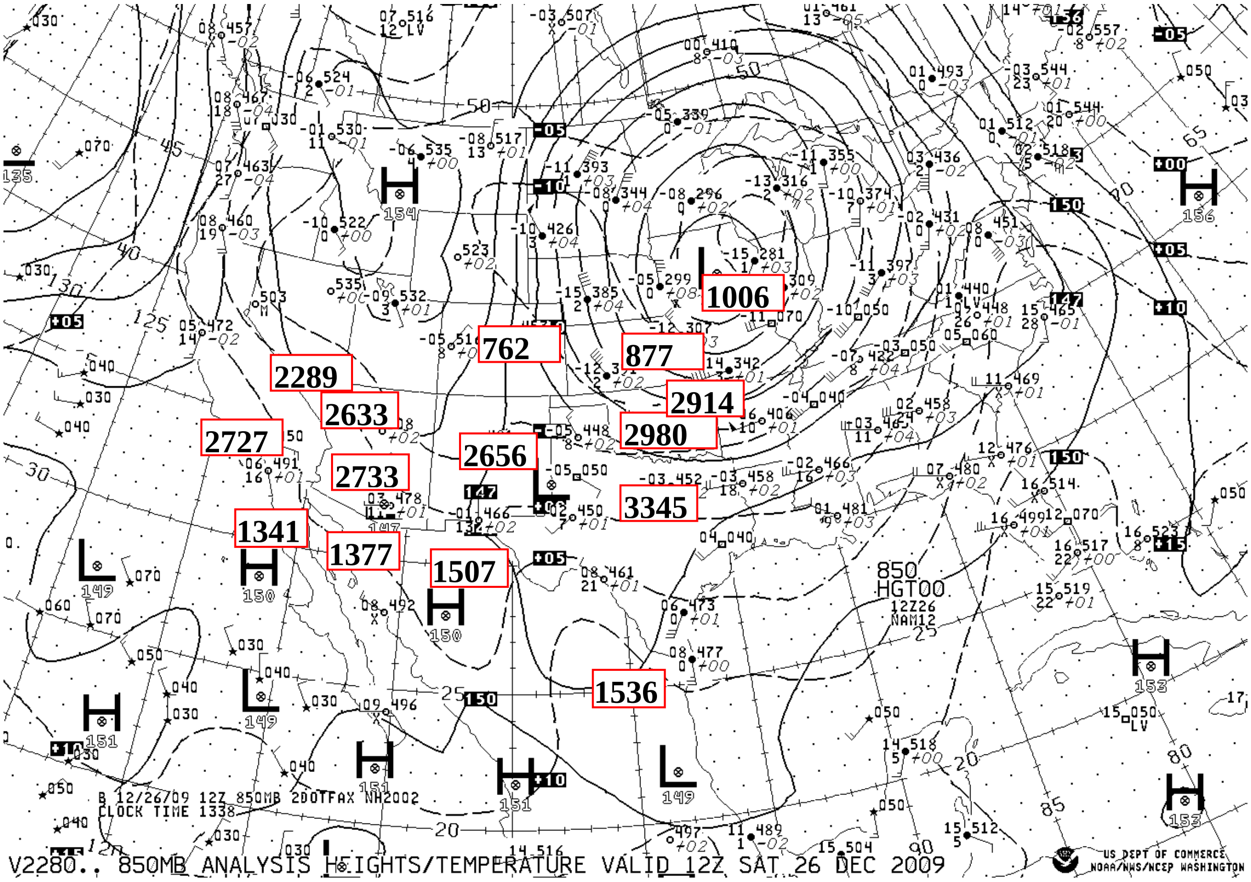

Figure 09G-n1-Used the 12/27/2009 week-ending Physician influenza-like illness report data, with the Dec 26 2009 12 z 850 mb chart. (Courtesy of NOAA NWS; Google Flu Trends).

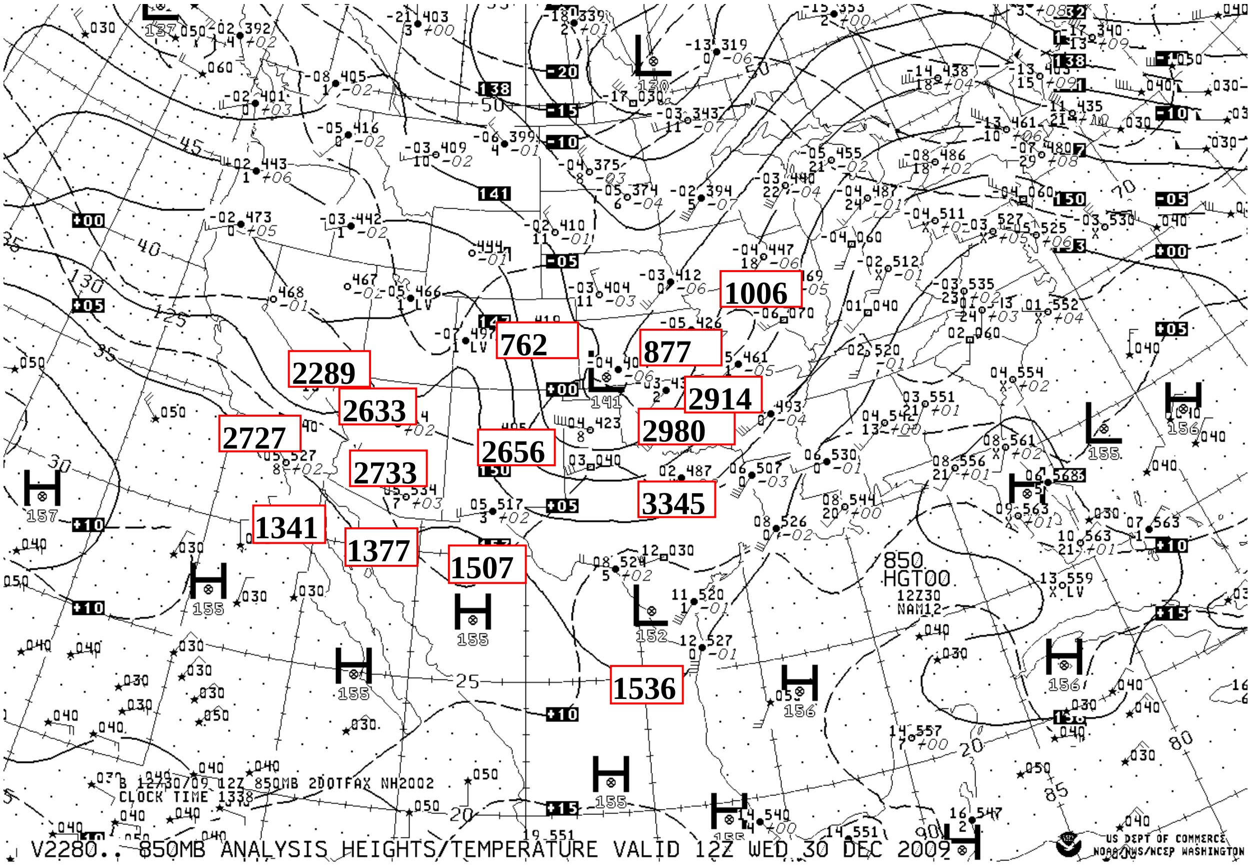

Figure 09G-n2-Used the 12/27/2009 week-ending Physician influenza-like illness report data, with the Dec 30 2009 12 z 850 mb chart. (Courtesy of NOAA NWS; Google Flu Trends).

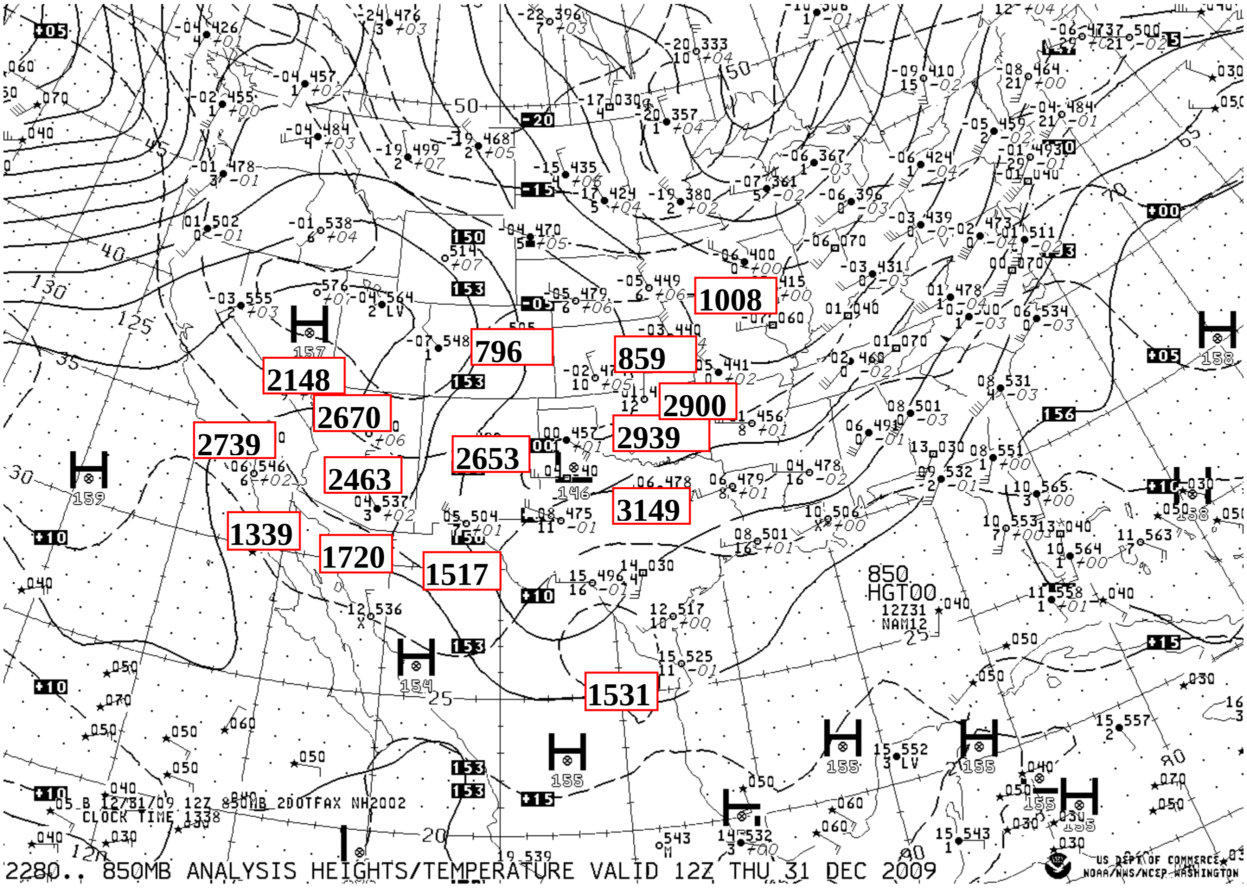

Figure 09G-o-Used the 01/03/2010 week-ending Physician influenza-like illness report data, with the Dec 31 2009 12 z 850 mb chart. (Courtesy of NOAA NWS; Google Flu Trends).

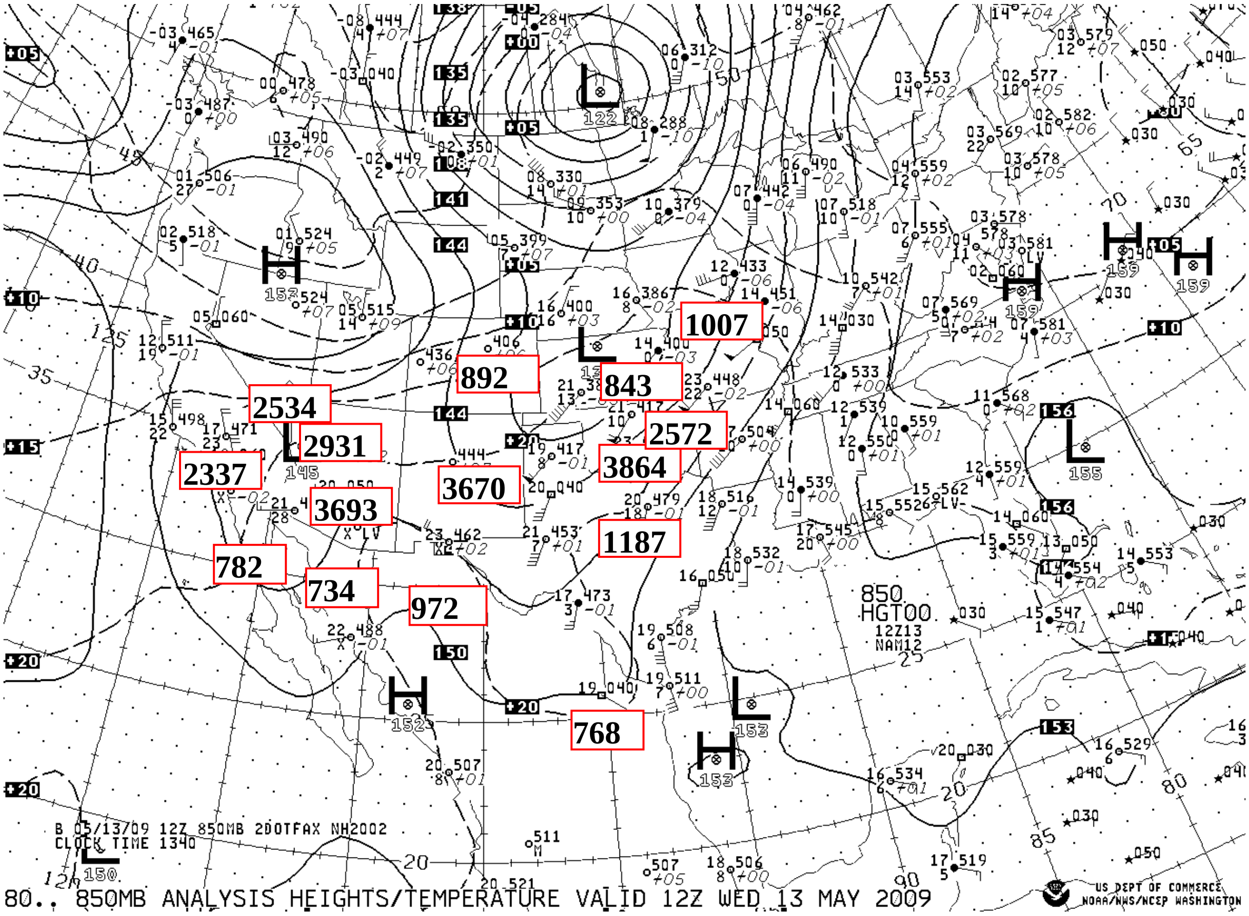

Figure 09H-a-Used the 05/10/2009 week-ending Physician influenza-like illness report data, with the May 13 2009 12 z 850 mb chart. (Courtesy of NOAA NWS; Google Flu Trends).

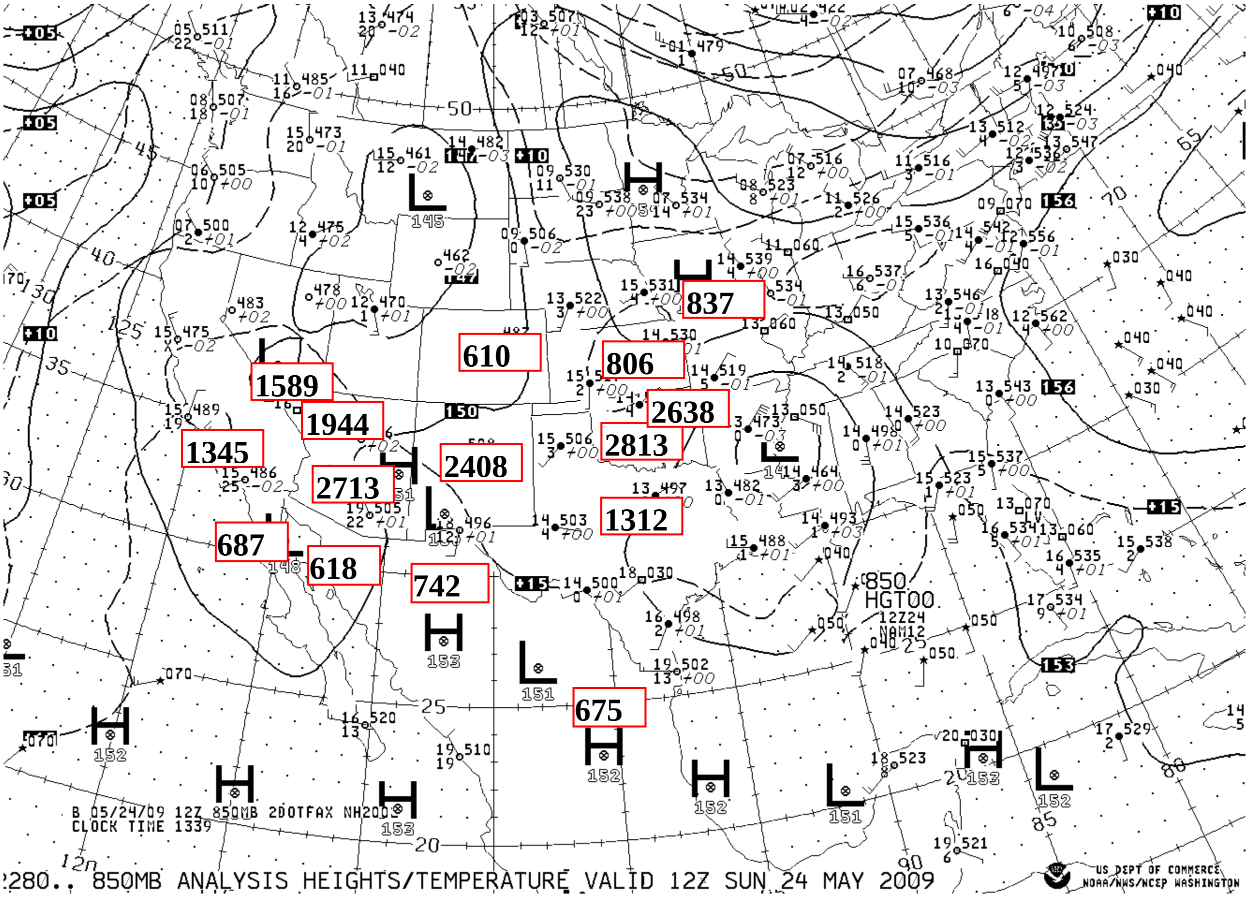

Figure 09H-b-Used the 05/24/2009 week-ending Physician influenza-like illness report data, with the May 24 2009 12 z 850 mb chart. (Courtesy of NOAA NWS; Google Flu Trends).

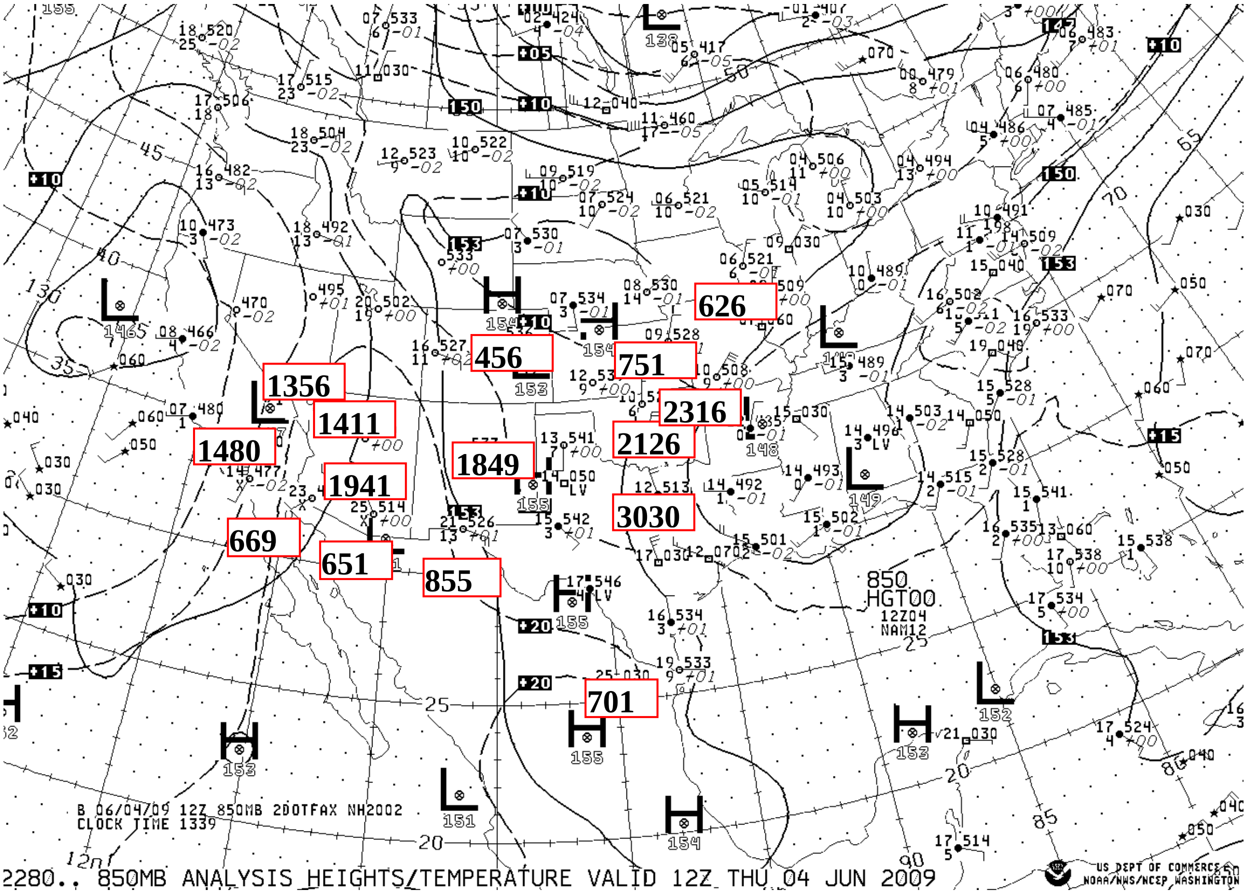

Figure 09H-b1-Used the 06/07/2009 week-ending Physician influenza-like illness report data, with the Jun 04 2009 12 z 850 mb chart. (Courtesy of NOAA NWS; Google Flu Trends).

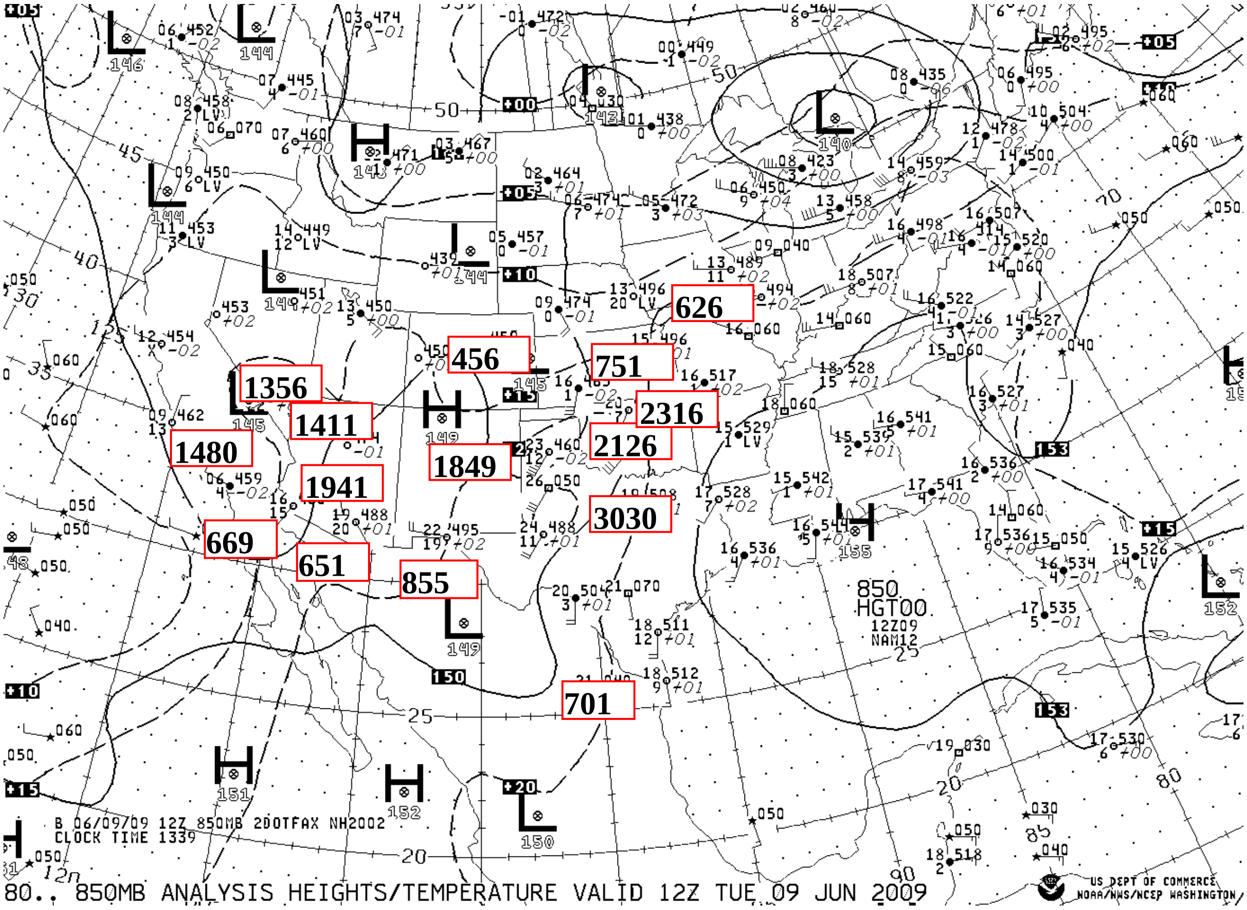

Figure 09H-b2-Used the 06/07/2009 week-ending Physician influenza-like illness report data, with the Jun 09 2009 12 z 850 mb chart. (Courtesy of NOAA NWS; Google Flu Trends).

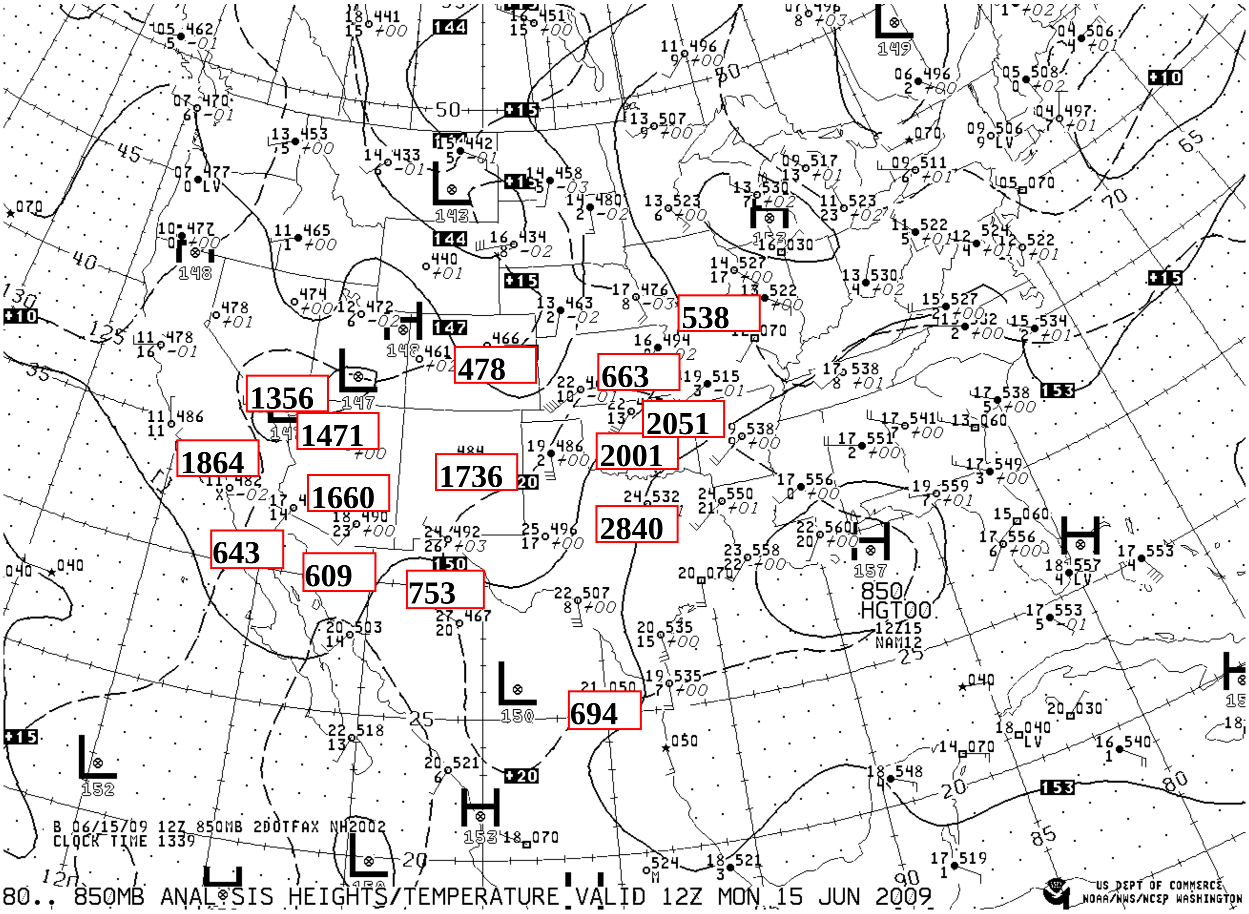

Figure 09H-c-Used the 06/14/2009 week-ending Physician influenza-like illness report data, with the Jun 15 2009 12 z 850 mb chart. (Courtesy of NOAA NWS; Google Flu Trends).

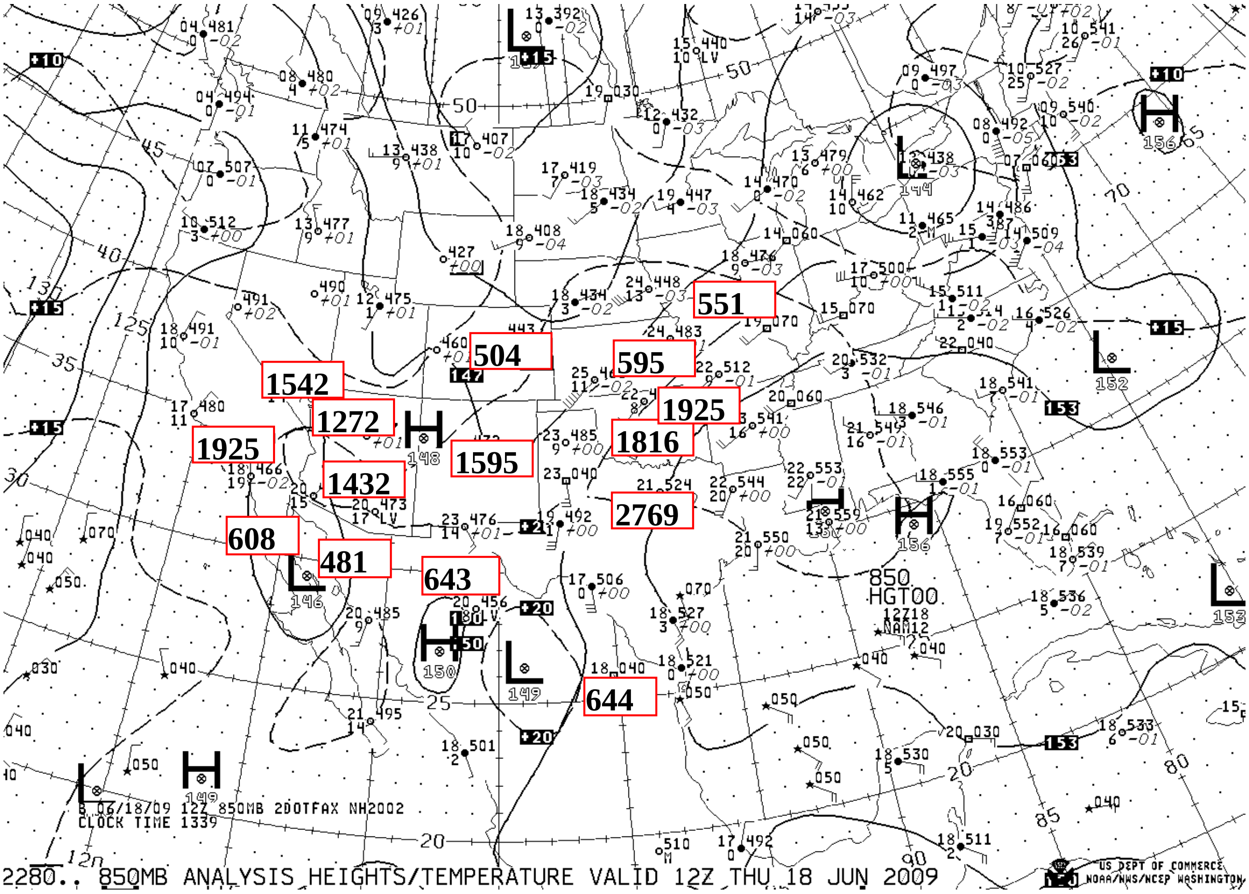

Figure 09H-d-Used the 06/21/2009 week-ending Physician influenza-like illness report data, with the Jun 18 2009 12 z 850 mb chart. (Courtesy of NOAA NWS; Google Flu Trends).

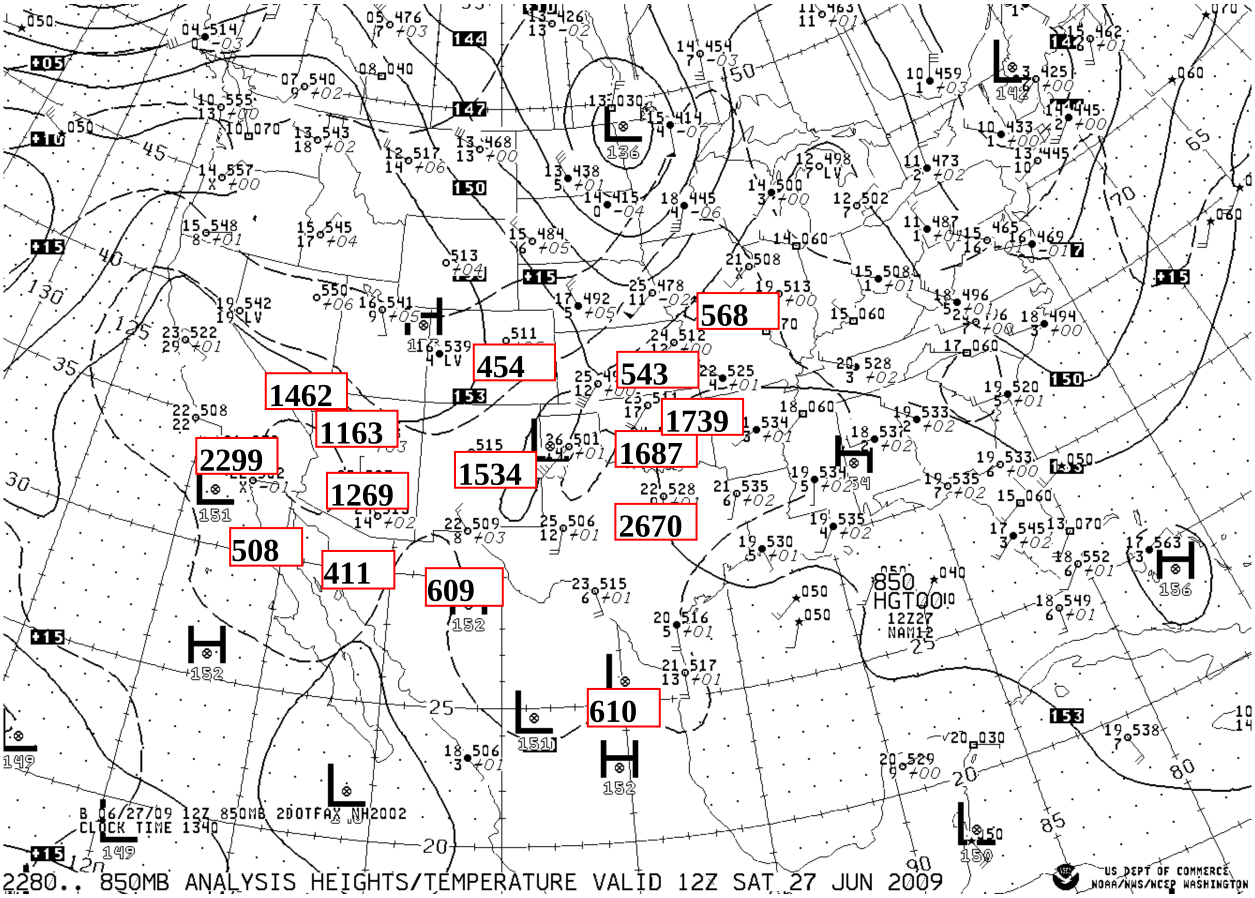

Figure 09H-e-Used the 06/28/2009 week-ending Physician influenza-like illness report data, with the Jun 27 2009 12 z 850 mb chart. (Courtesy of NOAA NWS; Google Flu Trends).

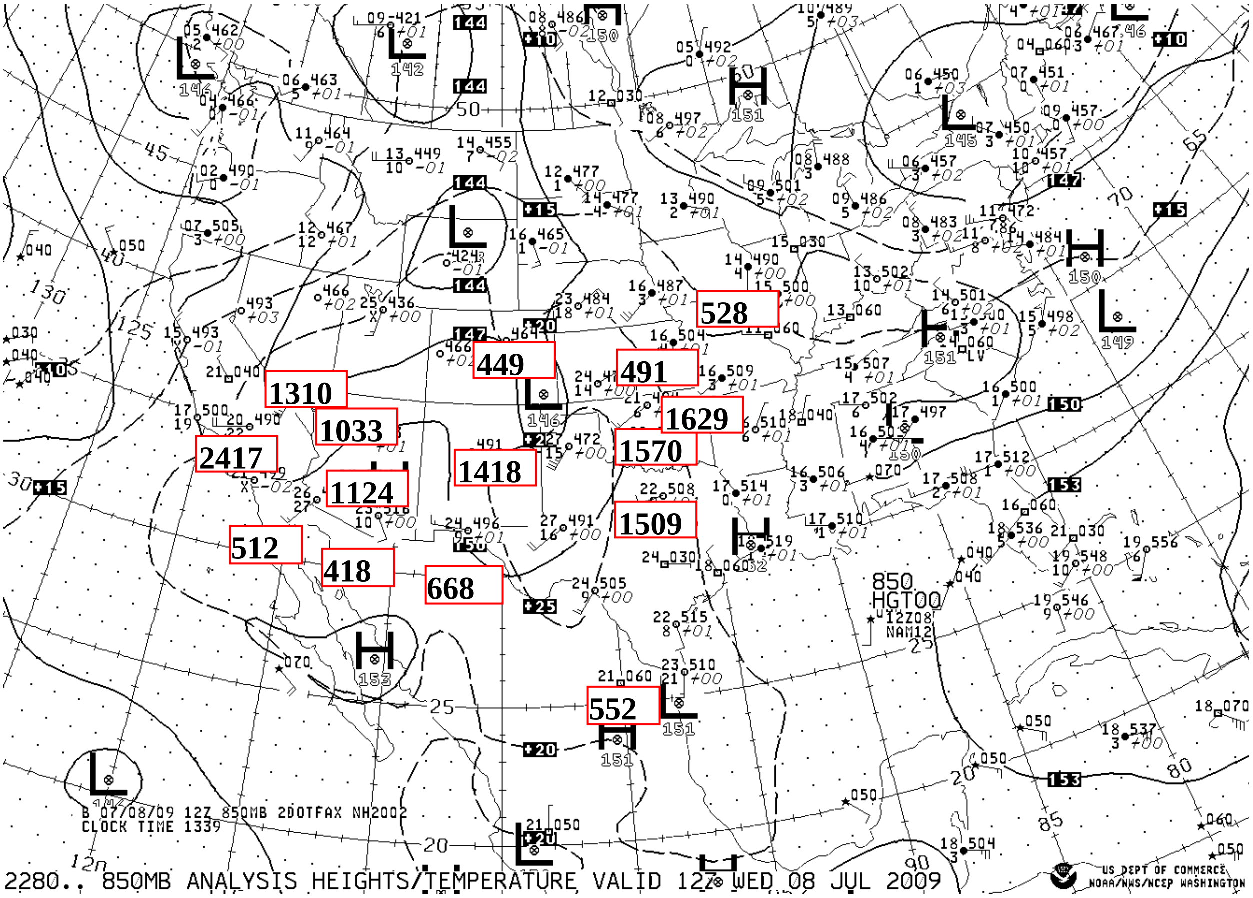

Figure 09H-f-Used the 07/05/2009 week-ending Physician influenza-like illness report data, with the Jul 08 2009 12 z 850 mb chart. (Courtesy of NOAA NWS; Google Flu Trends).

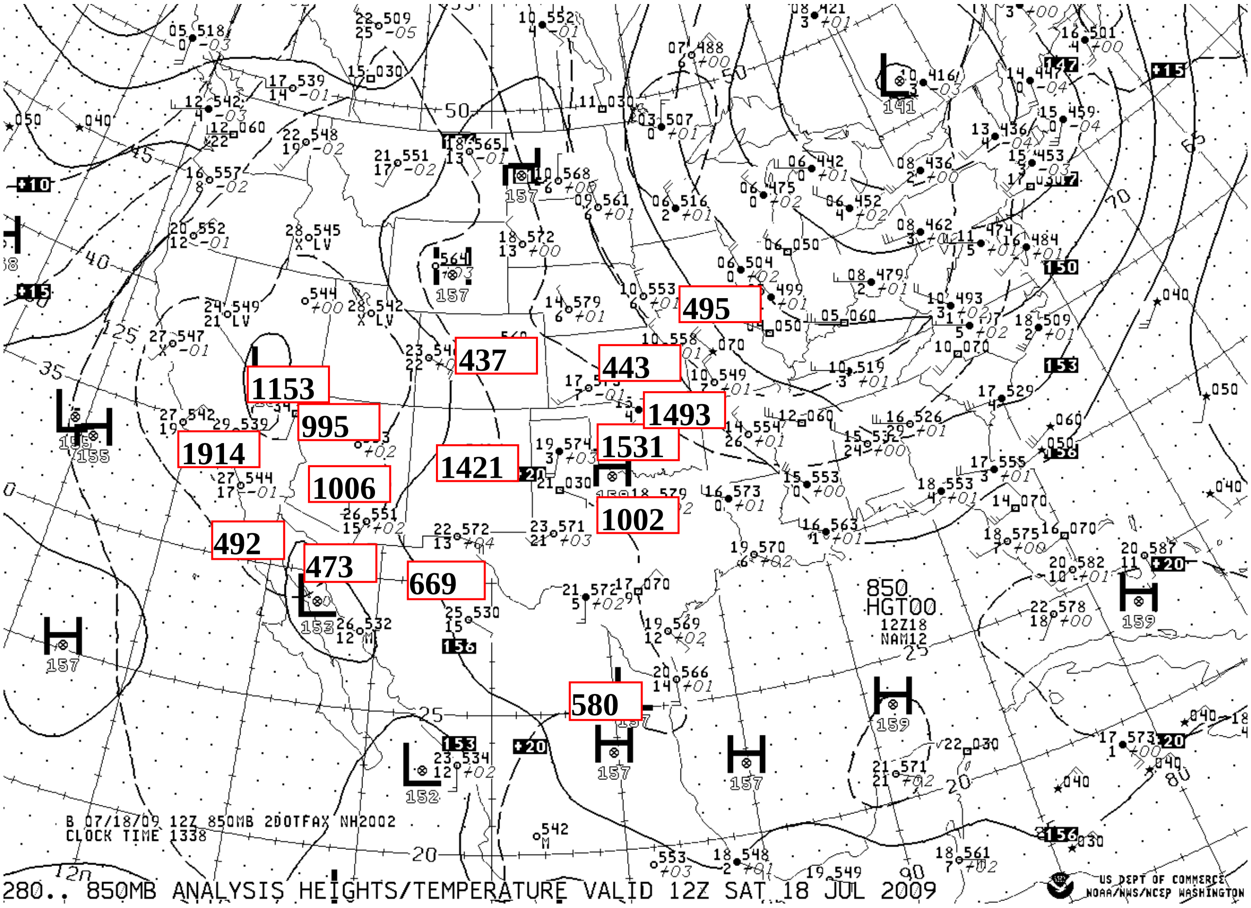

Figure 09H-g-Used the 07/19/2009 week-ending Physician influenza-like illness report data, with the Jul 18 2009 12 z 850 mb chart. (Courtesy of NOAA NWS; Google Flu Trends).

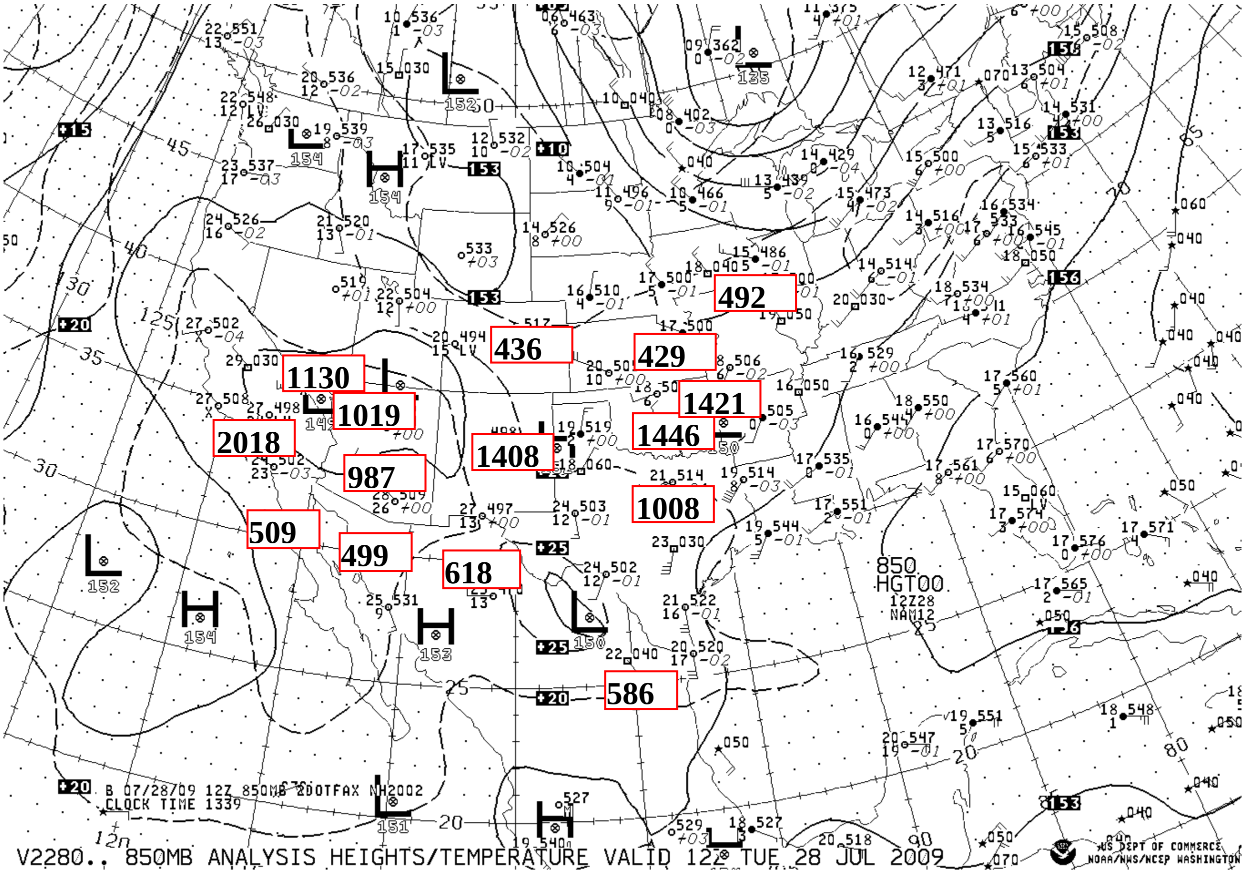

Figure 09H-h-Used the 07/26/2009 week-ending Physician influenza-like illness report data, with the Jul 28 2009 12 z 850 mb chart. (Courtesy of NOAA NWS; Google Flu Trends).

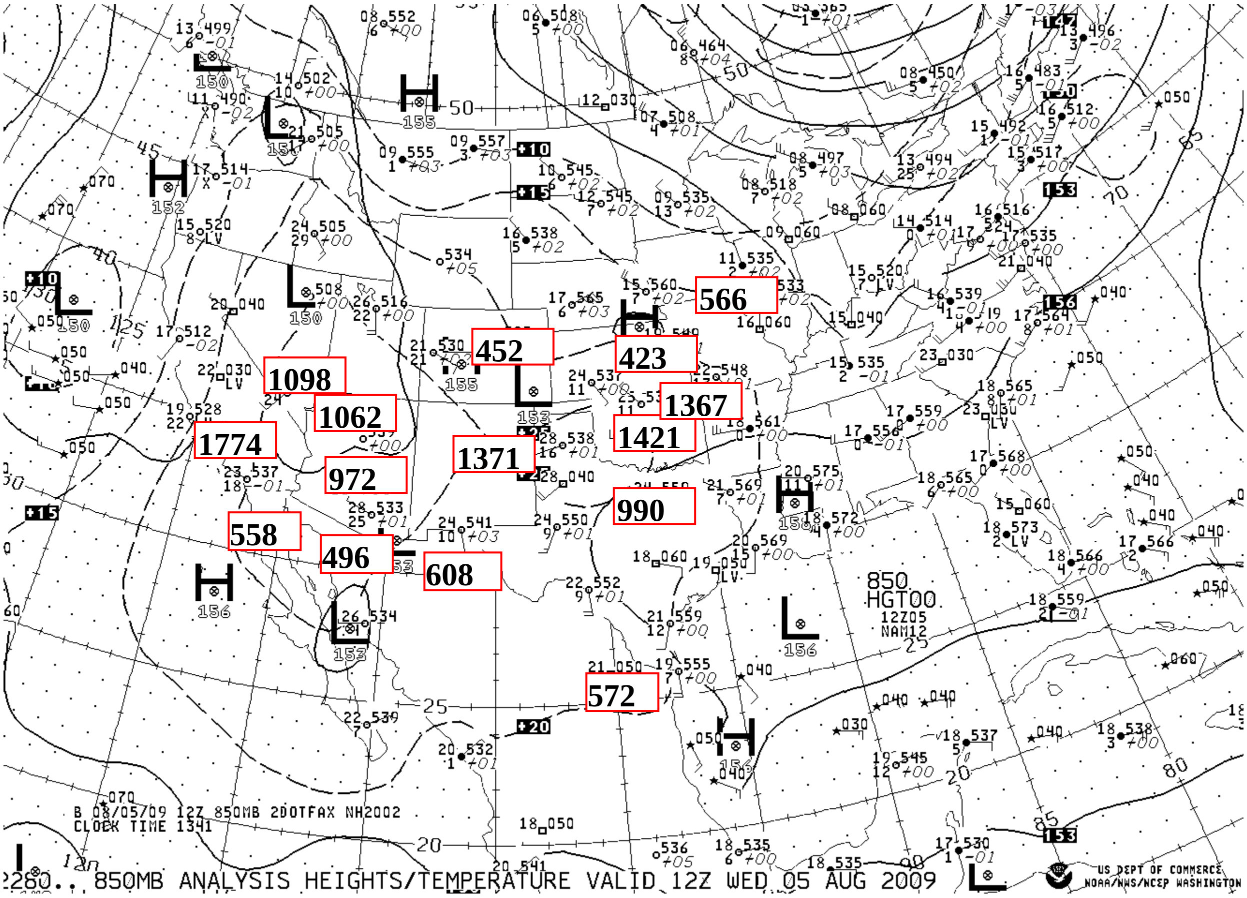

Figure 09H-i-Used the 08/02/2009 week-ending Physician influenza-like illness report data, with the Aug 05 2009 12 z 850 mb chart. (Courtesy of NOAA NWS; Google Flu Trends).

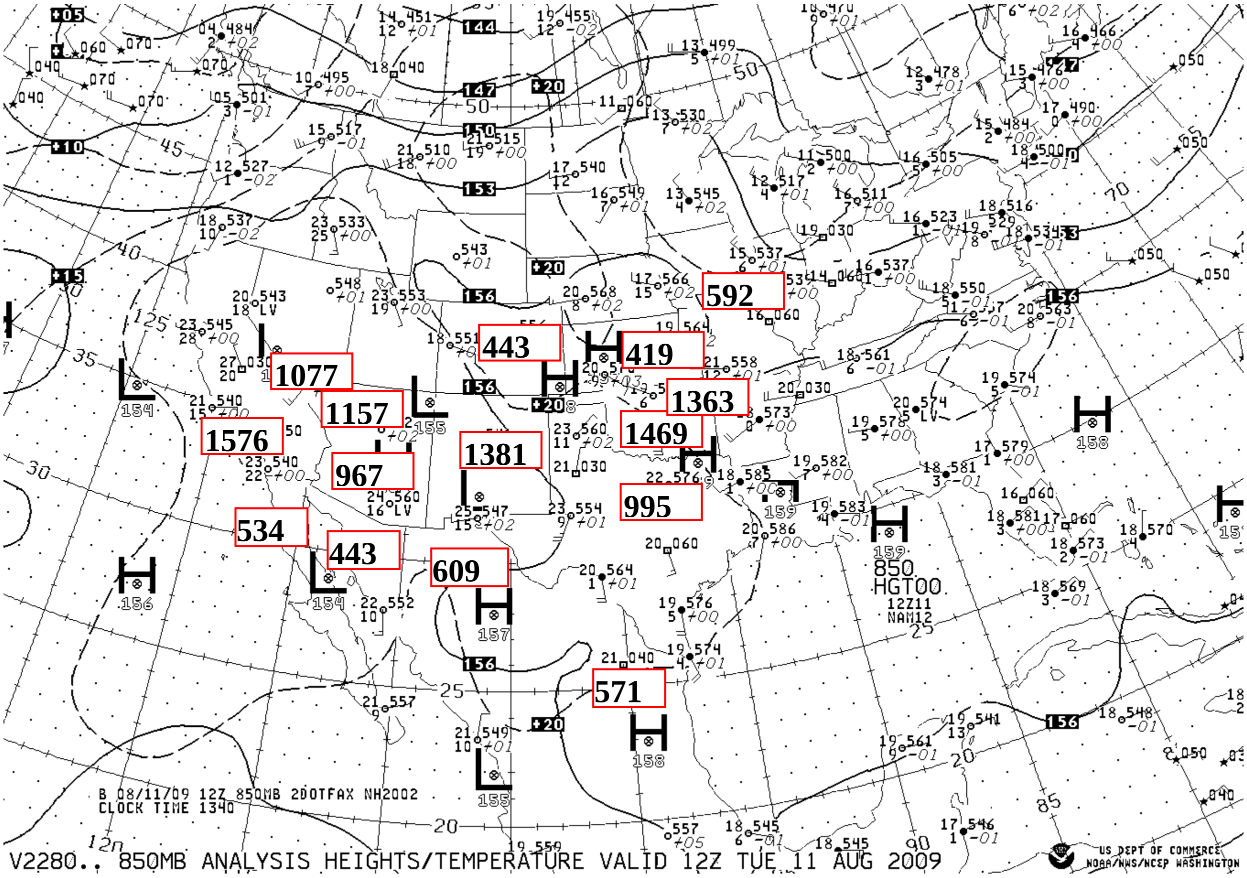

Figure 09H-j-Used the 08/09/2009 week-ending Physician influenza-like illness report data, with the Aug 11 2009 12 z 850 mb chart. (Courtesy of NOAA NWS; Google Flu Trends).

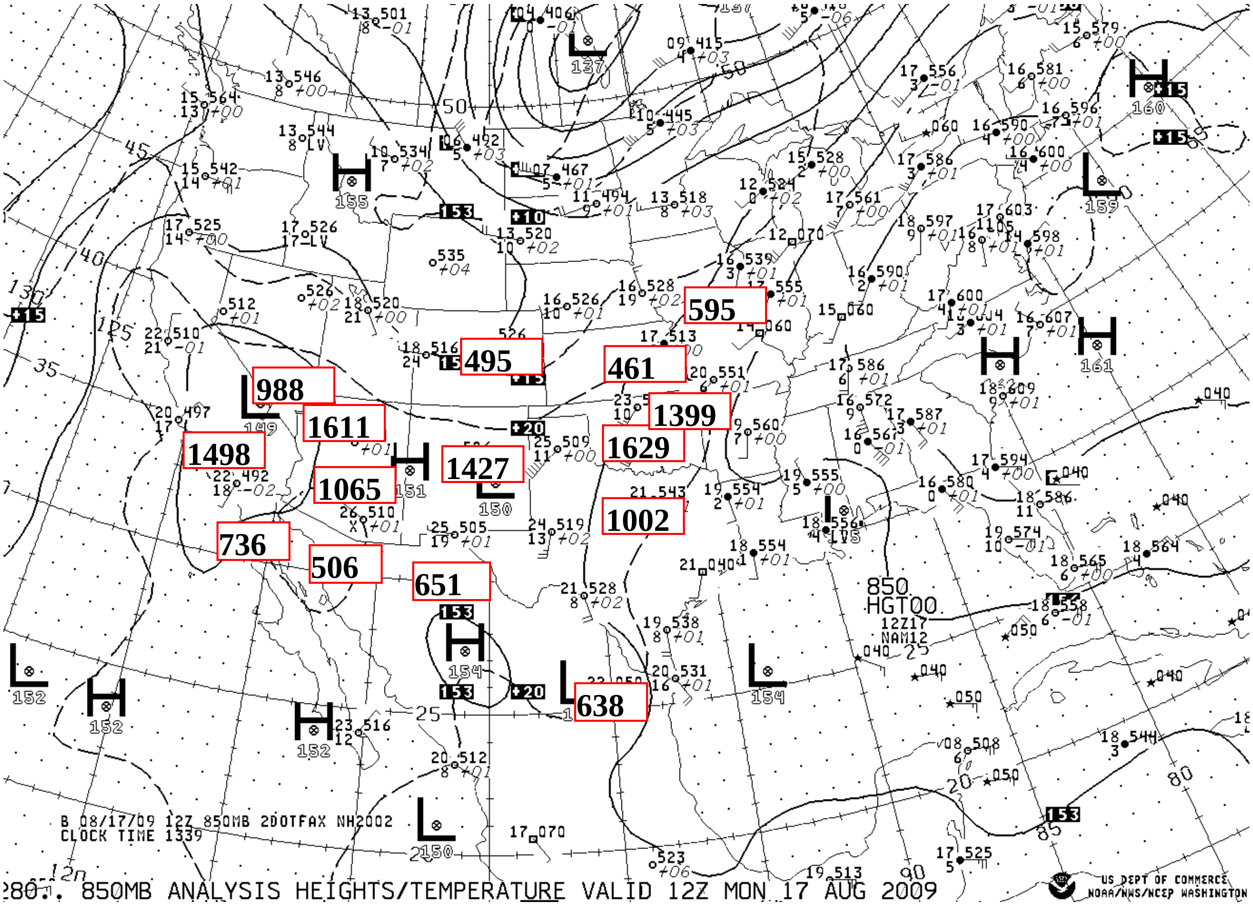

Figure 09H-k-Used the 08/16/2009 week-ending Physician influenza-like illness report data, with the Aug 17 2009 12 z 850 mb chart. (Courtesy of NOAA NWS; Google Flu Trends).

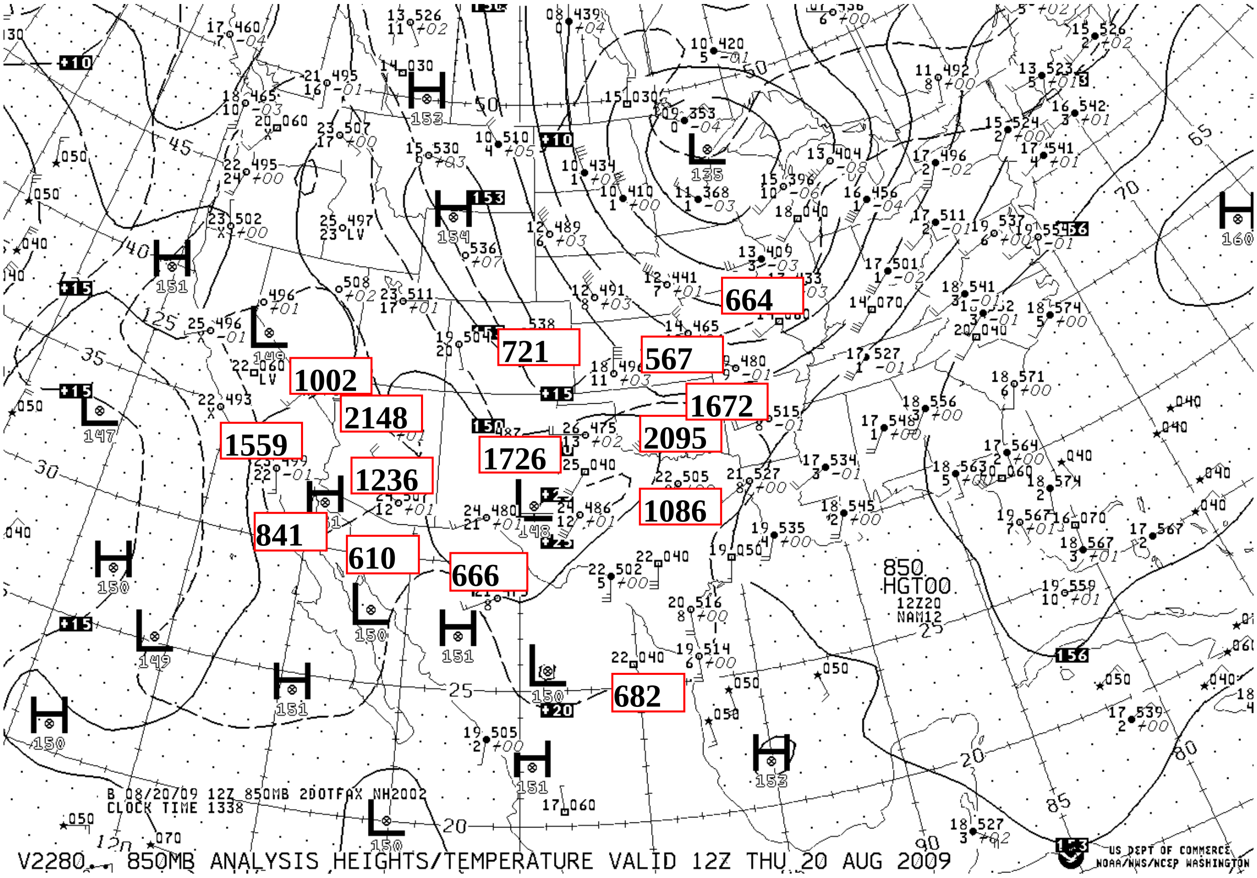

Figure 09H-l-Used the 08/23/2009 week-ending Physician influenza-like illness report data, with the Aug 20 2009 12 z 850 mb chart. (Courtesy of NOAA NWS; Google Flu Trends).

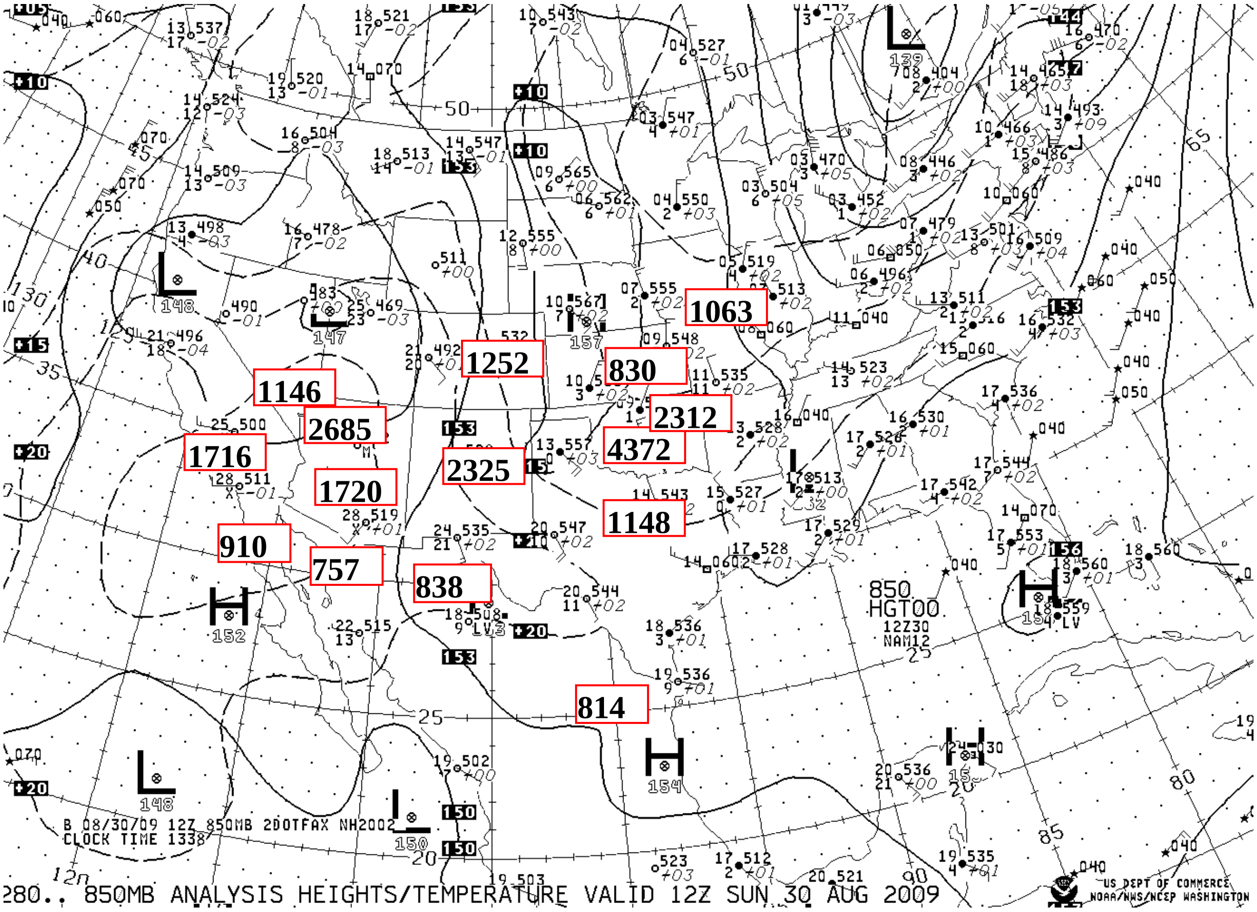

Figure 09H-m1-Used the 08/30/2009 week-ending Physician influenza-like illness report data, with the Aug 30 2009 12 z 850 mb chart. (Courtesy of NOAA NWS; Google Flu Trends).

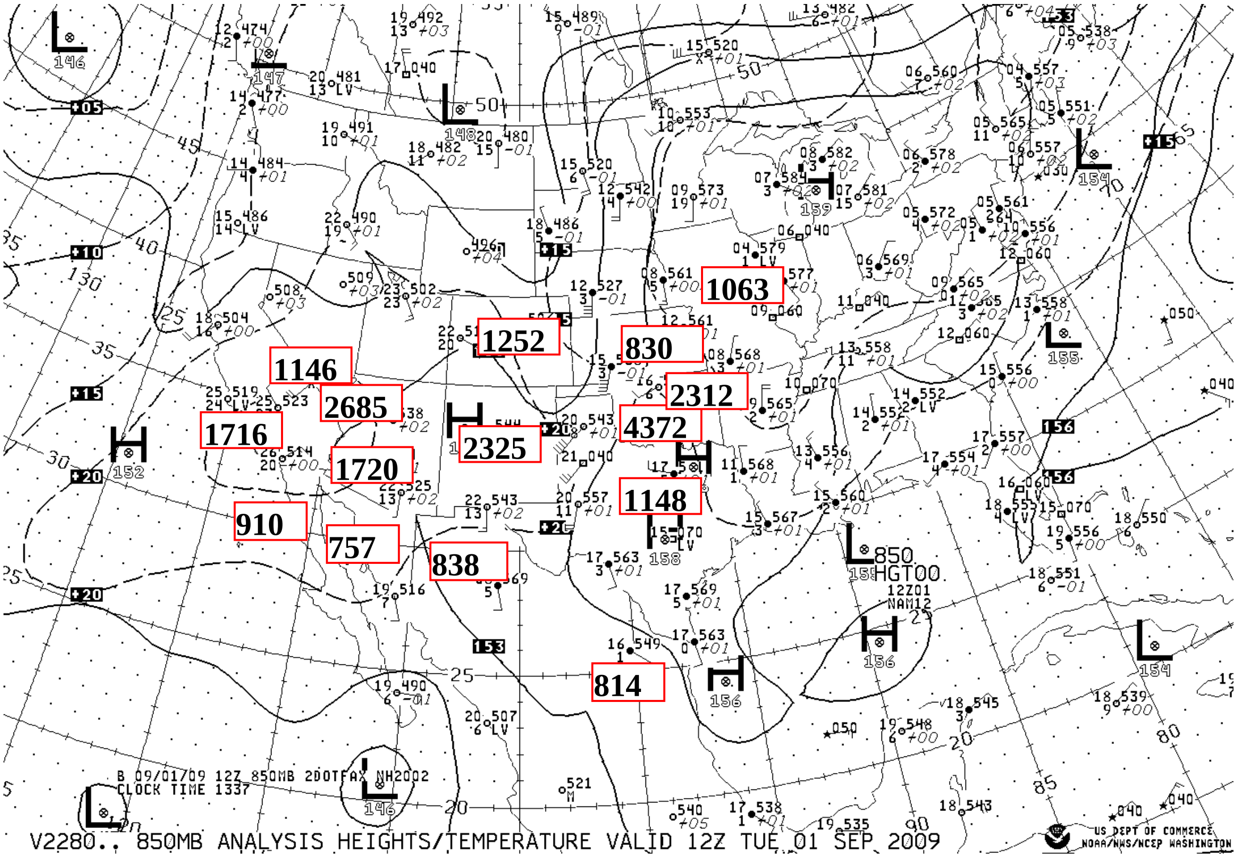

Figure 09H-m2-Used the 08/30/2009 week-ending Physician influenza-like illness report data, with the Sep 01 2009 12 z 850 mb chart. (Courtesy of NOAA NWS; Google Flu Trends).

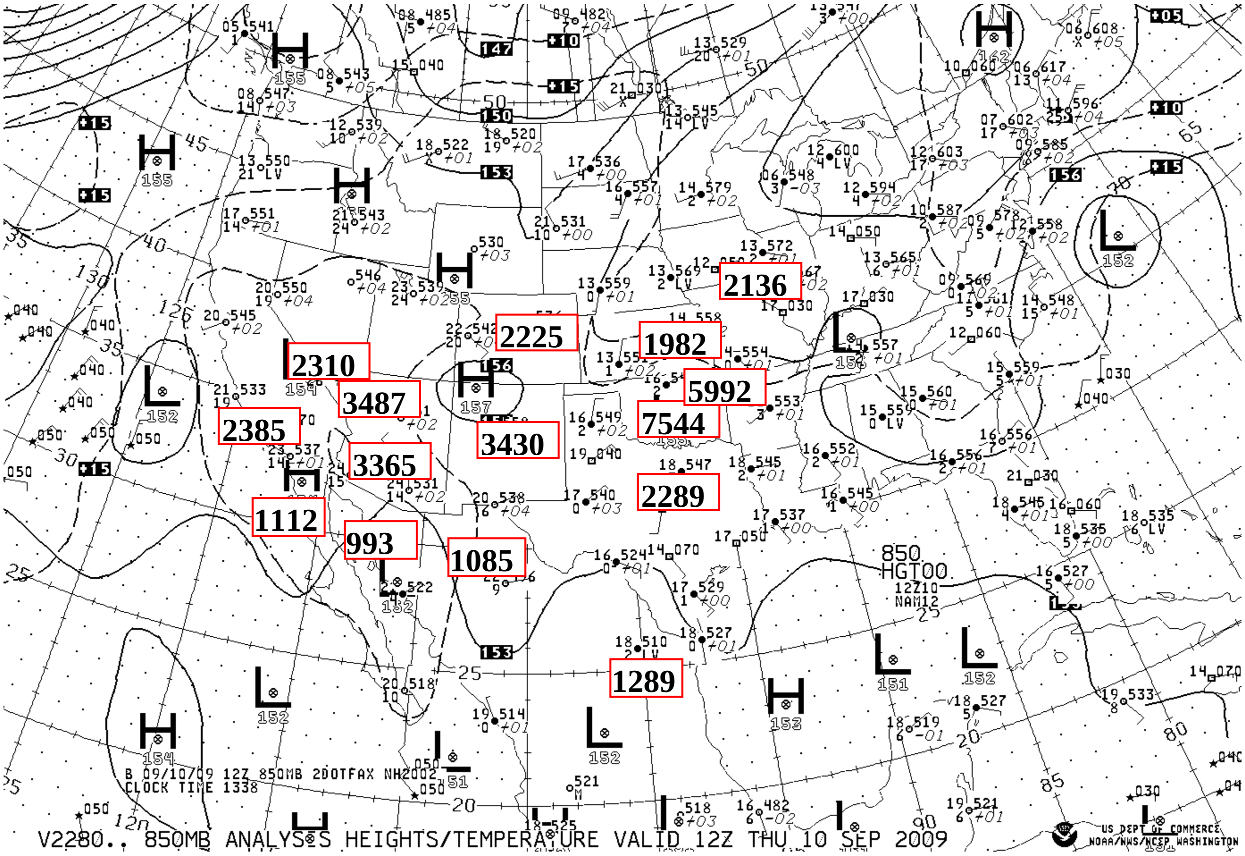

Figure 09H-n-Used the 09/13/2009 week-ending Physician influenza-like illness report data, with the Sep 10 2009 12 z 850 mb chart. (Courtesy of NOAA NWS; Google Flu Trends).

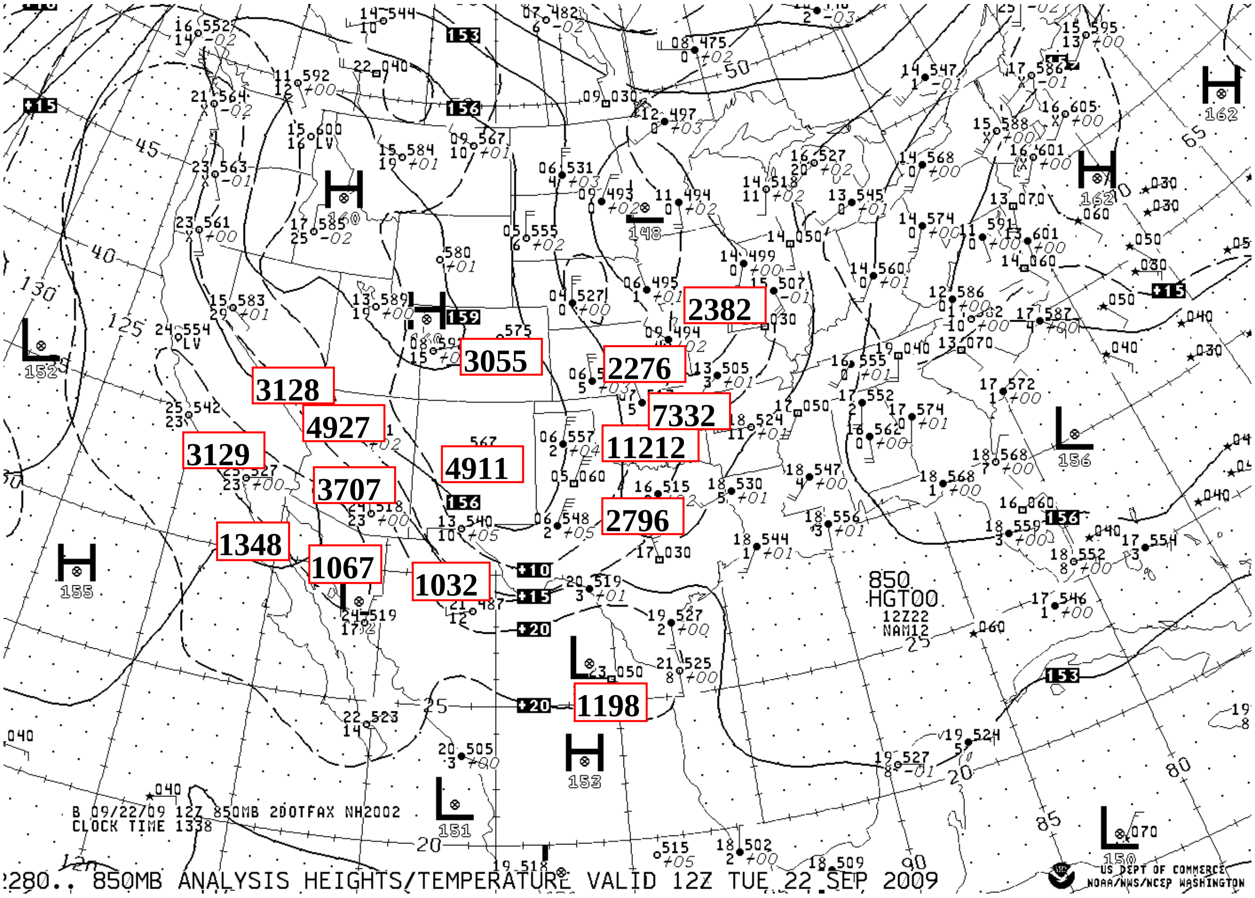

Figure 09H-o-Used the 09/20/2009 week-ending Physician influenza-like illness report data, with the Sep 22 2009 12 z 850 mb chart. (Courtesy of NOAA NWS; Google Flu Trends).

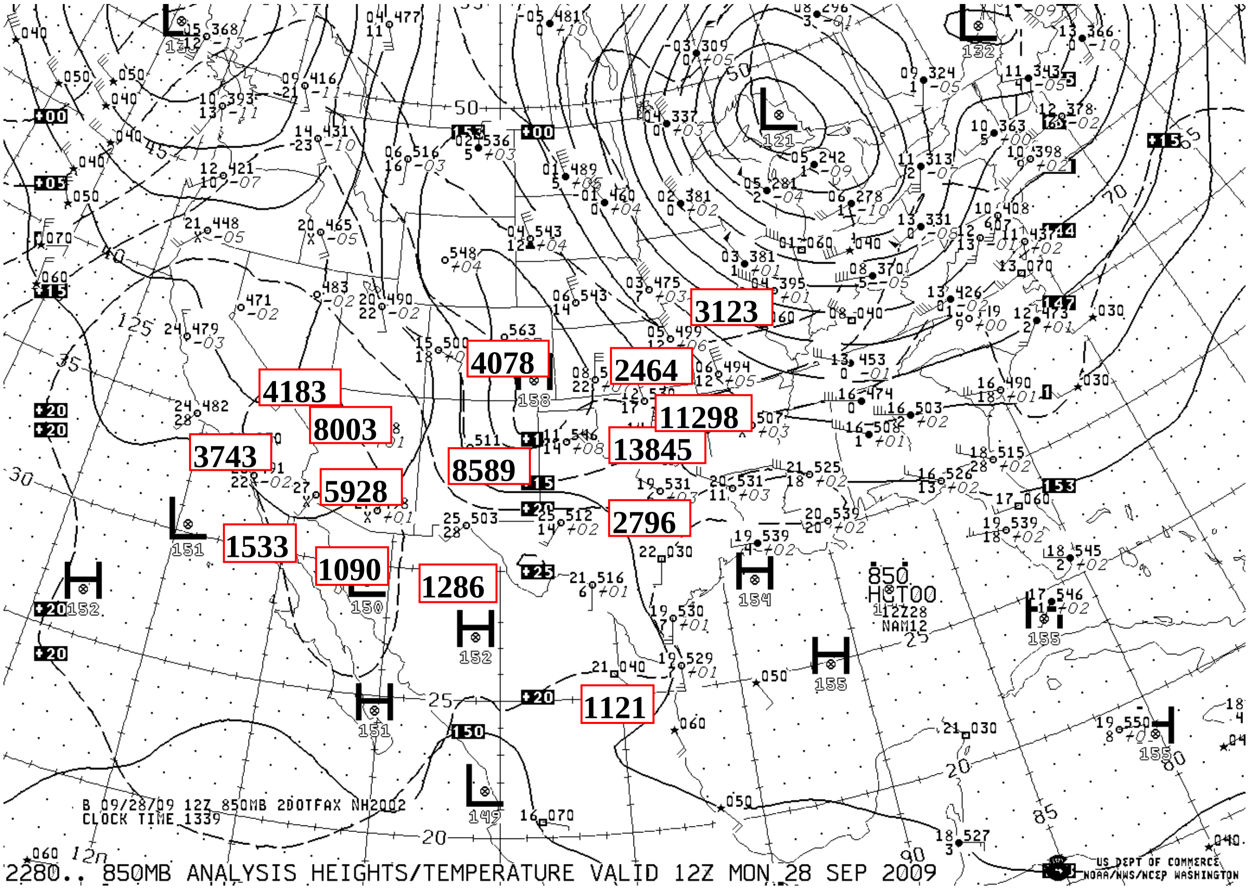

Figure 09H-p-Used the 09/27/2009 week-ending Physician influenza-like illness report data, with the Sep 28 2009 12 z 850 mb chart. (Courtesy of NOAA NWS; Google Flu Trends).
